# Computational Study of the Fries Rearrangement Catalyzed by Acyltransferase from *Pseudomonas protegens*

**DOI:** 10.1002/open.202300256

**Published:** 2024-01-15

**Authors:** Xiang Sheng, Wolfgang Kroutil, Fahmi Himo

**Affiliations:** ^1^ Tianjin Institute of Industrial Biotechnology Chinese Academy of Sciences Tianjin 300308 P.R. China; ^2^ National Center of Technology Innovation for Synthetic Biology National Engineering Research Center of Industrial Enzymes Tianjin 300308 P.R. China; ^3^ Institute of Chemistry NAWI Graz University of Graz 8010 Graz Austria; ^4^ Field of Excellence BioHealth BioTechMed Graz 8010 Graz Austria; ^5^ Department of Organic Chemistry Arrhenius Laboratory Stockholm University SE-10691 Stockholm Sweden

**Keywords:** acyltransferase, biocatalysis, Fries rearrangement, reaction mechanism, cluster approach

## Abstract

The acyltransferase from *Pseudomonas protegens* (*Pp*ATase) catalyzes in nature the reversible transformation of monoacetylphloroglucinol to diacetylphloroglucinol and phloroglucinol. Interestingly, this enzyme has been shown to catalyze the promiscuous transformation of 3‐hydroxyphenyl acetate to 2′,4′‐dihydroxyacetophenone, representing a biological version of the Fries rearrangement. In the present study, we report a mechanistic investigation of this activity of *Pp*ATase using quantum chemical calculations. A detailed mechanism is proposed, and the energy profile for the reaction is presented. The calculations show that the acylation of the enzyme is highly exothermic, while the acetyl transfer back to the substrate is only slightly exothermic. The deprotonation of the C6−H of the substrate is rate‐limiting, and a remote aspartate residue (Asp137) is proposed to be the general base group in this step. Analysis of the binding energies of various acetyl acceptors shows that *Pp*ATase can promote both intramolecular and intermolecular Fries rearrangement towards diverse compounds.

## Graphical abstract

Density functional theory (DFT) calculations are employed to uncover the detailed reaction mechanism for the Fries rearrangement of 3‐hydroxyphenyl acetate to 2′,4′‐dihydroxyacetophenone catalyzed by the acyltransferase from *Pseudomonas protegens* (*Pp*ATase). Relative binding energies of other acetyl acceptors are also calculated to evaluate the possibility of *Pp*ATase catalyzing an intermolecular Fries rearrangement.

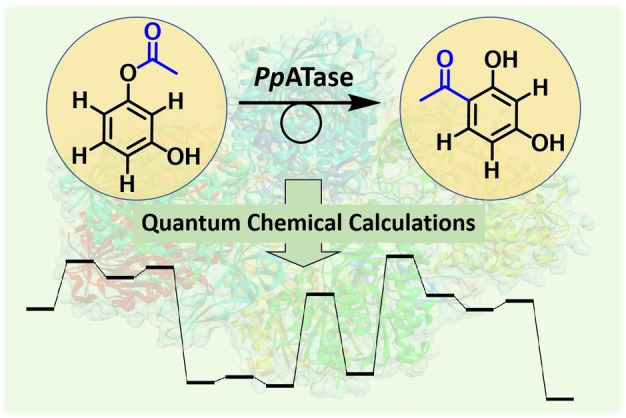

## 1 Introduction

**Scheme 1:**
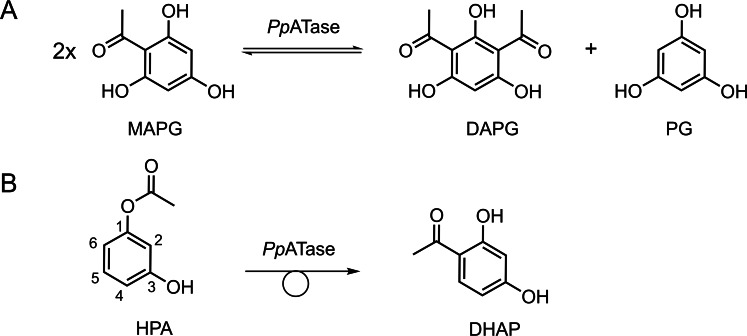
(A) The natural reaction and (B) promiscuous Fries rearrangement catalyzed by *Pp*ATase.

The acyltransferase from *Pseudomonas protegens* (*Pp*ATase) catalyzes in nature the cofactor‐independent reversible interconversion of monoacetylphloroglucinol (MAPG) to diacetylphloroglucinol (DAPG) and phloroglucinol (PG) (Scheme 1A).[^1^, ^2^, ^3^, ^4^] *Pp*ATase is of interest in biocatalysis due to its ability to produce acylated phenolic compounds. For example, the enzyme was shown to be capable of catalyzing the regioselective acetylation of resorcinol derivatives using a broad range of acyl donors, such as phenyl esters, acetate derivatives and thioesters.[^5^, ^6^, ^7^] The scope of acyl donors was further extended to sterically demanding compounds by a single‐point mutation.^[8]^ *Pp*ATase was also found able of catalyzing the amide formation using aniline derivatives as acyl acceptors.^[9]^

One interesting reaction of *Pp*ATase is the transformation of 3‐hydroxyphenyl acetate (HPA) to the corresponding mono‐C‐acetylated resorcinol derivative 2′,4′‐dihydroxyacetophenone (DHAP) shown in Scheme 1B.^[5]^ This reaction represents a Fries‐like rearrangement, giving access to hydroxy aryl ketones, which have wide applications, for example in the pharmaceutical and fragrance industries. The chemical Fries rearrangement is generally catalyzed by Lewis or Brønsted acids.[^10^, ^11^, ^12^]

In a previous study, we used quantum chemical calculations to investigate the detailed mechanism of the natural reaction of *Pp*ATase.^[13]^ A large cluster model of the active site, consisting of over 400 atoms, was designed on the basis of the crystal structure,^[14]^ and the intermediates and transition states (TSs) along the reaction pathway were located.

The *Pp*ATase reaction mechanism involves two half‐reactions, namely the acylation of the enzyme by the first MAPG substrate and the subsequent acyl transfer from the acylated enzyme to the second MAPG substrate. The calculations showed that the mechanism of the second half‐reaction follows essentially the reverse of the acylation in the first half‐reaction, with only some small differences in the energies.^[13]^ The calculations could confirm the role of the Cys88 residue as the nucleophile in the reaction, attacking the acyl moiety. Very importantly, the calculations could identify the Asp137 residue, which is located more than 10 Å from the substrate, as the source of the proton in the rate‐determining step. This prediction was corroborated by site‐directed mutagenesis experiments, which showed that the D137 N mutant is inactive.^[13]^ The calculations were also helpful in rationalizing the particular regioselectivity of the natural reaction.

In the present work, we use the same quantum chemical cluster methodology as in the previous study to investigate the Fries rearrangement of HPA to DHAP catalyzed by *Pp*ATase. This technique has over the years proven to be very valuable in modeling enzymatic reaction mechanisms.^[15]^ In particular, it has been used for understanding mechanisms and origins of selectivity of enzymes of biocatalytic interest.^[16]^ On the basis of the calculations, a detailed reaction mechanism with feasible energy barriers is proposed for the Fries rearrangement, and the key residues involved in the reaction are identified. The relative binding energies of various acetyl acceptors are calculated to evaluate the possibility of an intermolecular Fries rearrangement by *Pp*ATase.

## Computational Details

All calculations were preformed using the Gaussian 09 program^[17]^ with the B3LYP−D3(BJ) hybrid density functional method.[^18^, ^19^] Geometry optimizations were carried out with the 6‐31G(d,p) basis set. On the basis of the optimized structures, single‐point energies were calculated at the same level of theory using the SMD solvation model^[20]^ with the dielectric constant ϵ=4 to estimate the effect of the surrounding protein environment. Frequency calculations were performed at the same level of geometry optimization to obtain zero‐point energies (ZPE). To get more accurate electronic energies, single‐point calculations on the optimized structures were performed with the larger basis set 6‐311+G(2d,2p). The energy values reported in the present paper are thus the large basis set energies corrected for solvation and ZPE effects. The B3LYP density functional in conjunction with the Grimme's dispersion correction and the basis sets chosen for the present work have been extensively employed in previous studies of enzymatic reactions, and have been proven to provide reliable results.[^15^, ^16^] The nature of the optimized transition states was confirmed by the presence of an imaginary frequency representing the motion along the reaction coordinate connecting the preceding and following intermediates. Note that, since a number of atoms are kept fixed in the geometry optimizations (see below), the procedure also results in small imaginary frequencies related to the fixed atoms, which is a common feature of the cluster approach.

## Results and Discussion

### Active Site Model

**Figure 1:**
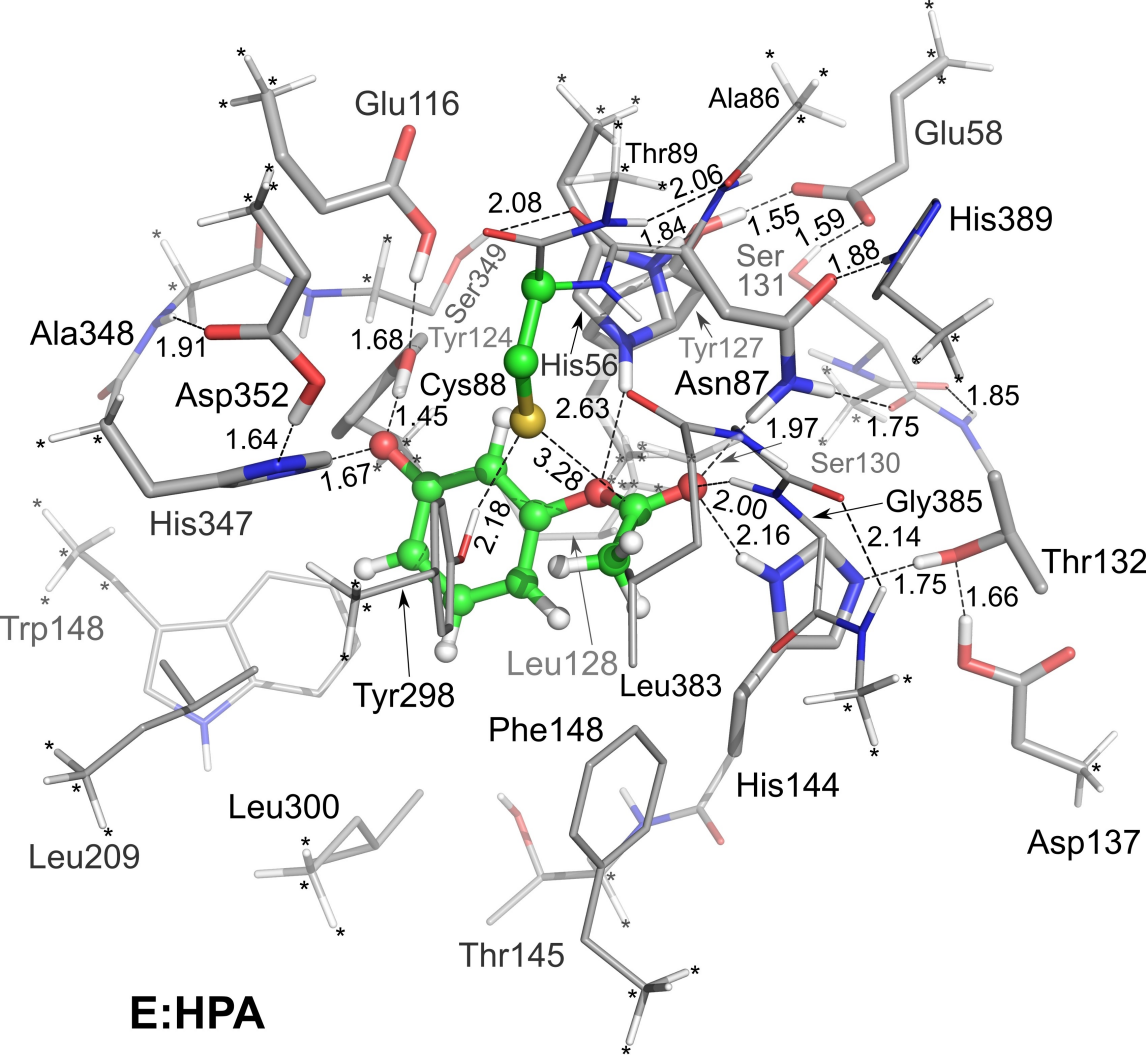
Optimized structure of the lowest‐energy enzyme‐substrate complex of the active site model of *Pp*ATase. The HPA substrate and the nucleophilic Cys88 are highlighted. Atoms fixed during the geometry optimization are marked with asterisks, and selected distances are given in angstrom.

We employ here the same active site model as in the previous study on the natural reaction of *Pp*ATase,^[13]^ which is based on the crystal structure (PDB 5MG5^[14]^). The model consists of the following groups, as shown in Figure 1: the HPA substrate, His56, Glu58, Ala86, Asn87, Cys88, Thr89, Glu116, Tyr124, Tyr127, Ile128, Ser130, Ser131, Thr132, Asp137, His144, Thr145, Phe148, Leu209, Trp211, Tyr298, Leu300, His347, Ala348, Ser349, Asp352, Leu383, Gly384, Gly385, Tyr386 and His389. The final active site model has 412 atoms and an overall charge of −2. As usual in the cluster approach, a number of atoms at the edge of the model were kept fixed during the geometry optimizations to maintain the overall structure of the active site close to the X‐ray and to prevent excessive movements. These positions are indicated by asterisks in the figures.

The choices for the protonation states for the various active site residues follow also the previous study on the natural reaction.^[13]^ Cys88 is in the ionized form, and the nearby Asp352 is in the neutral form. His347 is modeled as neutral due to the hydrogen bonding interaction with Asp352. Asp137 and His144, which are bridged by Thr132, are in the neutral forms. For the His56 and Glu58 residues, which are bridged by Tyr127, the previous study showed that a spontaneous proton transfer took place from Glu58 to His56 during the geometry optimization when starting with the initial model with the two residues in neutral forms.^[13]^ Therefore, Glu58 is here modeled in the ionized form and His56 is modeled in the protonated state. Finally, the Glu116 residue is modeled in the deprotonated form. However, during the geometry optimization of the lowest‐energy enzyme‐substrate complex (see below), a proton moved spontaneously from the hydroxyl group of the substrate to Glu116.

### Reaction Mechanism

First, the geometries of a number of enzyme‐substrate complexes were optimized, with the HPA substrate in different orientations in the active site model. The structure of the lowest energy (called **E:HPA**) is given in Figure 1 (see Supporting Information for other structures). In **E:HPA**, the substrate is oriented such that the acetyl group is anchored by three hydrogen bonds with the side chains of Asn87 and His144, and the backbone amide group of Gly385. During the geometry optimization of **E:HPA**, the hydroxyl group of HPA loses a proton to Glu116 via Tyr124 and forms a hydrogen bond with the latter. It also forms a hydrogen bond with the NH group of His347.

**Scheme 2:**
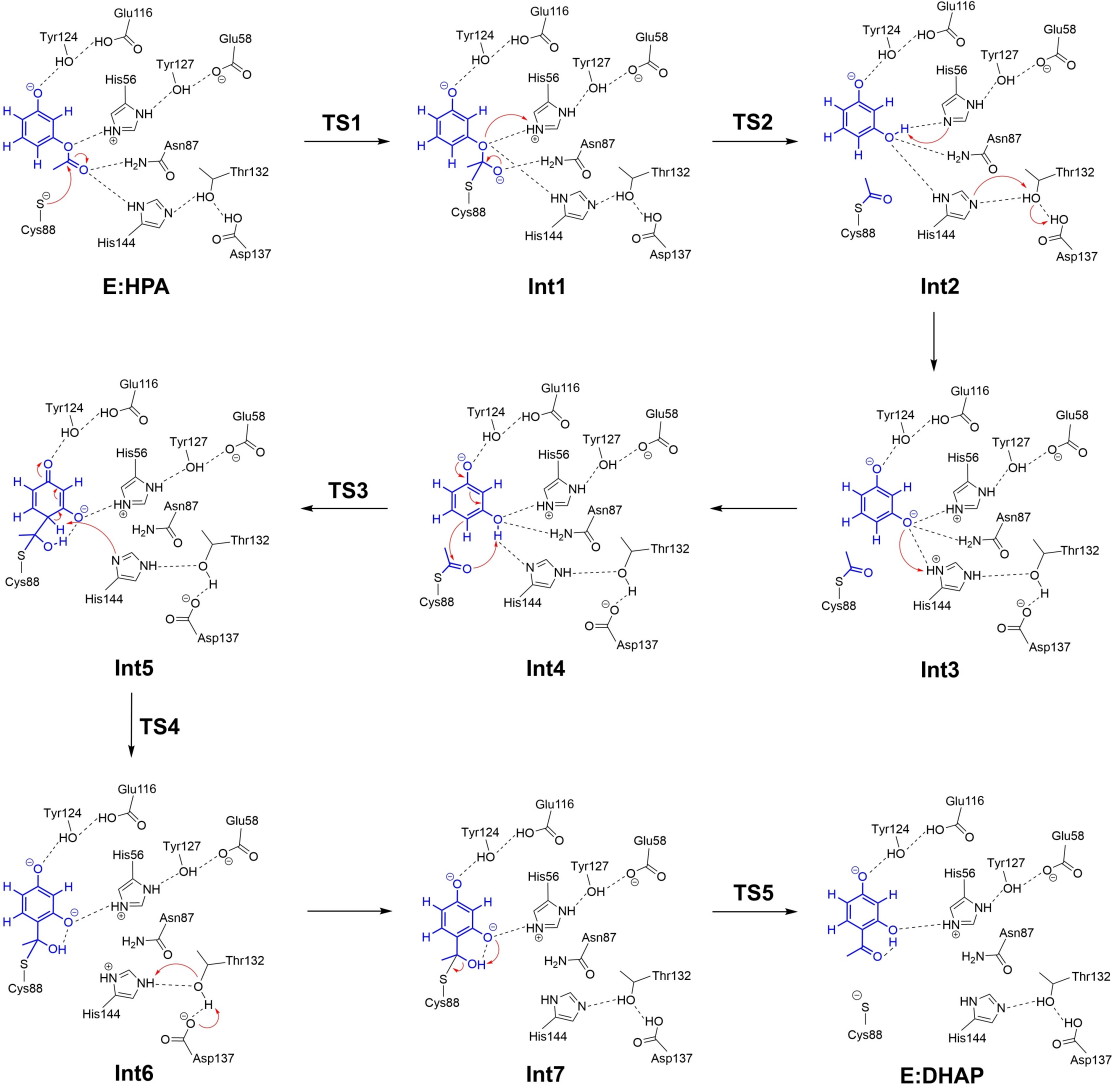
Reaction mechanism of *Pp*ATase‐catalyzed conversion of HPA to DHAP proposed on the basis of the current calculations.

**Figure 2:**
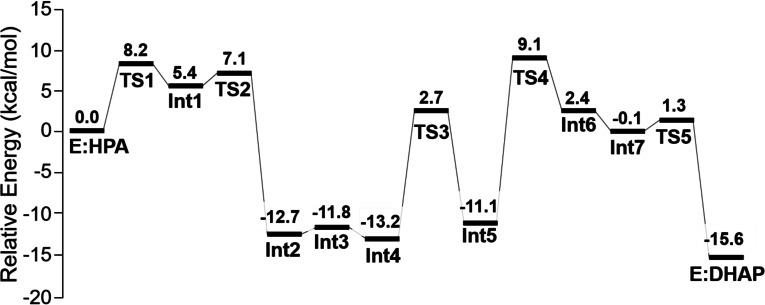
Calculated energy profile for the proposed mechanism of *Pp*ATase‐catalyzed conversion of HPA to DHAP.

**Figure 3:**
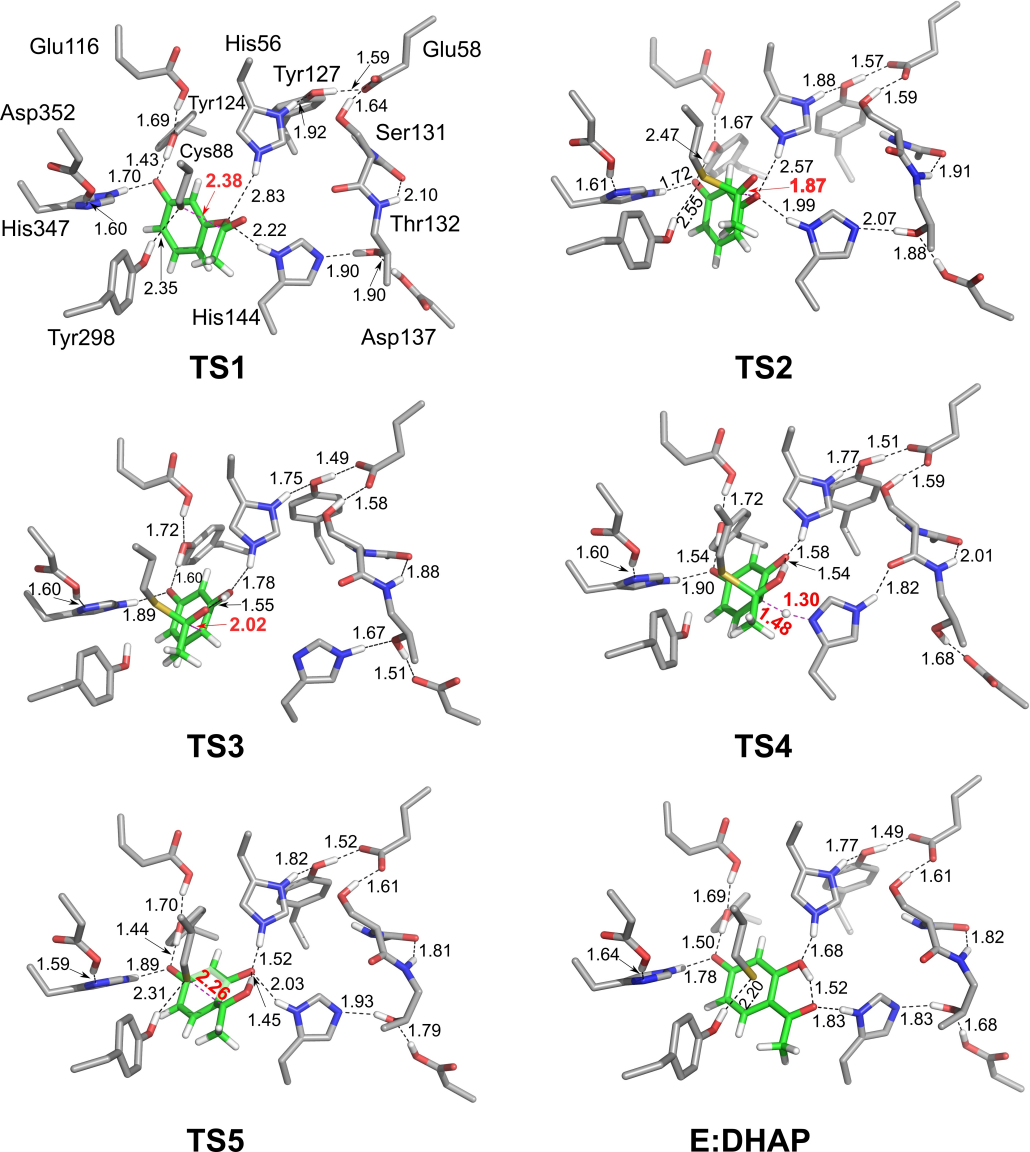
Optimized structures of the transition states and the enzyme‐product complex (**E:DHAP**) in the suggested mechanism for the *Pp*ATase‐catalyzed conversion of HPA to DHAP. Note that for clarity only a part of the active site model is shown here. In addition, most of nonpolar hydrogens have been omitted. For full models see Supporting Information. Distances are given in angstrom.

Starting from **E:HPA** we have optimized the transition states and intermediates for the entire reaction pathway. The obtained mechanism is shown in Scheme 2, the calculated energy profile is given in Figure 2, and the optimized geometries of the transition states and intermediates are displayed in Figure 3 and in SI.

The *Pp*ATase‐catalyzed Fries rearrangement starts with the formation of a covalent bond between the enzyme and HPA by a nucleophilic attack of Cys88 at the carbonyl carbon of the substrate. The barrier for this step is calculated to be 8.2 kcal/mol, and the formed alkoxide intermediate (**Int1**) is 5.4 kcal/mol higher than **E:HPA**. The alkoxide is stabilized by hydrogen bonds to the side chain of Asn87 and the backbone amide groups of Cys88 and Gly385.

Next, the reaction continues by a C−O bond cleavage, which was found to occur concertedly with a proton transfer from His56 to the ester oxygen, resulting in the formation of resorcinol and an acetylated Cys88 (**Int2**, Scheme 2). The barrier for this step is very low, only 1.7 kcal/mol higher than **Int1**, and the step is exothermic by 18.1 kcal/mol, i. e. **Int2** is 12.7 kcal/mol lower than **E:HPA** (Figure 2). Next follows two almost thermoneutral proton transfer events (Scheme 2). The first one, **Int2**→**Int3**, involves proton transfers from the resorcinol to His56 and from Asp137 to His144 via Thr132. The second one, **Int3**→**Int4**, involves proton transfer from His144 to the resorcinol.

Continuing from **Int4**, the reaction proceeds by a C−C bond formation between the acylated cysteine and the C6 carbon of the substrate. According to the calculations, the C−C bond formation takes place concertedly with a proton transfer from C1−OH to the carbonyl oxygen of the acyl group, leading to the formation of a dienone intermediate (**Int5**). The barrier for this step is calculated to be 15.9 kcal/mol relative to **Int4**, and it is interesting to note that the step is endothermic by only 2.1 kcal/mol, despite the fact that the aromaticity is broken. As discussed in the previous study on the natural reaction, the conjugations in the dienone intermediate make it rather stable.^[13]^

From **Int5**, re‐aromatization takes place via **TS4**, whereby the His144 residue abstracts a proton from the C6 carbon of the substrate resulting in **Int6**. The calculations show that this is the rate‐determining step of the reaction, with an overall barrier of 22.3 kcal/mol relative to **Int4** (Figure 2). Somewhat surprisingly, the re‐aromatization step (**Int5**→**Int6**) is quite endothermic, by 13.5 kcal/mol. The stability of the dienone intermediate contributes to this, as well as the nature of the proton acceptor, in this case a neutral histidine residue.

Next, a proton transfer takes place from His144 to the distant Asp137, shuttled via Thr132, to yield **Int7**, and in the final step, C−S bond cleavage occurs readily via **TS5**. The barrier for this step is 1.4 kcal/mol relative to **Int7**, and the obtained enzyme‐product complex **E:DHAP** is calculated to be 15.6 kcal/mol lower in energy than the enzyme‐substrate complex **E:HPA**.

The calculations show thus that the acylation of the enzyme is highly exothermic process and has a rather low barrier of ca 8 kcal/mol, while the acetyl transfer back to the substrate is only slightly exothermic with a barrier of ca 22 kcal/mol. Similarly to the natural reaction investigated in the previous study, the proton transfer from the C6−H of the substrate to His144 (**TS4**) is the rate‐limiting step. Furthermore, the distant Asp137 residue is also here involved in the reaction, first as a general acid to protonate the substrate via Thr132 and His144, and then as a general base to abstract the proton back via the same groups (Scheme 2).

Here, it is interesting to compare the position of the substrate in the active site throughout the reaction pathway. Superposition of the structures of the various intermediates and TSs shows that the substrate does not move much, and the side chains of the active site residues need only to move a little to accommodate the movement of the substrate (see Supporting Information).

### Exchange of Acetyl Acceptor

Experimental results showed that the acetyl group was not necessarily transferred to the same molecule that donated the acetyl group to the enzyme. Performing the *Pp*ATase reaction of HPA in the presence of 4‐hexylresorcinol led to the formation of both intramolecular product (DHAP) and the intermolecular crossover product, with the latter being the major product.^[5]^ This shows that a ligand exchange must take place between two acetyl acceptors (resorcinol and 4‐hexylresorcinol) after the acylation of the enzyme.

Here, we have estimated the relative binding energy of the two acetyl acceptors using the active site cluster model by optimizing the structure of **Int4** with 4‐hexylresorcinol bound instead of resorcinol (see Supporting Information for the structure). 4‐hexylresorcinol was modeled in the deprotonated form as the resorcinol in **Int4** is in that state.

We used the following equation to evaluate the relative binding energy: 

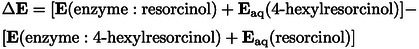




where **E**_aq_ refers to the energy of the acetyl acceptor in aqueous solution, here modeled as an implicit solvent.

Using this approach, it was found that 4‐hexylresorcinol binds better than resorcinol by 3.1 kcal/mol, which is consistent with the experimental finding of the crossover product.^[5]^

For comparison, we also evaluated the binding energies of two other relevant acetyl acceptors, namely MAPG and PG. The **ΔE** for these compounds was calculated to be −1.0 and +1.5 kcal/mol, respectively, relative to resorcinol. These small differences indicate that also MAPG and PG indeed can replace resorcinol and function as acceptors of the acetyl group. The reactions of these acceptors are in fact identical to the natural reaction, the energy profiles for which were considered our previous work.^[13]^

Here, it is worth mentioning that there are more advanced ways for calculating the relative binding free energies, such as the free energy perturbation (FEP) method used recently for the evaluation of the energetics of the ligand exchange step in *Mycobacterium smegmatis* acyl transferase.^[21]^ The current approach should, however, yield reasonable results owing to the structural similarities of the considered compounds.

## Conclusions

We have in the current study used the quantum chemical cluster approach to investigate a promiscuous catalytic activity of acyltransferase from *Pseudomonas protegens* (*Pp*ATase), namely the Fries rearrangement of 3‐hydroxyphenyl acetate (HPA) to 2′,4′‐dihydroxyacetophenone (DHAP).

A detailed mechanism is proposed (Scheme 2), in which the *Pp*ATase‐catalyzed reaction starts with the nucleophilic attack of Cys88 at the carbonyl carbon to form a covalent bond between the enzyme and the substrate. Next, the C−O bond breaks concertedly with the proton transfer from His56 to the ester oxygen, leading to the generation of an acylated enzyme in complex with resorcinol. Then, a couple of proton transfer events take place between the substrate and the active site residues, arriving finally at an intermediate which is the starting point for the C‐acylation process. A C−C bond formation takes then place between the carbonyl carbon of the acylated Cys88 and resorcinol, concertedly with a proton transfer from the *ortho*‐hydroxyl group to the carbonyl oxygen, leading to the formation of a dienone intermediate. The next step is a deprotonation of the C6−H by Asp137, via His144 and Thr132, which is followed by the formation of the DHAP product through a C−S bond cleavage.

The calculations suggest that the acyl transfer from 3‐hydroxyphenyl acetate to the enzyme has a low barrier and is highly exothermic, while the acetyl transfer back to the substrate is rate‐limiting and only slightly exothermic.

We have also considered the binding energies of several acetyl acceptors and the calculations indicate that these compounds can be competitive with the resorcinol intermediate, leading to intermolecular Fries rearrangement.

We believe that the mechanistic insights provided by the current calculations will be helpful in guiding future engineering of *Pp*ATase and related enzymes to obtain variants with improved activity and wider substrate scopes.

## Supporting Information

Optimized structures of enzyme‐substrate complexes with different binding modes; Geometries of optimized intermediates and transition states in the proposed mechanism; Superposition of the optimized structures of intermediates and transition states; Absolute energies and energy corrections; Cartesian coordinates of optimized structures.

## Conflict of interests

The authors declare no conflict of interest.

2

## Supporting information

As a service to our authors and readers, this journal provides supporting information supplied by the authors. Such materials are peer reviewed and may be re‐organized for online delivery, but are not copy‐edited or typeset. Technical support issues arising from supporting information (other than missing files) should be addressed to the authors.

Supporting Information

## Data Availability

The data that support the findings of this study are available from the corresponding author upon reasonable request.
