# Supplementary material for: Computational Study of the Fries Rearrangement Catalyzed by Acyltransferase from Pseudomonas protegens
Source: ChemistryOpen. 2024 Jan 15;13(7):e202300256. doi: 10.1002/open.202300256 (PMC11230933; doi:10.1002/open.202300256)
Supplement: Supplementary file 1 — Supporting Information [file OPEN-13-e202300256-s001.pdf]

ChemistryOpen

Supporting Information

Computational Study of the Fries Rearrangement Catalyzed by Acyltransferase from *Pseudomonas protegens*

Xiang Sheng,* Wolfgang Kroutil, and Fahmi Himo*

Supporting Information

Table of Contents

1. Optimized structures of enzyme-substrate complexes S2

2. Geometries of intermediates and transition states S7

3. Superposition of the optimized structures S14

4. Optimized structure of the enzyme model in complex with 4-hexylresorcinol S15

5. Absolute energies and energy corrections S16

6. Cartesian coordinates S17

1. Optimized structures of enzyme-substrate complexes

Optimized structures of the enzyme-substrate complexes with the HPA substrate in different orientations in the active site model are shown in the figures below (pages S2-S6). Energies relative to the lowest-energy one (**E:HPA**) are given in kcal/mol.

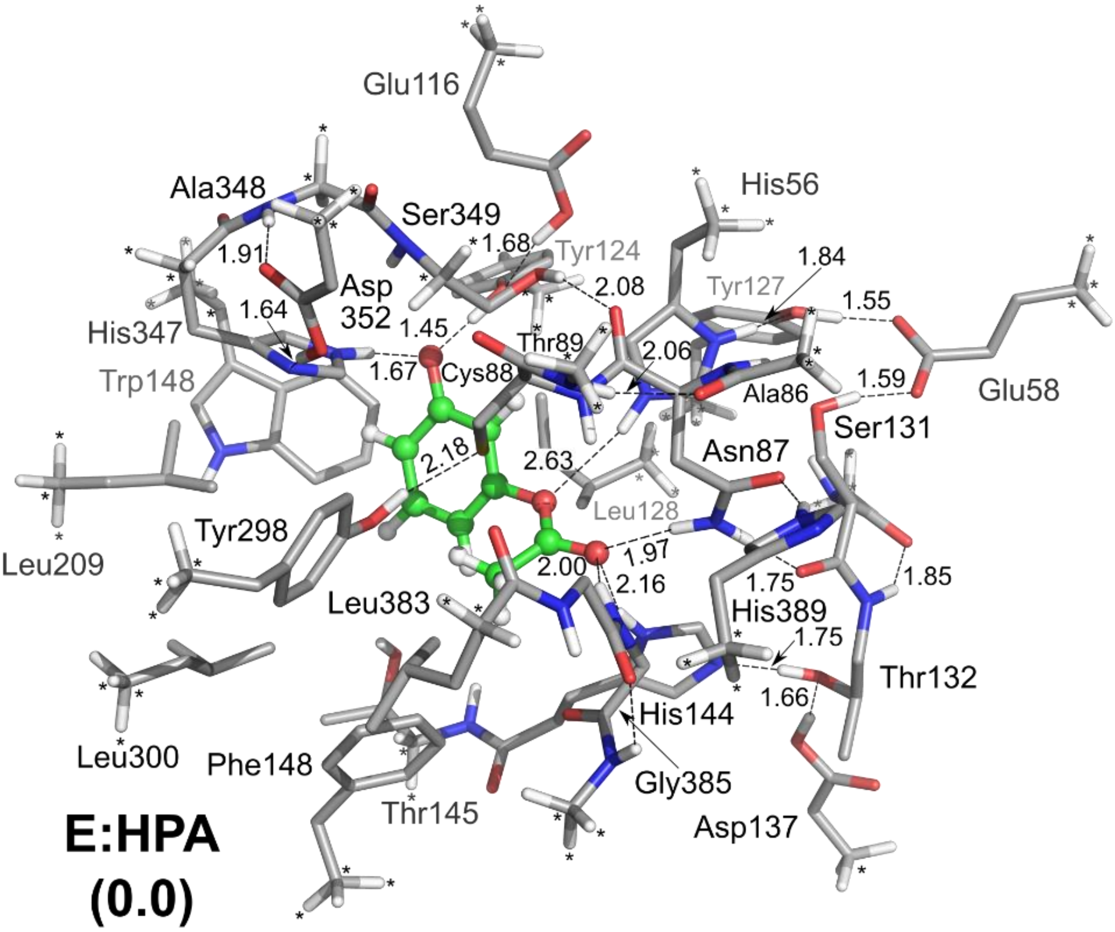

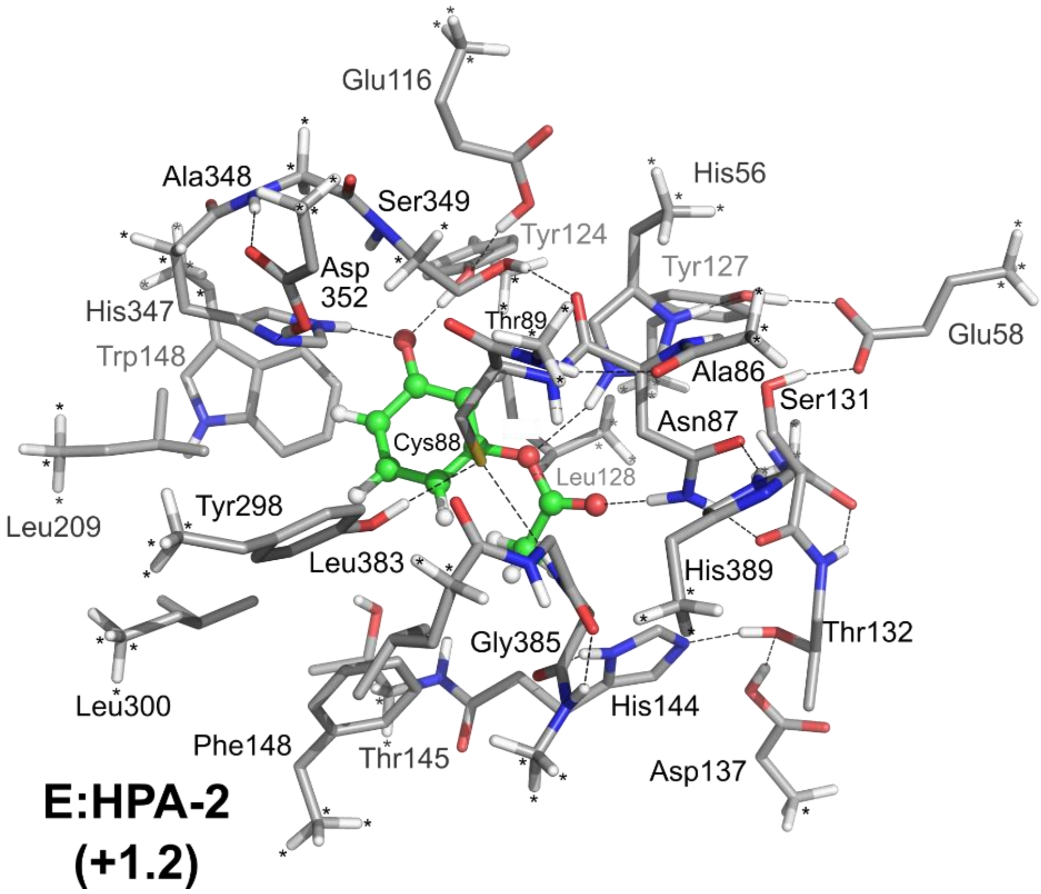

E:HPA-2
(+1.2)

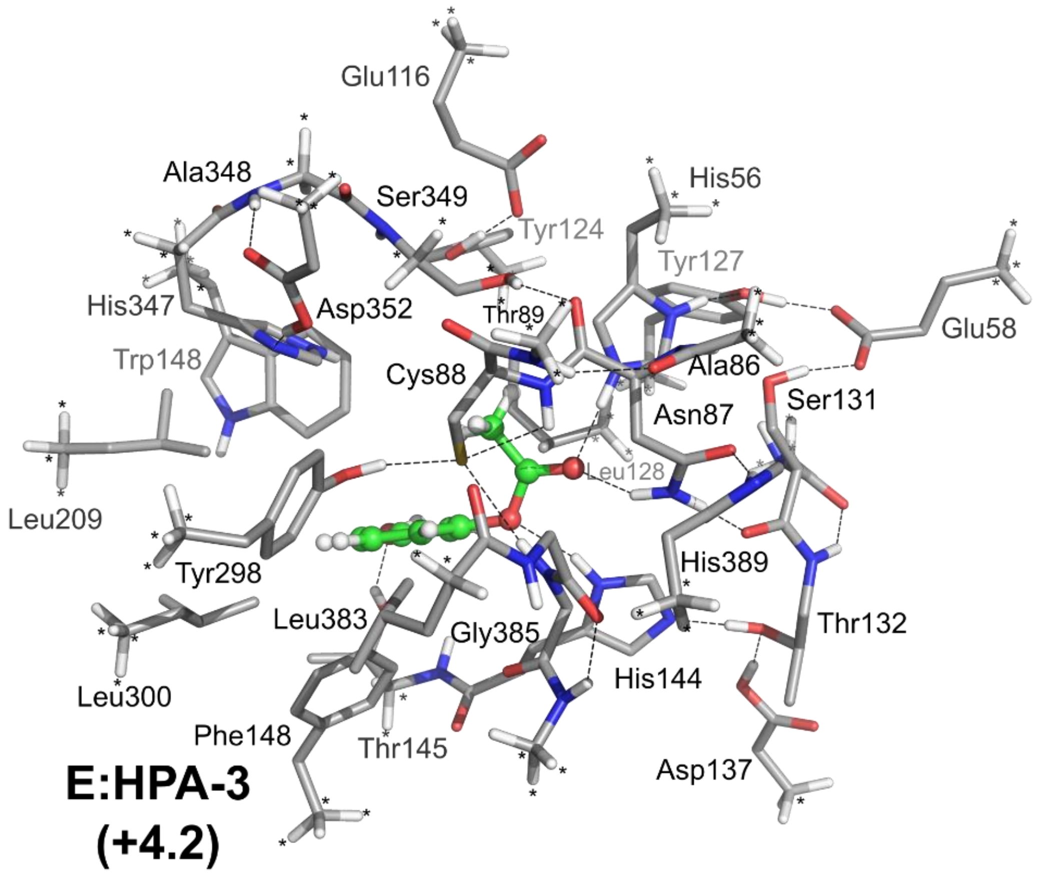

E:HPA-3
(+4.2)

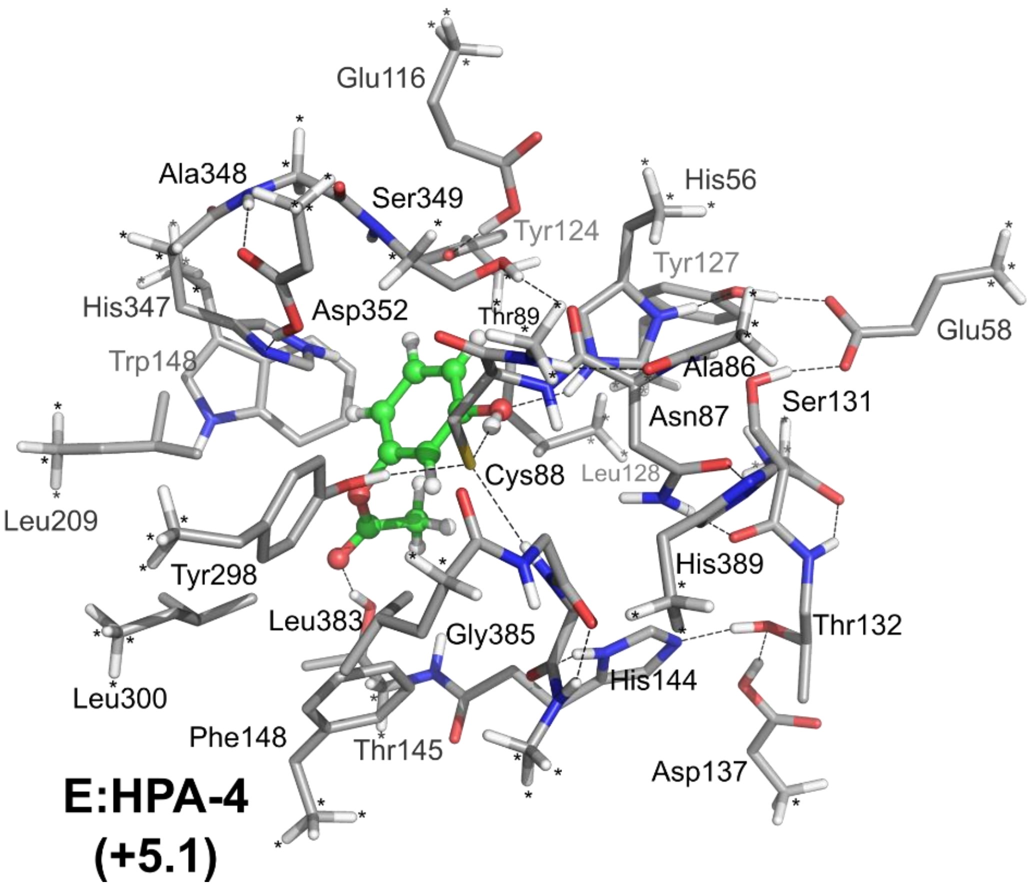
Glu116
Ala348
Ser349
Tyr124
His56
Tyr127
His347
Asp352
Thr89
Glu58
Trp148
Ala86
Asn87
Ser131
Cys88
Leu128
Leu209
Tyr298
His389
Leu383
Gly385
Thr132
Leu300
His144
Phe148
Thr145
Asp137
E:HPA-4
(+5.1)

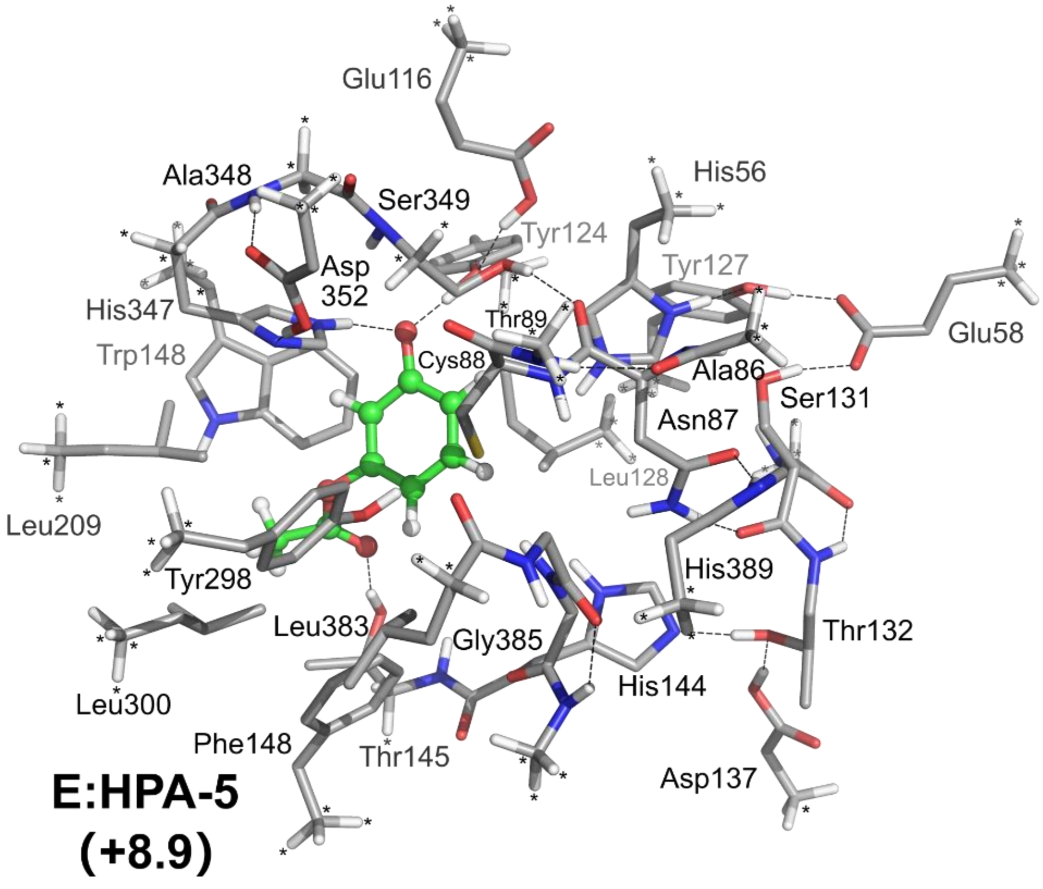
Glu116
Ala348
Ser349
Tyr124
His56
Tyr127
Asp
352
His347
Thr89
Glu58
Trp148
Cys88
Ala86
Ser131
Asn87
Leu128
Leu209
Tyr298
His389
Leu383
Gly385
Thr132
Leu300
His144
Phe148
Thr145
Asp137
E:HPA-5
(+8.9)

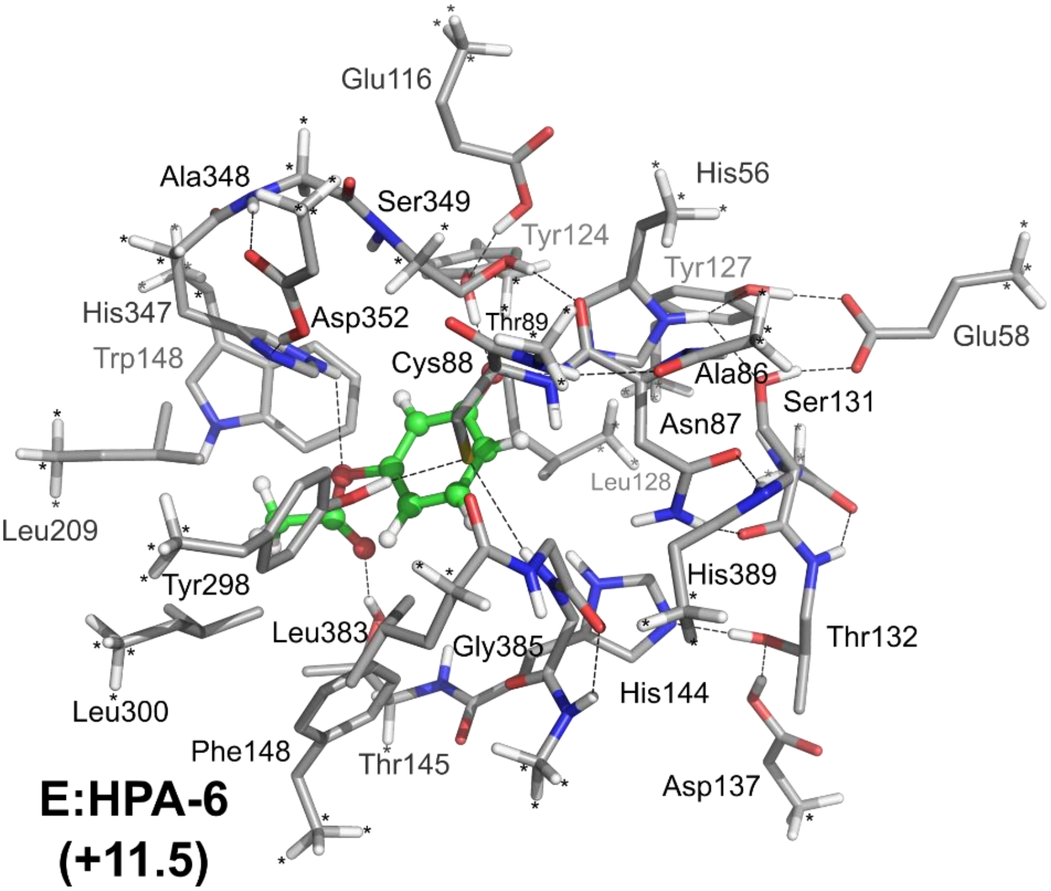

E:HPA-6
(+11.5)

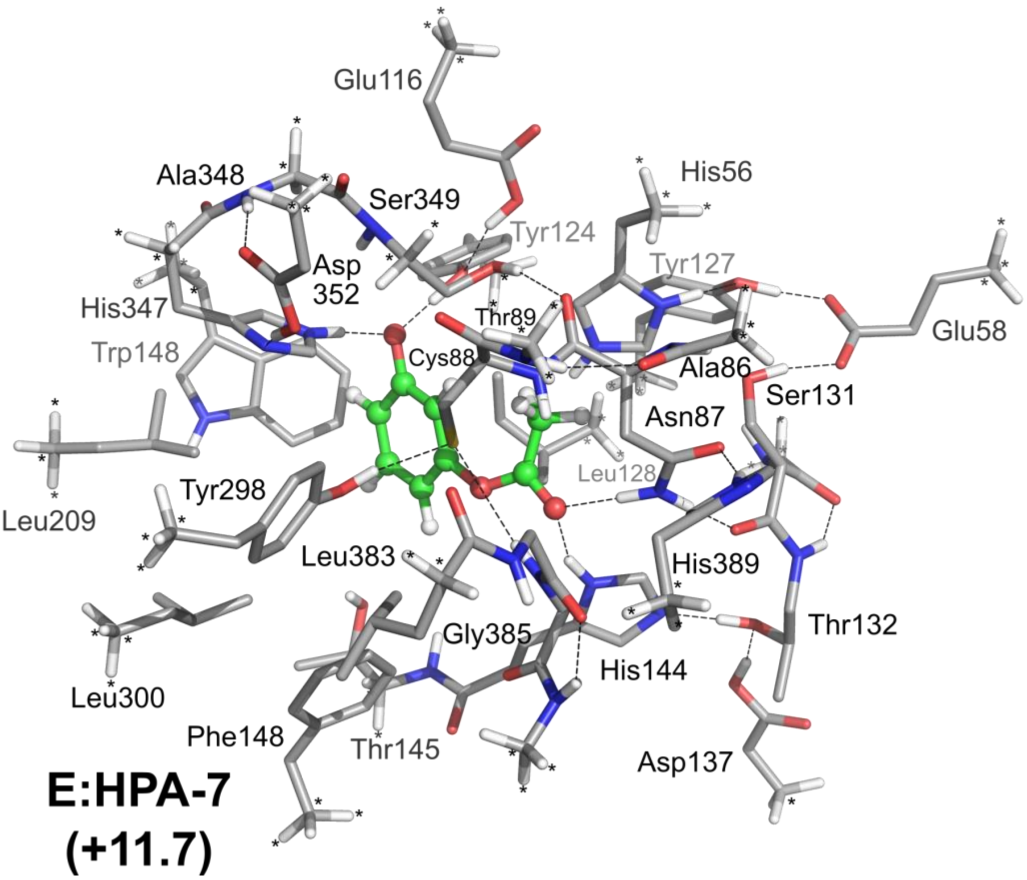

E:HPA-7
(+11.7)

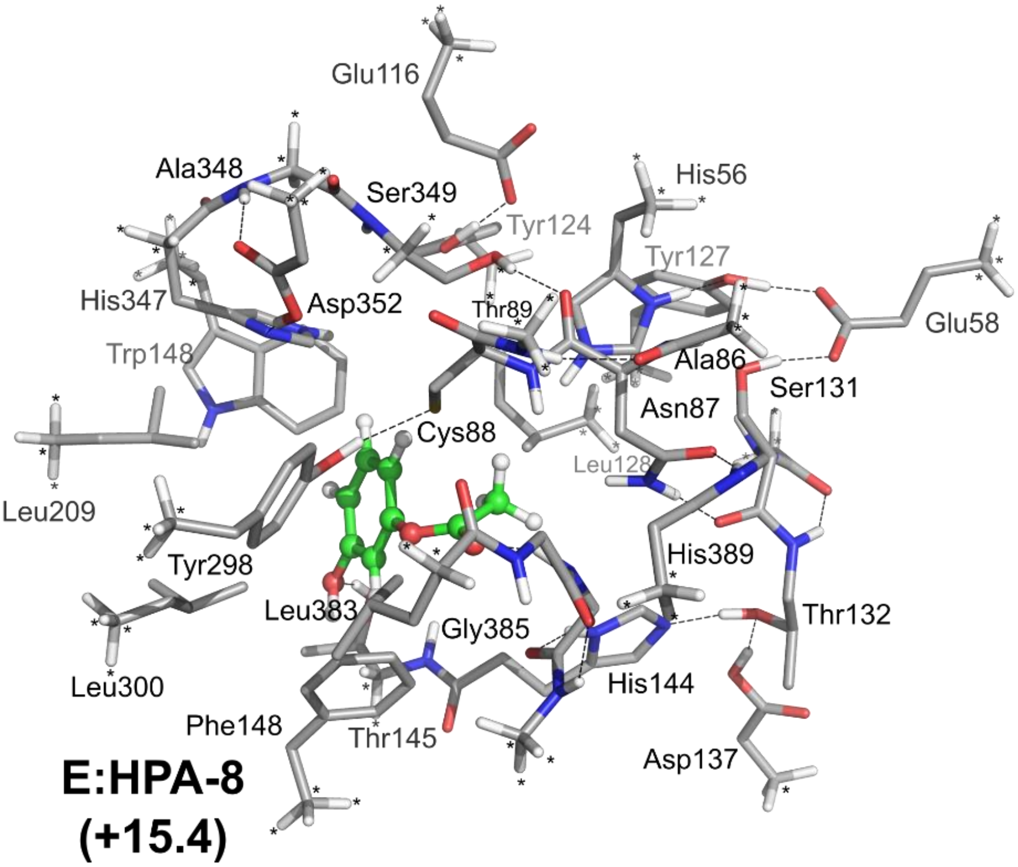
Glu116
Ala348
Ser349
Tyr124
His56
Tyr127
His347
Asp352
Thr89
Glu58
Trp148
Ala86
Ser131
Asn87
Cys88
Leu128
Leu209
Tyr298
His389
Leu383
Thr132
Gly385
Leu300
His144
Phe148
Thr145
Asp137
E:HPA-8
(+15.4)

2. Geometries of intermediates and transition states

Optimized structures of the intermediates and transition states in the lowest-energy pathway are shown in the figures below (pages S7-S13). Energies relative to **E:HPA** are given in kcal/mol.

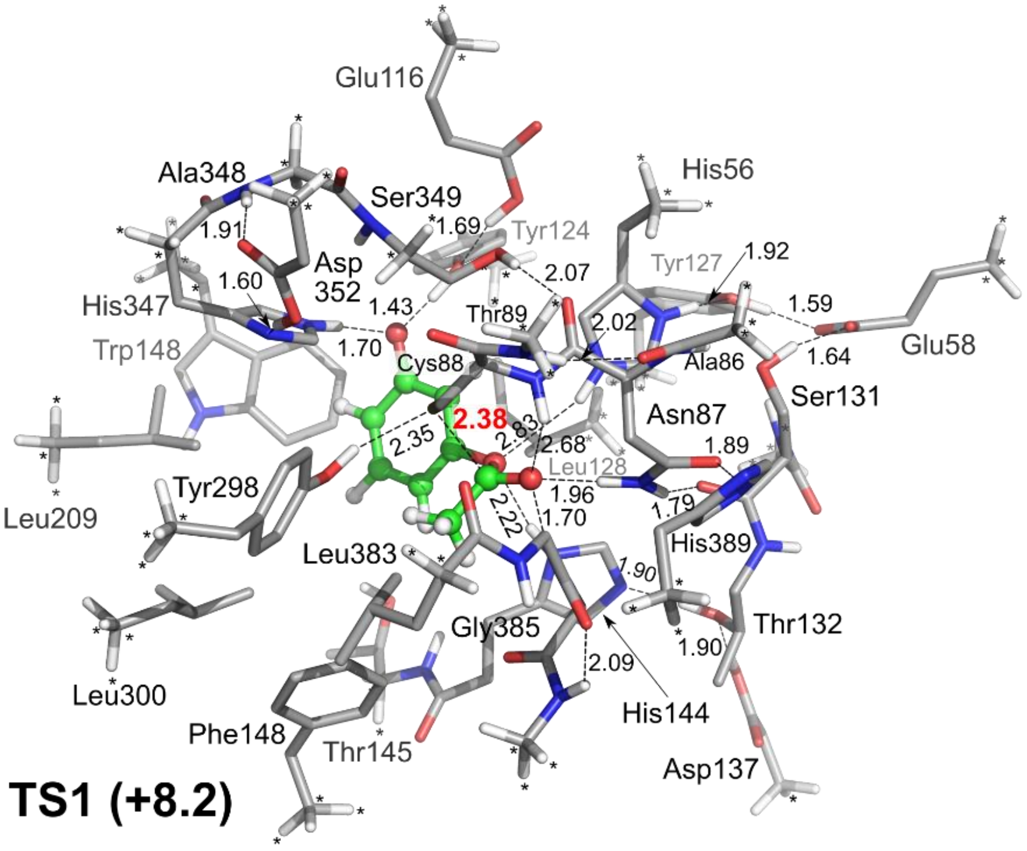

TS1 (+8.2)

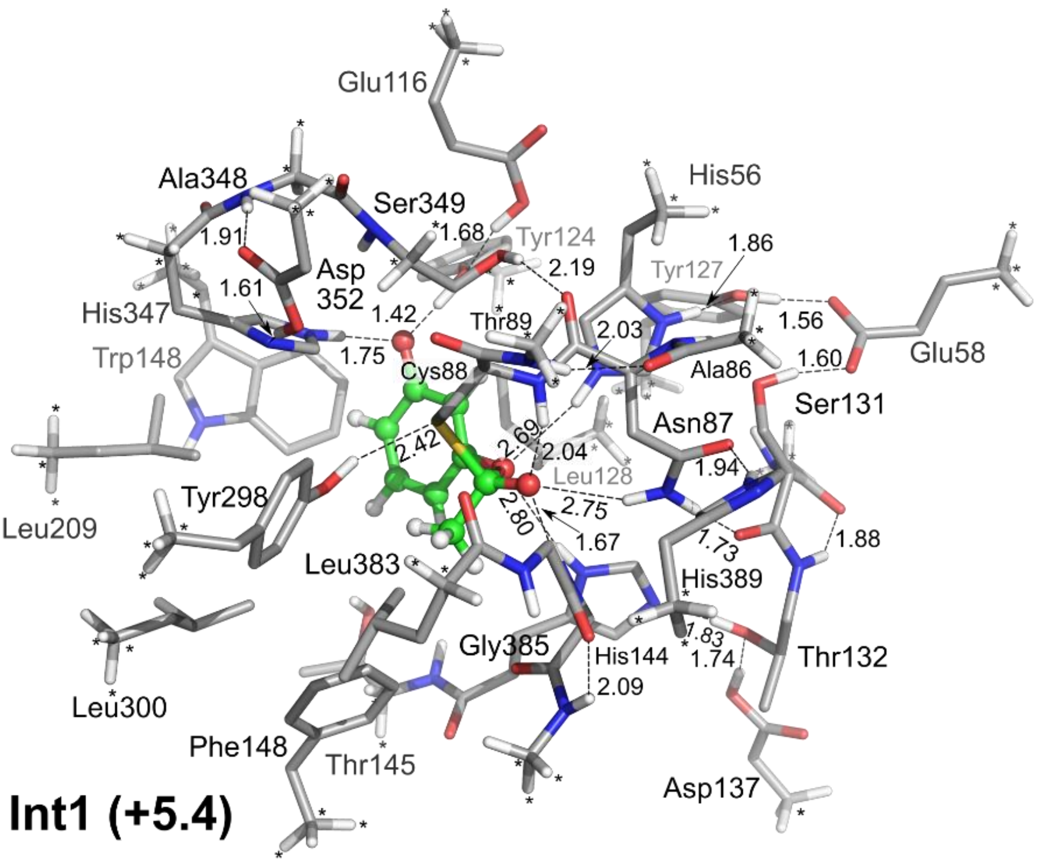

Int1 (+5.4)

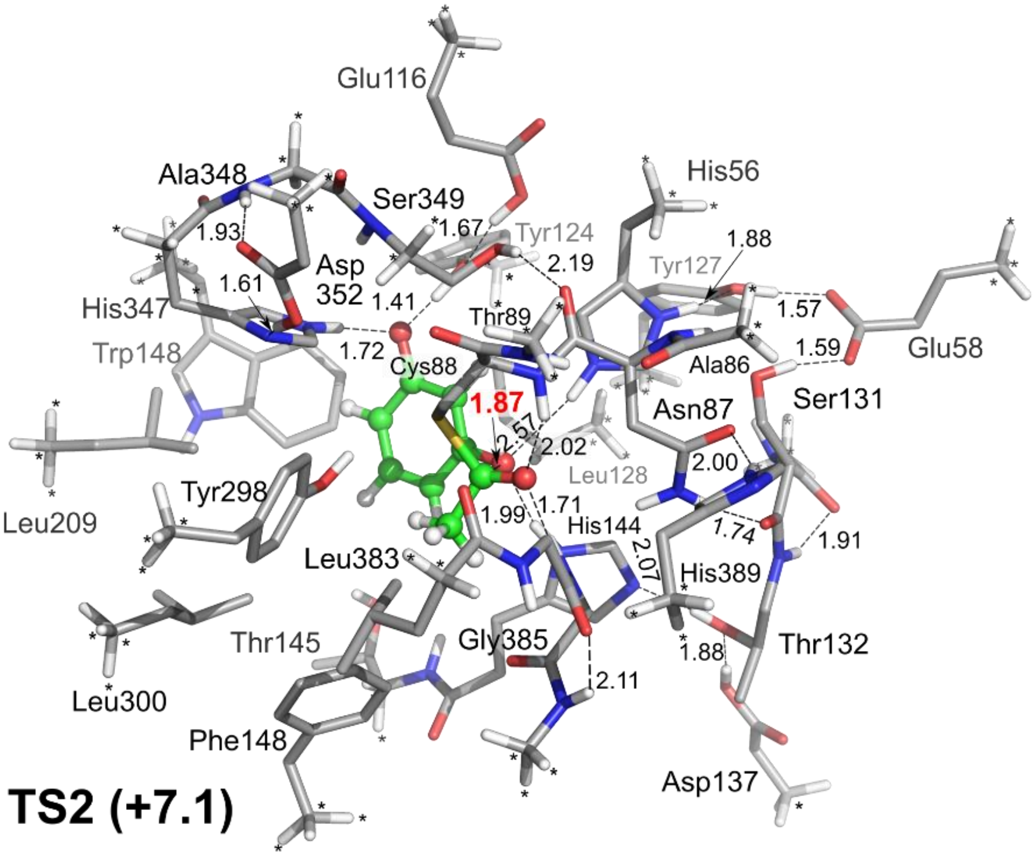

TS2 (+7.1)

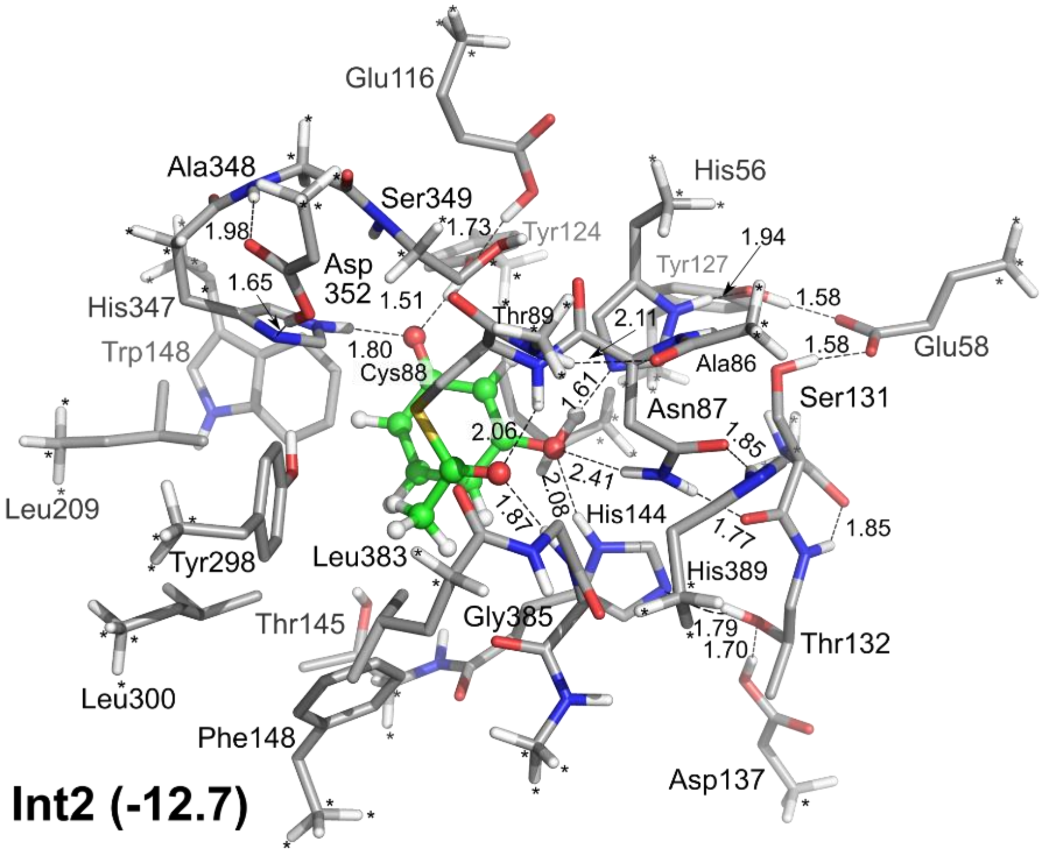

Int2 (-12.7)

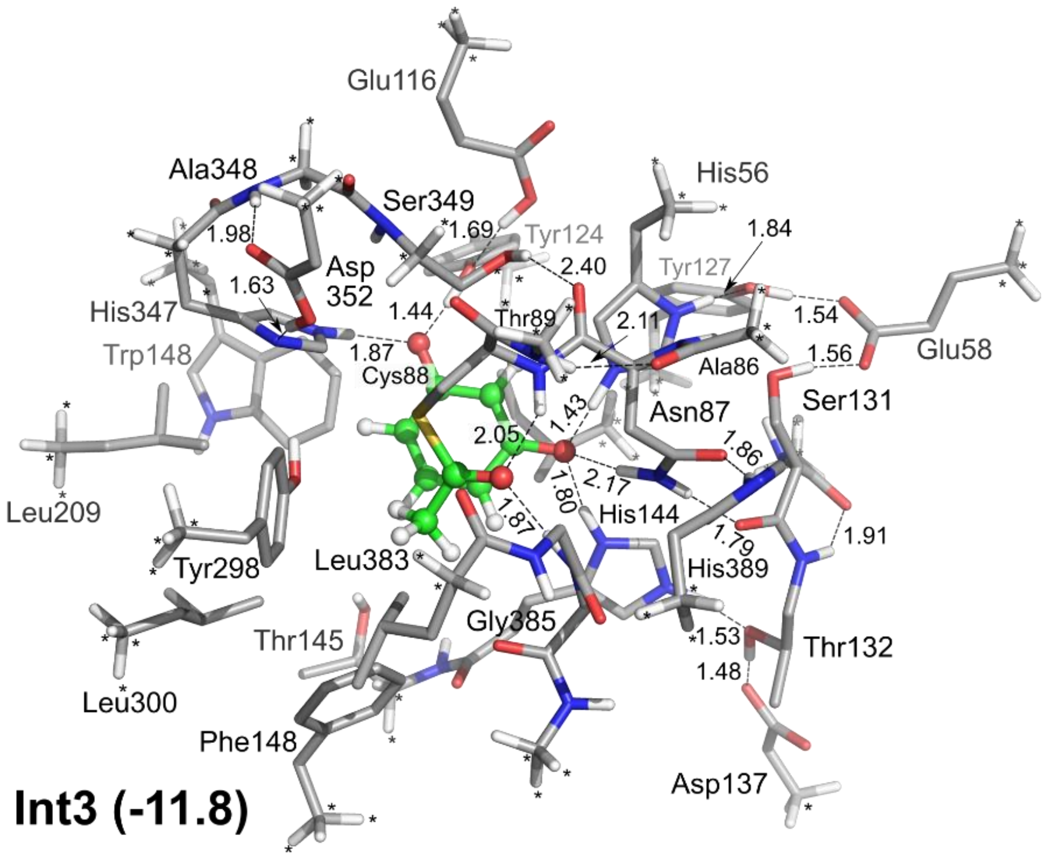

Int3 (-11.8)

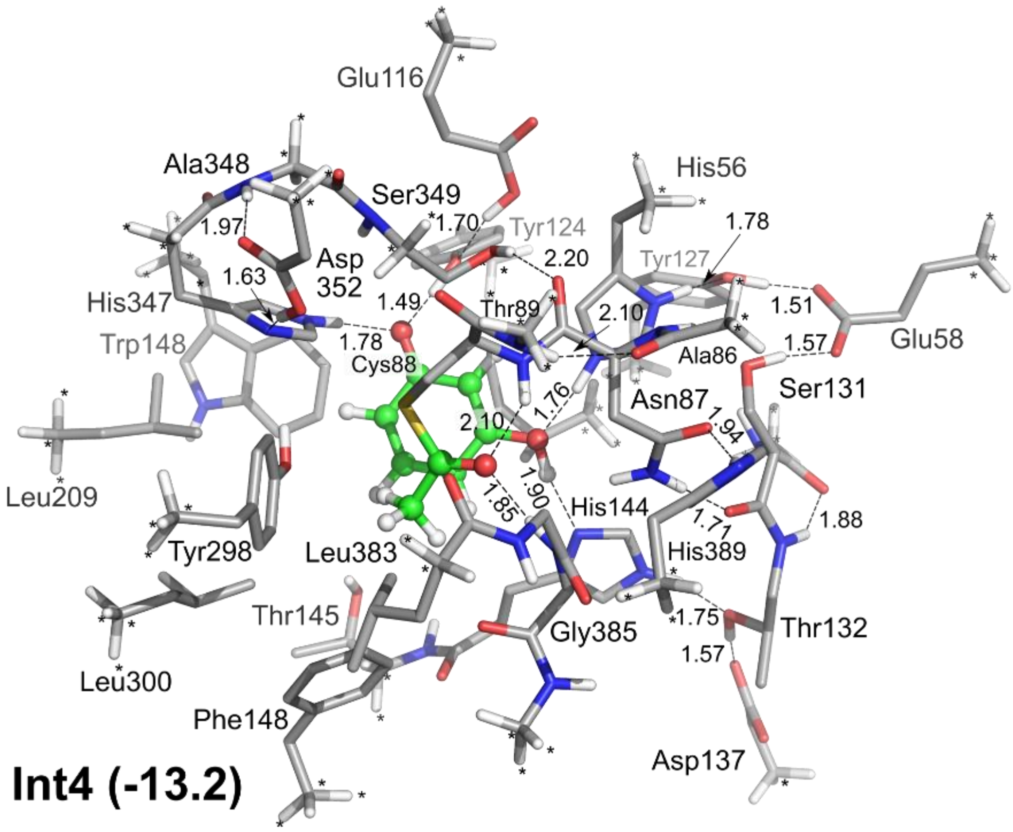

Int4 (-13.2)

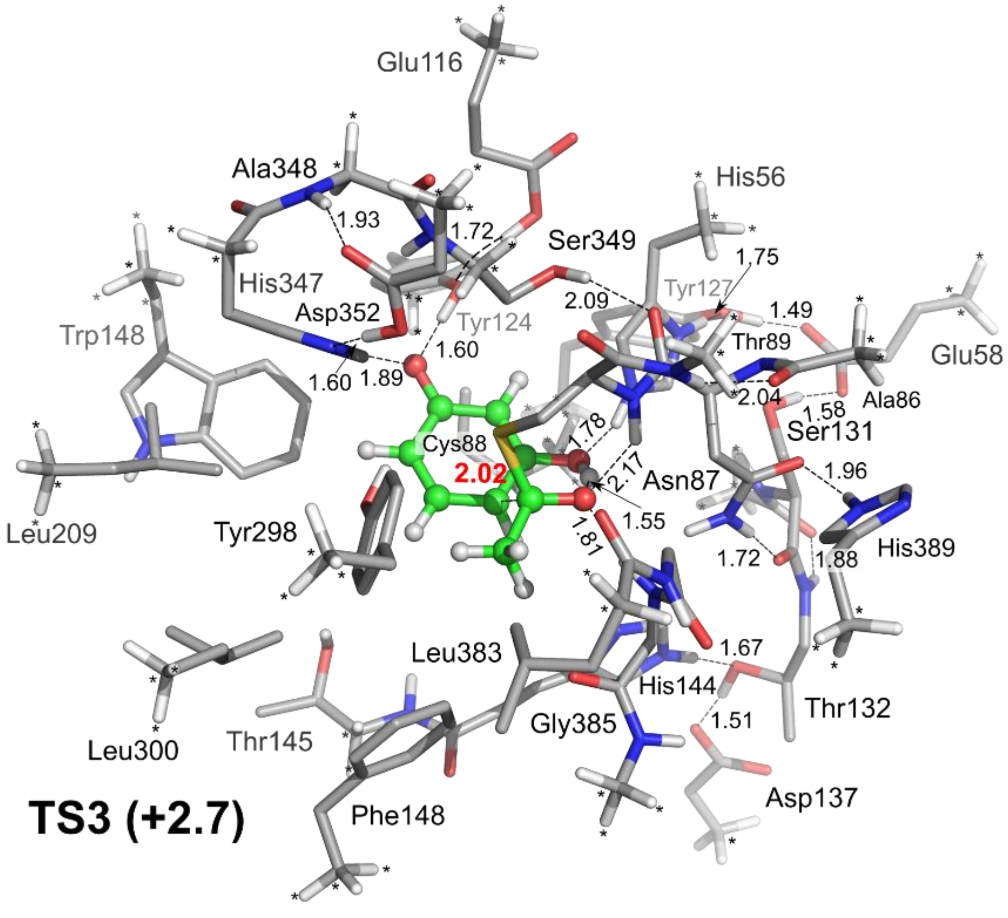

TS3 (+2.7)

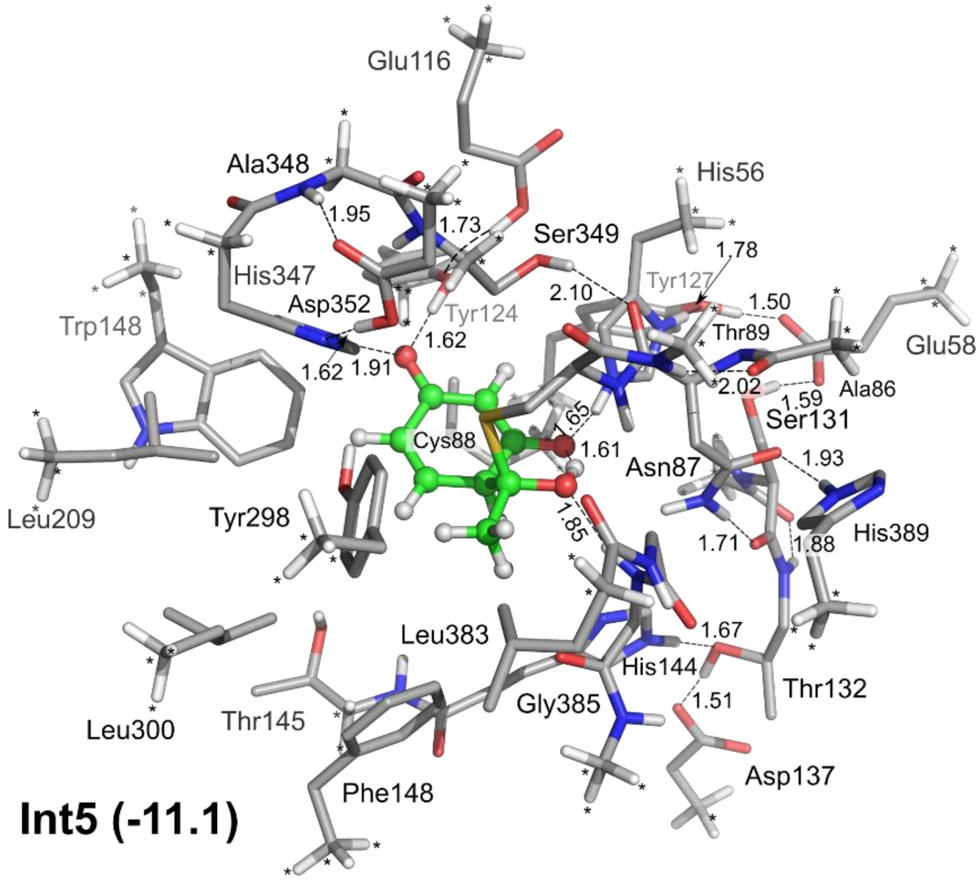

Int5 (-11.1)

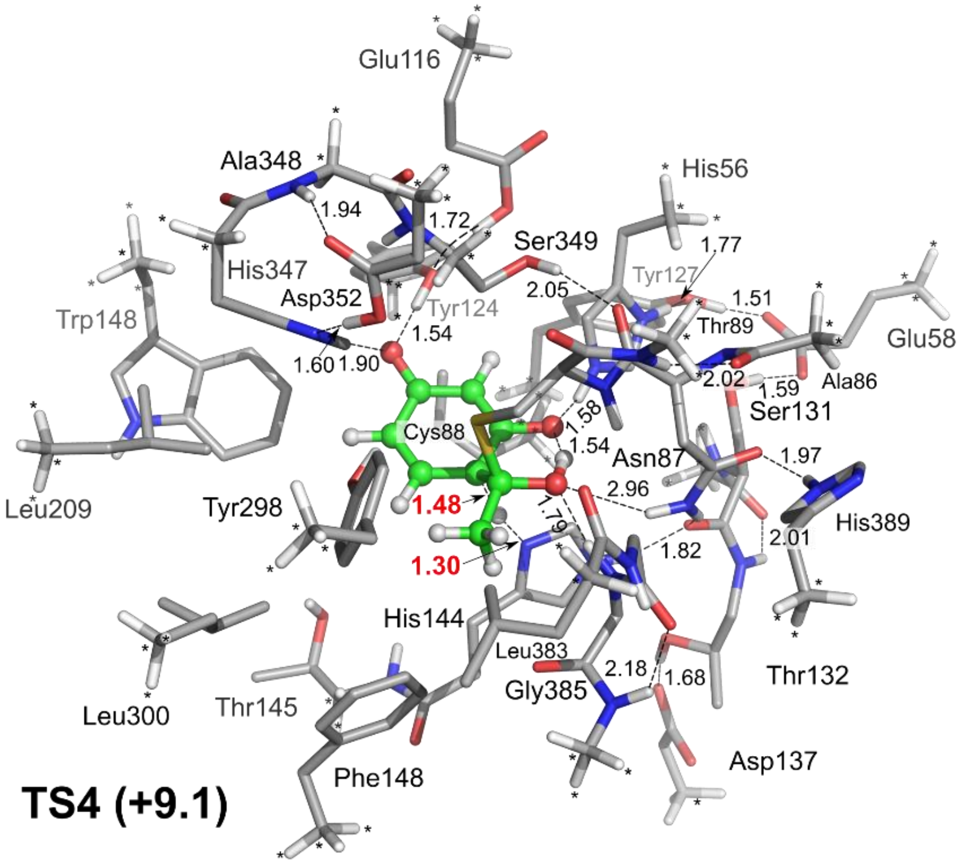

TS4 (+9.1)

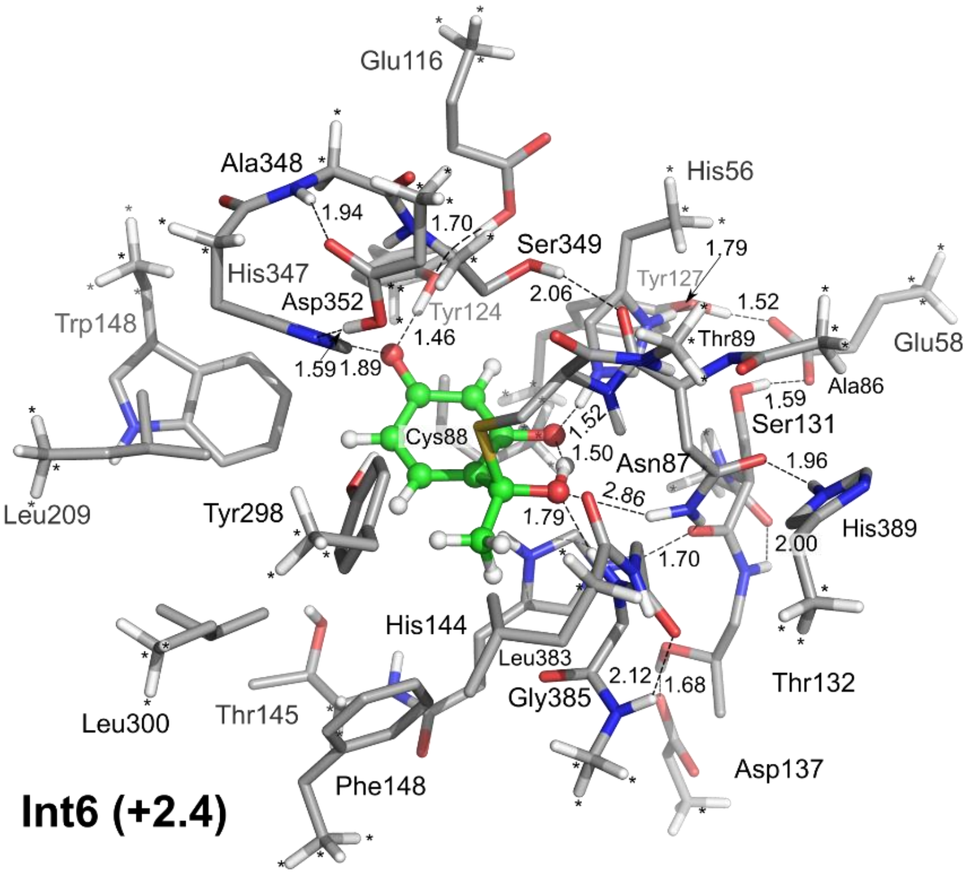

Int6 (+2.4)

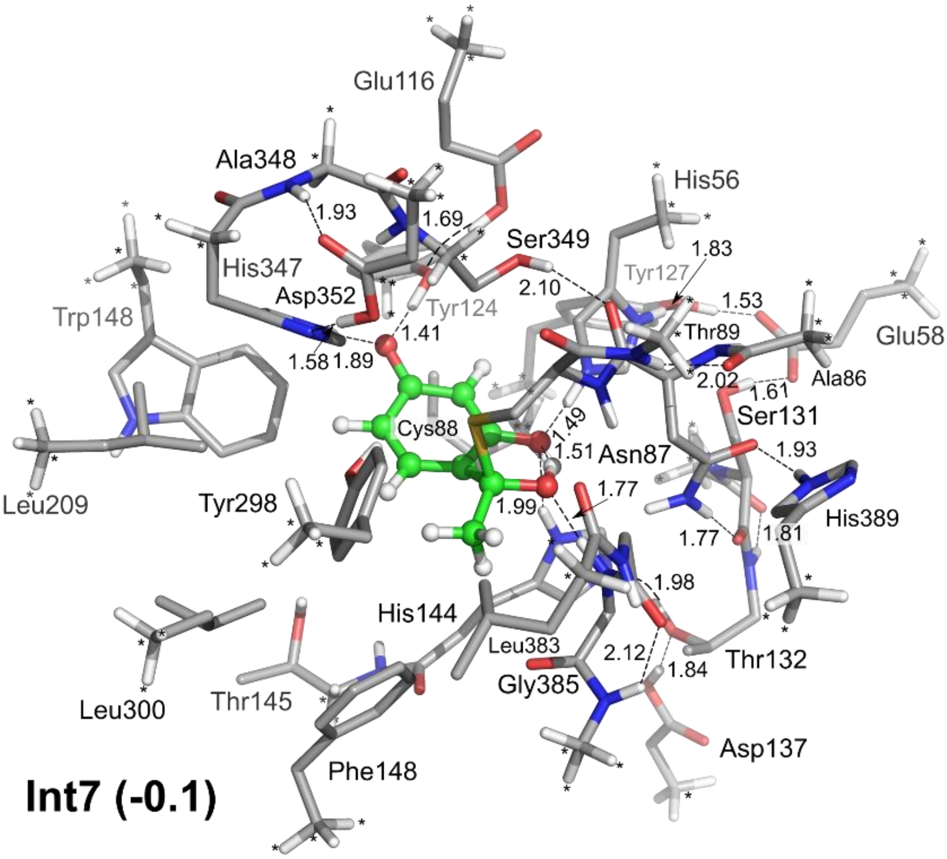

Int7 (-0.1)

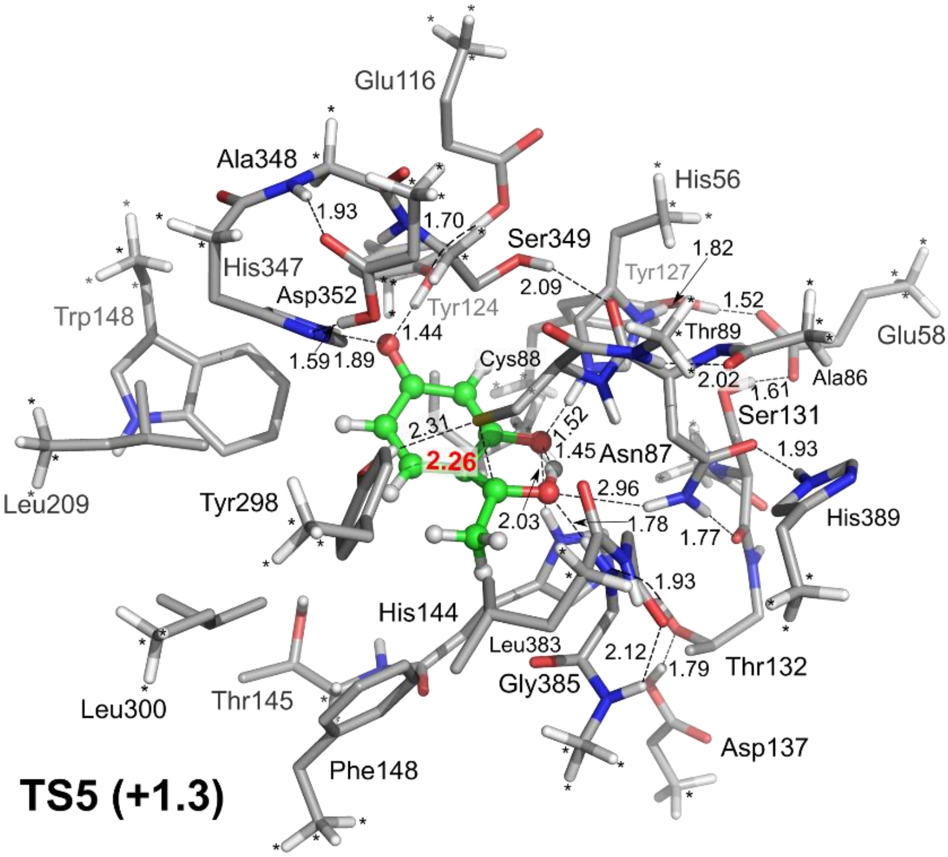

TS5 (+1.3)

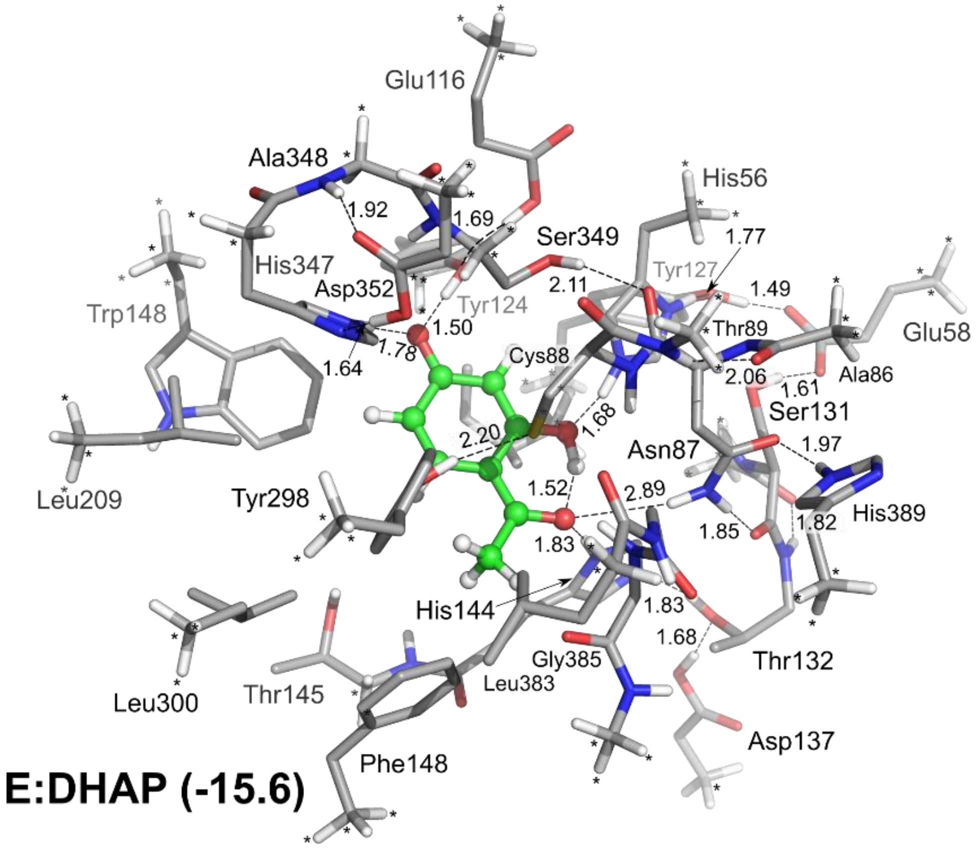

E:DHAP (-15.6)

3. Superposition of the optimized structures

Superposition of the optimized structures of the intermediates and transition states in the reaction pathway of the *Pp*ATase-catalyzed conversion of HPA to DHAP is shown in the figure below.

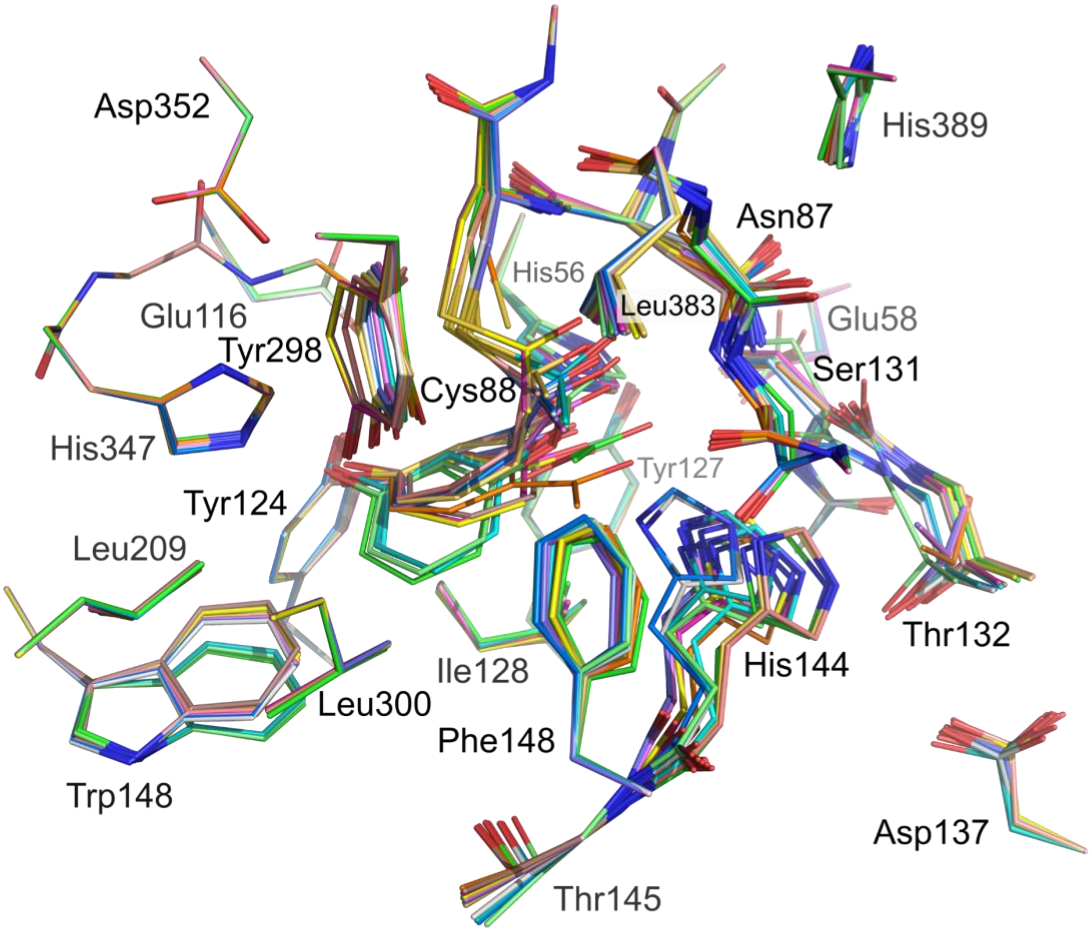

4. Optimized structure of the enzyme model in complex with 4-hexylresorcinol

The lowest-energy structure of the enzyme model in complex with 4-hexylresorcinol (called **E:4HR**) is shown in the figure below.

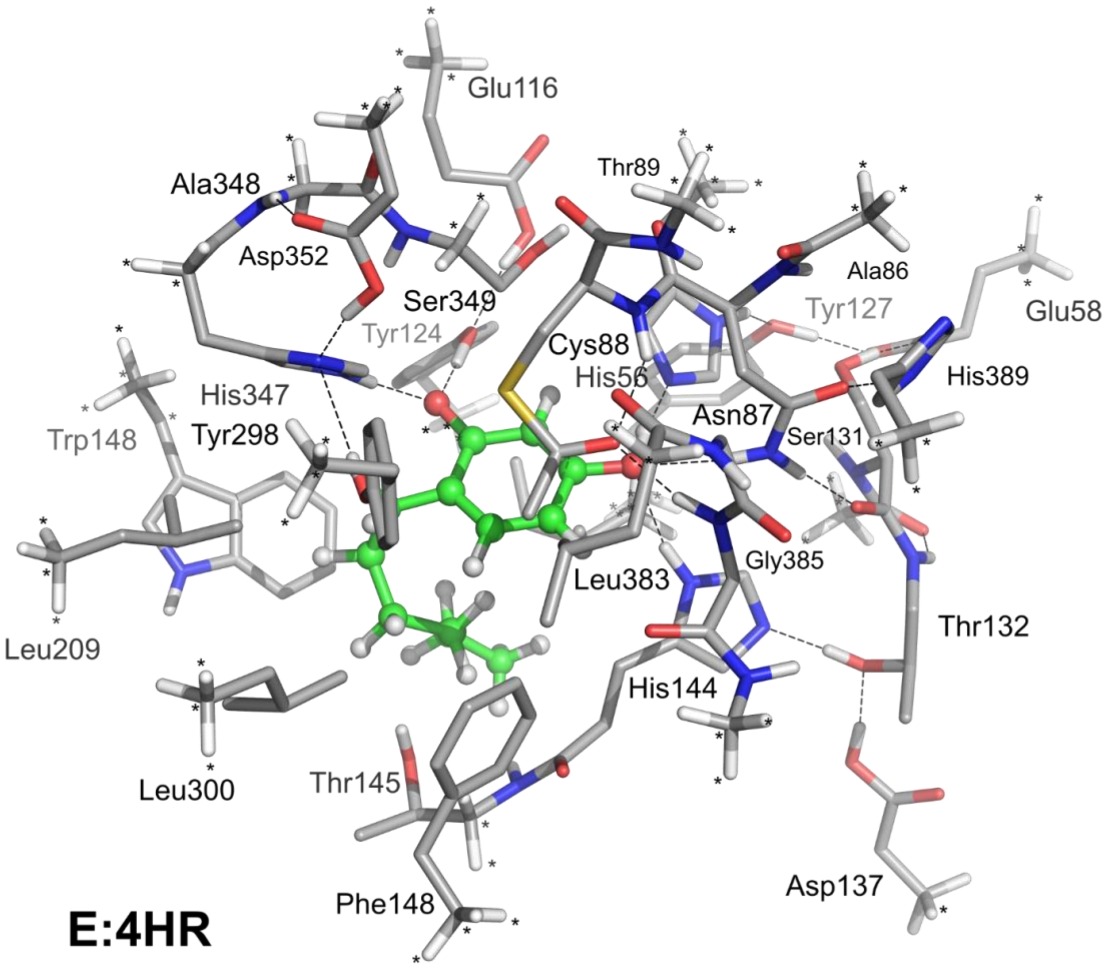

5. Absolute energies and energy corrections

Table S1. *Calculated absolute energies, energy corrections and final relative energies for the suggested reaction pathway. BS1=6-31G(d,p), BS2=6-311+G(2d,2p)*

	$E_{BS1\text{-}Gas}$ (au)	$E_{BS2\text{-}Gas}$ (au)	$E_{BS1\text{-}SMD}$ (au)	E_{ZPE} (au)	E_{total} (au)	ΔE_{total} (kcal/mol)
**E:HPA**	-9161.483688	-9164.013721	-9161.679924	3.537277	-9160.67268	**0.0**
**TS1**	-9161.479136	-9164.005149	-9161.671041	3.537378	-9160.659677	**+8.2**
**Int1**	-9161.477244	-9164.007054	-9161.670669	3.536368	-9160.664111	**+5.4**
**TS2**	-9161.472171	-9164.002496	-9161.665553	3.534542	-9160.661337	**+7.1**
**Int2**	-9161.510141	-9164.033663	-9161.705409	3.536062	-9160.69287	**-12.7**
**Int3**	-9161.488698	-9164.017925	-9161.69285	3.530627	-9160.69145	**-11.8**
**Int4**	-9161.49691	-9164.028486	-9161.700469	3.538363	-9160.693682	**-13.2**
**TS3**	-9161.467781	-9163.99909	-9161.672261	3.535131	-9160.668439	**+2.7**
**Int5**	-9161.491293	-9164.021894	-9161.697999	3.538217	-9160.690383	**-11.1**
**TS4**	-9161.455146	-9163.983453	-9161.66375	3.533916	-9160.658141	**+9.1**
**Int6**	-9161.464951	-9163.995024	-9161.675497	3.536697	-9160.668873	**+2.4**
**Int7**	-9161.490762	-9164.017518	-9161.681264	3.535245	-9160.672775	**-0.1**
**TS5**	-9161.489118	-9164.015468	-9161.679874	3.535591	-9160.670633	**+1.3**
**E:DHAP**	-9161.51289	-9164.040999	-9161.707717	3.538363	-9160.697463	**-15.6**

6. Cartesian coordinates

E:HPA (0.0 kcal/mol)

C	-8.65757600	2.09195300	2.77713800
C	-8.81240300	1.06338500	1.68453300
O	-9.79799700	0.31984200	1.63317300
N	-7.75087400	0.94059900	0.84844600
C	-7.83622000	0.10762200	-0.34749500
C	-7.55589200	-1.36526300	0.01278500
O	-6.53368900	-1.96184400	-0.37041200
C	-6.81303800	0.65386200	-1.36604100
O	-6.72249600	2.06105000	-1.30093800
N	-8.48714300	-1.96644100	0.76976800
C	-8.33800500	-3.34328800	1.20920400
C	-8.71074000	-3.58939800	2.67674000
C	-8.46062300	-5.05953400	3.02231300
O	-8.00897900	-2.71700000	3.56115100
C	-10.14894600	-5.20044000	8.62212400
C	-9.04735100	-4.28128700	8.09232600
C	-9.14249700	-4.01119900	6.59605800
O	-10.05483600	-4.41803000	5.89570700
O	-8.11505000	-3.28496700	6.16053400
C	-1.82559300	-3.65452500	5.67885300
C	-0.56811200	-4.26273100	6.28331400
O	-0.29025400	-5.45222900	6.14541200
C	-1.82167600	-3.74275200	4.14230500
C	-3.13090700	-3.30747800	3.56095900
C	-4.41938100	-3.36680700	4.04058100
N	-3.25400900	-2.74412900	2.30187300
C	-4.57387600	-2.49969800	2.06856000
N	-5.30576000	-2.86799800	3.10530800
N	0.21600000	-3.41089000	7.00266700
C	1.53096000	-3.81630800	7.46691200
C	2.21783000	-2.64409000	8.16932000
C	3.67219000	-2.96214200	8.49913700
O	2.10238700	-1.45091500	7.38737500
N	-4.12389100	3.49115200	-1.56326600
N	-2.57179000	2.14444400	-0.90093300
N	-3.51576200	0.32497900	-5.42628800
N	-4.09535300	-1.59297800	-1.64472000
N	-0.25692100	-0.34283700	-4.60231400
N	0.47967800	-1.46325600	-6.91403200
N	7.04222800	3.19882700	6.16072800
N	6.17007900	1.37936300	-1.41605800
N	4.95956400	2.69092300	-0.11015500
N	7.14771600	4.56513900	-3.24593400
N	3.80690100	4.17823000	-3.08777400
N	0.08181900	-5.75059100	-4.39373500
N	-1.77335200	-4.51291500	-1.54197200
N	-2.04923300	-7.24103100	0.12946200
N	-4.95507600	-4.72459600	-7.38234700
N	-5.12514900	-3.52586900	-5.51293600
C	-3.38981200	4.83536700	-4.52590100
C	-2.81019000	5.07586000	-3.11963800
C	-2.94831200	3.91894000	-2.17781800
C	-1.97062100	3.05661100	-1.75262600
C	-3.87800800	2.43055700	-0.79349000
C	-4.69706000	-0.41948200	-7.41614000
C	-3.46834500	-0.50908100	-6.51045700
C	-2.61699500	0.21095700	-4.27579200
C	-1.17195000	0.63960400	-4.63225000
C	-2.70989000	-1.16489800	-3.58158000
C	-4.07899900	-1.38701800	-2.97024500
C	1.15067700	-0.20486900	-4.91996100
C	1.46186500	-0.72019900	-6.33378600
C	2.01948100	-0.99693800	-3.92115400
C	-0.43898000	-1.60460400	0.22185500
C	0.62201800	-1.92902500	-8.27330800
C	2.15051600	8.96420800	-4.74218400
C	2.55477500	7.62939500	-4.09350400
C	1.80661900	7.23372100	-2.81702300
C	0.32042900	6.97243200	-3.01254300
C	-0.98926900	8.68780500	5.26990400
C	0.16157800	8.96741000	4.27371600
C	0.34694200	7.96583500	3.15850900
C	-0.60571200	7.81003000	2.14249000
C	1.50333600	7.17855200	3.09065700
C	-0.42348800	6.89549300	1.11024400
C	1.71114600	6.26383100	2.05957500
C	0.73620100	6.10619400	1.06680000
C	-5.29615300	5.86386000	5.34313300
C	-5.04384300	7.02660800	4.34709500
C	-5.41556200	6.60528000	2.93625400
C	-6.74636600	6.36683000	2.56383000
C	-4.42534000	6.32553600	1.98603100
C	-7.08403900	5.85566800	1.31108900
C	-4.74127300	5.82440000	0.72349500
C	-6.07625600	5.56889400	0.37315000
C	-3.53477800	2.92061000	3.65377500
C	-2.22509500	2.25125800	4.10390000
C	-1.09387900	3.25585900	4.37328100
C	-1.82141600	1.16511600	3.10351400
C	-0.72868100	4.14659900	3.19013500
C	3.47189600	-8.42803000	6.02417300
C	4.12036800	-7.16222400	5.43062900
C	3.50185200	-6.72953300	4.11900700
C	4.23099000	-6.78259800	2.92476500
C	2.17218200	-6.28798700	4.07408700
C	3.65413600	-6.39477900	1.71252100
C	1.58695000	-5.91598500	2.86400700
C	2.32788000	-5.96331000	1.68032700
C	11.14011100	0.40123600	3.73855000
C	9.99904100	0.05593400	2.76791800
C	8.59153000	0.54170300	3.16345700
C	8.49066400	2.07103800	3.15862700
C	7.53246900	-0.05201000	2.22374400
C	8.39881200	6.09092800	2.97903400
C	7.52119800	6.32761400	4.20308800
C	7.06129800	5.08199200	4.90911500
C	7.82390000	4.23562100	5.67481400
C	5.72334600	4.53739600	4.91450700
C	5.74891300	3.35222800	5.70215700
C	4.49989200	4.94805000	4.35345700
C	4.60332300	2.58261000	5.93401200
C	3.35874300	4.19396300	4.58959600

C	3.41146100	3.02159100	5.37198200
C	8.38008700	-5.62816500	-3.74847300
C	6.83849700	-5.68724700	-3.71271300
C	6.14261700	-4.68052600	-2.82103300
C	5.50571300	-3.57087500	-3.39087300
C	6.06747300	-4.81819900	-1.42674900
C	4.80234700	-2.65094900	-2.62347200
C	5.34783000	-3.91543500	-0.64066600
C	4.67907100	-2.83366600	-1.23865500
C	9.15395800	-5.23292800	2.83208500
C	7.77263400	-4.58499300	2.66009400
C	7.09900400	-4.12927400	3.96826500
C	7.97835900	-3.16244600	4.77276500
C	5.72877600	-3.50157500	3.67907000
C	8.92043100	3.31537600	-2.18492900
C	8.01145700	4.53858400	-2.19647400
C	8.49840100	2.34466900	-1.04281200
C	7.01770800	2.28317100	-0.79578200
C	6.26762100	3.10086200	0.02102400
C	4.94470600	1.64850900	-0.97600200
C	5.94347900	5.37492300	-3.23316500
C	4.84032000	4.54490500	-3.89271500
C	2.93060900	3.08626300	-3.49017600
C	1.68090700	2.97460000	-2.62689900
C	6.32940900	1.71346200	-7.31599100
C	5.85333200	0.78648600	-6.18751600
C	6.32669100	1.17934100	-4.80769900
C	2.03022300	-6.91122800	-5.30224300
C	1.30173900	-5.60033900	-4.99448600
C	2.35747800	-7.72142800	-4.02913600
C	3.28194600	-7.01073300	-3.01653800
C	2.59821300	-5.88442000	-2.22505600
C	3.88653600	-8.03156800	-2.04478900
C	-0.55346400	-4.67445300	-3.66461800
C	-1.44101400	-5.29951800	-2.58543900
C	-2.64888900	-4.98610900	-0.47607300
C	-1.90629900	-5.93846600	0.48779100
C	-1.20134800	-8.37291500	0.52381200
C	-3.10211400	-7.50218500	-6.69770900
C	-2.81668700	-6.00071000	-6.92999500
C	-3.90086100	-5.04176200	-6.54425700
C	-3.99733200	-4.30602900	-5.38284700
C	-5.65630500	-3.81058400	-6.73464400
C	2.13049100	2.28696700	1.21459900
C	2.82267300	1.70779300	2.30805600
C	2.44323100	0.48114700	2.83204600
C	1.06393100	1.54386600	0.65498900
C	0.73816400	0.30593900	1.18381300
C	1.37677900	-0.24408600	2.28999800
C	-12.95553200	5.33690900	-3.02550800
C	-11.47061700	5.00371200	-2.74323700
C	-11.14424400	4.18363200	-1.48390800
C	-9.62300300	3.94399600	-1.35946900
C	0.70085100	-2.56321600	0.41728500
O	-2.51402800	-1.24836100	-6.74094100
O	-0.93375100	1.82058700	-4.90007400
O	-5.11353600	-1.36135600	-3.66033400
O	2.56686100	-0.52259800	-6.83625500
O	-0.31659100	7.30043500	-3.99513300
O	-0.30348900	6.39212600	-1.97795400

O	0.87850100	5.20681800	0.05996700
O	-6.35397000	5.06135500	-0.85593600
O	3.94254300	-2.00152500	-0.46891200
O	8.01866600	5.37990800	-1.29600900
O	4.92313600	4.22280200	-5.07393000
O	0.58820600	3.74130300	-3.15348100
O	7.21614600	2.00050800	-4.60034400
O	5.73010300	0.48350000	-3.85170900
O	1.79082600	-4.49801700	-5.21114800
O	-1.84106300	-6.46724200	-2.69761100
O	-1.25909400	-5.52245400	1.44532600
O	2.52062900	3.44852500	0.71945500
O	-0.38822100	-0.32479900	0.60003500
O	-9.20738500	2.76100300	-1.50046900
O	-8.90818900	4.97257000	-1.15379300
O	-1.53656100	-1.99747700	-0.19954800
S	1.61462700	-0.66646500	-2.15739100
H	-3.25181700	5.97174900	-2.66977200
H	-1.74527800	5.27893300	-3.18907600
H	-0.92061400	3.05958500	-2.01240900
H	-4.61401100	1.91461700	-0.20460500
H	-3.19788100	3.79952000	-4.83000900
H	-5.58381400	-0.72979600	-6.85506300
H	-2.54113300	-1.94423600	-4.33096700
H	-1.93629500	-1.23235600	-2.81055800
H	-5.00572000	-1.73771100	-1.19725300
H	-3.23728000	-1.59261100	-1.10399200
H	-2.94596500	0.98312600	-3.57537100
H	-4.42518900	0.70260000	-5.20400100
H	1.68704600	-1.95570500	-8.50751900
H	-0.46650800	-1.38195400	-6.55963900
H	2.40314700	6.82805600	-4.82463300
H	3.62923100	7.63435300	-3.87153800
H	1.89536000	8.00616600	-2.03975300
H	2.25415200	6.32503500	-2.39964000
H	1.06530100	9.00771000	-4.84753700
H	0.01688700	9.97160800	3.85163500
H	1.09727100	9.01593000	4.84338800
H	2.25953400	7.27846100	3.86634100
H	-1.51028500	8.41417600	2.15649300
H	2.59455000	5.63513000	2.02674700
H	-1.16697300	6.78089900	0.32983600
H	0.29869200	5.91463000	-1.35284000
H	-1.96929700	8.91347100	4.83886300
H	-5.59528300	7.92554300	4.65096500
H	-3.98112800	7.29265800	4.36191000
H	-7.54402200	6.57751200	3.27358800
H	-3.38055000	6.49968300	2.23306200
H	-8.11877900	5.69063400	1.02880500
H	-3.95138200	5.64070800	0.00356300
H	-7.36868400	4.95871500	-1.00245400
H	-6.33362700	5.85552800	5.69266700
H	-2.43709900	1.75144400	5.06147100
H	-1.60757300	1.59867000	2.12225400
H	-0.91602400	0.64204800	3.42150900
H	-2.61931100	0.42337400	2.97667600
H	-1.39331700	3.89059000	5.21926100
H	-0.20124700	2.70541300	4.70152800
H	-1.59330200	4.70442100	2.82004400
H	0.02094100	4.88292600	3.47990400

H	-0.31613400	3.57424900	2.35484700
H	-3.46712500	3.32821100	2.64049400
H	4.03735000	-6.34733900	6.16282200
H	5.19329300	-7.33479500	5.28468600
H	5.26487100	-7.12012800	2.94695900
H	1.58007600	-6.23537600	4.98369500
H	4.23806300	-6.42049700	0.79816900
H	0.54858600	-5.60958200	2.82965200
H	1.87303900	-5.66278300	0.74217700
H	3.51276400	-9.24868200	5.30006600
H	10.23198000	0.47218600	1.77810700
H	9.97200600	-1.03389100	2.63549800
H	8.38587800	0.18490700	4.18599000
H	7.48673400	2.40325500	3.43501200
H	8.69910300	2.46117300	2.15622500
H	9.19352900	2.53936200	3.85331400
H	6.52212900	0.24052800	2.52696300
H	7.67124200	0.30488200	1.19808600
H	7.57622100	-1.14581900	2.20291900
H	12.09838700	0.11426600	3.28745000
H	6.63683000	6.90356900	3.90637100
H	8.06436400	6.96931700	4.91035900
H	8.87658700	4.28360200	5.91411300
H	7.39018100	2.39387200	6.65332900
H	4.44851700	5.84039800	3.73621800
H	2.41629300	4.49135100	4.14465200
H	4.64238100	1.67798500	6.53461200
H	2.50514200	2.44297000	5.51687000
H	7.89534300	5.47050900	2.23213900
H	6.46258200	-5.54609100	-4.73294000
H	6.54335500	-6.70533800	-3.42337200
H	6.56195100	-5.65980400	-0.94362300
H	5.54955500	-3.42328500	-4.46697400
H	5.26675500	-4.04825000	0.43338600
H	4.34591800	-1.78980200	-3.08912600
H	3.30274200	-1.49219000	-1.04999200
H	8.74174600	-4.60961200	-3.91194600
H	7.85496300	-3.72555700	1.98161000
H	7.09968900	-5.29335200	2.16047100
H	6.93677800	-5.02861700	4.58349400
H	8.21392200	-2.26952800	4.18372100
H	7.46149800	-2.83141700	5.68073100
H	8.92375300	-3.62163300	5.07815600
H	5.83155400	-2.59938500	3.06644500
H	5.22551600	-3.21504000	4.61097100
H	5.07235300	-4.19626700	3.14910700
H	9.90839500	-4.50503500	3.14604000
H	8.86034100	1.34075600	-1.28620100
H	8.98720800	2.63779800	-0.10928700
H	6.55304100	3.93806400	0.63630800
H	4.04365600	1.11204900	-1.24955400
H	8.86114000	2.79051300	-3.13887100
H	5.72253400	5.65380900	-2.19916600
H	7.15477300	3.77696800	-3.89214900
H	1.41380700	1.91441000	-2.53659500
H	1.87541900	3.34703100	-1.61836200
H	0.21727200	3.23606300	-3.89973900
H	3.49144100	2.14281200	-3.45879200
H	3.95985700	4.25832500	-2.09024300
H	4.76514700	0.66795100	-6.17791200

H	6.23996800	-0.23165900	-6.33583700
H	7.28951400	2.16492900	-7.05634100
H	1.43123400	-8.02586800	-3.52145600
H	2.84237500	-8.65208400	-4.35122300
H	4.10601400	-6.55625900	-3.58043600
H	1.67596700	-6.24606700	-1.75110900
H	2.36405800	-5.02447300	-2.85176000
H	3.26784800	-5.51812100	-1.44361100
H	4.39802500	-8.84398000	-2.57436300
H	4.61265200	-7.54536100	-1.38638500
H	3.11079700	-8.47917400	-1.41074000
H	1.42948700	-7.51931500	-5.98948500
H	-1.17927400	-4.05543900	-4.32264100
H	0.20221100	-4.01075600	-3.22841000
H	-0.27865400	-6.66877700	-4.16749600
H	-3.51786400	-5.47968700	-0.91917400
H	-2.98661600	-4.12044400	0.08553400
H	-1.39054800	-3.58012100	-1.41420200
H	-0.13888000	-8.11664900	0.44153200
H	-2.39554900	-7.32762600	-0.82061300
H	-2.56978900	-5.83675500	-7.98524500
H	-1.91847800	-5.74121400	-6.35792300
H	-3.39445500	-4.28282100	-4.48987400
H	-5.38006800	-2.77575900	-4.86761500
H	-6.55034100	-3.32556500	-7.10158400
H	-3.79772000	-7.91855000	-7.43012900
H	-10.90029500	5.93757800	-2.68585400
H	-11.07432700	4.46247100	-3.61281800
H	-11.48157000	4.72909800	-0.59343500
H	-11.65105000	3.21418900	-1.50659600
H	-13.56785300	4.43615900	-3.13767600
H	1.39875800	0.85630300	-4.88669500
H	1.91927600	-2.06479300	-4.14809500
H	3.05168300	-0.71886600	-4.14265700
H	1.56438900	4.45760800	0.30336000
H	3.65059500	2.25786000	2.73611800
H	0.54415900	1.91418800	-0.21794200
H	-2.50617100	-2.57352000	1.63759100
H	-5.04569900	3.96392900	-1.55335500
H	6.07270300	0.76069600	-2.92883800
H	1.67174300	-2.09292000	0.53608400
H	0.46925700	-3.18705200	1.28997000
H	0.73829800	-3.21309300	-0.45547000
H	4.10591500	3.10589200	0.31947500
H	-0.52585900	-1.24156800	-4.23716400
H	-13.39573800	5.96507100	-2.24419300
H	-12.95113700	5.88675600	-3.97199200
H	-5.15065800	4.96312400	4.73718700
H	-4.63457200	5.88126600	6.21591100
H	-4.27939100	2.11774500	3.65697800
H	-3.80721800	3.74107100	4.32591800
H	-0.98477800	7.63291800	5.56309900
H	-0.80738100	9.34863700	6.12388800
H	-4.47220700	4.99880200	-4.52735200
H	-2.88882000	5.45871700	-5.27449900
H	2.48164600	9.85437100	-4.19664400
H	2.58069600	8.99615200	-5.74881600
H	8.63748300	7.04985700	2.50899900
H	9.32502300	5.58127400	3.26200400
H	6.01867000	6.29635700	-3.81991300

H	2.58583500	3.21486100	-4.52163000
H	9.95161100	3.64331600	-2.01978200
H	5.59999100	2.51265700	-7.48209200
H	6.45574000	1.08947600	-8.20604400
H	0.13324100	-1.23120800	-8.96179200
H	0.19371500	-2.92220200	-8.43950100
H	-4.84392900	0.61883500	-7.73386900
H	-4.59462100	-1.05872800	-8.30044900
H	-3.51031000	-7.64088100	-5.69305300
H	-2.13230100	-8.00759800	-6.75436800
H	2.96778100	-6.64047600	-5.79910200
H	8.73076900	-6.27026200	-4.56281900
H	8.79436300	-6.00965000	-2.80940500
H	-1.46871400	-9.10261800	-0.24746600
H	-1.36424000	-8.78300600	1.52665500
H	2.41849000	-8.24425200	6.25297800
H	4.01821100	-8.74307400	6.91949600
H	9.49257600	-5.67774900	1.89030700
H	9.13101700	-6.03092100	3.58155900
H	11.07359200	-0.16268100	4.67453800
H	11.20830400	1.47755000	3.93235500
H	-2.09617800	1.38063100	-0.43111400
H	-7.18481100	1.77141200	0.69647100
H	-8.85175800	0.19003500	-0.75185500
H	-9.24086500	-1.37135500	1.12268200
H	-8.83651300	-5.27522300	4.02312200
H	-8.97085000	-5.72260500	2.31470900
H	-7.38741500	-5.27508900	2.98126500
H	-9.77245100	-3.35999000	2.82781400
H	-7.02991400	-2.75488900	3.35556800
H	-7.65139800	2.44179200	-1.44220500
H	-9.06448000	-3.30845600	8.59929200
H	-8.05096800	-4.69382000	8.29003300
H	-1.01401600	-3.11856500	3.73964000
H	-1.59434100	-4.76861700	3.83575200
H	-4.76917900	-3.72797000	4.99621700
H	1.06136200	-1.18948400	2.71290500
H	-4.96167300	-2.07877700	1.15292100
H	-8.18697900	-3.09321600	5.17250400
H	0.08816200	-2.41227400	6.89973100
H	1.67029200	-2.41116300	9.09008200
H	4.23619300	-3.17804000	7.58345700
H	4.14148300	-2.11203000	9.00038200
H	3.74701300	-3.83892200	9.15066400
H	-10.04588200	-5.34519700	9.70188900
H	-9.51647200	1.97082300	3.44102600
H	-8.64661900	3.14272400	2.46679400
H	-7.76372700	1.86290200	3.36470400
H	-11.13606000	-4.77861200	8.41701400
H	-10.10756800	-6.17813200	8.13444100
H	-8.95851900	-4.00220000	0.58611300
H	-7.29764400	-3.62648500	1.03649100
H	-5.82607700	0.25248700	-1.12624500
H	-7.06715300	0.28620800	-2.36703900
H	2.14569000	-4.14627300	6.61399500
H	-2.66746600	-4.23571300	6.06877900
H	1.45405800	-4.68027300	8.13561300
H	-1.96986800	-2.61732700	5.99844100
H	2.60563200	-1.58261700	6.56991700
H	2.98488400	0.07760400	3.68335300

TS1 (+8.2)

C	-8.81169500	1.80165000	2.91495800
C	-8.89132200	0.70503200	1.88009700
O	-9.44398400	-0.37124000	2.14381000
N	-8.23406200	0.90361300	0.71324900
C	-8.05291000	-0.21321100	-0.19137700
C	-7.01629200	-1.17026400	0.42514300
O	-5.79573000	-0.96491200	0.29742900
C	-7.63470900	0.26988100	-1.61420200
O	-7.18226600	1.60616000	-1.63539300
N	-7.55228500	-2.21491300	1.08202100
C	-6.81404300	-3.34094500	1.63101700
C	-7.25228700	-3.74936200	3.04755000
C	-6.65059500	-5.10548700	3.42482200
O	-6.93066700	-2.76318800	4.02607500
C	-9.76611800	-5.24272600	9.16254700
C	-9.15635100	-3.99259300	8.51028300
C	-8.53635000	-4.20424800	7.12887800
O	-8.48975700	-5.27510600	6.55232500
O	-8.05283700	-3.05306700	6.64602800
C	-0.71785600	-4.47430400	5.02489100
C	0.39445100	-4.56661500	6.06136100
O	0.94176900	-5.62698100	6.36455600
C	-0.60775800	-3.33675600	3.98510600
C	-1.95978800	-2.88410200	3.52711600
C	-3.21566100	-3.09517500	4.04859700
N	-2.16756000	-2.03284900	2.45254200
C	-3.50392700	-1.75937300	2.38324800
N	-4.16622200	-2.39179700	3.33753100
N	0.68588500	-3.40579400	6.70811600
C	1.79061100	-3.29963200	7.63458500
C	1.71405300	-1.95560600	8.36163100
C	2.94276400	-1.70362400	9.22820900
O	1.49960700	-0.89248200	7.42779400
N	-4.53091700	2.96568400	-1.73679100
N	-2.92677400	1.62597600	-1.21114600
N	-3.82760400	-0.35428700	-5.18987300
N	-3.76383500	-1.93533300	-1.30632300
N	-0.48046500	-0.96192300	-4.35390200
N	0.25514600	-1.80284700	-6.79887800
N	6.94436300	3.95146100	5.87496100
N	5.89616800	1.74111700	-1.64093400
N	4.69946800	2.99760600	-0.26931200
N	6.69361400	4.79979500	-3.63299300
N	3.40302400	4.21807400	-3.36810100
N	0.10529300	-6.05430800	-4.08654700
N	-1.50832400	-4.66278900	-1.23302900
N	-1.71986700	-7.24796300	0.61049700
N	-4.86953600	-5.07197500	-6.97312700
N	-5.27486000	-4.27014600	-4.93364900
C	-3.87266400	4.43494500	-4.65350900
C	-3.29432100	4.68232400	-3.24325800
C	-3.38102800	3.48261000	-2.33958200
C	-2.37094500	2.62463600	-1.99334900
C	-4.24302700	1.84854100	-1.06266700
C	-4.95738900	-1.02680600	-7.23587000
C	-3.71985500	-1.05265600	-6.35716900
C	-2.88363800	-0.56381000	-4.09624700

C	-1.46865800	-0.05803500	-4.46698900
C	-2.97975000	-2.01799000	-3.56900500
C	-4.13342100	-2.09450600	-2.59010600
C	0.91256100	-0.74254400	-4.67625800
C	1.28286000	-1.29102600	-6.07829300
C	1.80378500	-1.35914700	-3.58680800
C	0.13989100	-1.64960300	-0.64641100
C	0.41492800	-2.29308200	-8.15263400
C	1.42712400	8.83663000	-5.22498100
C	1.93501900	7.57099600	-4.51076500
C	1.25650600	7.20929400	-3.18341100
C	-0.19616300	6.77208300	-3.32352800
C	-1.45966300	8.91942300	4.86642300
C	-0.34369500	9.20142600	3.82781700
C	-0.10509700	8.14224000	2.77501500
C	-1.04990800	7.86748400	1.77661200
C	1.09519600	7.41969000	2.74587800
C	-0.81972400	6.90024900	0.80283300
C	1.34895500	6.45359100	1.77300100
C	0.38245900	6.17399800	0.79939400
C	-5.60291200	5.87728500	5.19612900
C	-5.39628900	7.00731900	4.15088000
C	-5.78101300	6.49676000	2.77804400
C	-7.11642200	6.20532000	2.47150300
C	-4.80717300	6.12715500	1.84239800
C	-7.47383600	5.53513700	1.30593300
C	-5.14256900	5.45602100	0.66561700
C	-6.47848900	5.12650100	0.40127800
C	-3.72292300	2.94758800	3.61448900
C	-2.33683300	2.42058100	4.03572700
C	-1.35354400	3.55101600	4.37893900
C	-1.77654100	1.46902400	2.97479300
C	-1.03560600	4.48488600	3.21544400
C	3.94681500	-7.87046800	6.37985100
C	4.53811900	-6.61214400	5.71312300
C	4.18170800	-6.45136400	4.25215300
C	5.17367300	-6.51521400	3.26689200
C	2.85562500	-6.23325700	3.85354800
C	4.85742100	-6.33139200	1.91962800
C	2.53481700	-6.04711100	2.50904800
C	3.53924300	-6.08287300	1.53920400
C	11.06575400	1.22331700	3.44644500
C	9.91673000	0.77439600	2.52883500
C	8.49825700	1.20967400	2.94422600
C	8.32480400	2.73163400	2.88687800
C	7.44490300	0.53049000	2.05709200
C	8.00055700	6.71064700	2.46806700
C	7.14002100	6.96423200	3.70091000
C	6.79284400	5.73470800	4.49301400
C	7.62498400	5.02604300	5.32332600
C	5.51747000	5.05709500	4.52294200
C	5.65053000	3.93884500	5.39265500
C	4.27055100	5.29537500	3.91597300
C	4.58724900	3.07020800	5.66175100
C	3.21021300	4.44155700	4.18627500
C	3.36892000	3.33864000	5.05142400
C	8.46658700	-5.32984400	-3.64765000
C	6.93098300	-5.39983000	-3.56155000
C	6.28537000	-4.36374100	-2.66468700
C	5.68406700	-3.22444000	-3.21473600
C	6.24653200	-4.50271900	-1.26964200
C	5.04445300	-2.27590600	-2.42207200
C	5.58771700	-3.57726700	-0.45995900
C	4.95651700	-2.46533000	-1.03682300
C	9.37011800	-4.54873000	2.88166900
C	7.95677400	-3.98030500	2.69431800
C	7.26896300	-3.47934500	3.97702000
C	8.09490000	-2.40836000	4.70153000
C	5.85868800	-2.96145100	3.66055000
C	8.55315400	3.70190600	-2.55612700
C	7.57780100	4.87230400	-2.60241100
C	8.21157400	2.76899600	-1.35781400
C	6.74229300	2.66268000	-1.04727300
C	6.00013500	3.44681200	-0.18861900
C	4.68365300	1.96669400	-1.14653400
C	5.44464600	5.53921600	-3.63329400
C	4.38014700	4.61515200	-4.22499400
C	2.55622800	3.08262900	-3.69431500
C	1.30205400	3.00891000	-2.81373300
C	5.93532900	1.69414300	-7.53188800
C	5.55710100	0.80466400	-6.33924300
C	6.03031400	1.32304700	-5.00140700
C	2.16200100	-7.03006200	-4.96798300
C	1.26809600	-5.80403400	-4.76555700
C	2.59484300	-7.68961300	-3.64118500
C	3.52423600	-6.82566700	-2.76703500
C	2.83367800	-5.60257100	-2.14481900
C	4.17417900	-7.68860500	-1.67915600
C	-0.67195200	-4.98551300	-3.49053900
C	-1.40035800	-5.52405400	-2.25707800
C	-2.24476000	-4.96823900	-0.01153800
C	-1.46628200	-5.94472700	0.89183000
C	-0.84871300	-8.35399800	1.00736900
C	-2.96135500	-7.96670300	-6.19737200
C	-2.80830400	-6.46931200	-6.53874600
C	-3.93118400	-5.58043400	-6.09349100
C	-4.17216100	-5.09350700	-4.82524300
C	-5.64327100	-4.28588800	-6.24545000
C	1.90450900	2.28272100	1.05794200
C	2.66592600	1.72507500	2.11599600
C	2.42598900	0.43817600	2.57712100
C	0.90568800	1.46215600	0.47906300
C	0.72552900	0.15435700	0.92241000
C	1.44420200	-0.37014700	1.99542400
C	-13.41411700	4.50314400	-2.93248700
C	-11.91216300	4.21399300	-2.70900700
C	-11.48247000	3.59374600	-1.37166500
C	-9.96486100	3.31132000	-1.35664100
C	1.09308500	-2.70315300	-0.11340100
O	-2.69704700	-1.66314900	-6.66337900
O	-1.30778900	1.11943000	-4.79519400
O	-5.31735900	-2.21637300	-2.95260800
O	2.44655400	-1.22378200	-6.47039000
O	-0.89373500	7.01929900	-4.28807700
O	-0.71919100	6.12673300	-2.27222500
O	0.57605100	5.22317600	-0.14985600
O	-6.78713300	4.42149800	-0.72152300
O	4.28549000	-1.60581900	-0.22715800
O	7.55596300	5.75503000	-1.74356900
O	4.44290800	4.25239000	-5.39574900

O	0.12227500	3.41925000	-3.51366400
O	6.88946100	2.19249900	-4.87867700
O	5.47650600	0.68578400	-3.98364900
O	1.57307800	-4.67322000	-5.12849500
O	-1.86068600	-6.67877200	-2.25285500
O	-0.68238100	-5.55815400	1.75748600
O	2.19324300	3.49587000	0.60542700
O	-0.24986900	-0.62760300	0.27389300
O	-9.49645100	2.64011100	-2.31440700
O	-9.28482000	3.78045100	-0.38960500
O	-0.84441000	-2.02518800	-1.34809900
S	1.71966000	-0.47292300	-1.97825400
H	-3.77912400	5.54368300	-2.77091400
H	-2.23777200	4.93176100	-3.30945000
H	-1.33287600	2.66082300	-2.28775800
H	-4.94921600	1.22621600	-0.53147000
H	-3.65657100	3.39791100	-4.94208500
H	-5.82700500	-1.34686100	-6.65377800
H	-3.12196100	-2.70169200	-4.40485000
H	-2.08252000	-2.28746700	-3.02061800
H	-4.49390800	-1.78973700	-0.60826100
H	-2.77286300	-1.84033000	-1.08407200
H	-3.19224800	0.12165400	-3.30387200
H	-4.74434600	-0.02734600	-4.92166300
H	1.47925800	-2.27499000	-8.39108900
H	-0.69219400	-1.73869300	-6.44534400
H	1.81419300	6.71690900	-5.18569500
H	3.01322700	7.66025600	-4.32793000
H	1.26994000	8.05528500	-2.48124200
H	1.81276400	6.40061900	-2.69694200
H	0.33866900	8.80828600	-5.29838500
H	-0.55821100	10.16397000	3.34302000
H	0.59523900	9.34723200	4.37511400
H	1.84964900	7.61033300	3.50621800
H	-1.98892200	8.41660900	1.76038300
H	2.27074800	5.88259200	1.76788000
H	-1.56080800	6.69189300	0.03927100
H	-0.06584100	5.78788300	-1.60869200
H	-2.45896300	9.06903500	4.44713100
H	-5.97038900	7.90193400	4.42431100
H	-4.34080700	7.30116800	4.13929100
H	-7.89756300	6.48682300	3.17505400
H	-3.76065700	6.34495000	2.04233700
H	-8.50906700	5.30157700	1.08387900
H	-4.36766300	5.16927100	-0.03781800
H	-7.76062100	4.12821800	-0.67605500
H	-6.63404700	5.84804700	5.56113000
H	-2.48065800	1.83857200	4.95827800
H	-1.61257600	1.98429100	2.02346600
H	-0.81669100	1.03848300	3.27649700
H	-2.47448000	0.64695300	2.79438300
H	-1.77421600	4.13522200	5.20977700
H	-0.42065600	3.10852600	4.75524300
H	-1.94394800	4.91617800	2.78581200
H	-0.40660000	5.31622000	3.53688500
H	-0.50473300	3.96780600	2.41211100
H	-3.72449900	3.31350000	2.58269200
H	4.19901000	-5.72972300	6.27037600
H	5.63082100	-6.63510900	5.80242100
H	6.20633000	-6.68935600	3.56016200
H	2.07425300	-6.20097300	4.60495000
H	5.64074500	-6.36869700	1.16854700
H	1.50241600	-5.87357400	2.22131000
H	3.29820700	-5.91344800	0.49813400
H	4.02752100	-8.72973000	5.70517800
H	10.10057400	1.15334800	1.51387700
H	9.94038600	-0.32051800	2.44850700
H	8.33584500	0.88384900	3.98445700
H	7.31088900	3.02506200	3.17054000
H	8.49757100	3.09562400	1.86767800
H	9.01568700	3.25768900	3.55170500
H	6.42937000	0.78770300	2.37435200
H	7.54255900	0.85173700	1.01512600
H	7.53804300	-0.55998600	2.07820600
H	12.02524600	0.96249400	2.98247500
H	6.20913400	7.45740000	3.39718300
H	7.65554000	7.68369700	4.35137900
H	8.66303800	5.19930100	5.56912400
H	7.36376300	3.22179000	6.42567100
H	4.13998400	6.13350700	3.23754400
H	2.25226700	4.60133400	3.70528300
H	4.71097200	2.21771800	6.32401400
H	2.52510700	2.67736200	5.21925200
H	7.51498600	6.02597600	1.76687600
H	6.51553600	-5.28724700	-4.56987700
H	6.64761800	-6.40628500	-3.22692200
H	6.71887000	-5.36690800	-0.80591800
H	5.70579400	-3.07208800	-4.29082800
H	5.52172000	-3.71766800	0.61291600
H	4.62509400	-1.38433600	-2.87094200
H	3.62872100	-1.10466100	-0.76563000
H	8.80026700	-4.31217200	-3.87148000
H	7.99130400	-3.16033600	1.96444200
H	7.31580400	-4.74781100	2.24623900
H	7.16738500	-4.34261200	4.65323400
H	8.26059100	-1.54177900	4.05293200
H	7.57288200	-2.05422600	5.59765900
H	9.07389500	-2.78504800	5.01475500
H	5.89383700	-2.11821600	2.96198700
H	5.35341000	-2.61858300	4.57134500
H	5.24013600	-3.74302700	3.21241400
H	10.09270500	-3.76809800	3.13983100
H	8.61093000	1.77032600	-1.55917500
H	8.70507400	3.13648700	-0.45354600
H	6.28605500	4.27972200	0.43195400
H	3.79050000	1.40777700	-1.39046400
H	8.50237300	3.12910500	-3.48250800
H	5.22887400	5.85725600	-2.60953500
H	6.73593900	3.98274900	-4.24073100
H	1.19238800	1.98695100	-2.44080300
H	1.39762900	3.66889700	-1.94971000
H	-0.15160100	2.69301200	-4.10256400
H	3.13924600	2.15558100	-3.61766200
H	3.57882900	4.35657500	-2.38083100
H	4.48419500	0.59692500	-6.28497300
H	6.02403000	-0.18560100	-6.43749700
H	6.87468000	2.21297700	-7.32825700
H	1.71875100	-7.99096800	-3.05039500
H	3.12058100	-8.61968600	-3.89354100
H	4.32487600	-6.44863100	-3.41626800

H	1.94454700	-5.89936300	-1.57384600
H	2.53773100	-4.87570700	-2.90076000
H	3.51962100	-5.08506700	-1.47161400
H	4.69406600	-8.55284300	-2.10915600
H	4.90098600	-7.10504400	-1.10860200
H	3.42658400	-8.06494500	-0.97080300
H	1.62613100	-7.76234300	-5.58525600
H	-1.42384900	-4.59457500	-4.18934900
H	-0.00742400	-4.15904600	-3.22705600
H	-0.13157200	-6.98455300	-3.77024000
H	-3.22736400	-5.38070900	-0.26299500
H	-2.38229200	-4.03579000	0.53479300
H	-1.12675400	-3.70385700	-1.32010500
H	0.20495500	-8.07427600	0.88959500
H	-2.15918000	-7.36958600	-0.29858900
H	-2.68471400	-6.34027100	-7.61857000
H	-1.88057800	-6.11552500	-6.07423900
H	-3.69446100	-5.29078000	-3.87840800
H	-5.55978600	-3.59247500	-4.21995300
H	-6.48319800	-3.71445000	-6.61439900
H	-3.64750600	-8.48071000	-6.87609800
H	-11.36472700	5.15759700	-2.83660600
H	-11.55151300	3.55328900	-3.50414800
H	-11.73117800	4.24583400	-0.52886500
H	-12.00309700	2.63812700	-1.22168300
H	-14.01593000	3.58902500	-2.96125100
H	1.07730300	0.33641200	-4.71631700
H	1.54824500	-2.41863800	-3.46723200
H	2.82975300	-1.32995600	-3.95381400
H	1.24317400	4.46407800	0.16047200
H	3.44470900	2.33353600	2.55716500
H	0.32426400	1.83204800	-0.35512100
H	-1.45665600	-1.62352400	1.84776700
H	-5.45981200	3.40577000	-1.66425600
H	5.81545900	1.05584400	-3.08433600
H	1.97696900	-2.28964000	0.36266200
H	0.54925400	-3.34177800	0.59818200
H	1.41507400	-3.32566400	-0.94946900
H	3.83935000	3.35119700	0.20501400
H	-0.70669800	-1.88305900	-4.02148300
H	-13.82801300	5.17161500	-2.17007300
H	-13.46176200	5.00194700	-3.90567200
H	-5.42248300	4.95500900	4.63355500
H	-4.92319600	5.97582300	6.04938400
H	-4.42224800	2.10739200	3.67816900
H	-4.02414000	3.78651700	4.25058800
H	-1.39077300	7.88311000	5.21301600
H	-1.29450000	9.63296500	5.68038700
H	-4.96213500	4.54002800	-4.63517500
H	-3.42402400	5.04394200	-5.44582700
H	1.72164500	9.77063800	-4.73454000
H	1.83144700	8.83856900	-6.24277500
H	8.17556900	7.65505700	1.94355900
H	8.95962600	6.26686400	2.75265800
H	5.45570700	6.43126800	-4.26820900
H	2.18112000	3.13826900	-4.72166800
H	9.56815700	4.09234200	-2.43235600
H	5.15966000	2.44342200	-7.71969300
H	6.07491200	1.03189800	-8.39183200
H	-0.12708400	-1.65958300	-8.86302400
H	0.03786600	-3.31516800	-8.25658700
H	-5.16811500	-0.01595700	-7.60242600
H	-4.84068900	-1.70526700	-8.08864600
H	-3.33792900	-8.07402500	-5.17669000
H	-1.96692300	-8.42190400	-6.25331900
H	3.07155900	-6.73609800	-5.50227500
H	8.83280200	-5.99428000	-4.43686000
H	8.92272200	-5.63889600	-2.70136900
H	-1.09370900	-9.13652700	0.28166900
H	-0.96572800	-8.71905600	2.03376600
H	2.89058200	-7.73147500	6.62631900
H	4.53019700	-8.10844700	7.27571800
H	9.71062700	-5.02367800	1.95543400
H	9.40826100	-5.30630100	3.67139500
H	11.05192600	0.70658900	4.41149000
H	11.07947300	2.31042100	3.58306300
H	-2.40900000	0.85015000	-0.81140800
H	-7.91703500	1.81514600	0.40003200
H	-9.00278100	-0.75209200	-0.26414700
H	-8.52449800	-2.08391900	1.34927900
H	-6.99337100	-5.39156200	4.42243000
H	-6.95200200	-5.88699300	2.71850700
H	-5.55670400	-5.04784200	3.42538100
H	-8.34768100	-3.83093800	3.06509100
H	-5.98077700	-2.52407300	3.88637300
H	-7.97999700	2.14136700	-1.92933400
H	-9.90679100	-3.20016400	8.40367600
H	-8.37379500	-3.56162500	9.14667200
H	-0.06686600	-2.47065900	4.38947600
H	-0.02602900	-3.69830800	3.13286000
H	-3.50309900	-3.71048700	4.88698500
H	1.27153600	-1.38319400	2.33722300
H	-3.95602300	-1.12548000	1.63534100
H	-7.66282600	-3.14251400	5.73742300
H	0.32257400	-2.52429200	6.37193000
H	0.81236500	-1.94626700	8.98458700
H	3.85251900	-1.69722300	8.61556400
H	2.85909800	-0.73630900	9.72962700
H	3.05779600	-2.48433800	9.98725000
H	-10.17839100	-4.99404700	10.14456900
H	-9.64086800	1.64534100	3.60912500
H	-8.86542400	2.83377700	2.55106700
H	-7.89327900	1.65185300	3.49004800
H	-10.56545000	-5.65572100	8.54146300
H	-9.01212600	-6.02461800	9.28638600
H	-6.95527000	-4.20859300	0.97175200
H	-5.75309600	-3.09443400	1.62863200
H	-6.84833300	-0.39186300	-1.99025100
H	-8.50977400	0.17283100	-2.26528700
H	2.75286800	-3.37209000	7.09978500
H	-0.77758900	-5.43425700	4.51152600
H	1.77665900	-4.13003400	8.34841900
H	-1.63765800	-4.35317400	5.60729400
H	2.28714500	-0.82398400	6.86794600
H	3.02621000	0.04457700	3.39359300

Int1 (+5.4)

C	-8.72598100	2.02247300	2.84759000
C	-8.85320300	0.93750200	1.80600000

O	-9.79715900	0.13955300	1.81864400
N	-7.80452200	0.82151300	0.95429900
C	-7.86842500	-0.09025000	-0.18254300
C	-7.48933600	-1.51951400	0.25807200
O	-6.45330000	-2.07832000	-0.13697900
C	-6.90404000	0.44057600	-1.26825700
O	-6.83081600	1.85024400	-1.25746700
N	-8.34750500	-2.13002300	1.09318500
C	-8.09117100	-3.47858200	1.57011300
C	-8.29690500	-3.69643900	3.07380400
C	-7.88305700	-5.12675200	3.43020200
O	-7.58938000	-2.74218600	3.86648600
C	-9.99367700	-5.10355300	8.94547900
C	-8.89120300	-4.16634200	8.44078800
C	-8.87202000	-3.97487000	6.92758700
O	-9.69794100	-4.45812500	6.17285300
O	-7.84089500	-3.21821700	6.54435100
C	-1.60557700	-3.59691500	5.62736600
C	-0.34389800	-4.11400800	6.30014800
O	0.03876100	-5.27534600	6.17967500
C	-1.41403100	-3.29832900	4.12695000
C	-2.71779200	-2.89619000	3.50956900
C	-4.01584500	-3.10601500	3.91194400
N	-2.83194500	-2.19811400	2.31653100
C	-4.16347400	-2.02032700	2.05578100
N	-4.90435500	-2.56308900	3.00456400
N	0.32581500	-3.20669900	7.06850700
C	1.64007500	-3.51438400	7.60154400
C	2.22296400	-2.27956700	8.29084500
C	3.67868900	-2.49431400	8.69111600
O	2.06790700	-1.12014200	7.46672100
N	-4.23965900	3.28154800	-1.60873600
N	-2.62955800	1.95334900	-1.04800900
N	-3.60389500	0.12115800	-5.40124900
N	-4.16965800	-1.88224200	-1.65031700
N	-0.34889900	-0.69011400	-4.36636600
N	0.35737000	-1.68852500	-6.81465900
N	7.03777500	3.59603300	6.04799600
N	6.03543500	1.54681900	-1.52003000
N	4.84978400	2.79170700	-0.12673700
N	6.95238500	4.62840600	-3.44692600
N	3.61974200	4.17134500	-3.22763700
N	0.03466800	-5.95648400	-4.21286400
N	-1.63713500	-4.58251400	-1.36901900
N	-1.91271800	-7.21862100	0.44135700
N	-4.96155500	-4.99852700	-7.21966600
N	-5.26373200	-3.83302000	-5.34658400
C	-3.60320800	4.63390100	-4.60533400
C	-3.02899400	4.91597400	-3.20237500
C	-3.10208600	3.74886600	-2.26492800
C	-2.08954400	2.89949100	-1.90152500
C	-3.93622000	2.20718000	-0.87779800
C	-4.83180900	-0.73926300	-7.30640100
C	-3.56867900	-0.74587300	-6.45953600
C	-2.71074500	-0.05047900	-4.25453500
C	-1.24283700	0.29182400	-4.61770500
C	-2.88248400	-1.43676600	-3.61826700
C	-4.23171700	-1.61583200	-2.96947600
C	1.05830600	-0.62029700	-4.69702200
C	1.39111800	-1.17396600	-6.10800900
C	1.86418800	-1.38312600	-3.63304500
C	0.33449300	-1.68508300	-0.99019900
C	0.50779500	-2.16462700	-8.17938500
C	1.84512300	8.86819800	-5.02448700
C	2.29465800	7.57018900	-4.33080100
C	1.58210200	7.20797300	-3.02284700
C	0.10873800	6.86216000	-3.18671300
C	-1.17121800	8.85539700	5.02928600
C	-0.03194500	9.10912700	4.00865700
C	0.16990000	8.06125800	2.93583800
C	-0.78317200	7.84144500	1.93138000
C	1.34396000	7.29740300	2.88974300
C	-0.58632400	6.88858400	0.93663300
C	1.56513800	6.34579500	1.89399000
C	0.59027700	6.12144600	0.91426100
C	-5.41593700	5.94605500	5.24768800
C	-5.19875600	7.07593600	4.20507500
C	-5.56954900	6.58912900	2.81461600
C	-6.89880000	6.32392600	2.45490500
C	-4.57804000	6.26904000	1.87817500
C	-7.23378000	5.74874000	1.22917200
C	-4.89102600	5.70121700	0.64340200
C	-6.22438000	5.41990300	0.30683900
C	-3.61213000	2.98631800	3.63360300
C	-2.26237500	2.36912600	4.04660800
C	-1.19431800	3.42539800	4.36885300
C	-1.78830100	1.36679600	2.98730600
C	-0.85788100	4.35836300	3.21070900
C	3.66167200	-8.12945900	6.28619000
C	4.31528400	-6.87268200	5.67700400
C	3.82647600	-6.52236800	4.29080300
C	4.65824400	-6.69099300	3.17744500
C	2.52818800	-6.03364000	4.09259500
C	4.21363400	-6.36274900	1.89501300
C	2.07759100	-5.71309500	2.81191500
C	2.92215100	-5.86881700	1.71005300
C	11.11304800	0.77823500	3.61940600
C	9.96202800	0.38267400	2.68052600
C	8.55340500	0.85350000	3.08989100
C	8.42756700	2.38090200	3.05909000
C	7.48942600	0.22298200	2.18014000
C	8.24258200	6.38126900	2.70862000
C	7.37448900	6.63978200	3.93511100
C	6.97243000	5.40794400	4.69717000
C	7.76395200	4.65739400	5.53058500
C	5.67522900	4.77289900	4.69007000
C	5.75360900	3.63573800	5.54235100
C	4.44911400	5.06424100	4.06449000
C	4.65709400	2.79930700	5.77726400
C	3.35588200	4.24187600	4.30002400
C	3.46070400	3.11965200	5.14861700
C	8.39504500	-5.55020500	-3.63238300
C	6.85686400	-5.58158400	-3.56836700
C	6.23189800	-4.55398600	-2.64883700
C	5.67381600	-3.38160400	-3.17292700
C	6.17935100	-4.72911200	-1.25829000
C	5.06732000	-2.42960600	-2.35853500
C	5.55531600	-3.79829100	-0.42788900
C	4.97461800	-2.64783300	-0.97887800
C	9.23684600	-4.92297000	2.92172200

C	7.83826400	-4.31667400	2.73515200
C	7.15682100	-3.82000900	4.02382300
C	8.01610500	-2.79986000	4.78261100
C	5.77121300	-3.23874200	3.70924100
C	8.75860700	3.44810100	-2.36752300
C	7.82196700	4.64911200	-2.40176800
C	8.37511900	2.50781700	-1.18980600
C	6.89594200	2.43544300	-0.89605800
C	6.15994400	3.21302200	-0.02413100
C	4.82359300	1.78638300	-1.03224100
C	5.73092500	5.41045700	-3.44611700
C	4.63819000	4.53136100	-4.05734400
C	2.76463100	3.05217500	-3.59120100
C	1.48928700	2.95845400	-2.76247400
C	6.14709400	1.62569400	-7.41182300
C	5.71723800	0.72519600	-6.24487700
C	6.18636200	1.19732400	-4.88973700
C	2.05613000	-7.01613000	-5.06504200
C	1.23395000	-5.74765500	-4.83225200
C	2.43107900	-7.73866500	-3.75216300
C	3.37499200	-6.95329000	-2.81905300
C	2.72424600	-5.72989700	-2.15625600
C	3.95100800	-7.88441000	-1.74540400
C	-0.69726500	-4.87750800	-3.58207900
C	-1.47268000	-5.44011000	-2.38936800
C	-2.40775100	-4.92795900	-0.18099100
C	-1.66019300	-5.91657900	0.74156500
C	-1.07550400	-8.35277500	0.84504900
C	-3.07828500	-7.75975600	-6.37712300
C	-2.84226400	-6.25995300	-6.66555300
C	-3.96014800	-5.32179700	-6.32046300
C	-4.13872800	-4.60729700	-5.15463000
C	-5.71128500	-4.10106000	-6.60553600
C	1.85807000	2.15451300	1.03349800
C	2.44652500	1.57270600	2.18236700
C	2.08636500	0.29978000	2.60703500
C	0.89583900	1.37782300	0.34049300
C	0.60078000	0.07150200	0.73753800
C	1.15872800	-0.46994000	1.89928700
C	-13.15929700	4.98448000	-3.00420400
C	-11.66103000	4.69079600	-2.73503200
C	-11.29297000	3.91080200	-1.46095400
C	-9.76410800	3.69763900	-1.35202000
C	1.09930600	-2.77075700	-0.23244000
O	-2.59433500	-1.45146800	-6.71086700
O	-0.95778300	1.39338500	-5.09192600
O	-5.30579400	-1.52289200	-3.58983400
O	2.55334000	-1.11278100	-6.50997500
O	-0.55873800	7.14589900	-4.16279500
O	-0.46913700	6.26141500	-2.13785900
O	0.74921900	5.18884900	-0.05852800
O	-6.49819700	4.84893900	-0.89503000
O	4.34668100	-1.77674400	-0.14378700
O	7.81325800	5.51130600	-1.52215000
O	4.71534100	4.16219700	-5.22483700
O	0.39355900	3.62994200	-3.39524200
O	7.07510800	2.02985600	-4.73155200
O	5.59126700	0.56064800	-3.89243400
O	1.63277300	-4.62451200	-5.12480700
O	-1.90994000	-6.60393500	-2.40930900

O	-0.91492500	-5.53328100	1.63982500
O	2.27093600	3.34425600	0.60443600
O	-0.26977900	-0.68756500	-0.05251700
O	-9.33458700	2.51594600	-1.45449200
O	-9.06012200	4.74213700	-1.19148200
O	-0.58212100	-2.12452300	-1.78971500
S	1.77846700	-0.60838100	-1.96936300
H	-3.52393900	5.78330400	-2.75232800
H	-1.97708800	5.17891400	-3.27548300
H	-1.05410800	2.93202700	-2.20640800
H	-4.63332100	1.66211500	-0.26804900
H	-3.39921700	3.59296600	-4.88505800
H	-5.68638600	-1.05037200	-6.69967700
H	-2.75884400	-2.19293500	-4.39810000
H	-2.11784300	-1.58362000	-2.85831300
H	-5.03561800	-2.01532000	-1.12023000
H	-3.26430800	-1.97708600	-1.21276700
H	-3.00123700	0.72350300	-3.53752700
H	-4.52576400	0.44836300	-5.14730700
H	1.57411500	-2.17714100	-8.40858200
H	-0.58466900	-1.61976500	-6.44523500
H	2.15058100	6.73564500	-5.02539500
H	3.37267400	7.61324600	-4.13065800
H	1.63893900	8.02704900	-2.29167400
H	2.08135100	6.34998800	-2.55971000
H	0.75771000	8.88065300	-5.11441500
H	-0.19734800	10.08966100	3.54102800
H	0.90635100	9.20130300	4.56856900
H	2.10437300	7.44421300	3.65386400
H	-1.70197200	8.42399300	1.92600600
H	2.46723500	5.74395700	1.87652300
H	-1.33354700	6.72516100	0.16836700
H	0.15577700	5.84147700	-1.49270500
H	-2.15889400	9.04705200	4.59963700
H	-5.76997900	7.97268900	4.47699600
H	-4.14257300	7.36740900	4.20151500
H	-7.69750400	6.56412900	3.15408800
H	-3.53442700	6.46334700	2.11469400
H	-8.26756200	5.56391200	0.95560600
H	-4.09991800	5.48245800	-0.06565600
H	-7.51203600	4.73872800	-1.03927500
H	-6.44921200	5.92913200	5.60908000
H	-2.43847600	1.80585500	4.97554200
H	-1.59706100	1.86202400	2.02999400
H	-0.85564600	0.87504600	3.27766700
H	-2.54398000	0.59001900	2.82026900
H	-1.54514100	4.02152200	5.22326200
H	-0.27969300	2.91583900	4.70250400
H	-1.74983100	4.85828500	2.82324900
H	-0.16615100	5.13971500	3.52698500
H	-0.38936800	3.82499800	2.37954900
H	-3.58707400	3.37239700	2.60912400
H	4.14293600	-6.02506800	6.35371600
H	5.40152900	-7.01985700	5.64224300
H	5.66872600	-7.06799200	3.31897200
H	1.86014800	-5.90434900	4.94000100
H	4.87570600	-6.48235400	1.04340700
H	1.06390500	-5.36184700	2.66243900
H	2.57539100	-5.60343500	0.71779200
H	3.71928500	-8.96783900	5.58424000

H	10.16836100	0.77520300	1.67507500
H	9.95319600	-0.71074100	2.57921500
H	8.36995400	0.51389600	4.12213300
H	7.41890500	2.70021900	3.33332300
H	8.62762000	2.75826300	2.04973000
H	9.12392900	2.87289900	3.74391600
H	6.47955500	0.50915600	2.49022500
H	7.61090600	0.55607200	1.14434700
H	7.54621600	-0.87013400	2.18626800
H	12.06955400	0.49485000	3.16257600
H	6.46629200	7.17271900	3.62982900
H	7.90643000	7.32666200	4.60726900
H	8.80179900	4.79208600	5.80025700
H	7.41897800	2.84811800	6.60193300
H	4.35883400	5.91943000	3.40080500
H	2.41127600	4.44588800	3.80918200
H	4.73642000	1.93322300	6.42837800
H	2.59060500	2.48784000	5.29523000
H	7.74433100	5.72668700	1.98778100
H	6.45792900	-5.42764600	-4.57773800
H	6.54114400	-6.58896900	-3.26723000
H	6.61441700	-5.62310200	-0.81506400
H	5.70516500	-3.20432000	-4.24480400
H	5.47899600	-3.96008000	0.64118200
H	4.68368700	-1.51348800	-2.78999000
H	3.71620200	-1.23890500	-0.66684300
H	8.75993200	-4.53833800	-3.83201300
H	7.89563100	-3.48653200	2.01853300
H	7.18072700	-5.06291700	2.27270900
H	7.01570800	-4.69501300	4.67775200
H	8.22958100	-1.92781300	4.15532900
H	7.49482200	-2.44187400	5.67771100
H	8.97290600	-3.22413300	5.10323700
H	5.84727300	-2.37709900	3.03707800
H	5.27115500	-2.90232900	4.62577000
H	5.12451500	-3.97995900	3.23358200
H	9.97627000	-4.16687900	3.20295700
H	8.74517100	1.50062000	-1.40419900
H	8.86830000	2.84173700	-0.27241400
H	6.45641200	4.02257200	0.62197300
H	3.91902100	1.26089700	-1.30287000
H	8.69858400	2.89301600	-3.30399400
H	5.51850400	5.71974500	-2.41879700
H	6.97501600	3.82472600	-4.07303800
H	1.25386800	1.90017000	-2.61258400
H	1.62033400	3.40655800	-1.77535900
H	0.07892200	3.05623300	-4.11715700
H	3.33826100	2.11798300	-3.52838300
H	3.77265800	4.29499300	-2.23436100
H	4.63691900	0.55358200	-6.21421900
H	6.15096900	-0.27873500	-6.35664900
H	7.10034300	2.10727000	-7.18324500
H	1.52598500	-8.01872800	-3.19566500
H	2.92037500	-8.68282300	-4.02517500
H	4.21019200	-6.58833400	-3.43069700
H	1.79722400	-6.01070000	-1.64079200
H	2.49853200	-4.94642900	-2.87922800
H	3.40650200	-5.29516200	-1.42244400
H	4.42974500	-8.76685900	-2.18614700
H	4.69734900	-7.35740600	-1.14428700

H	3.16517200	-8.23209400	-1.06377600
H	1.49132700	-7.69964600	-5.71143000
H	-1.40650600	-4.41020100	-4.27808500
H	-0.01096100	-4.09282500	-3.26058600
H	-0.26296100	-6.88430000	-3.94149500
H	-3.37797800	-5.34140400	-0.47606900
H	-2.57679000	-4.01614500	0.39095300
H	-1.19746800	-3.63679000	-1.44245600
H	-0.01274800	-8.10069800	0.74630200
H	-2.30689900	-7.31906500	-0.49104300
H	-2.60336100	-6.12527300	-7.72639900
H	-1.95216900	-5.95254600	-6.10579800
H	-3.59938900	-4.59804400	-4.22158900
H	-5.55950900	-3.08025300	-4.72414800
H	-6.58243300	-3.61538200	-7.02285200
H	-3.77197000	-8.22029200	-7.08508700
H	-11.11125300	5.63842100	-2.71241800
H	-11.26730900	4.13611700	-3.59728400
H	-11.62468800	4.47302300	-0.57865200
H	-11.78222400	2.93213600	-1.44833100
H	-13.75348700	4.06794600	-3.07862700
H	1.36920900	0.42794400	-4.71482300
H	1.53374400	-2.42445300	-3.59146700
H	2.90844200	-1.38845100	-3.94283400
H	1.38754300	4.39148900	0.22367000
H	3.19641000	2.14347200	2.71478400
H	0.45284700	1.76412000	-0.56676200
H	-2.06217200	-1.84476000	1.75684200
H	-5.17317900	3.72915500	-1.56222800
H	5.94120700	0.89535700	-2.98615200
H	1.92126200	-2.39202400	0.36997000
H	0.40384700	-3.33033100	0.40571700
H	1.51527300	-3.45856200	-0.97171100
H	3.98731800	3.15092400	0.33395800
H	-0.59176400	-1.42341700	-3.70674000
H	-13.60117900	5.62856600	-2.23701300
H	-13.17766300	5.50289400	-3.96808200
H	-5.25838100	5.02913200	4.66966600
H	-4.74477000	6.00593700	6.11120600
H	-4.33938400	2.16860700	3.67215900
H	-3.89410900	3.82255400	4.28193700
H	-1.14099100	7.81102500	5.35664900
H	-0.99357800	9.54761300	5.85889700
H	-4.68878300	4.77449600	-4.59875200
H	-3.12435500	5.24259200	-5.38000300
H	2.16349600	9.78254700	-4.51237400
H	2.26274500	8.87606200	-6.03685500
H	8.45535100	7.32914500	2.20469100
H	9.18273200	5.90081600	2.99668900
H	5.77963600	6.31349300	-4.06348300
H	2.40521800	3.13958700	-4.62194700
H	9.78447000	3.80275300	-2.22699800
H	5.39895600	2.40357600	-7.59490700
H	6.27631200	0.97555800	-8.28257200
H	-0.00376900	-1.50032800	-8.88415700
H	0.09883000	-3.17159900	-8.30776100
H	-5.00451900	0.28470900	-7.65587800
H	-4.72619200	-1.40499900	-8.17051600
H	-3.47172500	-7.87376500	-5.36346400
H	-2.09866500	-8.24632200	-6.42913400

H	2.98192200	-6.74224700	-5.58196400
H	8.74968400	-6.21137800	-4.42958400
H	8.82817000	-5.89200500	-2.68656900
H	-1.33618700	-9.11279000	0.10126600
H	-1.21784600	-8.73296300	1.86261800
H	2.60730800	-7.96027600	6.52181500
H	4.22492400	-8.40341700	7.18449400
H	9.57388400	-5.39130400	1.99085600
H	9.23964700	-5.69618500	3.69706500
H	11.06946900	0.24408600	4.57402700
H	11.16055100	1.86151500	3.77718200
H	-2.10897100	1.18189900	-0.63815600
H	-7.27012100	1.66164500	0.74864600
H	-8.89789200	-0.09648400	-0.55891700
H	-9.13057000	-1.57084900	1.43728600
H	-8.12692300	-5.33859200	4.47173900
H	-8.40860900	-5.85706300	2.80494800
H	-6.80625400	-5.25421800	3.27443300
H	-9.35296100	-3.55594100	3.33226900
H	-6.66392200	-2.64656300	3.51704000
H	-7.76504500	2.21368200	-1.40256900
H	-8.98503700	-3.16971600	8.88974900
H	-7.89795200	-4.52387500	8.73703100
H	-0.67644400	-2.49599300	3.99432300
H	-1.02011400	-4.18440400	3.61668600
H	-4.37031900	-3.60749200	4.79978500
H	0.90889500	-1.47761400	2.20945200
H	-4.54497600	-1.51714400	1.18104100
H	-7.82759100	-3.07247700	5.55273700
H	0.13139300	-2.22035200	6.94991500
H	1.62357300	-2.05334000	9.18061200
H	4.29469200	-2.69849000	7.80698400
H	4.07069100	-1.60189900	9.18524900
H	3.78012000	-3.34590700	9.37187800
H	-9.96043200	-5.18225700	10.03619100
H	-9.57491800	1.90331500	3.52448300
H	-8.74100500	3.06247900	2.50320800
H	-7.82069400	1.83151300	3.43135100
H	-10.97953600	-4.73613400	8.64945700
H	-9.87897100	-6.10407000	8.51982600
H	-8.73512200	-4.19119800	1.03631900
H	-7.05863000	-3.71209300	1.30316700
H	-5.90157400	0.05759900	-1.06486700
H	-7.19851200	0.03616200	-2.24304600
H	2.31064000	-3.83464300	6.78788000
H	-2.35348300	-4.38645100	5.73959400
H	1.58910600	-4.35725100	8.29905300
H	-1.98391800	-2.70383200	6.13584500
H	2.59614200	-1.25318100	6.66536900
H	2.55640900	-0.11535400	3.49566600

TS2 (+7.1)

C	-8.74393500	1.90924800	2.99285200
C	-8.85495200	0.78694000	1.98351600
O	-9.71168300	-0.09662400	2.10903800
N	-7.87780600	0.71937800	1.04854500
C	-7.95810100	-0.19487500	-0.08628000
C	-7.59415400	-1.65213400	0.23875700
O	-7.03105600	-2.35809700	-0.61485300
C	-6.98750500	0.33971100	-1.17546300
O	-6.91934300	1.74863000	-1.16919000
N	-7.90097700	-2.14170900	1.45168500
C	-7.51649100	-3.50466300	1.78377500
C	-7.65011300	-3.82319800	3.27130100
C	-7.14644600	-5.23834900	3.55397300
O	-6.96304000	-2.86594700	4.08691100
C	-9.78892200	-5.17503400	9.18073600
C	-8.81547200	-4.09574900	8.68571200
C	-8.50910100	-4.13778600	7.19069600
O	-9.00402500	-4.93025000	6.41098900
O	-7.62573900	-3.18836800	6.85330100
C	-1.07146600	-4.02427600	5.17495600
C	0.11158200	-4.34308600	6.08551200
O	0.58262300	-5.47397500	6.18784800
C	-0.78043400	-3.13871000	3.94007900
C	-2.06831600	-2.78383800	3.25114400
C	-3.36876800	-3.07212500	3.60780100
N	-2.18038000	-2.02343200	2.09666000
C	-3.51368700	-1.87943900	1.83132800
N	-4.26538700	-2.50524100	2.72550100
N	0.57215100	-3.30571300	6.84469100
C	1.79216600	-3.40262400	7.62815900
C	2.01220500	-2.08748100	8.37957400
C	3.36446300	-2.05483600	9.08353400
O	1.84740700	-0.96957200	7.50206600
N	-4.33545500	3.16482200	-1.54112800
N	-2.67601300	1.89575200	-0.97645400
N	-3.74241400	0.04359800	-5.33255300
N	-4.50808700	-2.20314400	-1.71296400
N	-0.50312800	-0.71774000	-4.33082300
N	0.21820700	-1.74087800	-6.78196400
N	7.02364700	3.77770200	5.90602300
N	5.94334500	1.64812800	-1.61017100
N	4.76718000	2.91444700	-0.22578000
N	6.78373200	4.71814800	-3.58715700
N	3.46003300	4.20730400	-3.31137400
N	0.02712900	-6.01468000	-4.11947400
N	-1.65665300	-4.63068200	-1.27771900
N	-1.82320800	-7.24269800	0.58513100
N	-5.04927800	-5.20843100	-7.10259600
N	-5.37660400	-3.97407500	-5.27982300
C	-3.78989100	4.53148300	-4.56955800
C	-3.19416700	4.81645900	-3.17461200
C	-3.22386200	3.65149400	-2.22746100
C	-2.18150100	2.83897300	-1.86045400
C	-3.98679700	2.11722200	-0.79006000
C	-4.97062100	-0.88875500	-7.19685200
C	-3.70606700	-0.87050800	-6.35063300
C	-2.86757600	-0.06925900	-4.16403900
C	-1.40209300	0.27213700	-4.53864200
C	-3.02759400	-1.41981200	-3.45000300
C	-4.43345800	-1.70060000	-2.96107400
C	0.89778900	-0.61878400	-4.68066900
C	1.23851500	-1.18645900	-6.08718200
C	1.75588200	-1.33999200	-3.62932900
C	0.39470200	-1.77178600	-1.08591200
C	0.37748600	-2.23224000	-8.14424600
C	1.57746800	8.85304300	-5.12086500
C	2.05414300	7.56900000	-4.41935000

C	1.36388400	7.21405700	-3.09738700
C	-0.10703400	6.84598100	-3.23684000
C	-1.27237700	8.89101900	4.98125800
C	-0.15757800	9.15293400	3.93707600
C	0.03832600	8.09791600	2.87036400
C	-0.93844400	7.84153900	1.89782700
C	1.23017600	7.36464000	2.79717200
C	-0.74554600	6.88403800	0.90680300
C	1.44731000	6.40951800	1.80426600
C	0.45103200	6.14907400	0.85512900
C	-5.46251100	5.91249700	5.29882500
C	-5.27485100	7.03491700	4.24208100
C	-5.65475900	6.52894900	2.86114100
C	-6.98542700	6.25107400	2.51729200
C	-4.66956300	6.20252200	1.92012100
C	-7.32805100	5.65827200	1.30215900
C	-4.99022600	5.61698900	0.69563800
C	-6.32488400	5.32416400	0.37498600
C	-3.63510400	2.96756900	3.68423200
C	-2.27415400	2.38781000	4.11643500
C	-1.23048500	3.47332600	4.42196300
C	-1.77418900	1.37014100	3.08783600
C	-0.86939200	4.35958000	3.23427500
C	3.87049200	-7.99584000	6.32492900
C	4.49489500	-6.74716900	5.67098100
C	4.07988100	-6.52677100	4.23469100
C	5.00998800	-6.65003700	3.19608200
C	2.75267400	-6.20926400	3.91558800
C	4.63080600	-6.43801200	1.86922200
C	2.36749600	-6.00390000	2.59062100
C	3.31023400	-6.10844000	1.56527200
C	11.12347000	1.00936000	3.44706200
C	9.96337400	0.58654600	2.53119700
C	8.55496200	1.04623300	2.95418900
C	8.41175300	2.57181300	2.90858200
C	7.48607500	0.39433000	2.06540700
C	8.14295900	6.55341300	2.52897400
C	7.29089900	6.80958400	3.76713300
C	6.91659400	5.57723500	4.54178400
C	7.72555900	4.85144200	5.38033300
C	5.63413300	4.91288300	4.53982900
C	5.73875000	3.78441600	5.40007800
C	4.40148300	5.17123700	3.91198700
C	4.66169500	2.92414200	5.63992400
C	3.32789200	4.32529500	4.15270900
C	3.45871600	3.21127100	5.00846100
C	8.39517500	-5.43741500	-3.69584200
C	6.85885700	-5.48305800	-3.60972100
C	6.23542600	-4.46737300	-2.67490500
C	5.64029400	-3.30478400	-3.17985100
C	6.21515900	-4.65124800	-1.28451000
C	5.03114400	-2.36952900	-2.34632200
C	5.59087100	-3.73892500	-0.43525500
C	4.97806500	-2.59598600	-0.96633300
C	9.33393200	-4.72964500	2.83683000
C	7.92329800	-4.15051000	2.65962800
C	7.24585400	-3.64755200	3.94707500
C	8.08682000	-2.58871300	4.67221200
C	5.83983900	-3.11384500	3.63802900
C	8.62786200	3.57843300	-2.52610300
C	7.67185300	4.76464000	-2.55817900
C	8.27764000	2.64347300	-1.33279300
C	6.80482900	2.55620300	-1.01556600
C	6.07436700	3.34603400	-0.14965400
C	4.73693100	1.89097400	-1.10886500
C	5.54736100	5.47785900	-3.57333700
C	4.46449700	4.56794700	-4.15606100
C	2.61987900	3.06830300	-3.64585100
C	1.36798200	2.96539400	-2.78538500
C	5.96297200	1.66071500	-7.50794900
C	5.56360600	0.76596400	-6.32463600
C	6.04633800	1.25762500	-4.98079000
C	2.05963800	-7.02505300	-5.00859100
C	1.20402500	-5.77799100	-4.77326900
C	2.47604400	-7.72713300	-3.69774600
C	3.43356600	-6.91550000	-2.80380700
C	2.78925900	-5.67570300	-2.16652300
C	4.03421700	-7.81779800	-1.71947600
C	-0.72970400	-4.94734300	-3.49806500
C	-1.47415500	-5.50772900	-2.28267300
C	-2.36914800	-4.97199200	-0.05087600
C	-1.55934900	-5.93498900	0.84262700
C	-0.95098600	-8.35444200	0.96579100
C	-3.08217500	-7.86883700	-6.22774000
C	-2.87994200	-6.36554800	-6.52700200
C	-4.02551800	-5.45233400	-6.20323100
C	-4.21874500	-4.69310000	-5.06781500
C	-5.82790700	-4.31560100	-6.51932000
C	1.84060700	2.24457800	0.97528800
C	2.54078900	1.67629500	2.06525500
C	2.20900900	0.41329000	2.54156400
C	0.81425600	1.47062300	0.38398100
C	0.52369500	0.16705900	0.82378400
C	1.20519700	-0.34853600	1.94203300
C	-13.32298300	4.73628400	-2.81332600
C	-11.81536300	4.47359100	-2.57065600
C	-11.40540100	3.73469400	-1.28545200
C	-9.87175200	3.54644600	-1.22006500
C	1.10429900	-2.75586700	-0.17871100
O	-2.73700800	-1.59562100	-6.56669900
O	-1.12224500	1.38003300	-4.99908000
O	-5.44016200	-1.52156500	-3.66653100
O	2.39843500	-1.10069900	-6.49103400
O	-0.78733800	7.09936600	-4.21292300
O	-0.66502000	6.26154400	-2.16937400
O	0.61095000	5.21676400	-0.11633100
O	-6.60616000	4.73761700	-0.81801900
O	4.36008400	-1.74288000	-0.10399300
O	7.66570400	5.63767000	-1.68955300
O	4.53997500	4.17222800	-5.31538600
O	0.25997600	3.64730900	-3.38203000
O	6.93178600	2.09638300	-4.84344200
O	5.46731100	0.62987000	-3.96644800
O	1.55660400	-4.64637900	-5.09257900
O	-1.87326300	-6.68282200	-2.27022400
O	-0.75623600	-5.53050300	1.68056000
O	2.21340200	3.43835800	0.50288300
O	-0.38338600	-0.60467000	0.15077700
O	-9.42661300	2.37417000	-1.36059500
O	-9.18014400	4.59865400	-1.06136600

O	-0.55403900	-2.18439200	-1.80573100
S	1.65107400	-0.60682800	-1.94111100
H	-3.69466100	5.67458500	-2.71316200
H	-2.14892000	5.09996800	-3.26668900
H	-1.15150900	2.89995100	-2.17600300
H	-4.65659800	1.56999300	-0.15130500
H	-3.57779700	3.49227200	-4.85137500
H	-5.81343200	-1.20778800	-6.57759100
H	-2.76704500	-2.21202800	-4.16029100
H	-2.32871900	-1.47383900	-2.61496200
H	-5.43195500	-2.38257100	-1.30258100
H	-3.66807100	-2.37143200	-1.18225300
H	-3.17232000	0.73892000	-3.49327700
H	-4.67006600	0.36824700	-5.09752800
H	1.44219300	-2.22405400	-8.37871800
H	-0.72512700	-1.69033000	-6.40968400
H	1.90985600	6.72491800	-5.10229500
H	3.13435500	7.62777200	-4.23537100
H	1.41960700	8.04328400	-2.37775100
H	1.87950400	6.36892500	-2.62872500
H	0.48868000	8.84857900	-5.19441100
H	-0.34694400	10.12680900	3.46429400
H	0.79016200	9.26428600	4.47729200
H	2.00856400	7.53940600	3.53692100
H	-1.87227100	8.39937700	1.91433100
H	2.36580300	5.83444300	1.76322300
H	-1.51055700	6.69377100	0.16248400
H	-0.02649800	5.85284300	-1.52788400
H	-2.27101300	9.06126200	4.56810700
H	-5.85590400	7.92606400	4.51172800
H	-4.22310100	7.34144000	4.22335300
H	-7.77895300	6.49517500	3.22106500
H	-3.62503700	6.40472500	2.14525800
H	-8.36300500	5.46289800	1.04071700
H	-4.20453200	5.39222500	-0.01768700
H	-7.61827900	4.61661800	-0.94560100
H	-6.49000000	5.88484900	5.67567800
H	-2.44641800	1.84586300	5.05884100
H	-1.58615100	1.84318400	2.11900000
H	-0.83291700	0.90514500	3.39442100
H	-2.51053600	0.57365200	2.93784700
H	-1.61306400	4.10116400	5.23956300
H	-0.32012600	2.99127900	4.80447100
H	-1.75457300	4.83108500	2.79839400
H	-0.19650400	5.16335100	3.53597500
H	-0.37077400	3.79497300	2.44208700
H	-3.61804300	3.33950600	2.65473200
H	4.23196200	-5.86555800	6.27012500
H	5.58751800	-6.83208700	5.71128900
H	6.04288100	-6.89760400	3.43056800
H	2.01949800	-6.11585400	4.71005200
H	5.36646300	-6.52224600	1.07511800
H	1.33274300	-5.77499000	2.35399600
H	3.01640900	-5.92977500	0.53853300
H	3.93006500	-8.84938400	5.64079000
H	10.15025100	0.96786200	1.51761600
H	9.96651500	-0.50817800	2.44470900
H	8.38835100	0.71601600	3.99220200
H	7.40146100	2.88262900	3.18623100
H	8.60151300	2.94084300	1.89407200

H	9.10683000	3.07867600	3.58381400
H	6.47722700	0.67188900	2.38642000
H	7.58968000	0.71865500	1.02488100
H	7.55556400	-0.69794800	2.08110500
H	12.07675100	0.73712300	2.97691700
H	6.37138300	7.32904600	3.47267200
H	7.82479100	7.50707100	4.42668900
H	8.76007300	5.01213000	5.64869900
H	7.42155700	3.04321900	6.46608200
H	4.29194200	6.01914500	3.24189300
H	2.37998600	4.49999400	3.65742300
H	4.76290700	2.06412500	6.29618900
H	2.60470500	2.55765500	5.15235300
H	7.64379700	5.88294100	1.82356100
H	6.44410200	-5.32853300	-4.61258900
H	6.55715600	-6.49444500	-3.30849000
H	6.67639900	-5.53909000	-0.85615200
H	5.64328100	-3.12108600	-4.25104200
H	5.53923600	-3.90993300	0.63346100
H	4.61592900	-1.46091800	-2.76594700
H	3.75408500	-1.16544600	-0.60354800
H	8.74536900	-4.42328700	-3.91073000
H	7.95811000	-3.32960200	1.93084300
H	7.27503500	-4.91470800	2.21493700
H	7.13799200	-4.51244500	4.62040800
H	8.26708000	-1.72501500	4.02368000
H	7.56909800	-2.22628400	5.56747500
H	9.05958200	-2.98014200	4.98684600
H	5.87961100	-2.27168500	2.93838500
H	5.34363900	-2.76424700	4.55122600
H	5.20885700	-3.88857700	3.19518000
H	10.06427800	-3.95795200	3.09893900
H	8.65899400	1.63970800	-1.54314600
H	8.78003500	2.99496600	-0.42703300
H	6.37409500	4.17130200	0.47458200
H	3.83048900	1.35678100	-1.35242300
H	8.56279900	3.01285400	-3.45594200
H	5.34617800	5.79349100	-2.54575000
H	6.80918300	3.90878000	-4.20534700
H	1.13021500	1.90746000	-2.63733600
H	1.52717300	3.39979300	-1.79650000
H	-0.07488300	3.08260700	-4.10107700
H	3.21439000	2.14675600	-3.58670100
H	3.60966400	4.36308900	-2.32231400
H	4.48605000	0.58215600	-6.27574100
H	6.00884600	-0.23328000	-6.43348000
H	6.91148700	2.15981900	-7.29832300
H	1.59041300	-8.01125000	-3.11293500
H	2.97073400	-8.66834900	-3.97076000
H	4.25480900	-6.56393900	-3.44131700
H	1.87984400	-5.94626300	-1.61531500
H	2.53446000	-4.92164600	-2.91039100
H	3.48802100	-5.20660500	-1.47086400
H	4.51756200	-8.70220100	-2.15100900
H	4.78129400	-7.27251400	-1.13667200
H	3.26250300	-8.16314400	-1.02122800
H	1.50025900	-7.72789600	-5.63889800
H	-1.46217400	-4.51471500	-4.19292600
H	-0.05988100	-4.13809200	-3.20413900
H	-0.23861200	-6.94707600	-3.83172700

H	-3.34302700	-5.40752700	-0.29736000
H	-2.52503100	-4.05513700	0.51616000
H	-1.22860800	-3.69145500	-1.36915200
H	0.10322000	-8.07876100	0.84377500
H	-2.27161400	-7.36750900	-0.31812600
H	-2.63403700	-6.23514400	-7.58697900
H	-2.00253200	-6.03161300	-5.96273100
H	-3.67035400	-4.61499200	-4.14307900
H	-5.71261200	-3.21982600	-4.68315300
H	-6.72270600	-3.88360900	-6.94554600
H	-3.77920700	-8.34530400	-6.92140300
H	-11.28169300	5.43067700	-2.58526300
H	-11.43150100	3.90238900	-3.42648600
H	-11.72059500	4.31742100	-0.41074300
H	-11.88026900	2.75010300	-1.23473000
H	-13.90176600	3.80868500	-2.86700300
H	1.17229300	0.43884300	-4.72044400
H	1.49995100	-2.40196500	-3.59687200
H	2.79733100	-1.26568200	-3.94154300
H	1.28970300	4.43771200	0.15279900
H	3.34096100	2.24808200	2.51661700
H	0.28712100	1.86281100	-0.47609000
H	-1.43296600	-1.60140600	1.52139200
H	-5.28082700	3.58375600	-1.48982200
H	5.82591500	0.98414700	-3.07071600
H	1.83700600	-2.28068200	0.46819700
H	0.38135400	-3.31898800	0.41869100
H	1.64151800	-3.45817200	-0.82511900
H	3.90029400	3.27825700	0.23165200
H	-0.73489700	-1.46370600	-3.68236100
H	-13.76178500	5.38139400	-2.04522200
H	-13.36607000	5.24450100	-3.78187400
H	-5.29880300	4.99257100	4.72733600
H	-4.77834700	5.99250800	6.15044500
H	-4.34751600	2.13807500	3.74291900
H	-3.92065000	3.80543100	4.32889000
H	-1.21889100	7.85065600	5.31837900
H	-1.09296700	9.59450500	5.80091300
H	-4.87748000	4.65373600	-4.54631000
H	-3.33437200	5.14036400	-5.35802400
H	1.88856900	9.77779400	-4.62313800
H	1.97823600	8.85774800	-6.14005200
H	8.33109100	7.49959000	2.01221500
H	9.09582200	6.09195000	2.80607800
H	5.57044700	6.37530300	-4.20027800
H	2.24209500	3.13916900	-4.67128700
H	9.64979200	3.95110200	-2.40507200
H	5.19869900	2.42390100	-7.68618600
H	6.08896200	1.00409000	-8.37426500
H	-0.15687100	-1.58385600	-8.84693300
H	-0.01620700	-3.24725400	-8.25596600
H	-5.16655000	0.12845700	-7.55327600
H	-4.86787400	-1.56141900	-8.05589000
H	-3.45682000	-7.97916700	-5.20649100
H	-2.09533100	-8.33916700	-6.29110900
H	2.97206800	-6.74064300	-5.54328200
H	8.74802100	-6.10047900	-4.49232100
H	8.84967700	-5.76219600	-2.75407500
H	-1.21073600	-9.12632100	0.23387000
H	-1.07000800	-8.72679500	1.98922800
H	2.81746400	-7.84222300	6.57634500
H	4.45313100	-8.25112800	7.21638200
H	9.66354100	-5.20153900	1.90514800
H	9.36268500	-5.49470500	3.61960500
H	11.10478200	0.48425900	4.40747600
H	11.15504600	2.09485300	3.59332800
H	-2.11832600	1.15332700	-0.55044100
H	-7.39030900	1.57869700	0.80705900
H	-8.98498500	-0.17979000	-0.47387900
H	-8.54452200	-1.61123800	2.03589600
H	-7.32689200	-5.49380700	4.59961000
H	-7.66712700	-5.97355100	2.93071400
H	-6.07379500	-5.30492600	3.34111900
H	-8.70220100	-3.75184100	3.57361600
H	-6.10161400	-2.66183600	3.65882600
H	-7.85686400	2.10314400	-1.32141400
H	-9.19851600	-3.09145400	8.90429500
H	-7.85567300	-4.15780700	9.21251000
H	-0.25762600	-2.21795700	4.23437600
H	-0.12562400	-3.68093000	3.25051900
H	-3.71571100	-3.65297300	4.44898900
H	0.97335900	-1.34514100	2.30073200
H	-3.87849900	-1.31135300	0.98752900
H	-7.42940100	-3.18395600	5.88100200
H	0.29569800	-2.35932100	6.61365100
H	1.20879100	-1.96614300	9.11551800
H	4.18012000	-2.15512600	8.35729600
H	3.49090400	-1.10767000	9.61370500
H	3.45569200	-2.87499100	9.80321100
H	-9.96007700	-5.06704200	10.25555900
H	-9.57750500	1.77890500	3.68642000
H	-8.78245300	2.94530800	2.63856400
H	-7.82599600	1.73960800	3.56333300
H	-10.74851800	-5.09928500	8.66295300
H	-9.39228800	-6.17541900	8.98824500
H	-8.11524000	-4.22555000	1.21088900
H	-6.47603800	-3.64234400	1.47495500
H	-5.98099000	-0.03741200	-0.96749600
H	-7.27638400	-0.07144100	-2.14702500
H	2.65411600	-3.59440900	6.96721200
H	-1.46774400	-4.98368100	4.83824900
H	1.76708400	-4.23914000	8.33469000
H	-1.83841100	-3.53365200	5.78616800
H	2.54803400	-1.00975700	6.83417500
H	2.75888900	0.00380400	3.38632400

Int2 (-12.7)

C	-8.83996800	1.81680100	2.77243100
C	-8.93205400	0.72819900	1.72717000
O	-9.80528700	-0.14580700	1.80572800
N	-7.95133600	0.70025600	0.78827000
C	-8.00423200	-0.26520500	-0.30880200
C	-7.50840200	-1.64860400	0.16335700
O	-6.42116900	-2.11949500	-0.20632800
C	-7.12446000	0.26856200	-1.46505800
O	-7.17000300	1.66885000	-1.55165100
N	-8.32718000	-2.32383200	0.99078800
C	-7.95729400	-3.63186000	1.50344500
C	-8.29413700	-3.86774900	2.98004500

C	-7.80715100	-5.25963200	3.39201800
O	-7.74818900	-2.85919800	3.82962300
C	-9.98770600	-5.25178200	8.96004900
C	-9.01738200	-4.19701600	8.41910900
C	-8.95657600	-4.12396200	6.89617800
O	-9.65837800	-4.79767400	6.16076300
O	-8.04707700	-3.24075400	6.48651200
C	-1.50249700	-2.80394200	5.61065100
C	-0.30016600	-3.53194700	6.20096500
O	0.06473000	-4.62561600	5.77818600
C	-1.48517400	-2.77299600	4.06829900
C	-2.85288800	-2.50627000	3.51072800
C	-4.09466600	-2.90160900	3.95351200
N	-3.10892600	-1.77598700	2.35715900
C	-4.45869500	-1.74654800	2.17291300
N	-5.08494400	-2.42732900	3.11860500
N	0.30753900	-2.92266500	7.26525600
C	1.61426700	-3.37197000	7.72178100
C	2.37259200	-2.21073000	8.38001600
C	3.83902200	-2.56873500	8.60268200
O	2.23017700	-1.00349700	7.63140300
N	-4.48094500	3.17390300	-1.68945400
N	-2.92690600	1.76457400	-0.99090800
N	-3.55196700	-0.16062200	-5.32492100
N	-3.93724800	-1.70732200	-1.41313200
N	-0.13140300	-0.59325700	-4.32432000
N	0.48727100	-1.76202700	-6.74570900
N	6.74481500	3.86846800	6.11113700
N	5.98914100	1.60857100	-1.35504000
N	4.73390100	2.89611500	-0.06197300
N	6.84367100	4.76256700	-3.38517100
N	3.50655000	4.21388500	-3.20908000
N	0.19708100	-5.90977600	-4.30280300
N	-1.44537400	-4.46363800	-1.50485900
N	-1.67451700	-7.18404200	0.70149400
N	-4.72266700	-5.11609200	-7.14854300
N	-5.07150900	-4.08253200	-5.20633600
C	-3.70322800	4.45637300	-4.66097400
C	-3.14400200	4.72340700	-3.25397800
C	-3.30456100	3.57223100	-2.30715300
C	-2.35739800	2.67489400	-1.86630500
C	-4.20681400	2.10522800	-0.90427100
C	-4.75789200	-0.98610400	-7.29616700
C	-3.50592600	-1.00091500	-6.39941600
C	-2.58134700	-0.19298300	-4.22933000
C	-1.16679700	0.20230600	-4.72393000
C	-2.62465900	-1.51513000	-3.43769200
C	-3.96306500	-1.73177200	-2.75756500
C	1.20925300	-0.38690700	-4.83053300
C	1.40411600	-0.87895000	-6.28214000
C	2.26922500	-1.18086800	-4.05525600
C	1.52791200	-2.08272600	-1.53413100
C	0.62747500	-2.28083700	-8.08888200
C	1.64380500	8.80319900	-5.06807800
C	2.08935800	7.52317400	-4.34276700
C	1.35768900	7.19380900	-3.03676000
C	-0.12359800	6.88056700	-3.20499200
C	-1.49474800	8.87986400	4.93661900
C	-0.36271000	9.13586900	3.91053500
C	-0.12844800	8.05960400	2.87084200

C	-1.08928200	7.74404900	1.89912500
C	1.09094700	7.37280700	2.81753000
C	-0.84845800	6.78224300	0.92252600
C	1.35541200	6.41615500	1.83678700
C	0.38092400	6.10456800	0.88311300
C	-5.66298600	5.86169400	5.15097100
C	-5.46132100	7.00583300	4.12604900
C	-5.85098300	6.52402200	2.74488000
C	-7.18703000	6.25725100	2.41842600
C	-4.87234400	6.16533200	1.80990100
C	-7.54195900	5.63196900	1.22554700
C	-5.20410000	5.54720000	0.60625400
C	-6.54350800	5.25092000	0.31103800
C	-3.76283500	2.92818000	3.60066800
C	-2.45761200	2.40816200	4.24648000
C	-1.39508400	3.50371600	4.41736600
C	-1.92056000	1.18004700	3.51359500
C	-0.82709700	4.05881900	3.11560500
C	3.77262500	-7.94984700	6.49703300
C	4.40129300	-6.68419900	5.87840000
C	3.91188700	-6.37380200	4.48394200
C	4.71856500	-6.63412800	3.36966800
C	2.62944500	-5.84839600	4.27944500
C	4.25455200	-6.37942300	2.07725400
C	2.16277200	-5.60110300	2.98955300
C	2.97168400	-5.86665300	1.88312500
C	11.01495400	1.11427300	3.78198200
C	9.87523800	0.68599800	2.84384600
C	8.47299800	1.21041500	3.20537200
C	8.39593400	2.73925100	3.11752100
C	7.40739800	0.57974200	2.29755600
C	8.00737600	6.62488900	2.75705500
C	7.13040800	6.87447400	3.95401800
C	6.73500500	5.62401700	4.68767500
C	7.37618300	5.05183600	5.75758300
C	5.64128200	4.74421300	4.35071300
C	5.67700300	3.65257000	5.26239600
C	4.64159400	4.76771200	3.36192900
C	4.75137800	2.60546100	5.21090400
C	3.71697600	3.73279100	3.30617300
C	3.77557800	2.66184400	4.22309400
C	8.54937600	-5.38804400	-3.40547100
C	7.01470200	-5.38072300	-3.31211700
C	6.51380200	-4.33425600	-2.34051500
C	6.41155900	-2.99637700	-2.74021400
C	6.19413600	-4.63902800	-1.01206800
C	6.02334800	-1.99666800	-1.85540100
C	5.78409500	-3.65443600	-0.11168100
C	5.71242900	-2.32254700	-0.53153600
C	9.29895200	-4.64454500	3.14760600
C	7.88425500	-4.08840800	2.93230200
C	7.17833900	-3.55826100	4.19385900
C	7.97876500	-2.44936400	4.88921500
C	5.76262600	-3.07383600	3.84811100
C	8.65763200	3.63604300	-2.26556900
C	7.68732700	4.80780600	-2.32056200
C	8.28752900	2.70033200	-1.08218400
C	6.80428700	2.58440700	-0.79395900
C	6.02298300	3.38406100	0.01897900
C	4.75933800	1.83432500	-0.89368400

C	5.59307200	5.49862800	-3.41017900
C	4.55150600	4.55180700	-4.00683400
C	2.69229800	3.06010400	-3.55330500
C	1.45241200	2.92251000	-2.68261100
C	6.15493600	1.66936800	-7.31473000
C	5.70471500	0.79139900	-6.14232400
C	6.14701000	1.30013300	-4.79509800
C	2.26832600	-7.04388600	-4.88663200
C	1.50002200	-5.74608200	-4.65869800
C	2.60834900	-7.82469900	-3.60398500
C	3.56914800	-7.13152100	-2.62218100
C	2.92412600	-5.95662900	-1.87790500
C	4.11505700	-8.15295000	-1.61636900
C	-0.59074700	-4.81704900	-3.76888100
C	-1.33042400	-5.33033100	-2.53720800
C	-2.00222000	-4.93872200	-0.25098200
C	-1.07905200	-6.00915900	0.35651300
C	-0.89419700	-8.37812500	1.00770100
C	-2.82922600	-7.94318100	-6.24428600
C	-2.65046800	-6.45558000	-6.61191600
C	-3.76649700	-5.52782000	-6.23821000
C	-3.97462000	-4.89583800	-5.03008400
C	-5.47445900	-4.24545100	-6.49691600
C	1.54033500	1.98961300	0.78581100
C	2.32523400	1.01487000	1.45693500
C	1.80135200	-0.24010300	1.74223700
C	0.22229300	1.62251800	0.40353700
C	-0.27750500	0.33893600	0.67163400
C	0.50963700	-0.59912100	1.35293000
C	-13.28303500	4.57459000	-3.17112400
C	-11.82224000	4.21116300	-2.85768000
C	-11.56567800	3.54409000	-1.49606300
C	-10.06663200	3.22088100	-1.36755800
C	2.13326200	-2.79574600	-0.36503000
O	-2.52397400	-1.70679600	-6.63441100
O	-1.00067900	1.20165300	-5.41466200
O	-5.00589900	-1.92192100	-3.40291800
O	2.42821900	-0.55805000	-6.88584600
O	-0.77427000	7.16169600	-4.19325400
O	-0.72068600	6.32115700	-2.14631600
O	0.59036600	5.18299800	-0.09620900
O	-6.83790800	4.61317400	-0.84741100
O	5.31463400	-1.37946400	0.37643600
O	7.62850200	5.66388000	-1.43727800
O	4.70550900	4.09263200	-5.13598700
O	0.41352800	3.81101400	-3.09038800
O	7.06810500	2.08825400	-4.62317800
O	5.47793700	0.73380300	-3.79188200
O	2.02633700	-4.63937400	-4.76997200
O	-1.80638000	-6.47026000	-2.51819300
O	0.11953100	-5.78109200	0.50324600
O	2.04828000	3.19221400	0.52793900
O	-1.54811100	-0.05500200	0.33190500
O	-9.65864000	2.20777000	-2.00012000
O	-9.35744800	4.03132900	-0.70044600
O	0.45567100	-2.40064900	-2.04187900
S	2.56761100	-0.83419600	-2.27617600
H	-3.59186500	5.63004200	-2.83153400
H	-2.07821700	4.93232400	-3.30873100
H	-1.30743800	2.67298800	-2.10944300
H	-4.95893200	1.62693100	-0.30051700
H	-3.48575100	3.41709000	-4.94148100
H	-5.62497500	-1.30684000	-6.71220400
H	-2.44793300	-2.34604500	-4.12574600
H	-1.84163600	-1.50607500	-2.67738200
H	-4.83044900	-1.81614900	-0.92615300
H	-3.11160300	-1.39489900	-0.92029300
H	-2.86251300	0.62421600	-3.55663400
H	-4.44421100	0.25155500	-5.09638000
H	1.68914900	-2.27062800	-8.33937000
H	-0.43843300	-1.78829000	-6.32936000
H	1.95192300	6.67416800	-5.02144000
H	3.16530300	7.57000400	-4.13073200
H	1.42767400	8.02241400	-2.31764100
H	1.82900100	6.33076600	-2.55585800
H	0.55805100	8.79768100	-5.17580500
H	-0.55526000	10.09526800	3.41028000
H	0.57281800	9.27312300	4.46589000
H	1.85908500	7.59285300	3.55559700
H	-2.04734200	8.25885800	1.90204800
H	2.30656200	5.89661000	1.81401500
H	-1.60184900	6.54510100	0.18039400
H	-0.10213900	5.84101200	-1.54344700
H	-2.48603400	9.03644400	4.50100700
H	-6.03356200	7.89527200	4.42049200
H	-4.40579500	7.30068300	4.11441000
H	-7.97129200	6.52689100	3.12365800
H	-3.82410800	6.34899000	2.03505600
H	-8.57799500	5.42278000	0.98122900
H	-4.42798400	5.27110700	-0.09854700
H	-7.80788500	4.31014900	-0.84903800
H	-6.69951600	5.82042000	5.50137500
H	-2.73458000	2.08820300	5.26367400
H	-1.79649400	1.37710500	2.44831300
H	-0.94493000	0.86383700	3.90098100
H	-2.61699500	0.34427700	3.61854100
H	-1.83087000	4.32626000	5.00303100
H	-0.57247700	3.09975300	5.02449000
H	-1.60841700	4.49104800	2.48478400
H	-0.10445500	4.84940700	3.31891600
H	-0.32077000	3.28605600	2.53169800
H	-3.64084500	3.26172500	2.56829300
H	4.20223500	-5.83148200	6.54083700
H	5.49075800	-6.80658500	5.85476000
H	5.71926100	-7.03406000	3.51708800
H	1.98053600	-5.63284100	5.12252700
H	4.89221300	-6.58554200	1.22253000
H	1.16204100	-5.21212000	2.83827700
H	2.58748000	-5.68708900	0.88845500
H	3.86013900	-8.79554300	5.80724800
H	10.10486300	1.02421100	1.82351300
H	9.84730900	-0.41069300	2.79700600
H	8.25430200	0.91526900	4.24385100
H	7.37711500	3.08843300	3.29084800
H	8.69483800	3.08060500	2.11931700
H	9.03977500	3.23668100	3.84850200
H	6.40427000	0.91034100	2.58535400
H	7.56064100	0.87728900	1.25485100
H	7.42588700	-0.51358000	2.33450200
H	11.98054100	0.84500900	3.33624500

H	6.22009900	7.40241700	3.64086100	H	-2.48856800	-6.36520800	-7.69130400
H	7.64295100	7.55309600	4.64858300	H	-1.73128800	-6.10106200	-6.13289000
H	8.24393200	5.39520000	6.30313500	H	-3.48781700	-4.98937300	-4.07373700
H	7.03696800	3.24724000	6.84566200	H	-5.32846400	-3.33927500	-4.55182200
H	4.59377800	5.58858500	2.65084700	H	-6.31725700	-3.70992500	-6.91046500
H	2.94939700	3.72577100	2.53943200	H	-3.49925800	-8.46102100	-6.93668000
H	4.79378700	1.77966900	5.91585900	H	-11.21009900	5.12058600	-2.91108200
H	3.04044500	1.86844000	4.14109200	H	-11.44157300	3.53967700	-3.63593100
H	7.53835100	5.94760800	2.03711900	H	-11.86790400	4.21054700	-0.68157100
H	6.58823200	-5.18728700	-4.30380000	H	-12.13952700	2.61310300	-1.42550100
H	6.66070400	-6.37265000	-3.00889200	H	-13.92220100	3.68618400	-3.22124900
H	6.26341500	-5.66929900	-0.67096100	H	1.44018600	0.67994900	-4.83234900
H	6.63379600	-2.72475900	-3.76923300	H	2.08328800	-2.25460000	-4.14999400
H	5.51323600	-3.90582600	0.90696500	H	3.23379800	-0.96775700	-4.51647800
H	5.94787400	-0.97251500	-2.19461000	H	1.17571000	4.37743300	0.19170800
H	5.40662100	-0.49770200	-0.01962600	H	3.33344900	1.26818500	1.76191300
H	8.92192200	-4.38008600	-3.61471800	H	-0.40098900	2.36549800	-0.07909000
H	7.91799500	-3.28844500	2.18084600	H	-2.44616800	-1.23822400	1.79202300
H	7.25404300	-4.87435100	2.49850000	H	-5.39662500	3.63270500	-1.68882200
H	7.08525100	-4.40023100	4.89812300	H	5.83789400	1.06783200	-2.90188700
H	8.13076800	-1.59984100	4.21531900	H	2.69488200	-2.11553600	0.27290700
H	7.44483900	-2.07787900	5.77118200	H	1.37549400	-3.34761900	0.19216800
H	8.96327500	-2.79610300	5.21905900	H	2.85460800	-3.50617700	-0.78202300
H	5.78996600	-2.26606100	3.10827200	H	3.83750300	3.20251800	0.37313800
H	5.24960900	-2.69338200	4.73998500	H	-0.25534200	-1.34708400	-3.66204500
H	5.15384600	-3.88448900	3.43880100	H	-13.70257500	5.25412700	-2.42056700
H	10.01171600	-3.86198200	3.42499500	H	-13.30411500	5.07841200	-4.14269500
H	8.68542400	1.70021700	-1.27770000	H	-5.47450700	4.94102900	4.58827800
H	8.76096300	3.06087400	-0.16442500	H	-5.00349900	5.95184100	6.02084300
H	6.27615300	4.24327100	0.61657700	H	-4.46850100	2.09196000	3.64319600
H	3.87642300	1.26588500	-1.14181700	H	-4.07431700	3.76586500	4.23343000
H	8.62681500	3.07044600	-3.19696200	H	-1.44055700	7.84152000	5.27977000
H	5.36091000	5.81791200	-2.39051700	H	-1.34504500	9.58849400	5.75789000
H	6.90678400	3.96692000	-4.01675900	H	-4.79216400	4.56802900	-4.66855300
H	1.11331400	1.87923300	-2.71548400	H	-3.23189700	5.06637300	-5.43932900
H	1.67122000	3.15063800	-1.63704200	H	1.93198600	9.73298800	-4.56596300
H	0.04214600	3.47231000	-3.91852400	H	2.07275100	8.80762500	-6.07573000
H	3.30669800	2.15210100	-3.48842700	H	8.20065700	7.57074200	2.24158800
H	3.55565800	4.45277700	-2.22765700	H	8.95662600	6.17397500	3.06255500
H	4.62181500	0.63495500	-6.14167800	H	5.62485400	6.39369300	-4.04014100
H	6.13996800	-0.21544900	-6.22044500	H	2.34247700	3.12300300	-4.58912400
H	7.09380800	2.17287400	-7.07542100	H	9.67115800	4.02010000	-2.11467900
H	1.67962200	-8.07852900	-3.07388900	H	5.38859800	2.42444000	-7.51758300
H	3.05057900	-8.78020300	-3.91432800	H	6.31142500	1.01064100	-8.17445900
H	4.41295900	-6.74100300	-3.20665600	H	0.10655400	-1.64060100	-8.80893600
H	2.01890700	-6.26961000	-1.34500400	H	0.24686800	-3.30007900	-8.20698100
H	2.66555500	-5.14398100	-2.55628900	H	-4.95353000	0.02767700	-7.66243900
H	3.62781800	-5.55413500	-1.14551500	H	-4.62452000	-1.66119400	-8.14911700
H	4.61072000	-8.99307600	-2.11655100	H	-3.23102500	-8.05296200	-5.23335300
H	4.83868300	-7.68674100	-0.94028100	H	-1.83645700	-8.40401900	-6.27813200
H	3.30600400	-8.55819600	-0.99670400	H	3.16616700	-6.75901000	-5.44512200
H	1.66976800	-7.69133200	-5.54060900	H	8.93020000	-6.05156000	-4.18874500
H	-1.32891600	-4.46228200	-4.49936800	H	8.98033300	-5.70505000	-2.44999300
H	0.07819500	-3.99117100	-3.53808400	H	-1.12607300	-9.15552500	0.27232600
H	-0.17837700	-6.83231000	-4.12390700	H	-1.03804700	-8.74730200	2.02912100
H	-3.01342300	-5.31754200	-0.42016800	H	2.71156500	-7.80558700	6.71843600
H	-2.06569400	-4.09449400	0.43993100	H	4.33271000	-8.19572000	7.40543000
H	-0.80654400	-3.66945000	-1.49261700	H	9.65890000	-5.11701700	2.22752300
H	0.15815300	-8.10322100	0.90659500	H	9.31336000	-5.40603500	3.93424300
H	-2.60877400	-7.32292100	0.34588000	H	10.97468900	0.59297400	4.74380700

H	11.03192000	2.20061400	3.92429300
H	-2.07094200	0.69027400	-0.14980200
H	-7.52522200	1.58197600	0.50709900
H	-9.04633400	-0.35962500	-0.63651900
H	-9.13081100	-1.80592400	1.35308300
H	-8.13401700	-5.48198800	4.40873400
H	-8.21046600	-6.03189000	2.72718200
H	-6.71333300	-5.30114200	3.34145600
H	-9.38010000	-3.81198000	3.12468100
H	-6.80626300	-2.67610000	3.55637100
H	-8.11852700	1.93881700	-1.78418700
H	-9.28191600	-3.19522900	8.77905600
H	-7.99662700	-4.37319900	8.77897700
H	-0.78323400	-2.00878200	3.71735600
H	-1.10518900	-3.73995400	3.71682000
H	-4.34151400	-3.49415900	4.82221800
H	0.11421700	-1.59065300	1.54591600
H	-4.93673000	-1.24147300	1.34933700
H	-8.01283200	-3.16077400	5.48425400
H	0.15231400	-1.92754600	7.37067500
H	1.90105500	-1.97946000	9.34304900
H	4.33502400	-2.76542600	7.64444400
H	4.36021600	-1.74561600	9.09765600
H	3.93817200	-3.46781700	9.22006700
H	-9.99377200	-5.24381300	10.05419800
H	-9.69313300	1.67458200	3.43883900
H	-8.87863200	2.85097800	2.41260000
H	-7.93660600	1.65854900	3.36883900
H	-11.00295000	-5.06318700	8.60190700
H	-9.70484800	-6.25150800	8.61967100
H	-8.45442800	-4.41743400	0.91679200
H	-6.88246900	-3.74503500	1.34647100
H	-6.08830200	-0.01862800	-1.28270300
H	-7.42921700	-0.23068400	-2.39402500
H	2.18092800	-3.74075500	6.85467300
H	-2.37922100	-3.36682300	5.94769300
H	1.57778500	-4.20576500	8.43106400
H	-1.60731400	-1.79050500	6.01061600
H	2.62791000	-1.14335300	6.75907800
H	2.42039900	-0.96308600	2.26825200

Int3 (-11.8)

C	-8.87375200	1.73525600	2.73998200
C	-8.92608600	0.63273600	1.71124600
O	-9.80255900	-0.23480300	1.74239400
N	-7.87349500	0.58210300	0.84598600
C	-7.90539200	-0.37258600	-0.25972200
C	-7.38042000	-1.73463100	0.23798500
O	-6.25190900	-2.15756800	-0.07136000
C	-7.04173200	0.19697400	-1.41164300
O	-7.07446400	1.60478900	-1.43941500
N	-8.19835100	-2.41804700	1.05460200
C	-7.85088100	-3.73471600	1.57480400
C	-8.17752800	-3.97608700	3.05092600
C	-7.83508400	-5.42348000	3.41158500
O	-7.46740500	-3.05980600	3.87797600
C	-9.97573500	-5.33362300	8.93538200
C	-9.04733700	-4.26758300	8.32388100
C	-8.82437200	-4.35513900	6.78618700
O	-9.44398900	-5.22606700	6.14696400
O	-7.99674300	-3.48788400	6.32648500
C	-1.40177900	-2.49910300	5.55170700
C	-0.19999000	-3.28907000	6.07109300
O	0.23714200	-4.26533500	5.47039700
C	-1.47067500	-2.47596700	4.01121500
C	-2.86784200	-2.25067300	3.51150200
C	-4.05857000	-2.70318100	4.02157000
N	-3.20379100	-1.51536400	2.37450000
C	-4.54074100	-1.52087600	2.23674000
N	-5.07897200	-2.24204600	3.21989600
N	0.30712500	-2.88195100	7.27777900
C	1.61231600	-3.35108200	7.72815700
C	2.41563800	-2.20434900	8.36098000
C	3.87494400	-2.60687500	8.55836700
O	2.29423400	-1.00057700	7.60764900
N	-4.52780600	3.22175900	-1.67647200
N	-3.00376800	1.87065600	-0.90434000
N	-3.54942500	-0.24187100	-5.30972200
N	-3.79765100	-1.61060700	-1.32313400
N	-0.12190200	-0.61865700	-4.33533100
N	0.51502500	-1.77082300	-6.74680000
N	6.68963800	3.89572600	6.07018100
N	5.97318900	1.65003500	-1.36132500
N	4.69296400	2.88806600	-0.04679200
N	6.79697400	4.80903800	-3.37901900
N	3.44423700	4.25315300	-3.20513700
N	0.24865200	-5.93174800	-4.30064300
N	-1.42887400	-4.49031400	-1.51594600
N	-1.62215300	-7.20407900	0.70678700
N	-4.65340000	-5.12262300	-7.10152100
N	-4.99493400	-4.15201500	-5.12537200
C	-3.73919100	4.40946100	-4.68254600
C	-3.16873100	4.70148100	-3.28660600
C	-3.34164600	3.58446200	-2.30294900
C	-2.39485500	2.71624500	-1.81632500
C	-4.29052600	2.20502100	-0.83450400
C	-4.73692600	-1.04638000	-7.31214500
C	-3.48612700	-1.05632800	-6.40654500
C	-2.57544000	-0.25692400	-4.21249900
C	-1.16381700	0.18554600	-4.68334800
C	-2.58638200	-1.57906700	-3.41880900
C	-3.88222300	-1.77350400	-2.65677900
C	1.22005600	-0.39191400	-4.83183900
C	1.42326600	-0.87884500	-6.28373100
C	2.28405100	-1.18077600	-4.05503000
C	1.52026700	-2.10471600	-1.54815700
C	0.66223000	-2.29363400	-8.08703700
C	1.56958000	8.80382700	-5.08092900
C	2.04654900	7.53826400	-4.34727800
C	1.32442400	7.19579900	-3.03786300
C	-0.13151200	6.78270300	-3.21492100
C	-1.59895300	8.86780100	4.91440100
C	-0.46544200	9.12709000	3.88854700
C	-0.20580400	8.04460600	2.85913200
C	-1.13995100	7.72285500	1.86327000
C	1.01765200	7.36242700	2.83427600
C	-0.86976400	6.76157400	0.89293700
C	1.31024200	6.40577300	1.86120800
C	0.36297500	6.08638500	0.88201700

C	-5.74037500	5.81246100	5.12135700
C	-5.55230100	6.94973800	4.08585400
C	-5.91942000	6.45998800	2.69873300
C	-7.24470000	6.16633800	2.34920800
C	-4.92479000	6.15455600	1.76172200
C	-7.57318300	5.57616500	1.12962900
C	-5.23135000	5.57681900	0.53123700
C	-6.56043200	5.26439800	0.20515100
C	-3.80927600	2.89379100	3.58128400
C	-2.51046900	2.44848300	4.30062600
C	-1.50169000	3.59893200	4.43920700
C	-1.88593600	1.19762400	3.69065900
C	-0.87420800	4.06387400	3.12971500
C	3.81552900	-7.91113400	6.51700600
C	4.44288500	-6.64878500	5.89002600
C	3.96569000	-6.35739000	4.48727400
C	4.77366900	-6.64461500	3.38058200
C	2.69128000	-5.82008800	4.26859900
C	4.31614000	-6.40596100	2.08278200
C	2.23024500	-5.58731300	2.97404900
C	3.03936500	-5.88253800	1.87524300
C	10.98368100	1.21375200	3.80875600
C	9.85518500	0.76864600	2.86505400
C	8.44711100	1.29425500	3.20065900
C	8.36931400	2.82140000	3.08730400
C	7.39627300	0.64346500	2.28959900
C	7.92949500	6.69536300	2.76624800
C	7.04676400	6.93889200	3.96021000
C	6.65587700	5.67662300	4.67833000
C	7.29778700	5.09894000	5.74479000
C	5.58402900	4.77895400	4.31434200
C	5.63613200	3.67134600	5.20630000
C	4.59239300	4.79603100	3.31678100
C	4.74033700	2.60052800	5.12336200
C	3.69516400	3.73842400	3.23152800
C	3.77390500	2.65005600	4.12601600
C	8.59800100	-5.32184000	-3.37553100
C	7.06291300	-5.33224600	-3.28650100
C	6.54593200	-4.29752400	-2.31092100
C	6.42659500	-2.95875300	-2.70274800
C	6.22428900	-4.61528100	-0.98596000
C	6.01764900	-1.97077200	-1.81380500
C	5.79251400	-3.64334700	-0.08227300
C	5.70170900	-2.31052800	-0.49477600
C	9.32161900	-4.56132000	3.17852500
C	7.90062300	-4.02330600	2.95758000
C	7.18382900	-3.50026900	4.21586800
C	7.96447400	-2.37544000	4.90795000
C	5.76091200	-3.04114800	3.86573100
C	8.61802000	3.70366700	-2.25034100
C	7.63413600	4.86257400	-2.30931400
C	8.25633000	2.76749700	-1.06587200
C	6.77121700	2.62925400	-0.78202700
C	5.97351900	3.39949700	0.04450600
C	4.73903600	1.84180500	-0.89740000
C	5.54361800	5.53768200	-3.40622000
C	4.50090300	4.58574900	-3.99566300
C	2.66541500	3.07283100	-3.55396000
C	1.41900300	2.85650300	-2.69908200
C	6.15154100	1.70756000	-7.30300600

C	5.70757800	0.82414900	-6.13268700
C	6.14548700	1.33161300	-4.78352200
C	2.33671600	-7.03659100	-4.87234300
C	1.54913400	-5.74885800	-4.65623200
C	2.66972100	-7.81999000	-3.58952800
C	3.63251400	-7.14015900	-2.60135400
C	3.01727800	-5.92270200	-1.90211500
C	4.11434600	-8.15795200	-1.55982000
C	-0.55301200	-4.84783100	-3.77182100
C	-1.28580000	-5.36482700	-2.53834200
C	-1.97503000	-4.97036600	-0.25945300
C	-1.03725600	-6.02891100	0.34740100
C	-0.83104700	-8.39017200	1.01473000
C	-2.74860900	-7.98406500	-6.24355400
C	-2.58897700	-6.49384600	-6.61259000
C	-3.70049400	-5.57284900	-6.20602200
C	-3.90525400	-4.98091200	-4.97666600
C	-5.39918900	-4.26945400	-6.42073800
C	1.38523000	1.93751800	0.77206100
C	2.17101500	0.92357600	1.37878700
C	1.61653500	-0.32485200	1.63978900
C	0.03783800	1.62471800	0.45494500
C	-0.52231100	0.34567700	0.69818900
C	0.30219600	-0.63692000	1.28754300
C	-13.32401800	4.44353800	-3.22096900
C	-11.84414300	4.18554400	-2.88700000
C	-11.55325600	3.48523300	-1.55080100
C	-10.03598900	3.28674900	-1.39325400
C	2.11302300	-2.82118400	-0.37624500
O	-2.48974500	-1.73546900	-6.65267300
O	-1.01492800	1.23743200	-5.30094400
O	-4.94673200	-2.05076900	-3.23106500
O	2.44347300	-0.54589400	-6.88614000
O	-0.79307400	7.03480500	-4.20427000
O	-0.69629300	6.16440100	-2.17230500
O	0.60545200	5.16446600	-0.08829800
O	-6.82808100	4.68005700	-0.98982900
O	5.28031100	-1.38042900	0.41672200
O	7.55962400	5.71665100	-1.42543500
O	4.66200800	4.11537700	-5.11899400
O	0.26927300	3.50726900	-3.23996400
O	7.05237000	2.13612200	-4.61014800
O	5.48994400	0.74746400	-3.78255300
O	2.05381800	-4.63361300	-4.78176100
O	-1.73591400	-6.51486700	-2.50946100
O	0.16055700	-5.78971200	0.48167500
O	1.92807300	3.13533300	0.52927300
O	-1.81788000	0.04977500	0.44456100
O	-9.58841700	2.11861500	-1.55473100
O	-9.35848800	4.33594200	-1.17093800
O	0.45727700	-2.42502300	-2.07420100
S	2.56629200	-0.85075000	-2.27127700
H	-3.59845000	5.62432600	-2.88137500
H	-2.09959000	4.88608700	-3.35437600
H	-1.34222200	2.68717900	-2.04756300
H	-5.04573100	1.75029000	-0.22025400
H	-3.50754100	3.37171800	-4.95652800
H	-5.60593700	-1.37351700	-6.73482700
H	-2.45728700	-2.41326400	-4.11265200
H	-1.76541400	-1.57877100	-2.70081100

H	-4.66611600	-1.74526300	-0.80130400
H	-2.96477300	-1.24095800	-0.86861400
H	-2.88317400	0.55332900	-3.54304100
H	-4.44472100	0.16540700	-5.08468700
H	1.72415500	-2.27501700	-8.33574500
H	-0.40960700	-1.80851800	-6.33012300
H	1.93515200	6.68097900	-5.02008300
H	3.12023700	7.61870000	-4.13404400
H	1.33206500	8.04633300	-2.34137000
H	1.84812300	6.38123800	-2.52720800
H	0.48379600	8.77804800	-5.18662900
H	-0.66850300	10.07805200	3.37677300
H	0.46506100	9.28384500	4.44704000
H	1.76777800	7.58643800	3.58949800
H	-2.09936300	8.23499600	1.84049700
H	2.26295300	5.88921300	1.86569500
H	-1.60320000	6.52376900	0.13093700
H	-0.06376600	5.76188700	-1.52250700
H	-2.59030500	9.01477700	4.47540100
H	-6.14214400	7.83178600	4.36686300
H	-4.50268000	7.26459700	4.07969200
H	-8.04359700	6.39250400	3.05287000
H	-3.88346500	6.35922000	1.99996000
H	-8.60311800	5.36010700	0.86519200
H	-4.44001600	5.36518100	-0.17883600
H	-7.82853800	4.46741900	-1.08976200
H	-6.77667300	5.76042300	5.47111400
H	-2.82121500	2.19092300	5.32562800
H	-1.76292800	1.29172000	2.61208100
H	-0.89763000	0.98803700	4.11643400
H	-2.52702400	0.33458200	3.88210300
H	-2.00180800	4.44939100	4.92541800
H	-0.70430900	3.28146200	5.12566200
H	-1.62947700	4.40372500	2.41549500
H	-0.20030800	4.90194400	3.30586000
H	-0.30102300	3.26743000	2.64849100
H	-3.66085500	3.22082400	2.55055600
H	4.23424400	-5.78866800	6.53979300
H	5.53301100	-6.76712600	5.87885700
H	5.76961000	-7.05253300	3.53793500
H	2.04593000	-5.57680100	5.10597100
H	4.95473500	-6.63242200	1.23401000
H	1.23670000	-5.18333000	2.81699300
H	2.66092100	-5.71530200	0.87583400
H	3.90982200	-8.76107300	5.83321500
H	10.09537000	1.09211200	1.84232600
H	9.83148500	-0.32868400	2.83419500
H	8.21486200	1.01577100	4.24074400
H	7.34614200	3.17132200	3.23022600
H	8.69363200	3.14848500	2.09239000
H	8.99407100	3.33042500	3.82678100
H	6.38926900	0.98672700	2.54713800
H	7.57297800	0.90874400	1.24192700
H	7.40942400	-0.44828900	2.35983600
H	11.95466700	0.95348200	3.36923200
H	6.13579800	7.46499400	3.64605400
H	7.55377900	7.61326300	4.66267300
H	8.15336000	5.45019700	6.30438400
H	6.98534700	3.26987000	6.79938600
H	4.52954600	5.63017500	2.62253500
H	2.92545800	3.72531900	2.46581000
H	4.79796100	1.76235100	5.81238000
H	3.06051800	1.83970600	4.01939000
H	7.46810300	6.01342600	2.04582100
H	6.63688800	-5.13952200	-4.27849700
H	6.72208700	-6.33062100	-2.98885000
H	6.30530600	-5.64680300	-0.65121800
H	6.65095900	-2.67668400	-3.72848200
H	5.51773600	-3.90590700	0.93241800
H	5.93015400	-0.94545100	-2.14702100
H	5.35402200	-0.49527300	0.02578300
H	8.96045500	-4.31032100	-3.58512700
H	7.92600600	-3.22434100	2.20466400
H	7.28221600	-4.81848000	2.52365000
H	7.10390100	-4.34105800	4.92317400
H	8.10167600	-1.52558600	4.23120100
H	7.42393200	-2.01057400	5.78863000
H	8.95492100	-2.70368300	5.23901200
H	5.77583900	-2.23574500	3.12296600
H	5.23954400	-2.66628100	4.75512500
H	5.16662900	-3.86332100	3.45816900
H	10.02456300	-3.77028500	3.45664100
H	8.66656500	1.77178700	-1.25879600
H	8.72384500	3.13372300	-0.14733300
H	6.20937800	4.24826000	0.66361300
H	3.86599600	1.26285600	-1.15623500
H	8.59603000	3.13565900	-3.18047600
H	5.31225700	5.86019000	-2.38715200
H	6.86784800	4.01295000	-4.00938600
H	1.24335900	1.77754800	-2.60650500
H	1.55466300	3.23031700	-1.68231100
H	-0.02002500	3.00772000	-4.02126700
H	3.32009100	2.19276400	-3.49838900
H	3.49516000	4.48268300	-2.22123600
H	4.62584400	0.66023400	-6.13226800
H	6.14964700	-0.17946900	-6.21366800
H	7.08513000	2.21980700	-7.06164600
H	1.73745800	-8.06697500	-3.06223700
H	3.10490500	-8.77884400	-3.89936800
H	4.50549000	-6.79721700	-3.17306300
H	2.08001300	-6.18322000	-1.39646700
H	2.82112800	-5.11359400	-2.60538200
H	3.71217600	-5.53806000	-1.15219800
H	4.57465000	-9.03509100	-2.02919400
H	4.85148400	-7.70797100	-0.88754500
H	3.27890400	-8.50498500	-0.94004500
H	1.74882900	-7.68903500	-5.53137900
H	-1.29685000	-4.50599800	-4.50265900
H	0.10564200	-4.01332400	-3.54365400
H	-0.10949400	-6.85874300	-4.10952400
H	-2.98259900	-5.36170300	-0.42285200
H	-2.04367600	-4.12354300	0.42770500
H	-0.80553200	-3.68419700	-1.50417900
H	0.21869700	-8.10537900	0.91515700
H	-2.55867300	-7.35290200	0.36120200
H	-2.45653000	-6.39728800	-7.69525000
H	-1.65968600	-6.13486800	-6.15650200
H	-3.42468400	-5.11615600	-4.02248800
H	-5.24574000	-3.42795400	-4.44662300
H	-6.24140200	-3.71911200	-6.81517000

H	-3.41194700	-8.51111300	-6.93540000
H	-11.29989600	5.13657200	-2.88918100
H	-11.40527100	3.58167900	-3.69223000
H	-11.92094300	4.10369600	-0.72316500
H	-12.04807000	2.51020000	-1.50772900
H	-13.89747900	3.51304000	-3.28986700
H	1.43804000	0.67703300	-4.82470000
H	2.11024100	-2.25535000	-4.16391600
H	3.24923100	-0.95207000	-4.50782200
H	1.14253000	4.30015800	0.22159700
H	3.19707100	1.13889500	1.65460600
H	-0.57568300	2.42356200	0.05600100
H	-2.57570300	-0.96035300	1.73115300
H	-5.44321200	3.69539200	-1.70435900
H	5.84267000	1.09019500	-2.89039600
H	2.68931100	-2.14806900	0.25546300
H	1.34808300	-3.35684900	0.18624700
H	2.81930900	-3.54735000	-0.79197000
H	3.79618800	3.17163500	0.39215800
H	-0.24150900	-1.40961700	-3.71683000
H	-13.80472000	5.09010300	-2.47870000
H	-13.34674900	4.94561100	-4.19340000
H	-5.54197400	4.89265100	4.56064600
H	-5.08433400	5.90990300	5.99300700
H	-4.50750000	2.05130800	3.62307900
H	-4.13018900	3.72962200	4.21179400
H	-1.53643700	7.83053500	5.25935800
H	-1.45809400	9.57905600	5.73496200
H	-4.82903400	4.51135600	-4.69352700
H	-3.27107000	5.02256700	-5.46050100
H	1.84786000	9.73698700	-4.57945900
H	2.00141400	8.81056600	-6.08733500
H	8.11576000	7.64211000	2.24985300
H	8.88187100	6.25351100	3.07526000
H	5.56916900	6.43200900	-4.03751200
H	2.31808700	3.13094500	-4.59089600
H	9.62650300	4.10004600	-2.09790200
H	5.37899000	2.45533600	-7.50930600
H	6.31646700	1.04891200	-8.16122300
H	0.13770900	-1.65926300	-8.80965100
H	0.29122300	-3.31647000	-8.20465100
H	-4.94057400	-0.03497500	-7.68064500
H	-4.59491400	-1.72155500	-8.16366400
H	-3.15234500	-8.09591500	-5.23364900
H	-1.75160700	-8.43599300	-6.27378900
H	3.23346900	-6.74460100	-5.42881900
H	8.98718500	-5.98306100	-4.15662900
H	9.02903900	-5.63344400	-2.41828500
H	-1.05375400	-9.17079200	0.27992000
H	-0.97458500	-8.75903500	2.03630700
H	2.75255900	-7.77618500	6.73505600
H	4.37513100	-8.15052600	7.42744400
H	9.68852600	-5.03196500	2.26025700
H	9.34058700	-5.32141200	3.96641700
H	10.94529200	0.69361600	4.77128800
H	10.99041700	2.30042300	3.94938700
H	-2.48495300	1.07987600	-0.28664300
H	-7.45689800	1.46941300	0.57038800
H	-8.94542700	-0.48647700	-0.58457100
H	-9.05254800	-1.94097100	1.34631200
H	-8.18153700	-5.62692200	4.42803500
H	-8.32975900	-6.12550600	2.72943400
H	-6.75122100	-5.57710300	3.33979200
H	-9.25589000	-3.82374300	3.21084200
H	-6.11858800	-2.50679400	3.41759500
H	-8.03944500	1.89620000	-1.55332200
H	-9.43230400	-3.26104700	8.53453700
H	-8.05768200	-4.29992800	8.79779700
H	-0.80835600	-1.70192700	3.61379800
H	-1.09017200	-3.43628800	3.64490200
H	-4.27535900	-3.30965000	4.88562700
H	-0.09999400	-1.63185400	1.45223100
H	-5.08893200	-1.05193500	1.43865800
H	-7.71535300	-3.26413700	4.88958500
H	0.07522200	-1.93895900	7.56166500
H	1.97278600	-1.95340200	9.33332700
H	4.34584300	-2.82341300	7.59184200
H	4.43001600	-1.79662900	9.03741500
H	3.95977100	-3.50449400	9.18014700
H	-10.09318100	-5.18432800	10.01548000
H	-9.72929400	1.58709100	3.40185400
H	-8.92064900	2.76853500	2.37835800
H	-7.97071100	1.58617600	3.33922500
H	-10.96164000	-5.29735000	8.46376700
H	-9.57796200	-6.33797400	8.76165700
H	-8.37627600	-4.50077300	0.98935800
H	-6.78246400	-3.87710000	1.39756900
H	-6.00347600	-0.10547700	-1.26804400
H	-7.37346900	-0.25495300	-2.35499400
H	2.14486900	-3.73167700	6.84657500
H	-2.28447800	-3.00391200	5.96027900
H	1.58129600	-4.18401300	8.43866600
H	-1.41827100	-1.47972900	5.95089300
H	2.63985700	-1.16805800	6.71831200
H	2.23178500	-1.08134400	2.12199200

Int4 (-13.2)

C	-8.82707000	1.84236300	2.84857200
C	-8.92290200	0.74404100	1.81638600
O	-9.84606500	-0.07461100	1.82845900
N	-7.86557700	0.64493500	0.96569200
C	-7.92042400	-0.26587400	-0.17525900
C	-7.51553600	-1.69596500	0.23796600
O	-6.51083100	-2.25409400	-0.24183300
C	-6.97980400	0.29148000	-1.26627900
O	-6.99364800	1.70168500	-1.29336500
N	-8.30380500	-2.30427500	1.13247500
C	-8.04711900	-3.66789500	1.58144700
C	-8.30500900	-3.91772200	3.06725500
C	-8.07279500	-5.39850700	3.37710400
O	-7.45970100	-3.08658400	3.84338500
C	-9.93656100	-5.21415300	9.05711400
C	-9.22679700	-4.04753500	8.35333800
C	-8.76206100	-4.33051900	6.89407700
O	-8.93478600	-5.48330800	6.44635700
O	-8.23834300	-3.32446200	6.30760000
C	-1.44054500	-2.85424600	5.55146300
C	-0.19634100	-3.52065000	6.12397700
O	0.26042400	-4.55214400	5.63880000

C	-1.37736900	-2.66594000	4.02220500
C	-2.73594100	-2.42505600	3.42351800
C	-3.96127300	-2.76443300	3.95253700
N	-2.93681300	-1.82235000	2.18158300
C	-4.26097900	-1.81282700	2.00025300
N	-4.91274300	-2.37357800	3.04272500
N	0.34587300	-2.94464600	7.24238900
C	1.65827700	-3.35337200	7.72477400
C	2.38012700	-2.17348900	8.38845900
C	3.84615400	-2.50677900	8.65218200
O	2.23942200	-0.97659000	7.62411400
N	-4.50623600	3.34940400	-1.58443100
N	-2.99601700	1.98013000	-0.86121800
N	-3.57022400	-0.12357900	-5.31779800
N	-4.10323600	-1.97356400	-1.51422700
N	-0.18002900	-0.63744000	-4.30053900
N	0.41675800	-1.76602200	-6.73616100
N	6.81793200	3.87207700	6.07134900
N	5.96435300	1.62644400	-1.41833500
N	4.73253700	2.89934000	-0.09078700
N	6.80585700	4.74688700	-3.43995500
N	3.44796600	4.23305000	-3.22563400
N	0.13111900	-5.93144500	-4.26727800
N	-1.50630900	-4.45476100	-1.49190400
N	-1.68255300	-7.16763600	0.74531800
N	-4.78341200	-5.11605600	-7.13790200
N	-5.20089400	-4.11397000	-5.19451500
C	-3.74488000	4.46190400	-4.62935100
C	-3.16354300	4.78682000	-3.24522100
C	-3.32892000	3.68946600	-2.24199600
C	-2.38159000	2.81003000	-1.78469200
C	-4.27949900	2.33188200	-0.75047200
C	-4.82811300	-0.98360800	-7.24685800
C	-3.55397100	-0.98948300	-6.37918900
C	-2.62769300	-0.21026400	-4.19571400
C	-1.19467200	0.21403600	-4.61151100
C	-2.70574000	-1.56023100	-3.45524200
C	-4.07815800	-1.78283500	-2.84262400
C	1.16622900	-0.43386400	-4.80131900
C	1.35076700	-0.90875300	-6.26173500
C	2.22776400	-1.24174300	-4.04199700
C	1.49661700	-2.14000100	-1.52074600
C	0.54905400	-2.28747600	-8.07946200
C	1.59637700	8.82507700	-5.09980500
C	2.07977200	7.55613900	-4.37611900
C	1.37256600	7.20851800	-3.06053400
C	-0.08327200	6.79300200	-3.22634500
C	-1.45481400	8.89826000	4.94315600
C	-0.32973600	9.15582700	3.90835500
C	-0.09268100	8.07748300	2.87111800
C	-1.03429500	7.78623200	1.87349300
C	1.11167700	7.36264300	2.84745000
C	-0.79121200	6.81840000	0.90276600
C	1.37743000	6.39747500	1.87554700
C	0.42023800	6.10883100	0.89826700
C	-5.62558400	5.88660500	5.19531600
C	-5.44014200	7.02274400	4.15811500
C	-5.82188900	6.54131600	2.77127000
C	-7.14738600	6.22806000	2.43806900
C	-4.83936200	6.27541900	1.81040200
C	-7.48614600	5.65630700	1.21255900
C	-5.15733400	5.71815900	0.57290800
C	-6.48510200	5.38467600	0.26338000
C	-3.74189600	2.94766300	3.63512600
C	-2.42861500	2.42554500	4.25906600
C	-1.40270700	3.54377000	4.49824300
C	-1.85054500	1.26904700	3.45080000
C	-0.89138700	4.22760800	3.23366600
C	3.80019600	-7.93649200	6.49088400
C	4.43359500	-6.68496700	5.84794600
C	3.96031900	-6.41667400	4.43884300
C	4.78526600	-6.68659700	3.34048800
C	2.67068900	-5.92213900	4.20788900
C	4.32970700	-6.47321200	2.03747900
C	2.21154800	-5.71780200	2.90780100
C	3.03723500	-5.99539300	1.81655200
C	11.03416700	1.11241100	3.70376500
C	9.88598200	0.68272500	2.77648400
C	8.48840400	1.21473300	3.14493000
C	8.41700900	2.74327800	3.04659500
C	7.41318400	0.58008600	2.25098100
C	8.02666800	6.62572700	2.69305200
C	7.14795000	6.88191500	3.91241800
C	6.77184900	5.63039000	4.65190500
C	7.43332600	5.06159300	5.71133100
C	5.68058600	4.74205500	4.32996700
C	5.73875800	3.64948000	5.23975000
C	4.66563600	4.75868700	3.35703400
C	4.82101700	2.59508600	5.20172100
C	3.74809500	3.71671100	3.31554000
C	3.82896500	2.64517900	4.23001400
C	8.50290300	-5.39847500	-3.45307100
C	6.96861400	-5.38581100	-3.34785000
C	6.47742600	-4.34364200	-2.36625800
C	6.39669700	-2.99929400	-2.74940400
C	6.13873400	-4.66154200	-1.04574100
C	6.00568300	-2.00745400	-1.85671900
C	5.72540100	-3.68498400	-0.13795300
C	5.66901000	-2.34818900	-0.54310100
C	9.30489000	-4.64495500	3.09265800
C	7.88736200	-4.09254800	2.88391600
C	7.18373400	-3.56806900	4.14879300
C	7.98086300	-2.45538300	4.84165200
C	5.76358500	-3.09231400	3.80946700
C	8.63154000	3.62552000	-2.33198900
C	7.66071200	4.79633500	-2.38358900
C	8.27108900	2.69426100	-1.14163300
C	6.79270900	2.58130900	-0.84293000
C	6.02617600	3.37185200	-0.00811100
C	4.74059200	1.85354200	-0.94382300
C	5.56247300	5.49253300	-3.45315400
C	4.49809500	4.55746400	-4.03186300
C	2.65712200	3.05804000	-3.56939700
C	1.42178000	2.84005800	-2.69740700
C	6.08817600	1.65585700	-7.35549900
C	5.64894100	0.77525100	-6.18094900
C	6.09761500	1.28392400	-4.83610900
C	2.20803200	-7.04756100	-4.88216600
C	1.41820600	-5.75585100	-4.67414300
C	2.49350600	-7.84534800	-3.59683600

C	3.46451600	-7.19789800	-2.59555600
C	2.91604200	-5.91276800	-1.96451900
C	3.83135000	-8.20855200	-1.50167700
C	-0.67082700	-4.83309300	-3.76432000
C	-1.42005100	-5.32480500	-2.52832200
C	-2.02779200	-4.93378600	-0.22122300
C	-1.08930300	-6.01338700	0.34318900
C	-0.91021200	-8.36726200	1.03903800
C	-2.90125300	-7.94173900	-6.19834100
C	-2.72346900	-6.45332500	-6.56407600
C	-3.84980100	-5.53099900	-6.20532100
C	-4.09812000	-4.91698100	-4.99490300
C	-5.56434800	-4.26371100	-6.49888600
C	1.58057100	1.98627600	0.79693400
C	2.38435200	1.01905300	1.45700800
C	1.87252100	-0.23063100	1.77960600
C	0.25553700	1.60687000	0.45581700
C	-0.22963300	0.33324400	0.77188100
C	0.56803600	-0.59832300	1.43866800
C	-13.31256000	4.59658200	-3.06465200
C	-11.82066100	4.34434800	-2.76297900
C	-11.48020400	3.62971500	-1.44570600
C	-9.95562800	3.44708000	-1.32078700
C	2.08094500	-2.84315900	-0.33574200
O	-2.57891600	-1.69930500	-6.62451900
O	-1.00711700	1.29743400	-5.16360400
O	-5.10794800	-1.77742900	-3.53708300
O	2.37874500	-0.59460500	-6.86105100
O	-0.75439600	7.03830500	-4.21000800
O	-0.63882800	6.17332900	-2.17721900
O	0.63272400	5.17844700	-0.07513300
O	-6.76271000	4.81610700	-0.93865300
O	5.25855000	-1.41274500	0.36913900
O	7.60958000	5.65672400	-1.50433400
O	4.63230400	4.09502600	-5.16130600
O	0.24748600	3.43662000	-3.24693200
O	7.01235500	2.08144500	-4.67160700
O	5.44235600	0.71138800	-3.82800400
O	1.91056900	-4.64148600	-4.84843400
O	-1.92518800	-6.45049500	-2.51108500
O	0.12221600	-5.80720100	0.40284200
O	2.07171900	3.18975500	0.52287000
O	-1.53350800	-0.00387700	0.43452600
O	-9.49943400	2.28006100	-1.45443200
O	-9.28128000	4.50854700	-1.13928100
O	0.42457700	-2.44697200	-2.03558700
S	2.55564100	-0.91120200	-2.26689800
H	-3.59386900	5.71566200	-2.85518000
H	-2.09523100	4.97006100	-3.32522900
H	-1.33710900	2.75457500	-2.05214100
H	-5.02459800	1.88378800	-0.11977000
H	-3.51714600	3.42038700	-4.88871500
H	-5.67932300	-1.30455300	-6.64100200
H	-2.51373200	-2.36736800	-4.16779200
H	-1.94660200	-1.59281700	-2.67054700
H	-5.01373400	-2.10449500	-1.05457200
H	-3.25693100	-1.95192100	-0.96615900
H	-2.93480600	0.58388600	-3.50680400
H	-4.48102100	0.22373100	-5.05345900
H	1.60912900	-2.27936300	-8.33576700
H	-0.50685900	-1.79044300	-6.31627400
H	1.96029700	6.70162600	-5.05098400
H	3.15571500	7.63507600	-4.17462300
H	1.38599400	8.05589100	-2.36036900
H	1.90340900	6.39165600	-2.56023900
H	0.50935300	8.80633500	-5.19215000
H	-0.53068700	10.11135100	3.40469900
H	0.60774300	9.30101800	4.45803100
H	1.86624800	7.56451600	3.60418300
H	-1.97791000	8.32661300	1.85073100
H	2.31464700	5.85322100	1.88007800
H	-1.52822400	6.60220900	0.13771800
H	-0.00063700	5.78681100	-1.52663300
H	-2.44908500	9.05564100	4.51459800
H	-6.02140400	7.90847800	4.44542500
H	-4.38882700	7.33152500	4.14233300
H	-7.93820600	6.42348500	3.15949000
H	-3.79848900	6.49835000	2.03333900
H	-8.51646800	5.42626800	0.96198800
H	-4.37437400	5.54428500	-0.15671300
H	-7.77353500	4.61391600	-1.04393300
H	-6.65779300	5.84350500	5.55786900
H	-2.70079300	2.03664700	5.25237800
H	-1.66203100	1.57744600	2.42254900
H	-0.89649500	0.91832400	3.86049600
H	-2.53988900	0.42151400	3.42098000
H	-1.85510900	4.29903700	5.15714100
H	-0.55042900	3.12664600	5.05237900
H	-1.70675900	4.66818700	2.65305700
H	-0.20387000	5.03619100	3.48318200
H	-0.35805000	3.53135500	2.57979300
H	-3.63145700	3.28849400	2.60260300
H	4.22433100	-5.81424400	6.48308200
H	5.52357000	-6.80564800	5.84110100
H	5.79250600	-7.06150300	3.50797300
H	2.01491100	-5.68947500	5.03983100
H	4.98314600	-6.68482000	1.19614600
H	1.20546100	-5.35076500	2.73812000
H	2.65913200	-5.84911800	0.81340400
H	3.87741300	-8.79044500	5.80973900
H	10.10927200	1.01354100	1.75227600
H	9.85346400	-0.41405600	2.73711900
H	8.27644100	0.92811100	4.18703800
H	7.39975800	3.09817400	3.21825500
H	8.71797800	3.07693600	2.04636900
H	9.06312500	3.24269400	3.77411700
H	6.41407500	0.91829800	2.54410100
H	7.55999100	0.86589200	1.20399200
H	7.42769000	-0.51274400	2.30048000
H	11.99557200	0.84076600	3.25069600
H	6.23345900	7.40191800	3.60006500
H	7.66646500	7.56737900	4.59481200
H	8.30591200	5.41119000	6.24499800
H	7.12089000	3.25658100	6.80645200
H	4.60018700	5.57999000	2.64798800
H	2.96524100	3.70829200	2.56476300
H	4.88019700	1.76918100	5.90506500
H	3.09787200	1.84684400	4.16161700
H	7.54662200	5.94735300	1.98192100
H	6.53582800	-5.18569700	-4.33547800

H	6.61381900	-6.37847700	-3.04725000
H	6.18970700	-5.69715300	-0.71800700
H	6.63604600	-2.71633500	-3.77150400
H	5.43763200	-3.94841700	0.87304700
H	5.94615600	-0.97754100	-2.18263800
H	5.34615700	-0.52879300	-0.02143400
H	8.87686100	-4.39191600	-3.66681500
H	7.91565400	-3.29051900	2.13419400
H	7.25846800	-4.87959200	2.45046100
H	7.09852200	-4.41128400	4.85230500
H	8.12377500	-1.60383000	4.16811100
H	7.44986200	-2.08880700	5.72737600
H	8.96947500	-2.79656800	5.16498200
H	5.78308200	-2.28461300	3.06910600
H	5.25188400	-2.71550800	4.70346400
H	5.15847200	-3.90729900	3.40320000
H	10.01857700	-3.86102700	3.36349100
H	8.66821500	1.69393000	-1.33820800
H	8.75213400	3.05560800	-0.22813700
H	6.29285800	4.21389200	0.60774500
H	3.85065500	1.29838200	-1.19677700
H	8.59329700	3.05618100	-3.26084900
H	5.34688300	5.81805500	-2.43149300
H	6.85938200	3.94791500	-4.06836200
H	1.27753200	1.76066300	-2.56307800
H	1.55883100	3.25700100	-1.69794300
H	-0.03579400	2.90416200	-4.01098300
H	3.30818800	2.17457200	-3.52635200
H	3.53425200	4.43797700	-2.23840800
H	4.56702300	0.61270600	-6.17249200
H	6.08891800	-0.22917800	-6.26357800
H	7.02965200	2.15826400	-7.12447700
H	1.54707400	-8.06063900	-3.08129100
H	2.90107500	-8.81888200	-3.89811200
H	4.38159200	-6.94081600	-3.14419000
H	1.94429200	-6.08509900	-1.48703400
H	2.80766000	-5.11604100	-2.70078900
H	3.61005400	-5.55438200	-1.20127600
H	4.23246400	-9.13736800	-1.92353800
H	4.58154200	-7.79439200	-0.82136400
H	2.95251100	-8.46256700	-0.89722200
H	1.63732800	-7.68652500	-5.56886100
H	-1.40775000	-4.50185800	-4.50780300
H	-0.00884600	-3.99833900	-3.54680400
H	-0.23045900	-6.85443400	-4.06556500
H	-3.04463900	-5.30741200	-0.36374800
H	-2.06667600	-4.09399600	0.47897800
H	-0.82138100	-3.70009800	-1.47671400
H	0.14204800	-8.09732300	0.92696900
H	-2.65297700	-7.28537200	0.49499800
H	-2.55064600	-6.36109600	-7.64153300
H	-1.81067400	-6.09691700	-6.07489300
H	-3.63917700	-5.02269800	-4.02553800
H	-5.50481200	-3.38677400	-4.54792300
H	-6.40176700	-3.73279200	-6.92892000
H	-3.57714400	-8.45706800	-6.88657100
H	-11.28588200	5.30075500	-2.76868500
H	-11.39826600	3.75637800	-3.58873600
H	-11.83391400	4.23056600	-0.59892500
H	-11.95894700	2.64699800	-1.40003700

H	-13.88392900	3.66472000	-3.12777000
H	1.39954400	0.63140400	-4.78608400
H	2.03332100	-2.31337100	-4.14427800
H	3.18667600	-1.03138500	-4.51642800
H	1.21527200	4.36628100	0.21628900
H	3.39884100	1.27894000	1.73352200
H	-0.38577800	2.34705500	-0.00699800
H	-1.91188000	-0.66061000	1.08662500
H	-5.42019100	3.84120200	-1.59350200
H	5.80641900	1.05536400	-2.94083400
H	2.65097800	-2.16184600	0.29381300
H	1.31241900	-3.37616500	0.22471200
H	2.79285000	-3.57313000	-0.73507000
H	3.84484300	3.20625000	0.35861400
H	-0.32819700	-1.45174400	-3.71969400
H	-13.78174700	5.24238000	-2.31487800
H	-13.34043600	5.09878500	-4.03686300
H	-5.44287600	4.96470700	4.63267300
H	-4.95921100	5.97721500	6.05983600
H	-4.44846100	2.11256500	3.68464400
H	-4.04724800	3.78686000	4.26891400
H	-1.39942300	7.86043900	5.28763000
H	-1.29762300	9.60807500	5.76201900
H	-4.83363100	4.57498800	-4.62850400
H	-3.27873100	5.06975100	-5.41237200
H	1.88980600	9.75527200	-4.60150300
H	2.01738900	8.82716000	-6.11076400
H	8.21728200	7.57036900	2.17446400
H	8.97762200	6.17389000	2.99187600
H	5.59061100	6.38648600	-4.08486400
H	2.29928700	3.11968000	-4.60254700
H	9.64594200	4.01055900	-2.19092700
H	5.32133600	2.41170600	-7.55357000
H	6.23693100	0.99545300	-8.21525000
H	0.02343700	-1.64778100	-8.79665100
H	0.16605900	-3.30645800	-8.19296400
H	-5.02505300	0.02987400	-7.61336100
H	-4.70231900	-1.66027900	-8.09971800
H	-3.29535700	-8.04912500	-5.18408000
H	-1.90949200	-8.40403900	-6.23908900
H	3.12849300	-6.75855500	-5.40020500
H	8.87661000	-6.06382100	-4.23817900
H	8.94087700	-5.71443600	-2.50045500
H	-1.14885200	-9.14547900	0.30681700
H	-1.04647400	-8.73440900	2.06219700
H	2.74109400	-7.79028400	6.72039800
H	4.36700800	-8.18162900	7.39529600
H	9.65692900	-5.11951100	2.17058800
H	9.32435800	-5.40513200	3.88045300
H	11.00069700	0.59278900	4.66675900
H	11.05384400	2.19896000	3.84408400
H	-2.52450200	1.22761300	-0.32794900
H	-7.38350100	1.51080100	0.74061000
H	-8.95248300	-0.28823900	-0.54572200
H	-9.08695400	-1.76380500	1.50333200
H	-8.28140800	-5.58884900	4.43611800
H	-8.72466000	-6.03608200	2.76600200
H	-7.02994100	-5.65976000	3.15679900
H	-9.35696900	-3.67197700	3.29154200
H	-5.93194700	-2.52954900	3.20448500

H	-7.94975400	2.01289000	-1.42261700
H	-9.87192300	-3.16010900	8.32459100
H	-8.33891500	-3.73972600	8.92221500
H	-0.71609200	-1.82795500	3.77757200
H	-0.92010700	-3.56494200	3.59035200
H	-4.25240300	-3.23790200	4.87602600
H	0.17574200	-1.58103700	1.67001800
H	-4.78112000	-1.42178000	1.14054200
H	-7.72927900	-3.22080600	4.82939600
H	0.10385000	-1.97934600	7.42378100
H	1.88260000	-1.94012100	9.33805300
H	4.36812000	-2.70930300	7.70909500
H	4.34245700	-1.66847200	9.14732500
H	3.94362100	-3.39510600	9.28524400
H	-10.21339800	-4.95414500	10.08684500
H	-9.67505900	1.70881900	3.52338600
H	-8.86691100	2.87598600	2.48735200
H	-7.91924500	1.68375000	3.43822200
H	-10.84225700	-5.49834200	8.51298000
H	-9.29139500	-6.09696700	9.07591000
H	-8.66749700	-4.36363600	1.00067900
H	-7.00515800	-3.89415600	1.34431400
H	-5.95471200	-0.02238300	-1.04927100
H	-7.24315200	-0.15724000	-2.23171100
H	2.24183800	-3.71297700	6.86564300
H	-2.26212000	-3.53656500	5.79520700
H	1.62595100	-4.18591700	8.43567100
H	-1.66243700	-1.90309900	6.04622400
H	2.61319200	-1.13978000	6.74551400
H	2.50916200	-0.94448000	2.29613800

TS3 (+2.7)

C	-8.73209500	2.01716500	2.89545200
C	-8.89924000	0.97083900	1.81865400
O	-9.88956000	0.23556500	1.78294600
N	-7.84380700	0.82168300	0.97633900
C	-7.95008900	-0.02218900	-0.21159200
C	-7.67354000	-1.50461500	0.12225000
O	-6.72834900	-2.12982800	-0.39760600
C	-6.94946300	0.51809300	-1.25218500
O	-6.89277400	1.92963900	-1.23263300
N	-8.52186300	-2.07866600	0.98203700
C	-8.40547300	-3.47594200	1.38099100
C	-8.76552300	-3.73976900	2.84500300
C	-8.66545600	-5.23982400	3.13498900
O	-7.90770300	-2.98021700	3.67410800
C	-10.08408100	-5.28096000	8.76840200
C	-8.95327100	-4.43791300	8.17010300
C	-9.01243100	-4.26871500	6.62849500
O	-9.98495700	-4.75656300	6.01797500
O	-8.02446500	-3.62478400	6.13399100
C	-1.72187400	-3.75177100	5.64749300
C	-0.43244600	-4.26995300	6.26244500
O	-0.04153200	-5.42606200	6.10424100
C	-1.75897200	-3.90509300	4.11690000
C	-3.09351900	-3.52787900	3.53848300
C	-4.33357800	-3.53710600	4.14571900
N	-3.22978500	-3.12000300	2.22076400
C	-4.53193000	-2.89796100	2.05612800
N	-5.23521600	-3.13566200	3.18975100
N	0.25709500	-3.38217400	7.03831200
C	1.56843400	-3.71138100	7.56573000
C	2.15721300	-2.50723900	8.29953000
C	3.60012800	-2.75944900	8.72541600
O	2.04458000	-1.32342200	7.50310500
N	-4.36309100	3.63684000	-1.45561200
N	-2.93858500	2.10960900	-0.91179800
N	-3.54335000	0.25653200	-5.31432700
N	-4.32661500	-1.87722100	-1.71091100
N	-0.24133800	-0.54736500	-4.30739600
N	0.42122200	-1.44983500	-6.80203700
N	7.06372300	3.38510200	6.23017100
N	6.02326700	1.70651200	-1.38616000
N	4.92259700	2.82361700	0.16763900
N	7.01228900	4.73515700	-3.19295200
N	3.66411600	4.29313600	-3.02017200
N	0.10073400	-5.60090400	-4.42956500
N	-1.66730800	-4.23515100	-1.63250000
N	-1.87630700	-7.11197900	0.42873000
N	-4.86529800	-4.66920900	-7.39709800
N	-5.25952500	-3.65186100	-5.45681400
C	-3.53608600	4.82309200	-4.43473400
C	-2.96513700	5.10419100	-3.03793500
C	-3.17423900	3.96581600	-2.09754400
C	-2.28080200	2.98743800	-1.75501000
C	-4.19571900	2.52414200	-0.73809400
C	-4.76848400	-0.46095600	-7.30463200
C	-3.50893400	-0.53110700	-6.43518100
C	-2.65146700	0.02016800	-4.17335100
C	-1.18691500	0.40378900	-4.51011100
C	-2.85373600	-1.39090800	-3.58452700
C	-4.25269500	-1.54490300	-3.00770400
C	1.14711800	-0.37944800	-4.69605400
C	1.44027400	-0.85691500	-6.14111800
C	2.11746300	-1.13026300	-3.76829800
C	1.03855900	-1.73148200	-1.16666000
C	0.57111700	-1.88701300	-8.17681900
C	1.93587100	9.04011000	-4.68381500
C	2.37889500	7.72403400	-4.02369200
C	1.65842200	7.33746900	-2.72767700
C	0.18477500	7.00120600	-2.91010500
C	-1.16230800	8.74344500	5.34060200
C	-0.01815400	9.04045900	4.33923000
C	0.21187800	8.02462400	3.24325200
C	-0.71672900	7.82666400	2.21193200
C	1.39165400	7.27149100	3.20825000
C	-0.48878300	6.90235600	1.19691700
C	1.64351200	6.35030200	2.19207000
C	0.69372200	6.15155700	1.18881900
C	-5.42267800	5.85058800	5.43835500
C	-5.20607900	7.02988500	4.45751100
C	-5.60013100	6.62726900	3.04916900
C	-6.92943300	6.33519800	2.71056100
C	-4.62738800	6.41678500	2.06532500
C	-7.27994800	5.83309600	1.45855400
C	-4.95718500	5.92908800	0.80165400
C	-6.28780100	5.61127800	0.48713600
C	-3.62020800	2.93101200	3.75116000
C	-2.27990600	2.30137900	4.17554200

C	-1.21072300	3.35469700	4.50444000
C	-1.80651100	1.29104400	3.12230900
C	-0.84004100	4.27230900	3.34356300
C	3.57798100	-8.29580700	6.12952700
C	4.21780400	-7.02082300	5.54198100
C	3.70300600	-6.65782400	4.16660800
C	4.51389100	-6.82067200	3.03661400
C	2.39619600	-6.17851500	3.99313400
C	4.03601300	-6.51364200	1.76018300
C	1.91616100	-5.88068700	2.71744200
C	2.73217600	-6.04587800	1.59553900
C	11.09374400	0.64949000	3.78916000
C	9.93972500	0.28993300	2.84035800
C	8.55610100	0.84114700	3.23063300
C	8.52823100	2.37359100	3.18737900
C	7.46276200	0.26520500	2.31914400
C	8.25795100	6.29157800	3.02277300
C	7.38123500	6.51795100	4.24949800
C	6.99612300	5.23793000	4.93598000
C	7.69064100	4.58313000	5.92240300
C	5.87140600	4.39622100	4.60691600
C	5.94672900	3.23956000	5.43355400
C	4.81067800	4.50500200	3.69165900
C	5.00988400	2.20431100	5.35847000
C	3.86855200	3.48658800	3.62180000
C	3.97390700	2.34416400	4.44260000
C	8.40460800	-5.44604800	-3.66954300
C	6.86695900	-5.44401800	-3.60044500
C	6.31589500	-4.44031900	-2.61305600
C	5.95152500	-3.15690500	-3.03537200
C	6.17734500	-4.74132000	-1.25178400
C	5.46685200	-2.20327600	-2.14426000
C	5.67739400	-3.80777200	-0.34539400
C	5.32347400	-2.53228300	-0.79387900
C	9.19571500	-5.01883700	2.90684300
C	7.79911700	-4.40835100	2.72528000
C	7.11220200	-3.92627300	4.01621900
C	7.95463200	-2.89403000	4.77703000
C	5.71701600	-3.36774000	3.70114000
C	8.79746600	3.50859300	-2.13359800
C	7.86235500	4.71001800	-2.13222800
C	8.40388200	2.53503100	-0.98818000
C	6.93191000	2.47698400	-0.68076200
C	6.24902300	3.17427000	0.29437200
C	4.83176500	1.93738100	-0.85253600
C	5.79193800	5.51721700	-3.17813900
C	4.70640700	4.65286500	-3.82091800
C	2.81554800	3.17941300	-3.41714100
C	1.54327800	3.04737600	-2.57333300
C	6.22212100	1.84999300	-7.25128500
C	5.76802600	0.92160600	-6.11881000
C	6.20512700	1.37402000	-4.75007200
C	2.07025900	-6.83702500	-5.19634000
C	1.38289600	-5.50471400	-4.87220600
C	2.31511700	-7.75216400	-3.98133400
C	3.34612400	-7.26334700	-2.94766100
C	2.92066500	-5.97560500	-2.23310900
C	3.61607600	-8.37042500	-1.92058600
C	-0.59499600	-4.47976500	-3.82884900
C	-1.46383800	-5.03818400	-2.70271600

C	-2.21562100	-4.83740000	-0.42615600
C	-1.27813400	-5.95759400	0.04195200
C	-1.11574100	-8.33279200	0.64643100
C	-3.05668300	-7.51513700	-6.57104200
C	-2.80585700	-6.01268600	-6.82646900
C	-3.92081700	-5.07672400	-6.47206700
C	-4.15310600	-4.45344700	-5.26400400
C	-5.63941000	-3.81287900	-6.75525500
C	1.73714600	1.93395000	0.85049500
C	2.52458200	0.81382600	1.33047500
C	2.06363300	-0.46494400	1.23367800
C	0.44348800	1.66117800	0.31643800
C	0.00191300	0.36417500	0.20549600
C	0.80572700	-0.77163300	0.59779900
C	-13.10317700	5.17435200	-2.90048400
C	-11.62036000	4.85800900	-2.61022800
C	-11.30461400	4.07100000	-1.32808700
C	-9.78679900	3.83439600	-1.20702400
C	1.74139200	-3.00604400	-0.75217000
O	-2.53228900	-1.22247400	-6.72145300
O	-0.93114400	1.53653800	-4.92200500
O	-5.26377300	-1.35106100	-3.70659500
O	2.57495100	-0.70264400	-6.59379200
O	-0.48506900	7.33807800	-3.86508300
O	-0.38980500	6.33786300	-1.89422200
O	0.88849300	5.24726000	0.17869800
O	-6.57546200	5.10240100	-0.73807600
O	4.82762400	-1.64457300	0.12543200
O	7.83225200	5.52854300	-1.21288100
O	4.80922100	4.28604500	-4.98663400
O	0.40243300	3.64074700	-3.19176600
O	7.12632300	2.15962800	-4.55341500
O	5.53945900	0.77532600	-3.76852700
O	1.95433200	-4.42098500	-4.99198300
O	-1.93746300	-6.17467000	-2.79500300
O	-0.05786000	-5.79128600	0.05453600
O	2.22817100	3.12329100	0.87394100
O	-1.18028700	0.07547900	-0.38867900
O	-9.36467800	2.66399500	-1.39708800
O	-9.08251200	4.86656700	-0.96690600
O	-0.15463500	-1.84762100	-1.65621900
S	2.17966200	-0.52736000	-2.03506400
H	-3.38561500	6.02710800	-2.62480600
H	-1.89224400	5.26339600	-3.10021400
H	-1.25275200	2.87793700	-2.06245300
H	-4.96747900	2.05239500	-0.15864100
H	-3.32857700	3.78554200	-4.72088400
H	-5.63229700	-0.78366600	-6.71750500
H	-2.72644700	-2.12596800	-4.38390100
H	-2.11422600	-1.59027400	-2.81054600
H	-5.25318800	-1.97306900	-1.26951200
H	-3.49727200	-2.09044900	-1.17845600
H	-2.94369800	0.76155600	-3.42287100
H	-4.46857100	0.55029800	-5.03172300
H	1.63694400	-1.89728000	-8.40707300
H	-0.52153300	-1.40261200	-6.42903200
H	2.23433300	6.90683800	-4.73870600
H	3.45614900	7.75839600	-3.81764800
H	1.71129200	8.14440100	-1.98280700
H	2.15391500	6.47135200	-2.27599100

H	0.84954100	9.05908900	-4.78093500
H	-0.19531700	10.02826800	3.89278800
H	0.91367200	9.13424400	4.90909900
H	2.13416100	7.40608200	3.99096200
H	-1.63959100	8.40159700	2.20122000
H	2.56079200	5.77260000	2.18335900
H	-1.21585100	6.74975100	0.40789300
H	0.24563300	5.90653300	-1.27833800
H	-2.14723900	8.95100100	4.91240100
H	-5.76352900	7.91552600	4.78896500
H	-4.14697200	7.31167200	4.45540700
H	-7.71355800	6.49060700	3.44864900
H	-3.58469200	6.63013100	2.29049700
H	-8.31279100	5.61869700	1.20471400
H	-4.18149400	5.79605100	0.05564000
H	-7.59685800	4.92283400	-0.85806000
H	-6.45658200	5.81754700	5.79685400
H	-2.46764800	1.74473600	5.10526500
H	-1.67791200	1.77368600	2.14920100
H	-0.84154600	0.84743900	3.39246500
H	-2.52545700	0.47398700	2.99726300
H	-1.57608400	3.96691200	5.34088600
H	-0.30729600	2.84678000	4.86943800
H	-1.71752800	4.78089700	2.93470400
H	-0.14041700	5.04355700	3.66677600
H	-0.36535800	3.72301500	2.52499900
H	-3.57626100	3.34232400	2.73703300
H	4.04242800	-6.18763400	6.23533700
H	5.30517900	-7.15475100	5.49036900
H	5.53069500	-7.18747600	3.15934200
H	1.74493500	-6.04397600	4.85293700
H	4.67986400	-6.64153300	0.89452900
H	0.89930100	-5.52508000	2.58566200
H	2.33778200	-5.82495400	0.61220600
H	3.63544900	-9.11639500	5.40673100
H	10.17395000	0.65812000	1.83166100
H	9.87993600	-0.80294300	2.75464800
H	8.34059900	0.52358600	4.26326100
H	7.52243800	2.75216000	3.37531300
H	8.83867900	2.73322800	2.19947800
H	9.19023100	2.82729700	3.93020100
H	6.47210800	0.61014200	2.63201700
H	7.60450600	0.58586900	1.28223400
H	7.45762100	-0.82916400	2.32873400
H	12.04984000	0.37109700	3.32908600
H	6.47284900	7.05967300	3.95849800
H	7.90857100	7.16963500	4.95747300
H	8.59614900	4.87497400	6.43537800
H	7.39772700	2.70508200	6.89150200
H	4.72865700	5.37870100	3.05036200
H	3.04501900	3.55601800	2.92161300
H	5.08718900	1.32590000	5.99261700
H	3.22448600	1.56383500	4.35592700
H	7.76198600	5.66346900	2.27722600
H	6.45980300	-5.22388300	-4.59394200
H	6.51904300	-6.45139100	-3.34067100
H	6.44485600	-5.73344800	-0.89401800
H	6.03957300	-2.89197200	-4.08570200
H	5.53374800	-4.05953500	0.69861100
H	5.20145100	-1.21577500	-2.50176300

H	4.44176500	-0.89256900	-0.35026500
H	8.78705400	-4.43430900	-3.83578600
H	7.85804600	-3.56989500	2.01845400
H	7.14402600	-5.15041700	2.25239100
H	6.98444600	-4.80416900	4.66863200
H	8.14563900	-2.01288200	4.15523700
H	7.43234200	-2.55407100	5.67841700
H	8.92212100	-3.30061000	5.08792600
H	5.77893600	-2.51715900	3.01261300
H	5.21716300	-3.02312700	4.61450600
H	5.07861100	-4.12771700	3.24276400
H	9.93402000	-4.27255900	3.21553700
H	8.76605300	1.53126900	-1.23022500
H	8.89079700	2.83940900	-0.05727000
H	6.58934100	3.87233500	1.04011200
H	3.90191100	1.49270000	-1.17149500
H	8.74447800	2.98444900	-3.08794900
H	5.57379200	5.79806500	-2.14394000
H	7.04520200	3.96160000	-3.85487500
H	1.36246700	1.98808300	-2.36993400
H	1.66636400	3.54206900	-1.60739000
H	0.09566300	3.04535600	-3.90008200
H	3.40080900	2.25098800	-3.39142200
H	3.78799900	4.41977600	-2.02395900
H	4.68782100	0.74835300	-6.12194000
H	6.21246800	-0.07687400	-6.24010900
H	7.17654600	2.31624500	-6.99840000
H	1.36112200	-7.93504400	-3.46697000
H	2.64235000	-8.72751500	-4.36428800
H	4.28273700	-7.06030000	-3.48657500
H	1.92632400	-6.08541800	-1.78476100
H	2.90765600	-5.12784200	-2.91802600
H	3.62616800	-5.72804900	-1.43659400
H	3.93673700	-9.30323900	-2.39914000
H	4.39457600	-8.06552300	-1.21381700
H	2.71291300	-8.58280100	-1.33612400
H	1.45104800	-7.37499600	-5.92531600
H	-1.23187900	-3.96346400	-4.56037500
H	0.13293800	-3.75962200	-3.45367400
H	-0.32994600	-6.49903100	-4.24540700
H	-3.22088900	-5.21742200	-0.62713000
H	-2.29476500	-4.08364100	0.36770600
H	-1.06045200	-3.41614900	-1.52967300
H	-0.06112200	-8.07120200	0.54537500
H	-2.86513400	-7.19572200	0.24748100
H	-2.55908900	-5.85844400	-7.88268500
H	-1.91732400	-5.72276000	-6.25438800
H	-3.67388000	-4.54658800	-4.30257200
H	-5.58381300	-2.94046100	-4.80312400
H	-6.48154500	-3.28368900	-7.17882100
H	-3.74584700	-7.95424700	-7.29679100
H	-11.05459400	5.79577800	-2.57139400
H	-11.21665200	4.29605700	-3.46298800
H	-11.64234100	4.64092800	-0.45388900
H	-11.81307100	3.10238600	-1.33010000
H	-13.70574100	4.26640000	-3.00710200
H	1.37591800	0.68870200	-4.68427400
H	1.92388100	-2.20682700	-3.79016400
H	3.12195500	-0.98293300	-4.16675300
H	1.41457600	4.43756900	0.47140300

H	3.48910000	1.02773200	1.77628800
H	-0.15217800	2.48964900	-0.04544200
H	-0.98753100	-0.74751800	-0.95753700
H	-5.25193800	4.17677800	-1.41295400
H	5.89556700	1.10027900	-2.86358100
H	2.64488300	-2.80923000	-0.17877200
H	1.06323400	-3.66476900	-0.20339200
H	2.03868500	-3.52797500	-1.66674700
H	4.10557300	3.12156400	0.71094800
H	-0.45963000	-1.35898300	-3.74680700
H	-13.55164000	5.80052100	-2.12198400
H	-13.11105100	5.72135000	-3.84851400
H	-5.26495900	4.95048100	4.83434000
H	-4.75837100	5.88118100	6.30853800
H	-4.35165200	2.11618400	3.75950300
H	-3.90338800	3.74897900	4.42188100
H	-1.13985400	7.68962600	5.63702600
H	-0.98811800	9.40974500	6.19185800
H	-4.62090100	4.96887900	-4.43254900
H	-3.04790900	5.45193800	-5.18707200
H	2.25458100	9.93713400	-4.14208000
H	2.36191300	9.07600000	-5.69205300
H	8.47938900	7.25299900	2.54901400
H	9.19345100	5.79813500	3.30396200
H	5.85007500	6.43803800	-3.76780100
H	2.46497600	3.29930600	-4.44765900
H	9.82282200	3.85489000	-1.97298800
H	5.47928700	2.63690500	-7.41710800
H	6.35533900	1.22560000	-8.14005500
H	0.06836300	-1.19940700	-8.86541900
H	0.15817100	-2.88755700	-8.33830300
H	-4.93357900	0.57364100	-7.62448800
H	-4.65916500	-1.10142300	-8.18708300
H	-3.45895300	-7.65698900	-5.56410300
H	-2.07906600	-8.00468200	-6.62925600
H	3.00177800	-6.55224800	-5.69684100
H	8.76207100	-6.08555900	-4.48312100
H	8.82807900	-5.81847000	-2.73091600
H	-1.37402200	-9.06900100	-0.12184900
H	-1.26830700	-8.74244400	1.65098100
H	2.52231200	-8.12817200	6.36153800
H	4.13232700	-8.59896100	7.02370900
H	9.53805300	-5.46091800	1.96517600
H	9.18837500	-5.81473300	3.65878700
H	11.03981900	0.08737100	4.72703200
H	11.14523700	1.72734400	3.97952800
H	-2.49540300	1.26682000	-0.53246700
H	-7.28071100	1.65005900	0.81090000
H	-8.97319100	0.06137600	-0.59839700
H	-9.24752900	-1.48302600	1.38433400
H	-9.02758600	-5.42625400	4.14880500
H	-9.27483400	-5.81919800	2.42982800
H	-7.62305500	-5.56747600	3.04452000
H	-9.80906300	-3.42550100	3.01603600
H	-6.27124600	-3.04910300	3.33801300
H	-7.82706500	2.29763600	-1.35715600
H	-8.94895100	-3.43081000	8.60811700
H	-7.97264200	-4.86460500	8.41748100
H	-0.98412300	-3.27819900	3.65854100
H	-1.49909200	-4.94296600	3.87185700
H	-4.66442700	-3.77980500	5.14379600
H	0.24956300	-1.62184800	0.98506000
H	-5.01556400	-2.57263100	1.14548300
H	-8.04515400	-3.25801600	4.66992000
H	0.05051300	-2.39519600	6.96213600
H	1.54290500	-2.29314100	9.18189600
H	4.23053300	-2.95703800	7.84975800
H	3.99871200	-1.88528700	9.24632900
H	3.67097200	-3.62678800	9.38987200
H	-10.00625500	-5.34460600	9.86139400
H	-9.58123800	1.89266000	3.57151700
H	-8.73914200	3.06686700	2.58270900
H	-7.83252600	1.80397300	3.48100600
H	-11.05608100	-4.85356100	8.50728200
H	-10.06730600	-6.29509800	8.35794800
H	-9.05475700	-4.09151300	0.74319700
H	-7.37712500	-3.78935400	1.18804900
H	-5.94948100	0.14875900	-1.00514300
H	-7.19661900	0.11436000	-2.24073800
H	2.24268600	-4.01081100	6.74658700
H	-2.53067400	-4.34873900	6.08358400
H	1.50673600	-4.57460000	8.23706700
H	-1.90993200	-2.70959700	5.92653800
H	2.55627400	-1.46778900	6.69335500
H	2.66823000	-1.28380900	1.60952100

Int5 (-11.1)

C	-8.73303700	2.02992900	2.90663700
C	-8.89907100	0.99890200	1.81610400
O	-9.89489800	0.27169400	1.76240000
N	-7.83912300	0.85543500	0.97931300
C	-7.94359400	0.02245500	-0.21555200
C	-7.67125800	-1.46194300	0.11520300
O	-6.70280600	-2.07865300	-0.37083600
C	-6.94002600	0.56801500	-1.25024000
O	-6.87103600	1.97829000	-1.21534400
N	-8.55298600	-2.04491100	0.93493400
C	-8.45815900	-3.44543100	1.32740400
C	-8.82580600	-3.71531000	2.78895500
C	-8.76831000	-5.22071400	3.06293200
O	-7.94861500	-2.98957900	3.62781300
C	-10.11691100	-5.30102000	8.73003900
C	-8.98052900	-4.47085700	8.12448700
C	-9.05102800	-4.29645400	6.58383400
O	-10.03131300	-4.77755400	5.98025300
O	-8.06384000	-3.65631500	6.08352200
C	-1.76106900	-3.84707300	5.64986800
C	-0.46684000	-4.35223300	6.26499000
O	-0.07787900	-5.51240700	6.13127800
C	-1.80922500	-4.03900100	4.12451100
C	-3.14038900	-3.65928900	3.54072500
C	-4.37937100	-3.62610900	4.14972200
N	-3.27193400	-3.29574400	2.21039000
C	-4.56964100	-3.05733900	2.03849800
N	-5.27508500	-3.24265200	3.18055700
N	0.23209300	-3.44660700	7.01062600
C	1.54229400	-3.76905200	7.54395300
C	2.11860700	-2.56511400	8.28696900
C	3.55757500	-2.81254800	8.72866700

O	2.01100600	-1.38060300	7.49047700
N	-4.32230700	3.59385900	-1.44380000
N	-2.83124400	2.11171100	-0.93586600
N	-3.55163600	0.29978800	-5.30473500
N	-4.30286000	-1.74660900	-1.65911000
N	-0.26528500	-0.52433300	-4.32927500
N	0.40363100	-1.40549900	-6.81332500
N	7.07666300	3.29921800	6.23532200
N	6.02585900	1.65036600	-1.32846600
N	4.92332900	2.81457700	0.19026700
N	7.02734900	4.73097200	-3.15380700
N	3.68274200	4.29124200	-2.99449800
N	0.06277200	-5.57552000	-4.48845400
N	-1.67713300	-4.22049300	-1.68396300
N	-1.90083400	-7.10296300	0.38639100
N	-4.87045200	-4.57396400	-7.43211100
N	-5.27361300	-3.60507200	-5.46860300
C	-3.52306200	4.86384700	-4.40235200
C	-2.97692000	5.15077300	-2.99432800
C	-3.14670200	3.99131900	-2.07131200
C	-2.21514600	3.04286700	-1.74813000
C	-4.10535900	2.46680100	-0.75716700
C	-4.77493800	-0.39566400	-7.30773000
C	-3.51861600	-0.47776200	-6.43274500
C	-2.66627600	0.05577400	-4.15932100
C	-1.19654000	0.45285700	-4.45794200
C	-2.87031000	-1.36136600	-3.58454500
C	-4.25609900	-1.50136600	-2.97523500
C	1.12995700	-0.37059900	-4.69632400
C	1.43095100	-0.84170200	-6.13945200
C	2.09188200	-1.12777700	-3.76173700
C	1.16553400	-1.59954400	-1.02693800
C	0.55959800	-1.83686600	-8.18654800
C	1.96591300	9.06138700	-4.62087600
C	2.39406400	7.73528600	-3.97170600
C	1.66805000	7.34620300	-2.68000700
C	0.18557300	7.04993600	-2.85961400
C	-1.13865800	8.71059100	5.39974300
C	0.00649000	9.01191400	4.40189400
C	0.22762600	8.00564300	3.29616300
C	-0.71316500	7.81216300	2.27515400
C	1.40842400	7.25556800	3.24364000
C	-0.49592000	6.89416800	1.25246800
C	1.64983400	6.34134000	2.21885900
C	0.68818100	6.14690600	1.22636600
C	-5.41059300	5.83386700	5.47617800
C	-5.18537600	7.01491300	4.49807300
C	-5.57356400	6.61489900	3.08699000
C	-6.90519600	6.35063900	2.73528300
C	-4.59517600	6.37525100	2.11501100
C	-7.25325200	5.84926600	1.48218000
C	-4.92174700	5.88638200	0.85090900
C	-6.25604400	5.59864000	0.52257400
C	-3.61861200	2.91854700	3.77062700
C	-2.28168900	2.28151500	4.19558200
C	-1.20931300	3.32863900	4.53465800
C	-1.80867600	1.27275900	3.14033700
C	-0.82799300	4.24943000	3.37980800
C	3.53497000	-8.35286800	6.07644600
C	4.17411500	-7.07549000	5.49393100
C	3.65072500	-6.70288300	4.12396600
C	4.46115800	-6.84204400	2.99046200
C	2.33735600	-6.23830900	3.95818300
C	3.97678100	-6.52726700	1.71837700
C	1.85085000	-5.93379600	2.68631900
C	2.66612800	-6.07651900	1.56059300
C	11.08608300	0.57909200	3.80051600
C	9.93651200	0.22924400	2.84273700
C	8.55069900	0.78157000	3.22377400
C	8.52583300	2.31427300	3.19202800
C	7.46677700	0.21419800	2.29582000
C	8.27297200	6.23728900	3.07020400
C	7.39627900	6.45875700	4.29791500
C	7.00724300	5.17282200	4.97143500
C	7.70719600	4.49860900	5.94086500
C	5.87441500	4.34296500	4.64029500
C	5.95122600	3.17264300	5.44753500
C	4.80308600	4.47446000	3.74051800
C	5.00669400	2.14492500	5.36647100
C	3.85227300	3.46442800	3.66635800
C	3.96021600	2.30749800	4.46632500
C	8.37664600	-5.45632600	-3.69946500
C	6.83928700	-5.42062900	-3.61889400
C	6.33509100	-4.41068500	-2.61174800
C	6.07824000	-3.08988500	-2.99755500
C	6.15285000	-4.73934300	-1.26233200
C	5.67708800	-2.12360400	-2.07889100
C	5.74056000	-3.79046400	-0.32755400
C	5.52144200	-2.47190100	-0.73504600
C	9.16617900	-5.07578600	2.87962000
C	7.77364700	-4.45589700	2.70549200
C	7.09627600	-3.97214300	4.00081300
C	7.94687800	-2.94297700	4.75667800
C	5.70149800	-3.40785000	3.69431300
C	8.80489900	3.48846100	-2.10203600
C	7.87361700	4.69260500	-2.09120600
C	8.40616200	2.50702000	-0.96551500
C	6.93291000	2.45218800	-0.65423800
C	6.24868200	3.17683300	0.29984700
C	4.83232300	1.89422300	-0.79933500
C	5.80693400	5.51372800	-3.13684800
C	4.72709500	4.65019100	-3.78832600
C	2.82131000	3.18923000	-3.39269900
C	1.54325300	3.09428000	-2.55755200
C	6.22482100	1.87186900	-7.23397700
C	5.77015100	0.94094400	-6.10461300
C	6.20713200	1.39072300	-4.73535300
C	2.03719400	-6.81304300	-5.23939600
C	1.34607200	-5.48009000	-4.92783600
C	2.27209500	-7.72256900	-4.01840800
C	3.29890000	-7.23417700	-2.98035100
C	2.88178900	-5.93423900	-2.28296200
C	3.54491600	-8.33393900	-1.93938500
C	-0.63400800	-4.45089100	-3.89546300
C	-1.50995800	-5.00514500	-2.77408200
C	-2.22810400	-4.83423200	-0.48550600
C	-1.29525500	-5.96624000	-0.03971000
C	-1.15584000	-8.33365400	0.59295600
C	-3.09103800	-7.46096000	-6.62012500
C	-2.83556400	-5.95970500	-6.87488800

C	-3.94238400	-5.01917700	-6.50808700
C	-4.18016400	-4.42600600	-5.28583900
C	-5.64062100	-3.72520900	-6.77491400
C	1.70316400	1.93448900	0.85109200
C	2.52711300	0.79285100	1.28923700
C	2.16727700	-0.47329500	1.03453600
C	0.41678700	1.67898700	0.33903800
C	-0.01603100	0.38241500	0.06030800
C	0.89889000	-0.81251400	0.31667900
C	-13.08932100	5.24304600	-2.87034900
C	-11.60624100	4.92031600	-2.58455000
C	-11.28771100	4.11964700	-1.31157900
C	-9.76897300	3.88067600	-1.19395400
C	1.87043000	-2.92718800	-0.80238400
O	-2.54308600	-1.17091200	-6.72013200
O	-0.92338100	1.61829200	-4.74802600
O	-5.27693400	-1.36992200	-3.67415800
O	2.57233600	-0.71179300	-6.58158800
O	-0.47744000	7.40215500	-3.81370300
O	-0.40402600	6.40992700	-1.83744200
O	0.87407800	5.24850200	0.20870600
O	-6.54211900	5.09349300	-0.70369100
O	5.13604500	-1.56149600	0.21596400
O	7.84191300	5.50026600	-1.16214400
O	4.84064500	4.28083400	-4.95277400
O	0.45498600	3.80037700	-3.15085700
O	7.14360200	2.15542000	-4.53198400
O	5.51919500	0.80962400	-3.75730500
O	1.91419500	-4.39538500	-5.05328800
O	-2.02468200	-6.12095500	-2.89051400
O	-0.07225700	-5.82503100	-0.07961500
O	2.20766400	3.11083200	0.93263800
O	-1.15409100	0.10222400	-0.46746500
O	-9.34852500	2.71029400	-1.38946200
O	-9.06227400	4.90965300	-0.94959300
O	-0.06268100	-1.87023100	-1.66127800
S	2.22823100	-0.45033800	-2.07046100
H	-3.44285400	6.04962500	-2.57679200
H	-1.91148900	5.35748200	-3.03998400
H	-1.18260800	2.98414700	-2.04906100
H	-4.85373500	1.95133700	-0.18439800
H	-3.31311700	3.82623600	-4.68480300
H	-5.64204200	-0.71858700	-6.72564500
H	-2.77752200	-2.09246700	-4.39253900
H	-2.11627900	-1.57976200	-2.83089200
H	-5.22232700	-1.84410600	-1.20129100
H	-3.45939500	-1.84103600	-1.11407200
H	-2.96691300	0.78665100	-3.40301700
H	-4.47144400	0.61703600	-5.03019800
H	1.62606900	-1.85001500	-8.41285900
H	-0.54498800	-1.32991200	-6.46014600
H	2.23887900	6.92626500	-4.69382400
H	3.47172200	7.75419800	-3.76530100
H	1.74405500	8.13895600	-1.92207000
H	2.14195900	6.46004900	-2.24427300
H	0.87995900	9.08932400	-4.72008100
H	-0.16545700	10.00538800	3.96596000
H	0.93939000	9.09431900	4.97182500
H	2.15923800	7.38691600	4.01893500
H	-1.63776200	8.38419900	2.27967300

H	2.56858300	5.76613700	2.19416800
H	-1.23262000	6.74392100	0.47213600
H	0.22300500	5.94127100	-1.24157500
H	-2.12267300	8.92482700	4.97278800
H	-5.74169700	7.90193600	4.82760000
H	-4.12520700	7.29296000	4.50198900
H	-7.69374800	6.52836500	3.46370000
H	-3.55035000	6.56644600	2.35014900
H	-8.28795400	5.65719800	1.21807800
H	-4.14140000	5.72805000	0.11480900
H	-7.56317600	4.93973000	-0.83115900
H	-6.44537200	5.80494300	5.83229700
H	-2.47495100	1.72162800	5.12235000
H	-1.67100900	1.75408900	2.16806700
H	-0.84861100	0.82169800	3.41666000
H	-2.53331400	0.46164200	3.01006900
H	-1.57628700	3.93876500	5.37195500
H	-0.31027800	2.81475200	4.90256800
H	-1.70149200	4.76232600	2.96816300
H	-0.12728300	5.01724300	3.70957300
H	-0.35179100	3.70067700	2.56204200
H	-3.57165400	3.33509900	2.75903500
H	4.00392300	-6.24670100	6.19387500
H	5.26101300	-7.21004300	5.43441400
H	5.48287100	-7.19701900	3.10711100
H	1.68669600	-6.12010200	4.82105400
H	4.62096900	-6.63704600	0.85052700
H	0.82923800	-5.58869200	2.56159000
H	2.26557700	-5.85185200	0.58045800
H	3.58886500	-9.17032500	5.34996800
H	10.17971300	0.60264400	1.83801100
H	9.87423100	-0.86285300	2.74975800
H	8.32319400	0.45637600	4.25133300
H	7.51927200	2.69362200	3.37541600
H	8.84454000	2.68109700	2.20940100
H	9.18316400	2.76069500	3.94323400
H	6.47324500	0.56009500	2.59868500
H	7.62404800	0.54156500	1.26301600
H	7.45704000	-0.87976300	2.29709600
H	12.04300900	0.30102900	3.34179900
H	6.49009000	7.00607900	4.01100800
H	7.92588000	7.10225300	5.01157500
H	8.61943200	4.77694000	6.44939900
H	7.41169700	2.60819800	6.88465300
H	4.71823300	5.36011300	3.11616900
H	3.01906700	3.55209600	2.98007000
H	5.08497700	1.25599900	5.98560300
H	3.20305900	1.53447500	4.37930700
H	7.77483100	5.61567400	2.32073200
H	6.42804500	-5.17826500	-4.60557600
H	6.46688600	-6.42012200	-3.36419700
H	6.32763600	-5.76145100	-0.93332300
H	6.18681300	-2.80391100	-4.04053400
H	5.57642800	-4.06007100	0.70865700
H	5.47762300	-1.11249300	-2.41214400
H	5.06307300	-0.68921800	-0.20017300
H	8.77470800	-4.44911100	-3.85873400
H	7.83490100	-3.61641200	2.00030600
H	7.11203400	-5.19279800	2.23379400
H	6.96933500	-4.84968200	4.65384700

H	8.13595900	-2.06214700	4.13389100
H	7.43158700	-2.60200500	5.66169100
H	8.91531600	-3.35223300	5.06094700
H	5.76239900	-2.55995200	3.00250700
H	5.20975500	-3.05863200	4.61033300
H	5.05662800	-4.16661400	3.24286200
H	9.90890400	-4.33581300	3.19319100
H	8.76240200	1.50308600	-1.21455500
H	8.89382100	2.80298000	-0.03235800
H	6.58795100	3.90349900	1.01821200
H	3.90482900	1.43692800	-1.10897700
H	8.75256400	2.97284000	-3.06091400
H	5.58610200	5.78739300	-2.10140100
H	7.06254300	3.96646100	-3.82576500
H	1.28943300	2.04031700	-2.41724700
H	1.69389600	3.52131400	-1.56331000
H	0.08727000	3.23208400	-3.85098700
H	3.38836100	2.25052100	-3.35793300
H	3.78586700	4.43881000	-1.99924900
H	4.69032000	0.76649400	-6.10793500
H	6.21651300	-0.05652400	-6.22776200
H	7.18126200	2.33380900	-6.98037000
H	1.31418600	-7.89811200	-3.50882600
H	2.59687100	-8.70158700	-4.39399500
H	4.24288800	-7.04866300	-3.51300400
H	1.88390400	-6.02889800	-1.83955800
H	2.88129700	-5.09350200	-2.97650900
H	3.58444800	-5.68558700	-1.48394800
H	3.85469300	-9.27681900	-2.40523800
H	4.32279700	-8.03352600	-1.23004300
H	2.63438600	-8.52528000	-1.35931300
H	1.42393200	-7.35488600	-5.97064400
H	-1.27007400	-3.94005700	-4.63228400
H	0.09529700	-3.73276600	-3.51931000
H	-0.37220300	-6.47258100	-4.31074700
H	-3.23438000	-5.20607800	-0.69348500
H	-2.30581000	-4.09512200	0.32194400
H	-1.02416600	-3.44692500	-1.55682200
H	-0.09859700	-8.08235700	0.49337600
H	-2.89997800	-7.16497500	0.26163800
H	-2.59882700	-5.80459300	-7.93318900
H	-1.93973600	-5.67567500	-6.31142600
H	-3.71585000	-4.55721400	-4.32157600
H	-5.58852700	-2.90239500	-4.79961600
H	-6.47177300	-3.17471600	-7.19289600
H	-3.78131200	-7.89657600	-7.34718800
H	-11.03870700	5.85667300	-2.53759300
H	-11.20528800	4.36662500	-3.44405000
H	-11.62372400	4.68025500	-0.43062500
H	-11.79659800	3.15127400	-1.32248600
H	-13.69474500	4.33772500	-2.98304700
H	1.35461900	0.69745500	-4.67378000
H	1.85029300	-2.19342300	-3.73366200
H	3.08842300	-1.04307600	-4.19630200
H	1.39408800	4.43877700	0.50180700
H	3.45452500	1.02180700	1.80306400
H	-0.20908600	2.52494900	0.08230300
H	-0.67452000	-1.13017200	-1.38026400
H	-5.22517600	4.10309000	-1.38688200
H	5.88072200	1.11213100	-2.85329700
H	2.82744500	-2.79258200	-0.30137000
H	1.23154500	-3.60274400	-0.22474200
H	2.06630900	-3.39921500	-1.76617200
H	4.11140400	3.12046500	0.73565800
H	-0.55105200	-1.44080000	-4.02404900
H	-13.53508700	5.86532000	-2.08722700
H	-13.09482400	5.79640900	-3.81482400
H	-5.25605400	4.93715300	4.86660500
H	-4.74649400	5.85615300	6.34705600
H	-4.35332800	2.10655400	3.77331500
H	-3.89904700	3.73313000	4.44668700
H	-1.12070500	7.65473900	5.68928500
H	-0.96248700	9.37058900	6.25555700
H	-4.60727500	5.01480100	-4.39989000
H	-3.03213000	5.49660600	-5.15024500
H	2.28726000	9.95383100	-4.07311700
H	2.39200600	9.10249400	-5.62881700
H	8.49845200	7.20092200	2.60294900
H	9.20634300	5.73825400	3.34856000
H	5.86908100	6.43812100	-3.72040600
H	2.47209400	3.31733100	-4.42269900
H	9.83097800	3.82959900	-1.93558000
H	5.48512900	2.66273300	-7.39499700
H	6.35602700	1.25286600	-8.12685900
H	0.06010700	-1.14245600	-8.87129100
H	0.14283000	-2.83452500	-8.35544900
H	-4.93551300	0.64180900	-7.62098700
H	-4.66740500	-1.03048100	-8.19455100
H	-3.49422800	-7.60810500	-5.61449600
H	-2.11524400	-7.95409000	-6.68140000
H	2.97109900	-6.52715700	-5.73621100
H	8.73233100	-6.09164800	-4.51751200
H	8.79858100	-5.83653500	-2.76344200
H	-1.41784300	-9.06444100	-0.18089000
H	-1.31174300	-8.75001700	1.59396100
H	2.47876000	-8.18184800	6.31136800
H	4.08643200	-8.66229200	6.97089700
H	9.50749100	-5.51294900	1.93529000
H	9.15571800	-5.87654400	3.62644300
H	11.02965800	0.01100100	4.73461500
H	11.14185100	1.65546800	3.99802000
H	-2.31853300	1.26594900	-0.59622400
H	-7.26369400	1.67853100	0.82972700
H	-8.96572300	0.11078300	-0.60315300
H	-9.28576800	-1.44753000	1.32194400
H	-9.13008600	-5.40752000	4.07713200
H	-9.39769100	-5.77474100	2.35493000
H	-7.73641700	-5.57786300	2.96373600
H	-9.86059900	-3.37352300	2.96154900
H	-6.30878200	-3.12708400	3.32127500
H	-7.80255200	2.35118000	-1.34047500
H	-8.95881100	-3.46530300	8.56543600
H	-8.00351100	-4.91144600	8.36184500
H	-1.02840500	-3.43435800	3.64663500
H	-1.56307200	-5.08581300	3.90465700
H	-4.71315600	-3.82996000	5.15553300
H	0.32078800	-1.52087100	0.92792300
H	-5.04871100	-2.75402300	1.11778200
H	-8.09131100	-3.27605500	4.61924600
H	0.02265800	-2.46149100	6.92116100

H	1.49328600	-2.35469400	9.16235900
H	4.19811500	-3.00849500	7.85999300
H	3.94756400	-1.93678600	9.25339600
H	3.62393000	-3.67932700	9.39429000
H	-10.02955500	-5.37060900	9.82199100
H	-9.58454800	1.90098300	3.57877400
H	-8.73690200	3.08236400	2.60079400
H	-7.83523000	1.81010700	3.49091900
H	-11.08558400	-4.85939500	8.48036400
H	-10.11785600	-6.31332700	8.31483600
H	-9.11693700	-4.04618800	0.68519300
H	-7.43489700	-3.77387600	1.13382900
H	-5.94342200	0.18943900	-1.00803400
H	-7.19165000	0.17623500	-2.24284900
H	2.22484700	-4.06109300	6.72871100
H	-2.56836400	-4.43026800	6.10709100
H	1.47616900	-4.63681600	8.20868300
H	-1.94383100	-2.79747400	5.90356600
H	2.53281400	-1.52285000	6.68684700
H	2.81808200	-1.29091400	1.32483400

TS4 (+9.1)

C	-8.86650000	1.68349400	3.00414100
C	-8.85112500	0.58448700	1.97069400
O	-9.62191900	-0.37661100	2.01993500
N	-7.81232000	0.64047500	1.09256800
C	-7.69615600	-0.38533900	0.07125400
C	-6.99659700	-1.61318600	0.69718600
O	-5.75778100	-1.78856500	0.56427300
C	-6.90701600	0.19648400	-1.12896100
O	-7.05118300	1.59385300	-1.22902100
N	-7.77856700	-2.45329800	1.36832100
C	-7.34466400	-3.75484600	1.87904400
C	-7.61953000	-4.03083900	3.36030100
C	-7.41437400	-5.52502800	3.63722000
O	-6.76483800	-3.23659600	4.14714900
C	-9.74056700	-5.45257100	9.15943100
C	-9.03886000	-4.25221200	8.50879100
C	-8.38692800	-4.52665100	7.11760900
O	-8.39816800	-5.70678800	6.70139100
O	-7.90097000	-3.49315100	6.55750200
C	-0.87200300	-4.01804400	5.01362600
C	0.23432500	-4.34405100	6.01435400
O	0.77215100	-5.44620200	6.09387300
C	-0.39461800	-3.12450400	3.85039000
C	-1.52547200	-2.64790000	2.98843600
C	-2.88649000	-2.82962700	3.11450700
N	-1.31283300	-1.86816200	1.85749600
C	-2.50580300	-1.58476600	1.33095000
N	-3.47536400	-2.15580000	2.06311700
N	0.59012400	-3.31660900	6.83968000
C	1.79462700	-3.36649000	7.64773900
C	1.94222000	-2.05138100	8.41630000
C	3.27338100	-1.97281500	9.15554800
O	1.75743300	-0.93241800	7.54325800
N	-4.64343300	3.41227600	-1.44927300
N	-3.03968200	2.06767600	-0.90637700
N	-3.78973900	-0.24943600	-5.17364200
N	-4.13995100	-2.51639600	-1.53030800
N	-0.43947200	-0.92110900	-4.26709600
N	0.23916100	-1.70082500	-6.76285700
N	7.02233600	3.98073100	6.01091200
N	5.80932900	1.89008000	-1.57753200
N	4.66420000	2.97808700	-0.03382100
N	6.59958600	4.93700000	-3.51936500
N	3.27679400	4.34365600	-3.25927100
N	0.18610100	-5.93190400	-4.01568500
N	-1.44836900	-4.57469000	-1.16631600
N	-1.66748700	-7.24451700	0.59605400
N	-4.84140000	-5.11866900	-7.14117300
N	-5.34221300	-4.18753000	-5.18302000
C	-3.96351300	4.46847100	-4.53244400
C	-3.44580600	4.92697100	-3.15689300
C	-3.51800700	3.84058800	-2.14285900
C	-2.51639600	2.97737900	-1.79783400
C	-4.32732900	2.34920900	-0.69985900
C	-4.99282600	-0.96944500	-7.18603700
C	-3.72449800	-0.97854500	-6.32997000
C	-2.85539400	-0.47685100	-4.06704700
C	-1.41838200	0.00552000	-4.39631700
C	-2.96127500	-1.93186400	-3.55617700
C	-4.26970400	-2.13582200	-2.81458200
C	0.95158300	-0.71922400	-4.62049200
C	1.27632300	-1.19362600	-6.05901200
C	1.90338100	-1.43184600	-3.64181000
C	0.89525300	-1.67195500	-0.76321900
C	0.39175000	-2.16677400	-8.12379500
C	1.28935100	8.93335200	-5.05013400
C	1.82927600	7.67242400	-4.35285300
C	1.17702200	7.29595900	-3.01752300
C	-0.26768800	6.83388900	-3.14531300
C	-1.58832100	8.85231000	5.04377200
C	-0.47477900	9.17209300	4.01461000
C	-0.19165500	8.12759500	2.95927100
C	-1.09548100	7.85843100	1.92268200
C	1.01727600	7.42083900	2.96635500
C	-0.81295800	6.91573700	0.93830400
C	1.32252000	6.48104600	1.98314200
C	0.40157400	6.21646900	0.96648100
C	-5.69903000	5.76257500	5.33684000
C	-5.56782100	6.92030900	4.31792600
C	-5.97938500	6.45662200	2.93423500
C	-7.29578700	6.06600900	2.64933600
C	-5.02643400	6.28621500	1.92370300
C	-7.65097000	5.50455300	1.42371800
C	-5.36205500	5.74089700	0.68615300
C	-6.67660000	5.32241400	0.42683700
C	-3.78974700	2.87405200	3.71491300
C	-2.41544500	2.34480900	4.16654300
C	-1.40208800	3.47302500	4.40907000
C	-1.90049500	1.28002200	3.19277900
C	-1.09292500	4.33362600	3.18732000
C	3.99698900	-7.89839500	6.33043800
C	4.58294300	-6.62192400	5.68933900
C	4.24395900	-6.44475900	4.22672300
C	5.23956500	-6.55953100	3.24895900
C	2.93041500	-6.17449200	3.81892800
C	4.93899300	-6.38527100	1.89677200
C	2.62499100	-6.00430300	2.46832700

C	3.63170500	-6.09876200	1.50461300
C	11.01605700	1.30870900	3.51161800
C	9.85797700	0.86112800	2.60618200
C	8.46146600	1.36685600	3.01182300
C	8.36303400	2.89295700	2.90303200
C	7.37058000	0.70389900	2.15896300
C	7.89219800	6.77577200	2.60853200
C	7.03001600	7.00375400	3.84527000
C	6.76610300	5.74157300	4.61580800
C	7.54273100	5.20261400	5.61100300
C	5.70108000	4.79815100	4.37769600
C	5.89527600	3.70217800	5.26505800
C	4.61033500	4.76673900	3.49265300
C	5.04588000	2.59217000	5.27794500
C	3.75283800	3.67415000	3.50964700
C	3.97618700	2.59469500	4.38998200
C	8.47931600	-5.17690800	-3.66606600
C	6.94809300	-5.26156100	-3.56985000
C	6.35216200	-4.25317900	-2.61613000
C	5.84036700	-3.04136200	-3.09327500
C	6.31503500	-4.47829300	-1.23374100
C	5.30192600	-2.08646300	-2.23539200
C	5.75896500	-3.54619800	-0.35958500
C	5.24697800	-2.34624300	-0.86263700
C	9.38082800	-4.47294700	2.87186200
C	7.95866200	-3.92622000	2.69278000
C	7.26435500	-3.44181000	3.97803300
C	8.06525900	-2.35054700	4.70001800
C	5.84050100	-2.96052800	3.66394200
C	8.46114300	3.83780300	-2.45451600
C	7.46710100	4.98977500	-2.47372400
C	8.14148700	2.88705100	-1.26941300
C	6.67661200	2.75215100	-0.92637200
C	5.96491500	3.43296100	0.04108700
C	4.61702500	2.04976600	-1.01844200
C	5.34264100	5.65814200	-3.50570300
C	4.28846500	4.71711800	-4.09086600
C	2.48012300	3.17215000	-3.59666200
C	1.24620600	2.99165300	-2.71165800
C	5.87026500	1.87021700	-7.45506700
C	5.49736000	0.96084600	-6.27756400
C	5.95354700	1.47547200	-4.93519400
C	2.19163200	-6.92694700	-5.00405100
C	1.34291200	-5.68759100	-4.70217300
C	2.60461800	-7.72609000	-3.75062600
C	3.62590200	-7.01921700	-2.84045900
C	3.08846500	-5.72759700	-2.21197300
C	4.11999800	-7.97876200	-1.75180000
C	-0.59979000	-4.86102700	-3.43522500
C	-1.36658900	-5.40155200	-2.22805700
C	-2.17430100	-4.94140400	0.04665400
C	-1.37865700	-5.95280100	0.88927000
C	-0.79925900	-8.36113900	0.95626200
C	-2.92253200	-7.90113400	-6.24123200
C	-2.75051900	-6.39840200	-6.54903300
C	-3.90989900	-5.51675100	-6.19992000
C	-4.20717900	-4.94750500	-4.97938500
C	-5.67205400	-4.31862600	-6.49853200
C	1.47928800	2.08417900	0.84338700
C	2.25696400	0.97698200	1.36745200

C	1.89240500	-0.30857000	1.10864600
C	0.22183100	1.79466200	0.26827100
C	-0.15592700	0.48328700	-0.02563000
C	0.68176200	-0.65979600	0.38499100
C	-13.50326300	4.41327100	-2.80266800
C	-12.00084700	4.16441900	-2.53911900
C	-11.62406800	3.37250100	-1.27565200
C	-10.09135300	3.22623800	-1.16491500
C	1.60232200	-2.96088300	-0.37866200
O	-2.70999700	-1.59722600	-6.65253300
O	-1.21854400	1.17880200	-4.70542300
O	-5.37097000	-1.95147900	-3.35517800
O	2.42912000	-1.09712600	-6.47744000
O	-0.98581400	7.09389300	-4.09041100
O	-0.76071500	6.14986200	-2.10298600
O	0.66178700	5.29696400	-0.01150900
O	-6.96286500	4.75550600	-0.77277000
O	4.69884000	-1.46329100	0.02579100
O	7.41247800	5.83749900	-1.58247000
O	4.39275200	4.30330300	-5.24144400
O	0.06785700	3.50273100	-3.33028800
O	6.83142800	2.32100300	-4.79420900
O	5.36070900	0.86085900	-3.92048800
O	1.67654400	-4.54751900	-5.01404400
O	-1.88419700	-6.52630500	-2.26982000
O	-0.54434300	-5.59400200	1.71756500
O	1.97408500	3.28282800	0.87695700
O	-1.25485500	0.20660900	-0.69501000
O	-9.59907800	2.08402500	-1.36452100
O	-9.45446500	4.29802000	-0.91632000
O	-0.32556500	-2.01239700	-1.38461900
S	1.98970200	-0.65568200	-1.98689200
H	-3.99733500	5.81260200	-2.82271100
H	-2.40297000	5.22377500	-3.22524300
H	-1.49488100	2.95307000	-2.14397300
H	-5.01052500	1.82875800	-0.05491900
H	-3.68315800	3.42987700	-4.73222400
H	-5.84089100	-1.30883200	-6.58437500
H	-2.91781300	-2.61168800	-4.41129900
H	-2.13524000	-2.16065500	-2.88364400
H	-4.92756500	-2.42601600	-0.88182400
H	-3.21993100	-2.57317500	-1.12403300
H	-3.16662900	0.20456200	-3.26968100
H	-4.71485000	0.02456100	-4.87248200
H	1.45374600	-2.13549300	-8.36992800
H	-0.70633400	-1.64593100	-6.40127200
H	1.71177800	6.81922200	-5.02965900
H	2.90858600	7.78013400	-4.18630000
H	1.18182900	8.14179400	-2.31521100
H	1.75225900	6.49721600	-2.53821000
H	0.20165600	8.88692200	-5.11757300
H	-0.71532500	10.12802300	3.52998300
H	0.45498700	9.34575800	4.56904200
H	1.74055300	7.60943100	3.75612500
H	-2.04097500	8.39438700	1.88214900
H	2.25913100	5.93577800	2.00840900
H	-1.52228300	6.70900300	0.14552900
H	-0.08387200	5.80467000	-1.47346900
H	-2.58895800	8.99624800	4.62611000
H	-6.15956400	7.78675700	4.64109300

H	-4.52472800	7.25385400	4.27750900
H	-8.06411200	6.18851800	3.40993800
H	-3.99393900	6.57548500	2.10762900
H	-8.67414600	5.21235200	1.21072000
H	-4.60339200	5.63674200	-0.08102100
H	-7.96232900	4.48815400	-0.85132700
H	-6.72140900	5.68844700	5.72202000
H	-2.57086500	1.85093300	5.13725800
H	-1.77623400	1.68402600	2.18408800
H	-0.92749100	0.88058900	3.49985000
H	-2.60257100	0.44208900	3.13533000
H	-1.79161900	4.11692900	5.21038100
H	-0.46856200	3.03931000	4.79311100
H	-1.99912300	4.77377500	2.76123800
H	-0.42899700	5.15610100	3.45403800
H	-0.60013000	3.75888800	2.39833400
H	-3.77301500	3.24569600	2.68504100
H	4.23100300	-5.75052600	6.25581800
H	5.67452600	-6.63822000	5.79248500
H	6.26247000	-6.77106300	3.55177200
H	2.14248800	-6.09044700	4.56090400
H	5.72575300	-6.46639600	1.15202700
H	1.59930000	-5.80947300	2.17228800
H	3.39827700	-5.94950900	0.45832500
H	4.08595700	-8.74513300	5.64185400
H	10.05061000	1.19975300	1.57858900
H	9.84577600	-0.23595400	2.56361400
H	8.28836200	1.08664400	4.06314900
H	7.34518500	3.23239300	3.09847900
H	8.63418600	3.22242200	1.89330600
H	9.01916600	3.40827100	3.61015600
H	6.37501800	1.02807400	2.47820300
H	7.47526700	0.97377900	1.10344700
H	7.40886600	-0.38786200	2.22615700
H	11.97400400	1.05917200	3.03896800
H	6.07542100	7.45714400	3.55135700
H	7.52284200	7.73399300	4.49974000
H	8.43842300	5.59390800	6.07251500
H	7.43374700	3.36691600	6.69290900
H	4.44135100	5.58974200	2.80328900
H	2.91098100	3.63766800	2.82754300
H	5.21746600	1.75901200	5.95363600
H	3.29241900	1.75174600	4.36967300
H	7.41393500	6.09606200	1.89729400
H	6.51397700	-5.10810400	-4.56457100
H	6.66856300	-6.27727100	-3.26357100
H	6.70644600	-5.41115700	-0.83323300
H	5.85782200	-2.83334500	-4.15991800
H	5.69115800	-3.74249000	0.70336700
H	4.93506600	-1.14777400	-2.63231800
H	4.15734100	-0.82631900	-0.46999200
H	8.80185500	-4.15280700	-3.87607600
H	7.97677100	-3.10316100	1.96571200
H	7.33033300	-4.70485200	2.24587000
H	7.18501400	-4.30704500	4.65445300
H	8.20492500	-1.47798600	4.05306700
H	7.53944600	-2.01235500	5.59997500
H	9.05547500	-2.70204300	5.00686500
H	5.85206600	-2.12469600	2.95515300
H	5.33138700	-2.61989200	4.57326500

H	5.23920500	-3.76290000	3.22855900
H	10.09235500	-3.68546800	3.13921400
H	8.55190300	1.89577400	-1.48478800
H	8.63125900	3.24851800	-0.36068200
H	6.26969200	4.18158900	0.75262300
H	3.71445500	1.52482000	-1.29411700
H	8.41581900	3.27891200	-3.38930600
H	5.13254500	5.96586100	-2.47746100
H	6.65770100	4.14166600	-4.15323300
H	1.12542700	1.93145400	-2.46849300
H	1.36576000	3.52229100	-1.76370200
H	-0.25936800	2.82445000	-3.94779300
H	3.12216200	2.28170700	-3.55590400
H	3.40200900	4.52487500	-2.27166000
H	4.42799600	0.73461100	-6.23526700
H	5.98465700	-0.01911200	-6.38295100
H	6.80491000	2.39378300	-7.24332000
H	1.71977400	-7.99541000	-3.15748800
H	3.03873300	-8.67564800	-4.08864000
H	4.48532700	-6.74875800	-3.46873700
H	2.15853400	-5.92122100	-1.66313600
H	2.89758500	-4.95893100	-2.96071400
H	3.81667600	-5.30826200	-1.51637400
H	4.52630200	-8.90250300	-2.17953700
H	4.90234200	-7.51077400	-1.14692000
H	3.30497800	-8.25276900	-1.07140300
H	1.61968900	-7.57844900	-5.67728800
H	-1.33196400	-4.46575900	-4.15374500
H	0.06160000	-4.04110700	-3.15167800
H	-0.10592300	-6.86902500	-3.77538500
H	-3.15688100	-5.33781100	-0.22410900
H	-2.30379800	-4.04525700	0.64762600
H	-0.94972300	-3.67793700	-1.17748000
H	0.25255000	-8.07330600	0.84226200
H	-2.17515800	-7.35643200	-0.27507700
H	-2.53168400	-6.26096300	-7.61301800
H	-1.86800100	-6.04708600	-6.00125600
H	-3.75503300	-5.05525400	-4.00542800
H	-5.70734600	-3.50328700	-4.52346200
H	-6.52264600	-3.81216600	-6.93237500
H	-3.60439900	-8.39708100	-6.93626600
H	-11.47995200	5.12749700	-2.49508800
H	-11.58514900	3.63502300	-3.40690300
H	-11.98779400	3.90298400	-0.38701600
H	-12.07447300	2.37545200	-1.29376400
H	-14.06949200	3.47802100	-2.86553900
H	1.14074100	0.35617800	-4.60126800
H	1.65936100	-2.49468200	-3.56944500
H	2.90640200	-1.37128200	-4.06677600
H	1.18576500	4.49196600	0.34431800
H	3.17502100	1.20314100	1.89873000
H	-0.38645200	2.62161200	-0.07873600
H	-0.88200200	-1.16833900	-1.28022600
H	-5.58127300	3.85335100	-1.40783000
H	5.70879600	1.23478400	-3.02864400
H	2.59118000	-2.77322900	0.03470600
H	1.00021700	-3.51337900	0.35186000
H	1.72812900	-3.58498600	-1.26552000
H	3.84145100	3.24150500	0.52419400
H	-0.68925300	-1.85714900	-3.99593800

H	-13.95939500	5.04524800	-2.03365200
H	-13.55726400	4.92441300	-3.76911200
H	-5.50952500	4.84974000	4.76191500
H	-5.01955600	5.85684500	6.19077900
H	-4.48009800	2.02568300	3.76814300
H	-4.09926700	3.70120700	4.36240700
H	-1.50850600	7.81232400	5.37673500
H	-1.43016200	9.55687500	5.86699100
H	-5.05380600	4.56246600	-4.51191100
H	-3.52197100	5.09303600	-5.31711500
H	1.57390900	9.86410200	-4.54763600
H	1.69215700	8.95326100	-6.06828000
H	8.05663100	7.72883800	2.09632600
H	8.85614400	6.33850000	2.88640400
H	5.34379300	6.55856900	-4.12870300
H	2.10378200	3.23738300	-4.62299800
H	9.47065600	4.24064200	-2.32795300
H	5.08643400	2.61374000	-7.63238200
H	6.01586200	1.22103500	-8.32397100
H	-0.15769000	-1.52940200	-8.82546700
H	0.02519500	-3.19104700	-8.24110300
H	-5.21468700	0.04381200	-7.53890400
H	-4.86982900	-1.63533300	-8.04778800
H	-3.29692300	-8.02563400	-5.22153400
H	-1.92338200	-8.34482100	-6.30371700
H	3.09779200	-6.61544900	-5.53449200
H	8.85165400	-5.82738700	-4.46458300
H	8.93979000	-5.49392800	-2.72465900
H	-1.03586700	-9.13646200	0.22030500
H	-0.91073900	-8.74078600	1.97781800
H	2.93917500	-7.77317800	6.57961800
H	4.58320100	-8.14123600	7.22274700
H	9.72565800	-4.93201900	1.93925100
H	9.42810000	-5.24042100	3.65164400
H	11.00878200	0.77918000	4.46974800
H	11.01850300	2.39403900	3.66257400
H	-2.43948600	1.25509700	-0.60855700
H	-7.46038100	1.54893300	0.80562200
H	-8.70418900	-0.67716200	-0.24175600
H	-8.71978300	-2.11412400	1.56850700
H	-7.63018100	-5.72789300	4.69340000
H	-8.07576600	-6.14504200	3.01700400
H	-6.37433000	-5.80074100	3.41775500
H	-8.67682100	-3.78509300	3.56798700
H	-4.47469100	-2.10321200	1.81691700
H	-8.02826600	1.83198800	-1.34901300
H	-9.73608300	-3.41379600	8.38416500
H	-8.24538400	-3.87190800	9.16698000
H	0.14278300	-2.24800000	4.23621100
H	0.31124100	-3.68760000	3.23206700
H	-3.49266300	-3.35724100	3.83457600
H	-0.26496700	-1.35039200	1.28151800
H	-2.65089200	-0.96299900	0.46446200
H	-7.10928200	-3.34900500	5.08937600
H	0.25833000	-2.38162700	6.63833200
H	1.11566300	-1.96902500	9.13121800
H	4.11064200	-2.04028000	8.44998400
H	3.35123600	-1.02451100	9.69289600
H	3.37574900	-2.79261000	9.87391200
H	-10.14032500	-5.20043300	10.15058300
H	-9.71081400	1.49808500	3.66982200
H	-8.93200400	2.72001700	2.65481900
H	-7.94636000	1.53597600	3.57700700
H	-10.56496000	-5.80472600	8.53144600
H	-9.04405800	-6.28972700	9.25974000
H	-7.84996800	-4.52294900	1.27995100
H	-6.27250600	-3.83826900	1.69228300
H	-5.84420300	-0.01690800	-0.98242900
H	-7.21338600	-0.32821200	-2.04228300
H	2.67783400	-3.51795200	7.00458200
H	-1.24026400	-4.96486700	4.61524400
H	1.78798200	-4.20724800	8.34983200
H	-1.69290400	-3.52296100	5.54600900
H	2.48511500	-0.93514800	6.90406900
H	2.55387700	-1.10984600	1.42463700

Int6 (+2.4)

C	-8.85536300	1.74130700	2.97645600
C	-8.84796400	0.64935600	1.93732800
O	-9.61575600	-0.31479800	1.99139800
N	-7.81804000	0.70843400	1.05036700
C	-7.70824000	-0.31982600	0.03186400
C	-7.00975700	-1.54104900	0.66749100
O	-5.76709100	-1.70906500	0.53902500
C	-6.91975500	0.25773000	-1.17419100
O	-7.03947900	1.65695300	-1.26037600
N	-7.78142000	-2.38274000	1.34610000
C	-7.34485400	-3.69203900	1.83917300
C	-7.58025800	-3.98957900	3.32360800
C	-7.41416200	-5.49457400	3.56391500
O	-6.67833100	-3.24126400	4.10967000
C	-9.82519400	-5.44954000	9.05311100
C	-9.08140100	-4.26512600	8.41438000
C	-8.38805400	-4.55081800	7.04420300
O	-8.41503100	-5.72720800	6.61955500
O	-7.85588600	-3.52694500	6.50555400
C	-1.16026800	-4.00627400	5.20081000
C	-0.00745100	-4.38449500	6.13028600
O	0.45972500	-5.51839700	6.19378000
C	-0.65104500	-3.23096200	3.97248800
C	-1.74485500	-2.69669100	3.10411400
C	-3.11483400	-2.81735300	3.12727300
N	-1.45516600	-1.89257600	2.00936600
C	-2.58795300	-1.52961400	1.39799200
N	-3.60368100	-2.08711800	2.05721700
N	0.48383500	-3.35712900	6.88068900
C	1.73618500	-3.46474100	7.60685300
C	1.99261900	-2.16249100	8.36766700
C	3.37331200	-2.14426900	9.01349900
O	1.79472600	-1.03311400	7.51106400
N	-4.57875500	3.42160200	-1.46213300
N	-2.96520800	2.08118700	-0.92362000
N	-3.75511900	-0.15167900	-5.20831800
N	-4.09666900	-2.37991800	-1.54751400
N	-0.40990400	-0.85780000	-4.29704100
N	0.25583700	-1.63470800	-6.79345200
N	7.05130300	3.85279100	6.07713700
N	5.84112000	1.84152000	-1.54852900
N	4.68177900	2.95637700	-0.03273300

N	6.66812700	4.90510900	-3.45469500
N	3.33614100	4.34568300	-3.21315300
N	0.15655700	-5.88824600	-4.07366700
N	-1.48963800	-4.55736900	-1.21340300
N	-1.73824100	-7.23699400	0.48163400
N	-4.86275900	-5.00105000	-7.21745600
N	-5.33799800	-4.06321800	-5.25586000
C	-3.89537600	4.55515700	-4.51198000
C	-3.37697200	4.98198600	-3.12599600
C	-3.44953800	3.87441300	-2.13403500
C	-2.44334800	3.01485100	-1.79044200
C	-4.25807300	2.34713900	-0.72873300
C	-4.96923200	-0.84398500	-7.22638300
C	-3.70371800	-0.88019800	-6.36551700
C	-2.82150900	-0.38911600	-4.10352600
C	-1.37889100	0.07754800	-4.43481300
C	-2.93678600	-1.84383900	-3.59344900
C	-4.23970300	-2.03909000	-2.84313400
C	0.98457500	-0.67771500	-4.65158100
C	1.29501400	-1.12157000	-6.09780100
C	1.91087100	-1.44856900	-3.69473500
C	0.90151100	-1.67605300	-0.75414100
C	0.40657900	-2.08591800	-8.15637700
C	1.40423800	8.97177200	-4.96297500
C	1.92897400	7.69916500	-4.27413600
C	1.26873400	7.31662900	-2.94373900
C	-0.17660700	6.85855500	-3.08181100
C	-1.51315600	8.81429800	5.11860900
C	-0.39589600	9.13426800	4.09402800
C	-0.12247500	8.09805100	3.02832900
C	-1.02620500	7.84943000	1.98646600
C	1.07789300	7.37668300	3.03132200
C	-0.75193600	6.91260300	0.99417000
C	1.37455900	6.44219100	2.04105300
C	0.45429700	6.19591300	1.01689700
C	-5.65593400	5.76346100	5.36374700
C	-5.50842800	6.92590600	4.35158300
C	-5.91528800	6.46978000	2.96359600
C	-7.23621100	6.10548600	2.66559100
C	-4.95510700	6.27681900	1.96353400
C	-7.58994000	5.54877100	1.43713200
C	-5.28861200	5.73485400	0.72387000
C	-6.60896400	5.34411200	0.45117800
C	-3.76962900	2.87287000	3.71878000
C	-2.40596600	2.33470300	4.19265100
C	-1.38749800	3.45614700	4.44508500
C	-1.88550500	1.25855400	3.23568500
C	-1.04958100	4.30774300	3.22488100
C	3.89782200	-8.00464000	6.25031000
C	4.49868500	-6.73167400	5.62020100
C	4.06859400	-6.48974900	4.19156800
C	4.99509700	-6.56832300	3.14475500
C	2.73152600	-6.20318300	3.88488200
C	4.60443000	-6.34508800	1.82277300
C	2.33479800	-5.99109200	2.56444800
C	3.27284200	-6.05109500	1.53073300
C	11.02027900	1.15984200	3.55480000
C	9.86635700	0.73313600	2.63404200
C	8.46773900	1.23787900	3.03408700
C	8.37554500	2.76607800	2.95271500

C	7.38432700	0.59653300	2.15525600
C	7.95527500	6.66740500	2.69740700
C	7.09066800	6.89116700	3.93323100
C	6.81530900	5.62544600	4.69360000
C	7.58470400	5.07248100	5.68668700
C	5.74253700	4.69357300	4.44547000
C	5.92446400	3.58930200	5.32453300
C	4.65537300	4.67841400	3.55557800
C	5.06676700	2.48545700	5.32329700
C	3.78991900	3.59225000	3.55742400
C	4.00218100	2.50354300	4.42919900
C	8.44608400	-5.22432500	-3.70007500
C	6.91365600	-5.30128800	-3.61391700
C	6.32302700	-4.29794800	-2.65300700
C	5.82706400	-3.07575800	-3.12025400
C	6.28841900	-4.53298700	-1.27238700
C	5.30975800	-2.11713400	-2.25407900
C	5.75469000	-3.59613900	-0.38984500
C	5.26292100	-2.38340800	-0.88244300
C	9.32926100	-4.59801200	2.84820800
C	7.91372400	-4.03259000	2.67377000
C	7.22904200	-3.54285300	3.96274600
C	8.04820600	-2.46801200	4.68898300
C	5.81361000	-3.03373500	3.65386400
C	8.51624200	3.77535500	-2.39558000
C	7.53636600	4.94043700	-2.40880100
C	8.18027600	2.81723500	-1.22014800
C	6.70838700	2.69912300	-0.89047000
C	5.98738400	3.39595300	0.05956300
C	4.64134000	2.01992800	-1.00931000
C	5.41827100	5.63969500	-3.43776900
C	4.35699900	4.71414000	-4.03474800
C	2.53118100	3.18391100	-3.56560800
C	1.29435300	3.00056400	-2.68614300
C	5.92287200	1.88813500	-7.42465500
C	5.53665300	0.97104800	-6.25747500
C	6.00144400	1.46211000	-4.90876100
C	2.14622000	-6.89650900	-5.08004900
C	1.31470600	-5.65093800	-4.76098700
C	2.54031000	-7.72144900	-3.83675200
C	3.55682300	-7.04589300	-2.89656000
C	3.03345500	-5.75686200	-2.25057000
C	4.01143900	-8.03252400	-1.81450200
C	-0.61106400	-4.81456900	-3.47506200
C	-1.40035700	-5.36581100	-2.28855700
C	-2.26553900	-4.93035500	-0.03372400
C	-1.51424800	-5.95037400	0.84248300
C	-0.88197200	-8.36254100	0.85358300
C	-2.97265600	-7.80578800	-6.34663000
C	-2.78043800	-6.30219400	-6.63803900
C	-3.92851500	-5.40874600	-6.28321000
C	-4.21021800	-4.83574000	-5.06114800
C	-5.67937500	-4.19137200	-6.56879000
C	1.52974000	2.10697300	0.89721100
C	2.32097800	1.01584600	1.37407200
C	1.98013700	-0.27943500	1.04213000
C	0.25798800	1.80240600	0.35260900
C	-0.10162300	0.48824200	0.03205700
C	0.80558400	-0.59643700	0.31282800
C	-13.44180700	4.57851400	-2.81880200

C	-11.94439000	4.30097500	-2.55511800
C	-11.58505500	3.48655500	-1.30070200
C	-10.05507400	3.31626500	-1.18382600
C	1.59183300	-2.97505100	-0.37337400
O	-2.70293800	-1.52260200	-6.68426900
O	-1.16821800	1.24616800	-4.75249300
O	-5.34694900	-1.88113100	-3.37760500
O	2.44197100	-1.01159400	-6.52907400
O	-0.88716500	7.12942400	-4.03058500
O	-0.67834900	6.16762800	-2.05025600
O	0.70681900	5.28112300	0.03893300
O	-6.89497100	4.78270100	-0.75130100
O	4.74544000	-1.49555000	0.01698000
O	7.49362500	5.78424000	-1.51317100
O	4.46539500	4.30610900	-5.18742000
O	0.12518300	3.53279600	-3.30565400
O	6.88396600	2.30115800	-4.75811600
O	5.40963300	0.83338200	-3.90262300
O	1.66274600	-4.51221000	-5.05972300
O	-1.93083000	-6.48401300	-2.35605800
O	-0.77200000	-5.59206600	1.75467700
O	2.00979100	3.33031300	0.93169900
O	-1.25107600	0.20113200	-0.59823800
O	-9.57868200	2.16853000	-1.39654000
O	-9.40314200	4.37322000	-0.91690100
O	-0.35035600	-1.99186600	-1.32733700
S	2.00917100	-0.70187500	-2.03085000
H	-3.92761200	5.86067700	-2.77219900
H	-2.33375900	5.27958300	-3.18741000
H	-1.41615200	3.01174000	-2.12196000
H	-4.94368100	1.81149200	-0.09841500
H	-3.62695700	3.51636900	-4.72780100
H	-5.82366600	-1.18087400	-6.63227700
H	-2.90281000	-2.52277800	-4.44943700
H	-2.10837700	-2.07553000	-2.92421700
H	-4.88660600	-2.29925000	-0.90264600
H	-3.17205400	-2.41421300	-1.14990800
H	-3.12397100	0.29435600	-3.30440900
H	-4.66974900	0.16176600	-4.91496500
H	1.46929900	-2.06222700	-8.40012500
H	-0.68772200	-1.58323200	-6.42732000
H	1.80587800	6.85303300	-4.95872900
H	3.00870000	7.79544900	-4.10307300
H	1.27221000	8.15834700	-2.23653700
H	1.83899700	6.51382700	-2.46543500
H	0.31628900	8.93632100	-5.03447100
H	-0.62711000	10.09822900	3.62060200
H	0.53504300	9.29280100	4.65119100
H	1.80108100	7.54944400	3.82509200
H	-1.96508400	8.39749200	1.94795900
H	2.30233700	5.88230500	2.06534500
H	-1.46166400	6.72271900	0.19738500
H	-0.01149600	5.81389900	-1.41052100
H	-2.51177900	8.97223200	4.70073300
H	-6.09541100	7.79565700	4.67424600
H	-4.46241100	7.25070800	4.31923400
H	-8.01001600	6.24612000	3.41755000
H	-3.91898900	6.54632700	2.15678300
H	-8.61705200	5.27905200	1.21332200
H	-4.52403400	5.61275500	-0.03469200
H	-7.89582900	4.53960600	-0.83941600
H	-6.68088200	5.69689600	5.74352000
H	-2.58125200	1.84823700	5.16403100
H	-1.74371900	1.64876800	2.22415300
H	-0.91909700	0.85601700	3.56026700
H	-2.59818700	0.42860300	3.18312700
H	-1.78654200	4.10640200	5.23660600
H	-0.46459000	3.01693700	4.84788900
H	-1.94467600	4.74867500	2.77672400
H	-0.38960500	5.12997600	3.50147100
H	-0.53938900	3.72791900	2.45146900
H	-3.73597300	3.25246500	2.69279000
H	4.22499800	-5.86750400	6.23969700
H	5.59277100	-6.79761700	5.65077300
H	6.03521300	-6.79219100	3.36999500
H	1.99511000	-6.14360700	4.68120300
H	5.33830000	-6.39448400	1.02387300
H	1.29140500	-5.79856900	2.33994700
H	2.96659800	-5.87040600	0.50722100
H	3.97726700	-8.84540400	5.55314600
H	10.06969500	1.08754900	1.61384300
H	9.85009400	-0.36314700	2.57285300
H	8.28148700	0.93854600	4.07788300
H	7.35741000	3.10560800	3.14692000
H	8.65411200	3.11240500	1.95068600
H	9.02853700	3.26630200	3.67354100
H	6.38579800	0.91283300	2.47271200
H	7.49848400	0.89359400	1.10802100
H	7.42194800	-0.49675700	2.19421500
H	11.97929800	0.90692300	3.08571200
H	6.14022300	7.35338200	3.63969800
H	7.58649800	7.61320600	4.59470900
H	8.48245600	5.45244900	6.15373000
H	7.45917500	3.22702800	6.75024500
H	4.49564600	5.50806500	2.87196800
H	2.95396300	3.56892000	2.86648700
H	5.22959400	1.64469800	5.99198300
H	3.31467100	1.66421900	4.39302400
H	7.47298600	5.99966400	1.97770800
H	6.48656500	-5.13432500	-4.60952100
H	6.62613000	-6.31863200	-3.32013000
H	6.67019900	-5.47367100	-0.88034600
H	5.84340500	-2.86139400	-4.18566600
H	5.69378100	-3.79357900	0.67337600
H	4.95154100	-1.17198500	-2.64311300
H	4.22626600	-0.83117500	-0.46569800
H	8.77622600	-4.20040500	-3.89795900
H	7.94092200	-3.20740300	1.94968600
H	7.27412400	-4.80171300	2.22465700
H	7.13682600	-4.40990600	4.63560100
H	8.19419600	-1.59196400	4.04846600
H	7.53093000	-2.13064000	5.59418000
H	9.03542500	-2.83399600	4.98817800
H	5.83942100	-2.19450100	2.94978000
H	5.31345200	-2.68772500	4.56626200
H	5.19526700	-3.82137000	3.21538900
H	10.04837300	-3.82105800	3.12560200
H	8.57810500	1.82250000	-1.44292300
H	8.67024600	3.16402600	-0.30590000
H	6.28914100	4.14884600	0.76802600

H	3.73680900	1.50227200	-1.29249600
H	8.46721500	3.22522300	-3.33538200
H	5.20790400	5.93921500	-2.40714900
H	6.71796000	4.11439600	-4.09498100
H	1.16311100	1.93728200	-2.46029300
H	1.41206400	3.51434800	-1.72862000
H	-0.19880200	2.87151500	-3.94260000
H	3.16758300	2.28911000	-3.53350600
H	3.45258700	4.52077500	-2.22332200
H	4.46371900	0.76195300	-6.21710500
H	6.00734600	-0.01565700	-6.37475600
H	6.86190200	2.40027200	-7.20424700
H	1.64579500	-7.99489400	-3.26007800
H	2.96971200	-8.66835700	-4.18794500
H	4.43318200	-6.78011000	-3.50298900
H	2.08629000	-5.94337600	-1.72920300
H	2.87675600	-4.96535500	-2.98367700
H	3.75388500	-5.37298200	-1.52599100
H	4.40756900	-8.95796300	-2.24798500
H	4.79066100	-7.58895600	-1.18743100
H	3.17761100	-8.29990200	-1.15415700
H	1.56787000	-7.52974600	-5.76517500
H	-1.32831400	-4.38643400	-4.18999800
H	0.06324100	-4.01447100	-3.16553100
H	-0.14959400	-6.82448800	-3.84848100
H	-3.23723700	-5.32123300	-0.34857600
H	-2.42036500	-4.03753200	0.56779400
H	-0.98347900	-3.66286000	-1.19987800
H	0.17452800	-8.08753300	0.74719900
H	-2.18535000	-7.32495900	-0.42609000
H	-2.55527400	-6.15705100	-7.69970600
H	-1.89572900	-5.96770800	-6.08312700
H	-3.75129400	-4.94868700	-4.09111600
H	-5.68782700	-3.37500900	-4.59171600
H	-6.52749700	-3.67565400	-6.99655800
H	-3.65699300	-8.28648100	-7.04983300
H	-11.40741100	5.25441900	-2.49719100
H	-11.53515100	3.77573700	-3.42845600
H	-11.94515100	4.01060000	-0.40676900
H	-12.05059600	2.49683000	-1.33435000
H	-14.02397400	3.65377100	-2.89193600
H	1.20520400	0.39032700	-4.59892600
H	1.62608500	-2.50282500	-3.64862500
H	2.91627600	-1.41757300	-4.11805400
H	1.24182200	4.45702500	0.40255700
H	3.25091200	1.22612600	1.88997300
H	-0.38686300	2.62406300	0.06138400
H	-0.88675000	-1.12591400	-1.19788200
H	-5.51604400	3.85921300	-1.41547600
H	5.75336900	1.19665900	-3.00374300
H	2.59076500	-2.80373800	0.02288200
H	0.99255500	-3.51139600	0.37258600
H	1.69031800	-3.61008000	-1.25619600
H	3.84837000	3.23489900	0.50994200
H	-0.67001400	-1.78927900	-4.02053100
H	-13.88825800	5.21008000	-2.04378000
H	-13.48692300	5.10026200	-3.78002300
H	-5.47342500	4.85481900	4.77998700
H	-4.97885500	5.84190500	6.22118200
H	-4.46871100	2.03103300	3.76049700

H	-4.07329400	3.69629900	4.37375200
H	-1.44512500	7.77012300	5.44094800
H	-1.35108200	9.50856200	5.94978300
H	-4.98473300	4.65998000	-4.49456200
H	-3.44451900	5.18340600	-5.28837000
H	1.69623100	9.89429400	-4.44966500
H	1.81116300	8.99826200	-5.97933400
H	8.13131200	7.62407100	2.19587100
H	8.91368600	6.21751700	2.97431600
H	5.43091500	6.54653300	-4.05127500
H	2.15949300	3.26369700	-4.59261100
H	9.53008200	4.16506600	-2.26205600
H	5.14738200	2.64142600	-7.59715200
H	6.06530200	1.24664200	-8.29983300
H	-0.13368000	-1.43571100	-8.85338600
H	0.03017000	-3.10514600	-8.28581800
H	-5.17948100	0.17510800	-7.56942200
H	-4.84961900	-1.50202900	-8.09460400
H	-3.35222600	-7.93716100	-5.32970700
H	-1.97780200	-8.25890200	-6.41000800
H	3.05757500	-6.58862200	-5.60371800
H	8.81488300	-5.87010000	-4.50398300
H	8.89965600	-5.55582700	-2.76033200
H	-1.12358000	-9.12768100	0.10863100
H	-1.00127800	-8.75172700	1.87066000
H	2.84036600	-7.87137900	6.49683800
H	4.47810300	-8.26276800	7.14223600
H	9.67310600	-5.05070200	1.91215100
H	9.36584800	-5.37397100	3.62009400
H	11.00398200	0.62040400	4.50728200
H	11.03310800	2.24343900	3.71715000
H	-2.34546400	1.26345800	-0.62208200
H	-7.45923000	1.61438100	0.76237800
H	-8.71737300	-0.61225000	-0.27645400
H	-8.72669100	-2.05030500	1.54077900
H	-7.61417000	-5.71438700	4.61992000
H	-8.10945800	-6.08001900	2.94772300
H	-6.38915300	-5.79843500	3.31286900
H	-8.61964300	-3.71401900	3.57318000
H	-4.58983700	-2.00433500	1.73513500
H	-8.01417400	1.91040600	-1.38145500
H	-9.76137700	-3.41677300	8.26393300
H	-8.30409600	-3.89252500	9.09575000
H	-0.02453400	-2.38774600	4.29060700
H	-0.02152900	-3.89076600	3.36693000
H	-3.79693200	-3.33343200	3.78745300
H	-0.53271500	-1.54938000	1.67757000
H	-2.60821200	-0.86455600	0.54597300
H	-7.02174000	-3.36064800	5.05260700
H	0.18810000	-2.40831400	6.68863000
H	1.22102800	-2.04978800	9.13774200
H	4.15690100	-2.23890100	8.25166800
H	3.52655700	-1.20492900	9.55033200
H	3.49110800	-2.97423900	9.71760100
H	-10.25321100	-5.17879500	10.02705200
H	-9.70295900	1.55919000	3.63902200
H	-8.90903700	2.78203300	2.63777300
H	-7.93896300	1.57849600	3.55117600
H	-10.63365800	-5.79469800	8.40110600
H	-9.14662900	-6.29681300	9.18698800

H	-7.87677000	-4.44759800	1.24801100
H	-6.28055400	-3.78703200	1.61607800
H	-5.85962000	0.02466200	-1.04291500
H	-7.24785300	-0.25379600	-2.08737600
H	2.56727100	-3.65606300	6.90784700
H	-1.64102400	-4.93259300	4.88207300
H	1.71840000	-4.31269100	8.30004200
H	-1.89355900	-3.40311100	5.74859100
H	2.49558500	-1.04280300	6.84256600
H	2.68301300	-1.07249100	1.27736700

Int7 (-0.1)

C	-8.78019800	1.95554300	2.90536800
C	-8.93108100	0.79949500	1.94861200
O	-9.90062000	0.03381400	2.02596400
N	-7.88203000	0.56699000	1.12282200
C	-7.96934900	-0.41240900	0.04121400
C	-7.66727500	-1.85190700	0.52332100
O	-6.85646000	-2.59280100	-0.05370200
C	-7.00919100	0.04862600	-1.07560800
O	-6.99585600	1.45424900	-1.19170300
N	-8.39521500	-2.24948800	1.58167600
C	-8.46901800	-3.61204000	2.09013300
C	-7.38215100	-4.02682800	3.11745800
C	-6.12168000	-4.58124800	2.45342000
O	-7.09726800	-2.96778500	4.03128100
C	-9.80131400	-4.89504400	9.35607000
C	-8.89380600	-3.84257100	8.70316700
C	-8.61814400	-4.04235700	7.21261700
O	-9.05582200	-4.95993600	6.54479400
O	-7.82670600	-3.06808200	6.74531400
C	-1.13991300	-2.51317500	5.39065100
C	0.04699500	-3.22736600	6.03686000
O	0.54335200	-4.23690400	5.55276100
C	-1.12430800	-2.53555200	3.84687000
C	-2.46169500	-2.12879600	3.28980400
C	-3.73122000	-2.26585100	3.80916500
N	-2.65493100	-1.50974600	2.05994400
C	-3.99608400	-1.28560200	1.91780800
N	-4.67989400	-1.73900900	2.95409500
N	0.47607800	-2.71083700	7.23994800
C	1.77429400	-3.09424800	7.78761800
C	2.46253100	-1.88373300	8.43738800
C	3.92909200	-2.18437700	8.73584200
O	2.31365700	-0.70544700	7.64846300
N	-4.48420600	3.21626600	-1.68193500
N	-2.88266800	1.91803400	-1.00052700
N	-3.74079000	-0.36551600	-5.18865800
N	-4.42747000	-2.34374900	-1.42111800
N	-0.45283700	-1.10407800	-4.22137000
N	0.25268800	-2.02060600	-6.68425800
N	7.09656800	4.06223700	5.83434500
N	5.90654700	1.69164100	-1.64755500
N	4.74937700	2.92778300	-0.22755500
N	6.75821100	4.63219700	-3.70944500
N	3.42405900	4.11716200	-3.43436800
N	0.14548900	-6.11459600	-3.74313800
N	-1.48747700	-4.59723400	-0.95916800
N	-1.75247500	-7.20139300	0.89991800
N	-4.89367400	-5.39885400	-6.92870500
N	-5.32866900	-4.28205300	-5.05282800
C	-3.81019600	4.32430000	-4.72955800
C	-3.23925200	4.72744100	-3.35862800
C	-3.33334500	3.63942300	-2.33905800
C	-2.33596600	2.80730100	-1.90350700
C	-4.18185500	2.18994100	-0.87338900
C	-4.93852000	-1.20128000	-7.15266100
C	-3.68653000	-1.20300400	-6.26909800
C	-2.83857400	-0.49502300	-4.03933900
C	-1.37487300	-0.12882700	-4.40397700
C	-2.99915100	-1.85615400	-3.32824700
C	-4.38424600	-2.03628000	-2.72925700
C	0.95330100	-0.99113700	-4.56244500
C	1.28616800	-1.50919000	-5.98014900
C	1.83869900	-1.72446500	-3.54107600
C	0.87132100	-1.72333000	-0.51588100
C	0.42417900	-2.53934200	-8.02392300
C	1.52727100	8.66284500	-5.42215200
C	2.02637500	7.41770600	-4.66706800
C	1.35442700	7.11310200	-3.32226100
C	-0.10524200	6.69314000	-3.43654500
C	-1.37030700	9.06512000	4.65856300
C	-0.26070100	9.31631200	3.60880500
C	-0.02267800	8.22216500	2.59434300
C	-0.96429700	7.91108700	1.60372300
C	1.17940400	7.50405900	2.59458000
C	-0.72403600	6.91768200	0.65921600
C	1.44182200	6.51293100	1.65137400
C	0.48480300	6.20243400	0.67776400
C	-5.53944900	6.06897400	5.07153600
C	-5.38050000	7.15933500	3.98064400
C	-5.78691800	6.60536600	2.62681400
C	-7.11919500	6.28601000	2.32812000
C	-4.81834700	6.26797500	1.67297600
C	-7.47784800	5.63969000	1.14495800
C	-5.15555600	5.63206400	0.47969000
C	-6.49035800	5.29294400	0.20574100
C	-3.68266100	3.07855400	3.57882500
C	-2.34313500	2.58593300	4.16749400
C	-1.32692300	3.72435900	4.34335400
C	-1.78698400	1.39769000	3.38379300
C	-0.86838200	4.38952500	3.04948100
C	3.89249900	-7.71846200	6.67003700
C	4.54069800	-6.49184600	5.99114300
C	4.16238500	-6.28726900	4.54285100
C	5.06360300	-6.60184100	3.51782100
C	2.90420500	-5.77965800	4.20041300
C	4.72032300	-6.39833900	2.17988800
C	2.55285100	-5.58268100	2.86489800
C	3.46440300	-5.88577200	1.85105000
C	11.09110800	1.22568500	3.48169000
C	9.91753300	0.76888200	2.60006000
C	8.53889700	1.33858700	2.98276300
C	8.48583200	2.85934200	2.79242200
C	7.42205800	0.66608100	2.17192300
C	8.07355800	6.70760800	2.33902000
C	7.21374400	7.00434600	3.56268700
C	6.90785700	5.77984500	4.37666100
C	7.65999700	5.25205400	5.39624400

C	5.81521700	4.86264100	4.16105400
C	5.96752800	3.79250200	5.08601100
C	4.73135400	4.83594400	3.26693000
C	5.08364400	2.70983200	5.12693100
C	3.84172100	3.77065100	3.30881100
C	4.02456200	2.71461900	4.22633700
C	8.44487800	-5.50959600	-3.42133200
C	6.91306500	-5.59657500	-3.33327000
C	6.30788900	-4.52323100	-2.46394500
C	5.78517500	-3.36176600	-3.04313700
C	6.29421500	-4.62510200	-1.06701800
C	5.26227200	-2.33263200	-2.26780900
C	5.75328000	-3.61629900	-0.27256300
C	5.23493900	-2.46527100	-0.87567200
C	9.34745600	-4.54598100	3.08364500
C	7.92981400	-3.99485500	2.87310000
C	7.22757700	-3.45618200	4.13234400
C	8.03526200	-2.35136000	4.82576800
C	5.81552900	-2.96495300	3.78368200
C	8.60212200	3.54436700	-2.59601300
C	7.63605300	4.72003900	-2.67419500
C	8.25580600	2.65385300	-1.36971900
C	6.78098800	2.57900900	-1.04044900
C	6.06174200	3.35139600	-0.14929900
C	4.70490800	1.92462700	-1.13498900
C	5.51479500	5.37970600	-3.72909500
C	4.44309000	4.43570900	-4.27753500
C	2.60540200	2.94678800	-3.72202800
C	1.37858000	2.82220700	-2.81934800
C	5.97717100	1.41798300	-7.51271100
C	5.58836400	0.56868600	-6.29611700
C	6.06279700	1.13010700	-4.97788900
C	2.12732600	-7.19536900	-4.70045200
C	1.28806600	-5.93105400	-4.47356400
C	2.53779600	-7.90923900	-3.39424100
C	3.55157500	-7.13628600	-2.52922600
C	2.99917500	-5.82522800	-1.95562500
C	4.07528300	-8.02685700	-1.39672600
C	-0.60698600	-5.00055700	-3.19804600
C	-1.38988300	-5.47833800	-1.97280800
C	-2.21432200	-4.89897100	0.27252900
C	-1.44150800	-5.90112200	1.15437200
C	-0.90073100	-8.31820900	1.30746300
C	-3.00222000	-8.12491900	-5.90930600
C	-2.79534700	-6.63894400	-6.27467100
C	-3.93875500	-5.71757500	-5.98053600
C	-4.19612600	-5.03267700	-4.81181700
C	-5.69745300	-4.53174700	-6.34000900
C	1.60458000	2.16238800	0.80348900
C	2.45598700	1.12458900	1.26953500
C	2.11435000	-0.19909700	1.04428600
C	0.28100300	1.80404100	0.43994500
C	-0.08934400	0.46119000	0.28905200
C	0.89121700	-0.56524600	0.45414500
C	-13.35303300	4.52328800	-3.02432500
C	-11.85771500	4.24117100	-2.74456000
C	-11.50969000	3.46765900	-1.46114300
C	-9.98058700	3.29249000	-1.30983800
C	1.55672600	-3.01330000	-0.10261900
O	-2.70189100	-1.90083600	-6.51053700

O	-1.10534100	1.00609200	-4.79288100
O	-5.41043700	-1.92345000	-3.42062400
O	2.44574300	-1.44229100	-6.38660300
O	-0.80407200	6.92326100	-4.40558700
O	-0.63096000	6.09233600	-2.36341000
O	0.70209000	5.23186300	-0.24654300
O	-6.78495600	4.65117200	-0.95255900
O	4.71390900	-1.49782100	-0.07047400
O	7.61345900	5.61959600	-1.83371600
O	4.54285300	3.97363000	-5.41074800
O	0.21511900	3.37800000	-3.43143300
O	6.95795400	1.96464600	-4.87994100
O	5.46451400	0.56995800	-3.93847900
O	1.62222400	-4.82107300	-4.87626100
O	-1.90274800	-6.60770900	-1.96492600
O	-0.61277700	-5.53704000	1.98241000
O	2.07225600	3.39820500	0.69971200
O	-1.34231400	0.08247000	-0.06100400
O	-9.51884700	2.12027000	-1.35921600
O	-9.31561000	4.36547800	-1.17302200
O	-0.41178100	-2.02824100	-1.00434800
S	1.94175100	-0.84883600	-1.93605900
H	-3.73000800	5.63702900	-2.99425400
H	-2.18479700	4.97453800	-3.44932500
H	-1.29418300	2.81091000	-2.18292400
H	-4.89730100	1.69206100	-0.24507000
H	-3.57713000	3.27327100	-4.93647500
H	-5.79989000	-1.49927400	-6.54704600
H	-2.85708700	-2.65276200	-4.06654600
H	-2.23814400	-1.95483200	-2.55192000
H	-5.33943500	-2.45235500	-0.95797200
H	-3.58297300	-2.39102200	-0.87332300
H	-3.12818600	0.30162900	-3.34780900
H	-4.66195600	-0.03336300	-4.93955800
H	1.48984200	-2.53194100	-8.25357700
H	-0.69521900	-1.95424200	-6.33019700
H	1.88929700	6.54122400	-5.30954200
H	3.10726600	7.50142200	-4.49653500
H	1.38556800	7.98294500	-2.65053800
H	1.90036700	6.31376300	-2.81048400
H	0.43877200	8.63964400	-5.49373000
H	-0.48148400	10.26076200	3.09219300
H	0.68002100	9.48476600	4.14647800
H	1.93099600	7.72389500	3.34960200
H	-1.90607900	8.45466300	1.56820100
H	2.37176600	5.95663900	1.67331100
H	-1.46103200	6.68148600	-0.09982200
H	0.02155900	5.73691700	-1.70599700
H	-2.37078700	9.20897200	4.23942700
H	-5.96199600	8.05441800	4.23682600
H	-4.33155400	7.47177400	3.92906300
H	-7.90001000	6.53736500	3.04356300
H	-3.77270300	6.49693400	1.86662900
H	-8.51392100	5.40784700	0.91950500
H	-4.38256300	5.39656300	-0.24292700
H	-7.79514500	4.46541400	-1.04936100
H	-6.56594500	6.03742300	5.45233600
H	-2.57485100	2.22377900	5.18173100
H	-1.64711900	1.63332400	2.32641500
H	-0.81287800	1.07634400	3.77117900

H	-2.47399700	0.55189200	3.45205500
H	-1.77193600	4.48495200	5.00172400
H	-0.44976700	3.33450700	4.87821700
H	-1.71183100	4.76978900	2.46582100
H	-0.21783200	5.23843100	3.26180500
H	-0.30861100	3.69893600	2.41433400
H	-3.60835900	3.38895500	2.53353200
H	4.28768300	-5.59281700	6.56803500
H	5.63044500	-6.59806800	6.05551800
H	6.04771100	-6.98988800	3.77142700
H	2.19250500	-5.51510900	4.97435600
H	5.43483600	-6.63000600	1.39519900
H	1.56639400	-5.20416300	2.62354600
H	3.20029900	-5.71737200	0.81395500
H	3.96114600	-8.59410100	6.01613400
H	10.11755200	1.04972600	1.55656800
H	9.87076700	-0.32808700	2.61230900
H	8.36356300	1.12041300	4.04852100
H	7.47743900	3.23816900	2.96295800
H	8.77046400	3.12575600	1.76796600
H	9.15351200	3.39313800	3.47483900
H	6.43920800	1.03045100	2.48683000
H	7.52007600	0.88410300	1.10381200
H	7.43362800	-0.42237800	2.28928900
H	12.04209800	0.93547800	3.01837000
H	6.27401400	7.47392200	3.24700900
H	7.72501000	7.74411000	4.19232200
H	8.56502700	5.62986400	5.85094500
H	7.48918300	3.45307600	6.53125000
H	4.59250900	5.64014600	2.54904700
H	3.01045500	3.73555800	2.61267600
H	5.22184100	1.89455300	5.83176900
H	3.32038100	1.88890900	4.21607000
H	7.58321400	6.00679300	1.65737300
H	6.48810900	-5.51802700	-4.34073500
H	6.63850600	-6.59091400	-2.95872200
H	6.69663700	-5.51698100	-0.59065800
H	5.78722100	-3.25372700	-4.12468600
H	5.70550600	-3.71205100	0.80559900
H	4.89699900	-1.42727600	-2.73775900
H	4.10087100	-0.95596100	-0.60131000
H	8.77103400	-4.49720700	-3.67478100
H	7.96003600	-3.20108400	2.11460700
H	7.29801600	-4.78628500	2.45252300
H	7.12739500	-4.29675900	4.83692700
H	8.19373500	-1.50366000	4.15043000
H	7.50373700	-1.97456000	5.70699500
H	9.01733800	-2.70495700	5.15613400
H	5.85165600	-2.14039200	3.06252100
H	5.29347200	-2.60167200	4.67695200
H	5.21021000	-3.76530900	3.35083000
H	10.06669100	-3.75576400	3.31951000
H	8.63800700	1.64284000	-1.53893100
H	8.75103500	3.04382100	-0.47584000
H	6.36773000	4.14812600	0.50748800
H	3.79660400	1.39493800	-1.38085500
H	8.54539300	2.94443400	-3.50447700
H	5.30814100	5.73407600	-2.71529400
H	6.79634800	3.80397500	-4.30167700
H	1.21969600	1.76775700	-2.56894600

H	1.52838400	3.34953500	-1.87365100
H	-0.13186600	2.71485600	-4.05330400
H	3.23355100	2.04877700	-3.64718600
H	3.55038400	4.33679100	-2.45459500
H	4.51411800	0.37042100	-6.23873100
H	6.05184000	-0.42616100	-6.36114800
H	6.92104400	1.93331400	-7.32210700
H	1.65268200	-8.14642000	-2.78820200
H	2.98085100	-8.87542800	-3.66835900
H	4.39999100	-6.88160600	-3.17805400
H	2.07822500	-6.00824200	-1.38877700
H	2.79072600	-5.09229000	-2.73477900
H	3.72905500	-5.36635500	-1.28652600
H	4.48681300	-8.96925100	-1.77662600
H	4.86201100	-7.51323200	-0.83622400
H	3.27553700	-8.26916900	-0.68659400
H	1.55362800	-7.88621200	-5.33135100
H	-1.32302100	-4.60304200	-3.93190600
H	0.07721200	-4.19206700	-2.93487900
H	-0.14632300	-7.02906600	-3.42701600
H	-3.21152900	-5.27822600	0.02885500
H	-2.31229700	-3.97157700	0.83824300
H	-0.99398600	-3.69520000	-0.99746400
H	0.15652400	-8.05392700	1.18555200
H	-2.22177800	-7.32719800	0.00821500
H	-2.55529000	-6.55163400	-7.33973200
H	-1.91479000	-6.28374300	-5.72658900
H	-3.71586300	-5.05145800	-3.84595200
H	-5.67432900	-3.54015800	-4.44496300
H	-6.55435100	-4.05590900	-6.79589200
H	-3.69235300	-8.63367000	-6.58670100
H	-11.31052800	5.18981700	-2.71827400
H	-11.45133700	3.68237400	-3.59827500
H	-11.87769500	4.02250500	-0.58840400
H	-11.97922000	2.47924600	-1.46121900
H	-13.94579500	3.60251600	-3.04924900
H	1.20293300	0.07207500	-4.56404900
H	1.51050400	-2.75915000	-3.41697900
H	2.84662900	-1.77084500	-3.95569500
H	1.27808500	4.42427100	0.15240800
H	3.42775700	1.37557000	1.67737900
H	-0.42575600	2.59276800	0.20554900
H	-0.97091800	-1.20518400	-0.76106000
H	-5.41169300	3.66866400	-1.66405100
H	5.81432800	0.97330600	-3.05616400
H	2.57257200	-2.84111000	0.24843100
H	0.97406900	-3.49224700	0.69223700
H	1.60857600	-3.69477100	-0.95481600
H	3.91207400	3.25465100	0.28395300
H	-0.75741000	-2.00209100	-3.88387500
H	-13.79245000	5.19869300	-2.28284500
H	-13.39523400	4.99391400	-4.01156400
H	-5.36636700	5.12920500	4.53634200
H	-4.86001300	6.18665000	5.92256800
H	-4.38907000	2.24644500	3.66607400
H	-3.97749200	3.93821800	4.18973000
H	-1.31071400	8.03884400	5.03561500
H	-1.20011900	9.80080400	5.45155300
H	-4.89899200	4.43943400	-4.71547000
H	-3.35588800	4.90630200	-5.53846700

H	1.82866900	9.60821000	-4.95855600
H	1.93240000	8.63156000	-6.43885700
H	8.25706900	7.63477200	1.78728800
H	9.02851600	6.26439500	2.63779200
H	5.53403300	6.25276500	-4.38970200
H	2.23243200	2.97550800	-4.75133300
H	9.62051900	3.92952500	-2.48490100
H	5.20819000	2.16820700	-7.72342400
H	6.11233000	0.73005900	-8.35290300
H	-0.11185100	-1.92160800	-8.75376600
H	0.03865600	-3.56010900	-8.09890900
H	-5.14004500	-0.19972700	-7.54835900
H	-4.82633500	-1.90514400	-7.98468300
H	-3.38083400	-8.19814400	-4.88559500
H	-2.01154600	-8.58898400	-5.94925100
H	3.04023800	-6.92443800	-5.24097300
H	8.80588100	-6.20057600	-4.19000700
H	8.89698200	-5.79537200	-2.46570800
H	-1.15188300	-9.11958500	0.60454700
H	-1.02246900	-8.65224000	2.34384600
H	2.83691500	-7.56300800	6.91081900
H	4.47251800	-7.93455900	7.57324400
H	9.68491200	-5.05062400	2.17216400
H	9.37823500	-5.28033300	3.89523900
H	11.07186100	0.73754400	4.46139200
H	11.11388800	2.31618300	3.58645800
H	-2.28454100	1.14022200	-0.52538300
H	-7.31811900	1.37263800	0.86635300
H	-8.99983800	-0.40234000	-0.33743100
H	-9.08566000	-1.57600600	1.91851100
H	-5.37205500	-4.82833200	3.21015200
H	-6.36763700	-5.49168900	1.89615600
H	-5.69925900	-3.85999600	1.75417500
H	-7.83015600	-4.81608100	3.73446100
H	-6.43885800	-2.36445700	3.61469000
H	-7.94029100	1.78747000	-1.32534800
H	-9.31600900	-2.83683900	8.81660200
H	-7.91889800	-3.80167300	9.20413600
H	-0.34680600	-1.86332500	3.46494900
H	-0.85810000	-3.54358700	3.50749400
H	-4.03003300	-2.71536400	4.74439100
H	-1.95798100	-1.08323500	1.43535400
H	-4.41222800	-0.79065700	1.05319900
H	-7.64062300	-3.15897400	5.77397800
H	0.22427600	-1.74666900	7.41795200
H	1.94370900	-1.64055600	9.37340100
H	4.47320600	-2.39249300	7.80673000
H	4.39978300	-1.32875000	9.22636200
H	4.03088700	-3.06036200	9.38543700
H	-9.93700800	-4.67235900	10.41834700
H	-9.61190500	1.86819400	3.60903600
H	-8.82453900	2.97704700	2.51244900
H	-7.86374100	1.81452100	3.48674700
H	-10.78266700	-4.91571900	8.87456800
H	-9.37001300	-5.89506800	9.25985900
H	-9.44657500	-3.69299600	2.57308100
H	-8.44709000	-4.31222800	1.24769700
H	-5.98898000	-0.26338100	-0.82831600
H	-7.26974700	-0.45908500	-2.01096200
H	2.38838800	-3.46989000	6.95722300
H	-2.02895400	-3.04755300	5.74657500
H	1.75084800	-3.90368000	8.52524200
H	-1.23376800	-1.48451300	5.75536800
H	2.71548100	-0.87182700	6.78283300
H	2.85648700	-0.96330100	1.24620200

TS5 (+1.3)

C	-8.76014100	1.96195200	2.88072600
C	-8.91346400	0.81624300	1.91254800
O	-9.89162000	0.06074100	1.97071200
N	-7.85723300	0.58329600	1.09471300
C	-7.94625100	-0.38262400	0.00093400
C	-7.65274100	-1.82670900	0.47599800
O	-6.80379400	-2.55148200	-0.06584600
C	-6.98077600	0.08662600	-1.10657100
O	-6.96984700	1.49344800	-1.21212100
N	-8.43072100	-2.24297500	1.49003400
C	-8.51558500	-3.60852800	1.98754600
C	-7.47251700	-4.02365100	3.06080300
C	-6.19306200	-4.59321500	2.44693400
O	-7.21546200	-2.95948300	3.97599200
C	-9.82859400	-4.93570000	9.27339400
C	-8.90175600	-3.89386200	8.63236700
C	-8.65571600	-4.06682400	7.13348100
O	-9.12717700	-4.96012800	6.45495700
O	-7.84987100	-3.10279200	6.67356200
C	-1.21499500	-2.56958100	5.43877500
C	-0.02305200	-3.29563200	6.06201200
O	0.43279500	-4.32840300	5.58621500
C	-1.22040500	-2.58183400	3.89516300
C	-2.56128400	-2.16281800	3.35705300
C	-3.82890800	-2.30773000	3.87741900
N	-2.75792700	-1.52125700	2.13970900
C	-4.09851300	-1.29339000	2.00275300
N	-4.77833300	-1.76521300	3.03264200
N	0.45818000	-2.75910300	7.23477000
C	1.75889400	-3.16657400	7.75743900
C	2.47223100	-1.97178300	8.40834100
C	3.93823200	-2.29447300	8.68476100
O	2.32909600	-0.78499700	7.63029700
N	-4.46663000	3.29349300	-1.68681900
N	-2.88887500	1.99299100	-0.96510700
N	-3.71679000	-0.31569700	-5.20740700
N	-4.36773800	-2.28334100	-1.42267600
N	-0.42802100	-1.02130300	-4.27570900
N	0.28917000	-1.96505200	-6.71409400
N	7.11245300	3.98375000	5.87708100
N	5.96317300	1.63169100	-1.61992300
N	4.77562000	2.86135100	-0.22078700
N	6.80856000	4.63033300	-3.66074300
N	3.47348100	4.12776600	-3.40019100
N	0.13550700	-6.10757900	-3.83647200
N	-1.52404900	-4.62404600	-1.04391800
N	-1.77163000	-7.21706700	0.81519200
N	-4.87279900	-5.32309800	-6.98470100
N	-5.29118200	-4.21714800	-5.09856100
C	-3.75586400	4.37232000	-4.71870700
C	-3.18386400	4.78036500	-3.35101900
C	-3.30006100	3.70462500	-2.32319500

C	-2.31570100	2.87054800	-1.86344000
C	-4.18848800	2.27254100	-0.86480700
C	-4.89702600	-1.12956600	-7.18937200
C	-3.65361000	-1.14452100	-6.29381200
C	-2.81430200	-0.43491900	-4.05757700
C	-1.35498100	-0.04617900	-4.41710400
C	-2.95238700	-1.79695200	-3.34179500
C	-4.33222900	-1.98913800	-2.73457200
C	0.97956000	-0.89262600	-4.61171200
C	1.31116900	-1.39979600	-6.03123000
C	1.86432900	-1.63957800	-3.59873600
C	0.68287500	-1.76876200	-0.24800000
C	0.46337600	-2.48112800	-8.05349100
C	1.60026000	8.69584700	-5.35922600
C	2.08785400	7.43962600	-4.61581400
C	1.40747700	7.12358700	-3.27848300
C	-0.05966700	6.73457600	-3.40010600
C	-1.32932800	9.02915900	4.71480100
C	-0.21587000	9.28368700	3.67041700
C	0.01471500	8.19619600	2.64750700
C	-0.93141800	7.89847500	1.65710000
C	1.21152400	7.46974400	2.64146600
C	-0.70057700	6.90881700	0.70660300
C	1.46487900	6.48196200	1.69204100
C	0.50270400	6.18544400	0.72011900
C	-5.51107700	6.04569400	5.09018800
C	-5.34653600	7.14682500	4.01179800
C	-5.75555200	6.61103600	2.65173400
C	-7.08810800	6.29388700	2.35185000
C	-4.78903600	6.28997400	1.69049700
C	-7.44874200	5.66524100	1.15989300
C	-5.12837700	5.67294300	0.48803300
C	-6.46330200	5.33558000	0.21238600
C	-3.66065900	3.06016500	3.57987500
C	-2.33231100	2.56562800	4.19464600
C	-1.31791400	3.70333300	4.38598700
C	-1.76217700	1.37244700	3.43001900
C	-0.83710400	4.36685000	3.09952700
C	3.86332200	-7.78981000	6.61013900
C	4.51434700	-6.55896400	5.94400500
C	4.10095300	-6.32248400	4.51081000
C	4.98213800	-6.59621900	3.45738700
C	2.82483700	-5.83272500	4.21142900
C	4.60150700	-6.37094800	2.13316700
C	2.43537600	-5.61680500	2.88977900
C	3.32676700	-5.87981600	1.84697900
C	11.10625600	1.15190300	3.51671700
C	9.93078600	0.71012800	2.63026300
C	8.55751400	1.29179900	3.01429900
C	8.51774000	2.81327700	2.82625500
C	7.43491600	0.63123000	2.20202000
C	8.11325600	6.65416900	2.40771200
C	7.25054700	6.94449200	3.63087700
C	6.93638500	5.71454900	4.43337000
C	7.68192100	5.17537200	5.45175900
C	5.84200100	4.80273600	4.20447500
C	5.98647300	3.72402100	5.12078300
C	4.76307100	4.78677900	3.30427700
C	5.09896600	2.64395600	5.14831400
C	3.87072700	3.72338200	3.33226900
C	4.04482300	2.65934000	4.24206100
C	8.45760900	-5.51840900	-3.44831600
C	6.92611600	-5.62237100	-3.36994300
C	6.28950700	-4.55948900	-2.51072600
C	5.74150500	-3.41701500	-3.10467000
C	6.25838600	-4.65345000	-1.11350000
C	5.16442100	-2.40724100	-2.34466500
C	5.66124100	-3.66365900	-0.33409900
C	5.09871000	-2.54093500	-0.95285900
C	9.34213200	-4.60973700	3.06707000
C	7.92805800	-4.04734400	2.85983400
C	7.23233700	-3.51228900	4.12463400
C	8.05069100	-2.41985900	4.82538800
C	5.82407500	-3.00500300	3.78379300
C	8.64510200	3.52969000	-2.54951400
C	7.68193600	4.70750400	-2.62116900
C	8.29248900	2.62942400	-1.33248000
C	6.81708400	2.54120000	-1.01639500
C	6.07977400	3.31023500	-0.13853600
C	4.75421000	1.84619300	-1.11740900
C	5.56971700	5.38423400	-3.67914300
C	4.49269600	4.45399300	-4.24107400
C	2.65112000	2.96240800	-3.70101900
C	1.43159300	2.82466400	-2.79114900
C	6.02977300	1.45099900	-7.49262800
C	5.63250700	0.59253400	-6.28509800
C	6.11525500	1.12893900	-4.95915400
C	2.13791700	-7.16977800	-4.76165600
C	1.28443000	-5.91329500	-4.55561200
C	2.55013500	-7.85398000	-3.43991000
C	3.52117700	-7.03618300	-2.56640400
C	2.90332900	-5.76066500	-1.97726900
C	4.09109100	-7.91033800	-1.44341300
C	-0.61492600	-5.00133200	-3.27516800
C	-1.40102700	-5.49965300	-2.05917700
C	-2.26065000	-4.92513300	0.18188900
C	-1.48996900	-5.91348900	1.08118800
C	-0.91431800	-8.32874500	1.22719100
C	-2.99100400	-8.07017700	-5.99470400
C	-2.77924000	-6.58236500	-6.35021900
C	-3.91605900	-5.65653400	-6.04342900
C	-4.16370500	-4.97843000	-4.86856000
C	-5.66754200	-4.45448700	-6.38590900
C	1.61121600	2.14895600	0.83221100
C	2.44863400	1.09461400	1.30373000
C	2.06885900	-0.21947500	1.13154300
C	0.27388700	1.82087600	0.49029500
C	-0.13473100	0.48922600	0.38360500
C	0.81452900	-0.56712700	0.58130200
C	-13.30350400	4.59404200	-3.04342900
C	-11.81161600	4.30303000	-2.75876200
C	-11.47560700	3.51916600	-1.47858500
C	-9.94858100	3.33993100	-1.31940500
C	1.42405500	-3.04084700	0.07381500
O	-2.66911300	-1.84339900	-6.53161300
O	-1.09802300	1.10658800	-4.76104200
O	-5.36231300	-1.89073900	-3.42185600
O	2.46150000	-1.29882300	-6.45623000
O	-0.75330300	6.98214400	-4.36820900
O	-0.59948000	6.14004500	-2.32918600

O	0.70805800	5.21964600	-0.21377300
O	-6.76040200	4.71149000	-0.95489500
O	4.49190600	-1.60278500	-0.17794200
O	7.65768800	5.60010800	-1.77297200
O	4.58661000	4.01215300	-5.38254600
O	0.26590500	3.41493100	-3.36809700
O	6.99571700	1.97819600	-4.85016700
O	5.54064200	0.52922000	-3.92944400
O	1.61185500	-4.80175300	-4.95753600
O	-1.89524400	-6.63678500	-2.05833700
O	-0.69006800	-5.53509200	1.93100700
O	2.10321400	3.36483500	0.70571800
O	-1.39518300	0.13686000	0.04813500
O	-9.48790300	2.16771800	-1.37568000
O	-9.28364300	4.41152600	-1.17067100
O	-0.57463300	-2.03219200	-0.72146800
S	1.86993300	-0.85526200	-1.94188700
H	-3.65963900	5.70236200	-2.99820500
H	-2.12528600	5.00861000	-3.44044600
H	-1.26848800	2.86255900	-2.12455300
H	-4.92047100	1.78167000	-0.25042600
H	-3.52152900	3.32178300	-4.92525500
H	-5.76544500	-1.42894700	-6.59421900
H	-2.80571200	-2.59470400	-4.07810200
H	-2.18318200	-1.87979400	-2.57062900
H	-5.27764200	-2.39966400	-0.95762800
H	-3.52002700	-2.32792700	-0.87969500
H	-3.11570500	0.35754900	-3.36646600
H	-4.63816400	0.01970700	-4.96434600
H	1.52956300	-2.47488000	-8.28068200
H	-0.66017000	-1.89903100	-6.36481500
H	1.94838700	6.57157000	-5.26913900
H	3.16835500	7.51331100	-4.43886100
H	1.45496300	7.97953300	-2.59002800
H	1.93708000	6.30321200	-2.78281600
H	0.51202100	8.67988800	-5.43611200
H	-0.42942200	10.23391200	3.16153700
H	0.72499700	9.44082400	4.21116000
H	1.96604100	7.67971300	3.39625900
H	-1.86885800	8.44964600	1.62652200
H	2.39106800	5.91915200	1.70857200
H	-1.43934600	6.68297600	-0.05378000
H	0.04791600	5.75876200	-1.68415700
H	-2.32792900	9.18029700	4.29378400
H	-5.92348100	8.04180500	4.27847600
H	-4.29608200	7.45476300	3.96376200
H	-7.86743400	6.53268300	3.07313100
H	-3.74322500	6.51691200	1.88551600
H	-8.48495500	5.43452500	0.93400800
H	-4.35671700	5.45169500	-0.24032800
H	-7.77078300	4.51883100	-1.04946800
H	-6.53854100	6.01364300	5.46840400
H	-2.58502800	2.21005200	5.20613300
H	-1.61792200	1.59724900	2.37161800
H	-0.78872900	1.06208400	3.82812800
H	-2.44437100	0.52333700	3.50347300
H	-1.77257200	4.46527000	5.03612900
H	-0.45047500	3.31257000	4.93582700
H	-1.66950800	4.75627600	2.50625300
H	-0.18123600	5.20928300	3.32123300

H	-0.27682600	3.67175200	2.46980600
H	-3.56894800	3.37493400	2.53759500
H	4.28702900	-5.66917500	6.54584900
H	5.60368400	-6.68086600	5.97995500
H	5.97924300	-6.97072700	3.67829600
H	2.12697500	-5.60004500	5.00839700
H	5.30051000	-6.56947400	1.32627100
H	1.43467500	-5.25769200	2.68087300
H	3.03263800	-5.69709800	0.82021100
H	3.93038600	-8.65928000	5.94795700
H	10.13496800	0.99435200	1.58855600
H	9.87342000	-0.38636300	2.63685000
H	8.38024000	1.07328400	4.07972500
H	7.51275900	3.20050600	2.99790800
H	8.80335600	3.07858700	1.80187500
H	9.19059600	3.34039700	3.50885900
H	6.45521200	1.00332700	2.51776500
H	7.53509800	0.85112800	1.13450000
H	7.43804100	-0.45761600	2.31724600
H	12.05653400	0.86080500	3.05268900
H	6.31396500	7.42084400	3.31587200
H	7.76266600	7.67667600	4.26862600
H	8.58627600	5.54596500	5.91366200
H	7.50149700	3.36579400	6.56823700
H	4.63076300	5.59696000	2.59196100
H	3.04359800	3.69653800	2.63146800
H	5.23085600	1.82248700	5.84711900
H	3.33807700	1.83600500	4.22157900
H	7.62280700	5.96040100	1.71896700
H	6.50970600	-5.55145200	-4.38166200
H	6.66211600	-6.62096400	-2.99835000
H	6.68873800	-5.52590000	-0.62544000
H	5.76411400	-3.31155200	-4.18627900
H	5.59572700	-3.75808000	0.74384000
H	4.78193900	-1.51334200	-2.82150300
H	3.81247900	-1.15413100	-0.73349200
H	8.77859800	-4.50255000	-3.69312200
H	7.96320600	-3.24828700	2.10710200
H	7.28968900	-4.83054900	2.43347000
H	7.12642200	-4.35731900	4.82307900
H	8.21545100	-1.56848400	4.15620400
H	7.52349100	-2.04523300	5.71013800
H	9.03003700	-2.78429500	5.15181400
H	5.86694000	-2.17469300	3.06971800
H	5.30841100	-2.64370400	4.68172100
H	5.20986800	-3.79563600	3.34579300
H	10.06516000	-3.82550800	3.31123200
H	8.67871700	1.62115100	-1.50974400
H	8.78629800	3.01025900	-0.43392700
H	6.36750200	4.11945000	0.51128500
H	3.85938300	1.29187700	-1.36234700
H	8.58980300	2.93754500	-3.46323300
H	5.36082500	5.73125700	-2.66323000
H	6.84471000	3.80497800	-4.25746200
H	1.26442900	1.76383200	-2.57378500
H	1.60065300	3.31869800	-1.83061300
H	-0.09939000	2.77243800	-4.00124000
H	3.27737800	2.06224800	-3.63367700
H	3.61127700	4.32350900	-2.41675700
H	4.55537900	0.40901900	-6.22836100

H	6.07968300	-0.40869400	-6.36295700
H	6.97476700	1.96155900	-7.29456300
H	1.66439700	-8.11843500	-2.84601700
H	3.03257800	-8.80570200	-3.69777000
H	4.35561000	-6.72893700	-3.21009200
H	1.98549600	-5.99398500	-1.42347800
H	2.67220800	-5.02331300	-2.74573600
H	3.60597100	-5.28310600	-1.29221200
H	4.54927800	-8.82669100	-1.83357000
H	4.85219800	-7.36165900	-0.88169000
H	3.30653500	-8.20045800	-0.73417300
H	1.57788000	-7.87892800	-5.38437700
H	-1.32961700	-4.59053700	-4.00277300
H	0.07249800	-4.19690900	-3.00145000
H	-0.14130900	-7.02393700	-3.51233200
H	-3.25247100	-5.31343500	-0.06884500
H	-2.36918200	-3.99389900	0.73881700
H	-1.04534800	-3.71888700	-1.06479200
H	0.14241000	-8.06111800	1.10931400
H	-2.22503000	-7.34563700	-0.08443400
H	-2.54466500	-6.48844800	-7.41591300
H	-1.89388800	-6.23568600	-5.80479800
H	-3.68046500	-5.00769500	-3.90469900
H	-5.62805700	-3.47853000	-4.48155700
H	-6.52309100	-3.96931400	-6.83442500
H	-3.68079700	-8.57253800	-6.67731600
H	-11.25992400	5.24870800	-2.72256300
H	-11.40314500	3.74800600	-3.61392700
H	-11.84804600	4.06893000	-0.60456100
H	-11.94777000	2.53210300	-1.48910800
H	-13.90043100	3.67623600	-3.07791800
H	1.22191800	0.17140500	-4.59524200
H	1.56493800	-2.69170000	-3.55059400
H	2.88119000	-1.63314000	-3.99403900
H	1.29132400	4.42687200	0.16343300
H	3.43688000	1.33274100	1.67767600
H	-0.41084400	2.62368200	0.24076200
H	-1.10067600	-1.15026400	-0.54631200
H	-5.39038900	3.75445200	-1.68294700
H	5.88462100	0.91767500	-3.03997100
H	2.45782000	-2.85503700	0.35517600
H	0.90297400	-3.55105700	0.89378600
H	1.42447100	-3.70176800	-0.79474700
H	3.93422200	3.18665700	0.28206900
H	-0.73470300	-1.94198300	-4.00641300
H	-13.74319000	5.26547300	-2.29847400
H	-13.34060000	5.07264500	-4.02702500
H	-5.33977200	5.10954400	4.54810600
H	-4.83402600	6.15401200	5.94435200
H	-4.37047600	2.23007300	3.65817600
H	-3.95425400	3.91607100	4.19661900
H	-1.27481200	7.99967500	5.08383400
H	-1.15895700	9.75783700	5.51416600
H	-4.84424300	4.49144200	-4.70732800
H	-3.29662700	4.95891600	-5.52148800
H	1.90369500	9.63633600	-4.88712900
H	2.00866200	8.67107600	-6.37479300
H	8.30210500	7.58497400	1.86396700
H	9.06553500	6.20496400	2.70609200
H	5.59443200	6.26239300	-4.33276200
H	2.28169700	3.00070300	-4.73129800
H	9.66399700	3.91171100	-2.43183900
H	5.26424400	2.20569300	-7.69991000
H	6.16500200	0.76911800	-8.33774500
H	-0.06769700	-1.85552900	-8.78026300
H	0.07436100	-3.49977000	-8.13797800
H	-5.09340100	-0.12415000	-7.57777000
H	-4.78465200	-1.82723000	-8.02658100
H	-3.37329100	-8.15008500	-4.97285900
H	-2.00195500	-8.53766700	-6.03507000
H	3.05370500	-6.89806300	-5.29691800
H	8.81856400	-6.20451700	-4.22127000
H	8.90548100	-5.81330200	-2.49350100
H	-1.16609700	-9.12355100	0.51709800
H	-1.04070000	-8.67051300	2.26048200
H	2.80754300	-7.63223700	6.84867100
H	4.43952300	-8.01526400	7.51349600
H	9.68070800	-5.10840800	2.15272000
H	9.36744000	-5.35062500	3.87289200
H	11.08190500	0.65608100	4.49243800
H	11.13280400	2.24144400	3.63022100
H	-2.32303400	1.22089900	-0.47110300
H	-7.29184900	1.39082800	0.84787600
H	-8.97524600	-0.36242700	-0.38069200
H	-9.12361700	-1.56817900	1.81954600
H	-5.46977900	-4.83473700	3.23060600
H	-6.42592200	-5.51036500	1.89493900
H	-5.74408200	-3.88460300	1.75104000
H	-7.94921900	-4.80543200	3.66549500
H	-6.51507900	-2.37634800	3.59545000
H	-7.91444400	1.82824200	-1.34260200
H	-9.29312200	-2.87960200	8.77613000
H	-7.91843300	-3.89274100	9.11845300
H	-0.44355100	-1.91139700	3.50845600
H	-0.96523100	-3.58885200	3.54447200
H	-4.12603900	-2.77399400	4.80492900
H	-2.05730800	-1.08321400	1.53233800
H	-4.51855800	-0.78740500	1.14662000
H	-7.68715800	-3.17342200	5.69389200
H	0.23888500	-1.78422200	7.39713700
H	1.96834000	-1.72914400	9.35247800
H	4.46719900	-2.50267000	7.74694000
H	4.42705100	-1.44932300	9.17570300
H	4.03644400	-3.17693900	9.32601100
H	-9.94201300	-4.73666700	10.34302100
H	-9.59452100	1.87260000	3.58098300
H	-8.79929100	2.98669300	2.49578400
H	-7.84614600	1.81281200	3.46396700
H	-10.81733300	-4.91654500	8.80713500
H	-9.42786000	-5.94482100	9.14526600
H	-9.51374100	-3.69993200	2.42418700
H	-8.45016200	-4.30245800	1.14238800
H	-5.96109200	-0.22511600	-0.85668600
H	-7.23559900	-0.41458200	-2.04709400
H	2.35710700	-3.54451100	6.91631200
H	-2.10197200	-3.10181700	5.80267900
H	1.72995600	-3.98173200	8.48852700
H	-1.29795500	-1.54272200	5.81086600
H	2.72753600	-0.94651800	6.76214800
H	2.79194100	-0.99864800	1.34214900

E:DHAP (-15.6)

C	-8.76337500	1.79646000	2.79365300
C	-8.90220200	0.69432200	1.77522700
O	-9.89272700	-0.04468200	1.75875500
N	-7.81584900	0.48631900	0.98898200
C	-7.87711300	-0.42920200	-0.14845600
C	-7.58012900	-1.87948400	0.30721900
O	-6.60107700	-2.53156400	-0.08999800
C	-6.89266600	0.09280800	-1.21133000
O	-6.90873500	1.50488800	-1.26320400
N	-8.49515100	-2.36715300	1.16246700
C	-8.56558200	-3.73322100	1.65572300
C	-7.70808700	-4.06465800	2.91106800
C	-6.33635100	-4.62834400	2.53775700
O	-7.62994800	-2.94391400	3.79325500
C	-9.90314600	-5.36176800	8.88024400
C	-8.92745700	-4.31293300	8.33739400
C	-8.76546100	-4.31063500	6.81882600
O	-9.28996200	-5.11934800	6.07340600
O	-7.96978800	-3.31504500	6.42490100
C	-1.63918000	-3.01944900	5.84700400
C	-0.37391100	-3.71945900	6.32929700
O	-0.05622400	-4.83396200	5.92221700
C	-1.64513000	-2.87374400	4.31856700
C	-2.96322700	-2.40139700	3.78835900
C	-4.21102900	-2.24384500	4.34826400
N	-3.11979100	-2.03132200	2.46522600
C	-4.41813900	-1.66675000	2.28024800
N	-5.11085100	-1.78892500	3.40028700
N	0.36665200	-3.03945300	7.25393700
C	1.69933900	-3.49942100	7.61184400
C	2.50358400	-2.35654700	8.24042300
C	3.97513400	-2.73088200	8.38631800
O	2.33632600	-1.14338300	7.50389800
N	-4.47276900	3.50454200	-1.62246100
N	-2.98489800	2.12751400	-0.88627900
N	-3.64798800	-0.12107800	-5.29596800
N	-4.13922900	-2.20054400	-1.51469800
N	-0.34582300	-0.68022200	-4.45647200
N	0.45827900	-1.68887700	-6.82227300
N	7.01125900	3.73976200	6.10920100
N	6.06962600	1.60573900	-1.46759400
N	4.81090100	2.83845700	-0.13333100
N	6.88443400	4.76625500	-3.40151100
N	3.53796300	4.26456500	-3.19882700
N	0.30632600	-5.69751400	-4.35643000
N	-1.26153100	-4.25151800	-1.49716300
N	-1.61275400	-7.17654100	0.56915500
N	-4.74675000	-5.07403500	-7.21169000
N	-5.06715800	-3.98583800	-5.29573700
C	-3.65557600	4.53194900	-4.62428900
C	-3.09192000	4.92397500	-3.25418800
C	-3.27542900	3.85738300	-2.23424100
C	-2.34159300	2.97186600	-1.77103400
C	-4.27070700	2.46969400	-0.80247800
C	-4.74037900	-0.86620100	-7.33647200
C	-3.52515600	-0.90537200	-6.41053100
C	-2.73183200	-0.18648200	-4.15595300
C	-1.30595100	0.25596800	-4.57582500
C	-2.78332200	-1.54556200	-3.42469700
C	-4.14379900	-1.82476500	-2.81098000
C	1.05192500	-0.47246800	-4.78588500
C	1.37613900	-0.88198200	-6.23109700
C	1.94420300	-1.33225500	-3.85618800
C	0.27063000	-1.83389900	0.98507700
C	0.63600100	-2.16589700	-8.17605900
C	1.69496600	8.89304700	-5.00621700
C	2.17328800	7.60714900	-4.31225200
C	1.46127000	7.22951000	-3.00961000
C	-0.00745900	6.86880600	-3.18134000
C	-1.38461600	8.80265900	5.02811300
C	-0.25285700	9.10851000	4.01836000
C	-0.00631400	8.07601300	2.94452100
C	-0.93561400	7.83680100	1.92297800
C	1.18789000	7.34621900	2.92221200
C	-0.69177200	6.89794900	0.92581500
C	1.45565500	6.40953900	1.92593500
C	0.50853300	6.17222800	0.92543600
C	-5.56135500	5.79470600	5.21782900
C	-5.39360300	6.95085900	4.20251100
C	-5.79801300	6.49638800	2.81274200
C	-7.11832700	6.13454800	2.50792800
C	-4.83555900	6.30867800	1.81414300
C	-7.46850900	5.58603700	1.27458600
C	-5.16597800	5.77680600	0.56941300
C	-6.48574500	5.38802900	0.28847900
C	-3.67866400	2.87906100	3.61359400
C	-2.33988400	2.32446000	4.14039600
C	-1.31520100	3.43382100	4.42609900
C	-1.79893200	1.24659400	3.20115000
C	-0.92112800	4.27444300	3.21445300
C	3.83649900	-8.06577800	6.30667200
C	4.43960400	-6.77832300	5.71106100
C	3.92646100	-6.47236700	4.32548000
C	4.76918000	-6.59068900	3.21467300
C	2.58619400	-6.11646900	4.12104900
C	4.29159900	-6.35507700	1.92432000
C	2.10401000	-5.90753700	2.83018400
C	2.95301700	-6.01906600	1.72614900
C	11.09395400	1.01602300	3.69261800
C	9.92942600	0.61375000	2.77350900
C	8.55134900	1.17533300	3.17102900
C	8.50443500	2.70294600	3.04598500
C	7.43727200	0.54712500	2.32243600
C	8.09904700	6.55086900	2.76646200
C	7.21741500	6.78827500	3.98760000
C	6.88215200	5.52768500	4.73150400
C	7.60438200	4.93812100	5.73853600
C	5.77895000	4.64182300	4.45149900
C	5.89282400	3.52679300	5.32809900
C	4.71467600	4.67771100	3.53524700
C	4.98492800	2.46360300	5.30548300
C	3.80526800	3.62934300	3.51189800
C	3.94432200	2.53132400	4.38621500
C	8.57146600	-5.36879400	-3.57966800
C	7.03586700	-5.47053700	-3.52841400
C	6.32826800	-4.47228800	-2.63697300
C	5.64868000	-3.38969200	-3.20815100

C	6.27364500	-4.60488800	-1.24172300
C	4.90209200	-2.50620400	-2.43889900
C	5.50898400	-3.73977400	-0.45570700
C	4.77594600	-2.70780700	-1.05926400
C	9.35621700	-4.72706200	2.97979600
C	7.94622400	-4.15005600	2.78561200
C	7.24879400	-3.67010500	4.07223200
C	8.07335700	-2.62039800	4.82916100
C	5.84709800	-3.13156900	3.75311300
C	8.71386600	3.63312700	-2.30978200
C	7.74644000	4.80793900	-2.35087900
C	8.34739600	2.68432000	-1.13312600
C	6.87464100	2.55745600	-0.86362200
C	6.09560500	3.32760000	-0.02746200
C	4.84248700	1.79733900	-1.00290600
C	5.65023900	5.52600700	-3.40483700
C	4.56889500	4.63030400	-4.01633200
C	2.74088300	3.09896200	-3.57003500
C	1.52113300	2.85600000	-2.68068000
C	6.18017900	1.75460400	-7.36971000
C	5.74452300	0.84466800	-6.21151500
C	6.21022600	1.29838800	-4.84956000
C	2.27767400	-6.98212900	-5.05407600
C	1.54461100	-5.64510500	-4.92075600
C	2.59025700	-7.66518600	-3.71184000
C	3.50907800	-6.86334500	-2.76946000
C	2.82669000	-5.64397200	-2.13273200
C	4.06910100	-7.77855000	-1.67392100
C	-0.30439800	-4.52480500	-3.75550300
C	-1.15959200	-5.03198300	-2.60389200
C	-1.84379500	-4.87942400	-0.32103500
C	-0.97106000	-6.05729200	0.13454500
C	-0.85964400	-8.39559300	0.83558100
C	-2.82939800	-7.84509300	-6.39991700
C	-2.62001500	-6.34015900	-6.68659400
C	-3.74656700	-5.42166600	-6.32029000
C	-3.93384100	-4.75520900	-5.12805900
C	-5.50794900	-4.20925100	-6.56641600
C	1.72658900	2.17965400	0.83203600
C	2.52361300	1.12715400	1.39527300
C	2.05441900	-0.15646800	1.44854800
C	0.41495800	1.86528000	0.39864600
C	-0.04611000	0.56531900	0.46086700
C	0.75047200	-0.49502600	0.99717500
C	-13.22742700	4.65794200	-3.08427800
C	-11.74000800	4.36156700	-2.78579600
C	-11.42449400	3.52883700	-1.53141800
C	-9.90040600	3.35974600	-1.34503200
C	1.14775800	-2.98305800	1.39125500
O	-2.52071400	-1.56853100	-6.65486400
O	-1.12121400	1.40282600	-4.99193400
O	-5.19473200	-1.70801200	-3.46100300
O	2.45124100	-0.56248500	-6.73881200
O	-0.68102400	7.16165000	-4.14986200
O	-0.57335800	6.23449400	-2.14489700
O	0.72394900	5.25988000	-0.06218300
O	-6.77014100	4.83596500	-0.91738800
O	3.95115800	-1.93609600	-0.30323200
O	7.70822300	5.66882800	-1.47000900
O	4.66522900	4.25111600	-5.17851500
O	0.32153700	3.42281300	-3.21207500
O	7.06464800	2.16232700	-4.67021000
O	5.64961100	0.60720400	-3.86703700
O	2.06464600	-4.58804600	-5.26390900
O	-1.73084200	-6.12532900	-2.67769800
O	0.25235500	-5.95008900	0.13983100
O	2.22640000	3.37710700	0.70254100
O	-1.29180100	0.26772500	0.01264600
O	-9.42339500	2.19704700	-1.42899000
O	-9.25457700	4.43657700	-1.14536000
O	-0.93950400	-2.09797900	0.63303200
S	1.39040800	-1.33257900	-2.09564500
H	-3.52876200	5.86852600	-2.91159800
H	-2.02294400	5.10053400	-3.32836200
H	-1.29540000	2.89572700	-2.03357200
H	-5.04393400	1.99835600	-0.22468100
H	-3.40402700	3.49159700	-4.85662800
H	-5.62556700	-1.19201100	-6.77989800
H	-2.57021900	-2.33592300	-4.15081900
H	-2.01317600	-1.56885600	-2.64754800
H	-5.03231900	-2.40019100	-1.05463900
H	-3.26666700	-2.40414400	-1.05033500
H	-3.06246800	0.59882600	-3.46964500
H	-4.58751500	0.15637200	-5.05184300
H	1.70247100	-2.14714300	-8.40363000
H	-0.47830200	-1.71683800	-6.43538700
H	2.05449700	6.76965200	-5.00811100
H	3.24865700	7.67593400	-4.10542900
H	1.50550600	8.04586900	-2.27437600
H	1.96968000	6.37479100	-2.55086700
H	0.60849100	8.87925200	-5.10246700
H	-0.45662200	10.08381000	3.55536200
H	0.67850300	9.23578100	4.58269300
H	1.92962000	7.51320900	3.69978300
H	-1.86887000	8.39523500	1.90376400
H	2.38194700	5.84663800	1.92582100
H	-1.41469400	6.71752300	0.13865700
H	0.06801100	5.83244600	-1.50957700
H	-2.37599800	8.96934400	4.59633100
H	-5.97080600	7.82939000	4.51921700
H	-4.34336100	7.26198200	4.17478400
H	-7.89504500	6.27192700	3.25760300
H	-3.79964500	6.57481900	2.01234100
H	-8.49519600	5.31829400	1.04639500
H	-4.39842500	5.66376900	-0.18742800
H	-7.77707500	4.59299800	-1.01782100
H	-6.59236700	5.73775300	5.58338500
H	-2.55929800	1.84426400	5.10636700
H	-1.62482800	1.65388500	2.20479500
H	-0.84492100	0.83579200	3.55164600
H	-2.51003200	0.42129700	3.10499000
H	-1.73022500	4.09470600	5.20068400
H	-0.41342500	2.98181300	4.86171800
H	-1.79005100	4.74825700	2.74877100
H	-0.23601000	5.07251300	3.50241600
H	-0.42026300	3.67514000	2.44936100
H	-3.60723500	3.25064200	2.58698900
H	4.22438800	-5.94116900	6.38930000
H	5.53139700	-6.87179400	5.67583700
H	5.81179500	-6.86165200	3.36538000

H	1.91238800	-6.00078200	4.96648400
H	4.96099200	-6.43073100	1.07386600
H	1.06109900	-5.65562700	2.67668600
H	2.56507900	-5.84552600	0.73131700
H	3.92273500	-8.89462400	5.59744200
H	10.14392300	0.94608200	1.74842400
H	9.87351900	-0.48187900	2.72868400
H	8.36729500	0.91105500	4.22505300
H	7.50037200	3.07928700	3.24628500
H	8.77299600	3.01084600	2.02904300
H	9.18541300	3.20458700	3.73971800
H	6.45389700	0.90060900	2.64885900
H	7.54364900	0.81630600	1.26675000
H	7.44513000	-0.54580400	2.38838700
H	12.05026600	0.74597800	3.22873900
H	6.28789700	7.28242900	3.67887900
H	7.72080400	7.48971300	4.66554000
H	8.50705000	5.27658500	6.22746400
H	7.38343100	3.08815200	6.77847800
H	4.60839400	5.51398700	2.84948400
H	2.99245800	3.64245900	2.79642000
H	5.09034800	1.61610000	5.97707500
H	3.22268900	1.72286900	4.32772300
H	7.62141500	5.88414600	2.04292400
H	6.64288500	-5.35083100	-4.54462400
H	6.76787600	-6.49062100	-3.22167900
H	6.80595900	-5.42357800	-0.75981300
H	5.68079700	-3.24392900	-4.28467400
H	5.42495600	-3.88559300	0.61602800
H	4.39611700	-1.67341800	-2.90477400
H	3.19897600	-1.62025500	-0.88536400
H	8.89935700	-4.34437200	-3.77719400
H	7.98931600	-3.31726700	2.07103500
H	7.30679500	-4.90855400	2.31750400
H	7.13297100	-4.54668200	4.72922600
H	8.25411300	-1.74109500	4.20129100
H	7.54224700	-2.28087800	5.72572400
H	9.04529800	-3.01065700	5.14700900
H	5.90429600	-2.26219500	3.08868300
H	5.33080400	-2.81639700	4.66859100
H	5.22791000	-3.88910800	3.26609800
H	10.07751100	-3.95540800	3.26591900
H	8.75251500	1.69008200	-1.34573100
H	8.82649200	3.02922900	-0.21239200
H	6.34315800	4.16800000	0.59902900
H	3.97620800	1.19820000	-1.24558700
H	8.67291800	3.07485500	-3.24550100
H	5.43183600	5.83316500	-2.37794300
H	6.92564700	3.96218400	-4.02684400
H	1.40151000	1.77603900	-2.52362300
H	1.67019100	3.30494800	-1.69646900
H	-0.00142600	2.83905600	-3.92366800
H	3.39046600	2.21278000	-3.54731100
H	3.68896100	4.38568000	-2.20490900
H	4.66125400	0.68587000	-6.18884000
H	6.16785600	-0.16264200	-6.33043100
H	7.12075000	2.25441900	-7.12811500
H	1.65669200	-7.90785700	-3.18413000
H	3.06745700	-8.62810500	-3.93843700
H	4.35540800	-6.50043100	-3.36710300
H	1.88336500	-5.92360800	-1.65294100
H	2.63656000	-4.85058000	-2.85428000
H	3.47618200	-5.21021800	-1.37045700
H	4.55301800	-8.66970000	-2.09218500
H	4.80929700	-7.24074600	-1.07471200
H	3.27338600	-8.11135100	-0.99636500
H	1.70879000	-7.65892100	-5.70349400
H	-0.93812600	-3.98699400	-4.47419800
H	0.46501300	-3.83070800	-3.40231700
H	-0.07807000	-6.57706500	-4.03751400
H	-2.86580800	-5.19760300	-0.54261100
H	-1.85506500	-4.13517200	0.47585800
H	-0.58161100	-3.50249600	-1.36246100
H	0.19486400	-8.13562000	0.72842500
H	-2.59416500	-7.25764500	0.34820100
H	-2.39659400	-6.19792000	-7.74968400
H	-1.72933700	-6.01982900	-6.13490500
H	-3.39914000	-4.80129900	-4.19313300
H	-5.38946500	-3.26952800	-4.64715400
H	-6.38275800	-3.71651100	-6.96735500
H	-3.50784400	-8.32113100	-7.11250600
H	-11.19358000	5.30718600	-2.70157000
H	-11.31355900	3.84265600	-3.65467700
H	-11.82214200	4.03878900	-0.64446000
H	-11.88495200	2.53819100	-1.59216700
H	-13.81969400	3.74010900	-3.16644700
H	1.29250300	0.58666600	-4.67766200
H	1.97153200	-2.35434700	-4.24623000
H	2.95221200	-0.92699500	-3.95428100
H	1.35346200	4.50055300	0.23691800
H	3.52406500	1.36012800	1.73539800
H	-0.20074600	2.65136900	-0.01970900
H	-1.37388700	-0.73459000	0.12187300
H	-5.38280400	3.99853600	-1.64031100
H	5.99271100	0.92878900	-2.96287600
H	2.16477600	-2.86569700	1.01108900
H	1.19807900	-3.05740600	2.48459100
H	0.73604400	-3.91194600	0.99973600
H	3.95301900	3.17214500	0.33461200
H	-0.49115400	-1.43676700	-3.80040800
H	-13.68267100	5.29630000	-2.31982600
H	-13.25154400	5.17645900	-4.04780100
H	-5.37890200	4.88212500	4.64008700
H	-4.89742500	5.86953500	6.08559800
H	-4.38674300	2.04448200	3.64703600
H	-3.98423200	3.70798800	4.26061600
H	-1.33214600	7.75893700	5.35514700
H	-1.22858300	9.49821800	5.85907300
H	-4.74409200	4.64741700	-4.62447100
H	-3.18611000	5.15237200	-5.39546200
H	1.98828600	9.81408600	-4.49136000
H	2.11850200	8.91126800	-6.01575900
H	8.29278600	7.50397000	2.26442300
H	9.04839200	6.09259500	3.06035300
H	5.68176800	6.43039500	-4.02125100
H	2.38614500	3.17884600	-4.60317800
H	9.72921900	4.01482000	-2.16410100
H	5.41525200	2.51506100	-7.55716000
H	6.33023100	1.10851700	-8.24010100
H	0.11373200	-1.51331200	-8.88304200

H	0.25181400	-3.18210500	-8.30695600
H	-4.93541600	0.15339900	-7.68581200
H	-4.61429100	-1.52912400	-8.19966200
H	-3.22665200	-7.96831800	-5.38821600
H	-1.83842900	-8.30793600	-6.44501800
H	3.20007300	-6.68612200	-5.56474800
H	8.94607200	-6.02132200	-4.37515700
H	9.00605900	-5.70172200	-2.63161700
H	-1.09749300	-9.16101100	0.08914400
H	-0.99956900	-8.77994600	1.85152500
H	2.77654200	-7.92095300	6.53562400
H	4.39974000	-8.32656800	7.20845500
H	9.70999200	-5.18667300	2.05084700
H	9.37209400	-5.50046200	3.75471200
H	11.05682100	0.48033300	4.64662000
H	11.11511800	2.10002800	3.85126400
H	-2.49601600	1.36006400	-0.40218800
H	-7.24814000	1.30526500	0.79353700
H	-8.89784400	-0.39960400	-0.54872800
H	-9.21658900	-1.70688200	1.46054100
H	-5.71868600	-4.76024100	3.43024100
H	-6.46311900	-5.60436800	2.05649800
H	-5.81979900	-3.97339400	1.83522800
H	-8.25690300	-4.82727900	3.47645300
H	-6.82980900	-2.39896800	3.57556900
H	-7.85405600	1.83551600	-1.39260500
H	-9.22890400	-3.30106800	8.63223700
H	-7.92605200	-4.45111500	8.76433200
H	-0.85533800	-2.17420800	4.01422200
H	-1.38060200	-3.84459500	3.88264300
H	-4.52476500	-2.43421000	5.36399600
H	-2.35878000	-1.99750000	1.78127300
H	-4.81991300	-1.36921100	1.32353800
H	-7.88572500	-3.27115000	5.42444800
H	0.22949600	-2.03915800	7.32662000
H	2.08207100	-2.12599600	9.22634800
H	4.41966900	-2.93102100	7.40379600
H	4.52976300	-1.91449400	8.85542300
H	4.09568100	-3.63246600	8.99592200
H	-9.94802700	-5.31500900	9.97241700
H	-9.60735700	1.67859700	3.47825300
H	-8.80048200	2.83601900	2.45070600
H	-7.85763800	1.62598700	3.38392200
H	-10.90900700	-5.20039300	8.48297300
H	-9.59536000	-6.36807800	8.58428400
H	-9.61796700	-3.90598500	1.89663800
H	-8.28926700	-4.41542100	0.84513800
H	-5.87407000	-0.20879800	-0.94615600
H	-7.11342000	-0.37472500	-2.17684400
H	2.20868500	-3.86758100	6.70918500
H	-2.48236000	-3.65002000	6.15021300
H	1.66253200	-4.34428400	8.30802000
H	-1.77350200	-2.04393800	6.32573100
H	2.71982700	-1.27505900	6.62399300
H	2.70432700	-0.93968500	1.81820500

E:HPA-2 (+1.2)

C	-8.50493400	2.34950000	2.83370600
C	-8.74886200	1.37295900	1.70829000
O	-9.80733000	0.73974800	1.62870600
N	-7.70391100	1.17008000	0.86872300
C	-7.87139400	0.40602700	-0.36582800
C	-7.74875900	-1.10674000	-0.08387100
O	-6.79944500	-1.79352300	-0.49418300
C	-6.80123400	0.89784100	-1.35810500
O	-6.61898000	2.29550700	-1.26409000
N	-8.75920500	-1.63120300	0.63203200
C	-8.77657100	-3.02856200	1.01881800
C	-9.14249400	-3.26568500	2.48861300
C	-9.08468600	-4.76430300	2.79629400
O	-8.30162600	-2.51094200	3.36146500
C	-10.26794100	-5.13007200	8.35768700
C	-9.12713100	-4.26317200	7.82755900
C	-9.33924900	-3.81094600	6.39037200
O	-10.36602400	-4.02069100	5.76613100
O	-8.27508400	-3.17421400	5.90598700
C	-1.72556900	-4.00097500	5.23199200
C	-0.63284900	-4.62390500	6.09441500
O	-0.47961000	-5.83850800	6.20391700
C	-2.89770000	-4.95632500	4.95493800
C	-3.93192200	-4.34047900	4.06771000
C	-5.19608800	-3.85270300	4.29952400
N	-3.69116000	-4.08786300	2.72717400
C	-4.79263500	-3.47865800	2.20721600
N	-5.72038900	-3.32037600	3.13623100
N	0.17971900	-3.72216200	6.71544200
C	1.45684900	-4.09818900	7.29367900
C	2.03213700	-2.91927200	8.07736500
C	3.44218200	-3.20657000	8.58105200
O	1.99000900	-1.72987400	7.28127500
N	-3.96405700	3.72076200	-1.48621100
N	-2.51050100	2.21319200	-0.95814200
N	-3.43125700	0.78323300	-5.43460200
N	-4.46465100	-1.57081000	-1.97369500
N	-0.32567600	-0.23982200	-4.53926100
N	0.49288100	-1.13991300	-6.95969600
N	7.35139000	2.80108300	6.38540800
N	6.27409300	1.43928300	-1.36162000
N	5.16068800	2.77907200	0.00314500
N	7.39434900	4.52590300	-3.04175500
N	4.01599100	4.28331700	-2.89490000
N	-0.02888800	-5.43665100	-4.75830800
N	-1.43962400	-4.28254000	-1.68618500
N	-2.03557300	-6.99196600	-0.24259400
N	-4.99982700	-4.17779600	-7.74023300
N	-5.26544200	-3.06282700	-5.83112900
C	-3.11702900	5.22004900	-4.33141900
C	-2.54348800	5.34326000	-2.90625000
C	-2.75829200	4.12632900	-2.05668000
C	-1.84521100	3.16220200	-1.71477200
C	-3.79282500	2.57441000	-0.82402400
C	-4.59055000	0.14097900	-7.44704100
C	-3.39482100	-0.03621700	-6.52954400
C	-2.61576000	0.54337800	-4.24518400
C	-1.12775800	0.83734000	-4.55703200
C	-2.86494600	-0.84692200	-3.63032200
C	-4.30552100	-1.08563000	-3.21256600
C	1.10350900	-0.22679800	-4.76019600
C	1.47284900	-0.54403500	-6.22768700

C	1.76515200	-1.30695000	-3.85660600
C	-1.48172600	-0.97269300	0.73264600
C	0.67731400	-1.51151300	-8.35380600
C	2.56150400	9.16251600	-4.35632300
C	2.93448700	7.79676100	-3.75295400
C	2.18448800	7.37965200	-2.48222600
C	0.70156500	7.10654700	-2.69334000
C	-0.62705400	8.56711200	5.62641700
C	0.52639500	8.85903900	4.63640100
C	0.70209500	7.88632000	3.49390100
C	-0.24212500	7.77281100	2.46379100
C	1.84549500	7.08152900	3.41712100
C	-0.06460200	6.88045800	1.41088100
C	2.04650600	6.18982500	2.36590000
C	1.08105600	6.07084000	1.35944300
C	-5.02788500	5.89151300	5.56746300
C	-4.73518800	7.08392100	4.61765300
C	-5.12015600	6.72573400	3.19270900
C	-6.45963400	6.57155700	2.80712500
C	-4.14095800	6.41054700	2.24195600
C	-6.81712900	6.10609800	1.54215700
C	-4.47595400	5.95154400	0.96830400
C	-5.82095600	5.77846800	0.60482100
C	-3.36058900	2.96451900	3.75841800
C	-2.06659900	2.28064500	4.23474300
C	-0.97725000	3.28727000	4.63928700
C	-1.57905100	1.27822300	3.19068000
C	-0.49031600	4.19198000	3.51137000
C	3.24576200	-8.70772500	5.66060500
C	3.93491700	-7.43323600	5.13726300
C	3.27905000	-6.82874500	3.91331600
C	3.97662400	-6.70374700	2.70665800
C	1.95677600	-6.36473700	3.96968600
C	3.38748500	-6.10204600	1.59123500
C	1.35931300	-5.77725000	2.85432100
C	2.07674300	-5.63030600	1.66420800
C	11.21936900	-0.05643200	3.77458000
C	10.03748300	-0.29009300	2.82040500
C	8.69913900	0.32987300	3.26482600
C	8.75919500	1.86161900	3.28504300
C	7.55199000	-0.13376800	2.35685200
C	8.67656300	5.75093800	3.25169300
C	7.80236000	5.96498300	4.48231900
C	7.36676200	4.68836500	5.14159300
C	8.05415000	3.95734400	6.07800000
C	6.16894900	3.94262400	4.84336800
C	6.19332400	2.76036600	5.63386200
C	5.08359700	4.15805100	3.97738300
C	5.17783200	1.80052000	5.57018000
C	4.06749000	3.21531600	3.91665400
C	4.11987700	2.04512600	4.70262400
C	8.28656700	-5.66355400	-3.97042100
C	6.75120300	-5.74557500	-4.00380200
C	5.94171100	-4.91664900	-3.03188700
C	4.78546500	-4.28709400	-3.50785000
C	6.20421600	-4.82785600	-1.65767800
C	3.90849600	-3.62902500	-2.66029600
C	5.34472200	-4.14303500	-0.79278900
C	4.17434600	-3.54743500	-1.28950600
C	9.04634200	-5.57563900	2.62288300
C	7.70377900	-4.84597900	2.48288400
C	7.06693700	-4.39258500	3.81062500
C	8.01267000	-3.51747000	4.64460800
C	5.74439900	-3.65777500	3.55331900
C	9.13139800	3.17393500	-2.03403700
C	8.27164000	4.43510900	-2.00576300
C	8.66499900	2.17833700	-0.92634800
C	7.18666100	2.21797400	-0.67214700
C	6.49948500	3.04788700	0.18829100
C	5.07433000	1.79338900	-0.92334800
C	6.22384800	5.38460200	-2.99199400
C	5.08266400	4.62441500	-3.67585500
C	3.13581700	3.21332300	-3.35461600
C	1.88802700	3.01557700	-2.49780800
C	6.50167500	1.88945600	-7.22652100
C	5.99295100	0.93440400	-6.13756000
C	6.46509500	1.27438800	-4.74497500
C	1.90312000	-6.66112600	-5.59452900
C	1.27462800	-5.35364700	-5.15854400
C	2.09406200	-7.68964600	-4.45977700
C	2.86489000	-7.20754500	-3.21391200
C	1.99846000	-6.39272800	-2.24225000
C	3.48182100	-8.40335400	-2.47707300
C	-0.63829500	-4.38147800	-3.97795400
C	-1.42198800	-4.99638300	-2.82943200
C	-2.36643600	-4.60504900	-0.60377500
C	-1.92474700	-5.77099600	0.32082900
C	-1.40001800	-8.25841500	0.15510100
C	-3.24057500	-7.01632600	-7.02765800
C	-2.92158100	-5.51839700	-7.22113400
C	-4.00593500	-4.55320500	-6.85371700
C	-4.16082100	-3.86953500	-5.66749500
C	-5.72361500	-3.27972300	-7.09535300
C	2.27334400	2.04393000	1.09292100
C	3.15119400	1.01719800	1.53034400
C	2.77731300	-0.31663800	1.47600500
C	0.95920500	1.66440900	0.73326300
C	0.60757300	0.32025500	0.74111800
C	1.50108500	-0.69511000	1.05819300
C	-12.66621500	5.98392400	-2.83638200
C	-11.20052900	5.57100500	-2.56413900
C	-10.92435200	4.68717800	-1.33626800
C	-9.41800200	4.36855000	-1.21395400
C	-1.09825200	-1.84058500	1.90478900
O	-2.48644800	-0.83298100	-6.75081100
O	-0.76659400	1.99001600	-4.81594400
O	-5.25974000	-0.89556400	-3.99143600
O	2.61260900	-0.33299600	-6.63852800
O	0.06170100	7.50210900	-3.64856100
O	0.08383800	6.43727700	-1.71036900
O	1.22579800	5.19410500	0.33126700
O	-6.11940500	5.30943900	-0.63368100
O	3.32000900	-2.92586200	-0.43784400
O	8.32494300	5.25210400	-1.08507100
O	5.16420000	4.32320600	-4.86195000
O	0.76543600	3.73027700	-3.01021300
O	7.40271500	2.03401200	-4.51153300
O	5.80794000	0.60798900	-3.80957200
O	1.90616600	-4.30312000	-5.15057800
O	-2.02547800	-6.07369600	-2.96819900

O	-1.56502200	-5.55051600	1.48676500
O	2.69878500	3.29679100	1.00241700
O	-0.71318800	0.04640500	0.31472000
O	-9.05620200	3.17913400	-1.42247100
O	-8.66001700	5.35129000	-0.94349500
O	-2.54374100	-1.13829300	0.14926700
S	0.92637000	-1.58549300	-2.21753500
H	-2.95014400	6.22655300	-2.40158500
H	-1.46830100	5.49656700	-2.95040300
H	-0.79776900	3.10792300	-1.97611600
H	-4.56737800	2.05170300	-0.29417900
H	-2.96765800	4.19475500	-4.69172900
H	-5.49354200	-0.16136100	-6.90735700
H	-2.62812500	-1.59997100	-4.38872100
H	-2.19345500	-0.99499000	-2.77849600
H	-5.40924700	-1.67106900	-1.59023100
H	-3.70090700	-1.51936200	-1.31075700
H	-2.90558600	1.31582700	-3.52870300
H	-4.33545000	1.18564000	-5.23448500
H	1.74843400	-1.55446200	-8.55423600
H	-0.45923500	-1.10456400	-6.60983600
H	2.76132500	7.02184300	-4.50736800
H	4.00994900	7.77154300	-3.53678000
H	2.25971100	8.14790100	-1.69929400
H	2.64154300	6.47440500	-2.06837900
H	1.47777400	9.23882600	-4.45793300
H	0.39281300	9.87608900	4.24253100
H	1.46332800	8.88143300	5.20559900
H	2.59894200	7.15000300	4.19854800
H	-1.13679700	8.39129300	2.48399100
H	2.92961700	5.56315900	2.32461100
H	-0.80199500	6.79684200	0.62055500
H	0.68218100	5.95012000	-1.09053200
H	-1.60123100	8.84093300	5.20967200
H	-5.25760300	7.98813100	4.95496000
H	-3.66411100	7.31368800	4.64276300
H	-7.24896200	6.81227800	3.51674600
H	-3.08971600	6.52049700	2.49872100
H	-7.85759100	6.00437200	1.25122800
H	-3.69278700	5.73292900	0.25060300
H	-7.14043800	5.25895700	-0.78807100
H	-6.06661800	5.90355300	5.91310700
H	-2.32318000	1.71582600	5.14386200
H	-1.37041600	1.77485500	2.24100900
H	-0.65445200	0.78321000	3.50429500
H	-2.34046800	0.51139400	3.00831800
H	-1.37159500	3.90827800	5.45608000
H	-0.12298700	2.73768700	5.05782100
H	-1.31640300	4.72995200	3.03856600
H	0.20756700	4.94116500	3.88666100
H	0.02942500	3.62846100	2.73202100
H	-3.26278500	3.40852400	2.76372700
H	3.94465200	-6.68737300	5.94445600
H	4.98594500	-7.65063400	4.91268500
H	5.00166900	-7.06252700	2.64466800
H	1.38227900	-6.45714400	4.88688100
H	3.95637400	-5.98309200	0.67497500
H	0.32674800	-5.45123100	2.90269600
H	1.63012300	-5.14139400	0.80440800
H	3.26191900	-9.49249600	4.89705000

H	10.28534200	0.11673800	1.83024600
H	9.90391900	-1.37044700	2.67640300
H	8.48656000	-0.01967600	4.28810400
H	7.78507300	2.28688000	3.53134300
H	9.04328600	2.24467500	2.29818600
H	9.47729300	2.24617000	4.01489000
H	6.58969200	0.23540200	2.72676600
H	7.67473700	0.24710500	1.33779700
H	7.49853300	-1.22590200	2.30091500
H	12.15640000	-0.36544400	3.29591300
H	6.91429100	6.54559300	4.20424600
H	8.34908700	6.58207200	5.20737000
H	9.00132000	4.16594200	6.55525700
H	7.67497000	2.06780900	6.99253700
H	5.04461300	5.04829400	3.35561700
H	3.24491300	3.36323500	3.22823600
H	5.21741200	0.89654600	6.17176400
H	3.32115700	1.31683500	4.60812100
H	8.15582400	5.17837100	2.47891300
H	6.41861700	-5.48132000	-5.01451600
H	6.47870300	-6.80598700	-3.88033500
H	7.09416400	-5.29863900	-1.24492800
H	4.52930800	-4.33643500	-4.56224700
H	5.56795900	-4.06137700	0.26621700
H	2.99806500	-3.20465100	-3.04978300
H	2.60887400	-2.47649000	-0.96885500
H	8.65634900	-4.64162000	-4.08461100
H	7.83759000	-3.96812500	1.83600200
H	6.98720400	-5.49402200	1.96270100
H	6.84287400	-5.29997300	4.39335900
H	8.30956800	-2.62417200	4.08396800
H	7.52006400	-3.18014700	5.56345500
H	8.92392900	-4.05018200	4.93367900
H	5.91470100	-2.73318000	2.99104300
H	5.25883000	-3.38450400	4.49790300
H	5.04282000	-4.27308000	2.98579600
H	9.83460600	-4.89938300	2.96891400
H	8.96229500	1.16403900	-1.20754900
H	9.15981300	2.41425800	0.02011000
H	6.84786400	3.81244100	0.86216800
H	4.13957000	1.34459600	-1.22116200
H	9.05543400	2.68578700	-3.00599400
H	6.01874000	5.63111500	-1.94630600
H	7.37072700	3.76444800	-3.71964000
H	1.66481900	1.94171700	-2.43263600
H	2.05169900	3.36796000	-1.47595500
H	0.41620400	3.23427700	-3.77564600
H	3.71305700	2.27946900	-3.39799900
H	4.16812100	4.31522400	-1.89425900
H	4.90288300	0.83463700	-6.13495500
H	6.36373100	-0.08483000	-6.31624400
H	7.47586000	2.29643700	-6.94679600
H	1.11305100	-8.07364500	-4.14396500
H	2.62066800	-8.54758100	-4.89698000
H	3.68378800	-6.56026300	-3.54552000
H	1.10815800	-6.96115000	-1.94130400
H	1.67838700	-5.44065400	-2.66348800
H	2.57252200	-6.15638900	-1.34381500
H	4.15824300	-8.97029300	-3.12699400
H	4.05184200	-8.06668400	-1.60548500

H	2.70397500	-9.09197200	-2.12175400
H	1.26894800	-7.11666500	-6.36786200
H	-1.33927500	-3.78836400	-4.58234300
H	0.12829700	-3.69277200	-3.60805100
H	-0.47908500	-6.33966500	-4.68686900
H	-3.33949400	-4.84766600	-1.04490500
H	-2.47803000	-3.70980400	0.00679900
H	-0.85015800	-3.44328500	-1.64797300
H	-0.30892300	-8.14185000	0.09791700
H	-2.23302600	-6.93584100	-1.24478700
H	-2.64495300	-5.34074400	-8.26667300
H	-2.03417500	-5.28171100	-6.62307800
H	-3.61406600	-3.90240100	-4.73879100
H	-5.54234700	-2.32826300	-5.17777000
H	-6.58198900	-2.75716800	-7.49448600
H	-3.94526800	-7.39171100	-7.77412800
H	-10.58733200	6.47395100	-2.46661100
H	-10.82468200	5.04591300	-3.45241900
H	-11.24151500	5.21321800	-0.42700700
H	-11.47747200	3.74529300	-1.40084600
H	-13.31820900	5.11696700	-2.98582800
H	1.49531100	0.76746000	-4.53609900
H	1.77921800	-2.24467600	-4.41268100
H	2.80153800	-1.00788800	-3.69448600
H	1.83866300	4.38224800	0.59572000
H	4.14867600	1.28571300	1.85071600
H	0.24908700	2.42373900	0.42572700
H	-2.86868000	-4.43420500	2.22440500
H	-4.85107200	4.25201200	-1.42489500
H	6.16176300	0.84795500	-2.88407900
H	-0.35972600	-1.37898600	2.55461800
H	-2.02059200	-2.05330700	2.44639700
H	-0.69885100	-2.79444400	1.54478100
H	4.32991400	3.14809100	0.51410900
H	-0.62495800	-1.03969500	-3.99154600
H	-13.07998100	6.59701400	-2.02894400
H	-12.63921800	6.57312800	-3.75851300
H	-4.91059100	5.01305500	4.92394900
H	-4.36963400	5.84912300	6.44191900
H	-4.13234300	2.18812500	3.72552500
H	-3.60794900	3.76433600	4.46445400
H	-0.65968900	7.50121100	5.87428200
H	-0.42630200	9.18440400	6.50835400
H	-4.19320600	5.42029600	-4.32875800
H	-2.59202500	5.85723900	-5.05135500
H	2.92048500	10.01680500	-3.77236200
H	2.99663800	9.22262300	-5.35951600
H	8.94966200	6.72018100	2.82361300
H	9.58366700	5.19818200	3.51500200
H	6.33274800	6.32712800	-3.53863400
H	2.79997200	3.39747700	-4.38049200
H	10.17347500	3.45576600	-1.85440600
H	5.80059200	2.71945500	-7.36025900
H	6.61036100	1.30000800	-8.14210900
H	0.21582500	-0.76890000	-9.01314500
H	0.21652600	-2.48175400	-8.56340800
H	-4.70059000	1.19622300	-7.72049700
H	-4.50620900	-0.46320600	-8.35760600
H	-3.65737500	-7.18370200	-6.03104700
H	-2.28827500	-7.55177300	-7.10339600

H	2.85138400	-6.40181600	-6.07707800
H	8.61845500	-6.28166500	-4.81078800
H	8.68371800	-6.09840300	-3.04762400
H	-1.68927700	-8.94524100	-0.64746100
H	-1.58126700	-8.70521600	1.13886500
H	2.19820000	-8.49802600	5.89389700
H	3.77730700	-9.07919200	6.54274700
H	9.37348800	-5.99125000	1.66385600
H	8.99315100	-6.40361500	3.33750200
H	11.12986200	-0.65712800	4.68543100
H	11.32340100	1.00768200	4.01439000
H	-2.08738800	1.36679900	-0.57945800
H	-7.04976800	1.93965100	0.76036800
H	-8.87246300	0.61384100	-0.76160100
H	-9.43259500	-0.96655400	1.02069800
H	-9.46381800	-4.95475200	3.80079200
H	-9.69593300	-5.33204400	2.08578200
H	-8.05230300	-5.12144200	2.72357800
H	-10.15802100	-2.89697200	2.67621800
H	-7.34526500	-2.73844400	3.16585000
H	-7.51891300	2.74152900	-1.38348300
H	-8.99622200	-3.36080200	8.43794300
H	-8.16613200	-4.78856100	7.87330100
H	-2.49748000	-5.87396900	4.51238500
H	-3.36761500	-5.24313800	5.90009300
H	-5.76605300	-3.84509400	5.21791400
H	1.26150900	-1.73781300	0.91932100
H	-4.87954700	-3.15558500	1.17935300
H	-8.41895700	-2.90035200	4.94103800
H	0.10828700	-2.74265500	6.46924300
H	1.37012500	-2.69916900	8.92252800
H	4.11487200	-3.41974800	7.74113900
H	3.83290100	-2.34232500	9.12386100
H	3.45506700	-4.07527800	9.24727200
H	-10.08851700	-5.41424500	9.39916500
H	-9.36059300	2.24768600	3.50630500
H	-8.45721600	3.41168000	2.56852600
H	-7.62214400	2.06523400	3.41326800
H	-11.21874200	-4.59484800	8.29802400
H	-10.37227600	-6.04102300	7.76214300
H	-9.48953800	-3.58414700	0.39339000
H	-7.78361100	-3.43467600	0.81166700
H	-5.84733200	0.42636400	-1.10809100
H	-7.06121700	0.56810900	-2.37119800
H	2.16567600	-4.38081500	6.49642600
H	-2.08871600	-3.07201000	5.68593600
H	1.34804600	-4.98653800	7.92473200
H	-1.25368800	-3.71677300	4.28223500
H	2.61504800	-1.84262200	6.54953400
H	3.49794500	-1.09365200	1.70859600

E:HPA-3 (+4.2)

C	8.71722600	1.82600100	-2.86154000
C	8.88527400	0.83286500	-1.73875800
O	9.84267800	0.05264300	-1.71252400
N	7.86998100	0.78408800	-0.83948500
C	7.98460300	-0.04887300	0.35314500
C	7.64005200	-1.50930900	-0.00134800
O	6.55043000	-2.02352400	0.30137800
C	7.01974100	0.52344800	1.41312700

O	6.97637300	1.93109200	1.36100000
N	8.59311700	-2.19319100	-0.65632200
C	8.40820700	-3.58332700	-1.03731400
C	8.68771400	-3.90287200	-2.51162200
C	8.43347000	-5.39196700	-2.76072000
O	7.91812600	-3.08679900	-3.39278500
C	9.99031600	-5.76544100	-8.36744600
C	8.92700500	-4.78203900	-7.87630000
C	9.02739800	-4.46503300	-6.38948700
O	9.93450000	-4.86298500	-5.67697800
O	8.01292000	-3.70802300	-5.97881500
C	1.67521400	-3.98477400	-5.39604400
C	0.36332400	-4.52403900	-5.94698800
O	-0.03980900	-5.65568500	-5.68368400
C	1.69442900	-3.99397700	-3.85666800
C	3.02987700	-3.57554400	-3.32031700
C	4.31723600	-3.91721800	-3.66609300
N	3.17981700	-2.66768800	-2.28410200
C	4.51010800	-2.50632400	-2.04134900
N	5.22566700	-3.25249600	-2.86523000
N	-0.32152300	-3.67978100	-6.76309800
C	-1.64361100	-4.00555100	-7.26071000
C	-2.16640100	-2.84271100	-8.12729300
C	-3.68150200	-2.91953300	-8.28679800
O	-1.74938300	-1.57425900	-7.63305500
N	4.36501400	3.64903300	1.44803000
N	2.96725600	2.05714500	1.04107600
N	3.59513700	0.41090400	5.34987600
N	4.20660300	-1.72200100	1.70982800
N	0.30751400	-0.44709500	4.46215200
N	-0.46024300	-1.08255800	6.98006500
N	-6.47448700	3.16651800	-6.01903000
N	-6.13988200	1.64709600	1.32045000
N	-4.99408000	2.54200800	-0.33346500
N	-7.01241200	5.04080900	3.05804800
N	-3.63883300	4.54925600	2.92479300
N	-0.18216600	-5.46321500	4.71865500
N	0.96500700	-4.29245400	1.64659700
N	1.66411700	-7.05520400	0.18737100
N	4.73621400	-4.25649700	7.70971500
N	5.13995000	-3.40514100	5.69185300
C	3.54710600	5.04717000	4.31503400
C	2.97222800	5.23939600	2.89729100
C	3.16808800	4.02180900	2.05485400
C	2.28681000	3.00347400	1.79694900
C	4.22214100	2.47361500	0.83676200
C	4.71595300	-0.10391400	7.44179700
C	3.50527000	-0.26534200	6.53284800
C	2.67274400	0.17405400	4.24694700
C	1.22463500	0.52021600	4.66500400
C	2.83265400	-1.23632500	3.64040900
C	4.20154600	-1.45090100	3.02528400
C	-1.10276700	-0.29482500	4.75113300
C	-1.43199800	-0.49302800	6.24485100
C	-1.90667300	-1.35449500	3.96622400
C	0.84212400	-0.44283100	-0.41800300
C	-0.64080400	-1.42412600	8.37481400
C	-1.87611000	9.33396300	4.34927100
C	-2.36403900	8.02453700	3.70944800
C	-1.63508600	7.61666600	2.42197800
C	-0.26911100	6.93803000	2.66119600
C	1.22810300	8.51132300	-5.64401300
C	0.12266100	8.93464000	-4.64717000
C	-0.18746100	8.04369500	-3.46625800
C	0.68264000	7.86987700	-2.37788100
C	-1.44410700	7.43070100	-3.38072300
C	0.33108000	7.10450300	-1.26727300
C	-1.81812700	6.67891700	-2.27423100
C	-0.93625500	6.47839300	-1.19882500
C	5.45479700	5.56825700	-5.59339700
C	5.25473700	6.79471700	-4.66859600
C	5.65053100	6.44221000	-3.24789000
C	6.97283600	6.11326300	-2.91746700
C	4.68408900	6.30743400	-2.24411600
C	7.32234200	5.64151400	-1.65368600
C	5.01209500	5.84509600	-0.97031200
C	6.33399100	5.48646800	-0.66588200
C	3.61660400	2.75616700	-3.76838200
C	2.19014900	2.24568000	-3.98470500
C	1.20130400	3.37588600	-4.33027100
C	1.75157500	1.47063900	-2.73906500
C	1.03170000	4.44924700	-3.25466200
C	-3.70770900	-8.49040100	-5.60500600
C	-4.37833000	-7.17528300	-5.15095400
C	-3.94274300	-6.63779600	-3.80788900
C	-4.77839700	-6.75581000	-2.69044700
C	-2.70687800	-5.99408400	-3.65760900
C	-4.40029600	-6.22786700	-1.45378100
C	-2.32771000	-5.46379400	-2.42508800
C	-3.17529000	-5.57400600	-1.32004000
C	-11.12217800	0.64418200	-3.71661000
C	-9.96613500	0.31316500	-2.76127100
C	-8.57545900	0.79529100	-3.21216900
C	-8.49628100	2.32596000	-3.24664200
C	-7.48070400	0.22661600	-2.29844000
C	-8.22218700	6.28474400	-3.22303400
C	-7.34135600	6.43996400	-4.45784300
C	-6.76152300	5.12909000	-4.92598100
C	-7.23820900	4.32150700	-5.92691600
C	-5.62946000	4.42956600	-4.35384800
C	-5.48056500	3.20165300	-5.06341600
C	-4.71842400	4.72348700	-3.32611300
C	-4.47092300	2.27951400	-4.76700800
C	-3.69545800	3.82202300	-3.04085300
C	-3.58151300	2.60767100	-3.74933100
C	-8.51028400	-5.10960600	4.03572000
C	-6.97639100	-5.19894300	4.00185100
C	-6.31098600	-4.20368400	3.08401300
C	-5.77915400	-3.02197900	3.61276800
C	-6.20303900	-4.41217500	1.70364100
C	-5.11268900	-2.10546000	2.81065700
C	-5.51046400	-3.51449600	0.88916300
C	-4.92114800	-2.37785300	1.45350000
C	-9.29052100	-4.99549500	-2.55505500
C	-7.89948800	-4.36226200	-2.45593800
C	-7.27234500	-4.01796100	-3.81935100
C	-8.16013700	-3.08872300	-4.65869800
C	-5.88266400	-3.40303200	-3.63356300
C	-8.78537800	3.77157000	2.05046000
C	-7.82499600	4.94401400	1.97566900

C	-8.41486600	2.73077400	0.96673800
C	-6.96022700	2.54151800	0.65271200
C	-6.25071100	3.10776600	-0.37980400
C	-4.96990400	1.66402500	0.70180400
C	-5.77108300	5.78615700	3.01199800
C	-4.71824600	4.89152100	3.66627900
C	-2.82194900	3.42857700	3.36937900
C	-1.60654600	3.14087900	2.47051000
C	-6.24742600	2.32797000	7.25922900
C	-5.80609100	1.34785900	6.16363200
C	-6.29328500	1.69524500	4.77753300
C	-2.19427200	-6.49624200	5.63776900
C	-1.35414000	-5.25033400	5.39023900
C	-2.54159400	-7.28402400	4.35732300
C	-3.44693500	-6.54444700	3.35220000
C	-2.77671800	-5.34766200	2.66358500
C	-3.97502100	-7.52683300	2.29977500
C	0.50618700	-4.37883100	4.03996800
C	1.20225700	-4.94005400	2.80544500
C	1.71032300	-4.60291100	0.42553100
C	1.23206800	-5.86909000	-0.32521100
C	0.97995100	-8.31305800	-0.11937400
C	2.92338100	-7.16494200	7.05168600
C	2.68585100	-5.65116200	7.23620500
C	3.80198700	-4.74636000	6.81560400
C	4.04103400	-4.22830700	5.56026600
C	5.50845700	-3.45111900	7.00227400
C	-1.92774400	-0.93457200	-3.92009800
C	-3.08177200	-1.45066500	-3.33268500
C	-3.01064700	-2.00673900	-2.06118800
C	-0.70530600	-0.97843300	-3.25304000
C	-0.68589600	-1.49702100	-1.95698100
C	-1.80770700	-2.03209800	-1.34953500
C	13.11891300	5.21313100	2.77982100
C	11.64781300	4.84414100	2.50902300
C	11.36763900	3.99633500	1.25714500
C	9.85873600	3.71283700	1.15218800
C	-0.18777400	0.58415300	-0.10573000
O	2.52653100	-0.94523700	6.83310100
O	0.95871200	1.62872900	5.12774500
O	5.24494500	-1.38724900	3.69819900
O	-2.55123300	-0.20227600	6.66634600
O	0.37468800	7.19965600	3.68462200
O	0.13526700	6.12748400	1.73288100
O	-1.32255200	5.71251700	-0.17518400
O	6.61941100	5.00203000	0.57035800
O	-4.14780600	-1.55625400	0.68073100
O	-7.74905800	5.67775900	0.98819400
O	-4.91138700	4.44272400	4.79571100
O	-0.36468900	3.27354100	3.14154700
O	-7.23290800	2.44671900	4.54206200
O	-5.64323600	1.01925300	3.83563400
O	-1.71761000	-4.13113700	5.73262900
O	1.94837700	-5.92774200	2.89400500
O	0.52383600	-5.78420000	-1.32421900
O	-2.05713400	-0.36483100	-5.17033400
O	0.54172100	-1.46914800	-1.26509400
O	9.45278800	2.59528000	1.57313000
O	9.14317300	4.66023900	0.70434300
O	1.96467700	-0.47542700	0.07154800

S	-1.37801500	-1.59624500	2.20901600
H	3.43395800	6.10695800	2.41214600
H	1.90176100	5.45443900	2.93228100
H	1.25667000	2.90676400	2.12488900
H	5.00299800	1.96139200	0.30655100
H	3.33253300	4.03015100	4.66444200
H	5.60754500	-0.45612300	6.91268400
H	2.69190900	-1.97034300	4.43888400
H	2.07151400	-1.39764300	2.87188200
H	5.10936100	-1.83950400	1.23862600
H	3.36381600	-1.63327600	1.15960200
H	2.91977600	0.92210200	3.48807500
H	4.51222100	0.73399900	5.07900100
H	-1.70948800	-1.40392600	8.59175100
H	0.47481300	-1.15785100	6.59788300
H	-2.25149800	7.21192200	4.43497700
H	-3.44035300	8.10788100	3.50128200
H	-1.46088500	8.49408500	1.78191600
H	-2.25603600	6.94229000	1.82610000
H	-0.78763900	9.31327200	4.43429900
H	0.37223700	9.94469600	-4.29164200
H	-0.80744900	9.04991400	-5.21713900
H	-2.15657100	7.56450800	-4.19295100
H	1.65781900	8.35444300	-2.39100500
H	-2.80905000	6.25183300	-2.20659900
H	1.00883000	6.98800200	-0.42806700
H	-0.67061200	5.88671900	0.69416000
H	2.22820200	8.71816600	-5.24839400
H	5.82407200	7.65594500	-5.04138800
H	4.20021200	7.09144300	-4.67600600
H	7.75154900	6.21545000	-3.67071700
H	3.64908600	6.56431300	-2.45796000
H	8.35057200	5.40241000	-1.40304900
H	4.24220400	5.76601500	-0.21071100
H	7.62158000	4.78229500	0.66995200
H	6.48843300	5.50399500	-5.94912300
H	2.19444100	1.55057900	-4.83854700
H	1.89448100	2.07678400	-1.83958100
H	0.69258200	1.20823800	-2.78644600
H	2.33679800	0.55291800	-2.61575200
H	1.53229500	3.84900000	-5.26470500
H	0.21946300	2.92860600	-4.54830400
H	1.98234400	4.93212000	-3.01370100
H	0.34623500	5.23073000	-3.58647600
H	0.62510700	4.04405900	-2.32343000
H	3.68346200	3.24815000	-2.78930000
H	-4.19670800	-6.41339900	-5.92035900
H	-5.46417600	-7.33006400	-5.12795200
H	-5.74162600	-7.25042600	-2.79487500
H	-2.02789800	-5.91534300	-4.50265400
H	-5.06314000	-6.31839400	-0.59874700
H	-1.36207300	-4.98815200	-2.31370500
H	-2.88072100	-5.15783100	-0.36234100
H	-3.78554000	-9.25676300	-4.82753300
H	-10.17081000	0.75221600	-1.77508100
H	-9.93668800	-0.77361600	-2.60679900
H	-8.39894000	0.41750200	-4.23237900
H	-7.48580300	2.66213800	-3.48227500
H	-8.76023800	2.74155300	-2.26762700
H	-9.16987000	2.76448000	-3.98863300

H	-6.48505400	0.52808100	-2.64055200
H	-7.59197900	0.59290000	-1.27316200
H	-7.51297300	-0.86750300	-2.26580000
H	-12.07930600	0.39944300	-3.24009000
H	-6.51763400	7.12981900	-4.23484500
H	-7.91406300	6.90050900	-5.27158600
H	-8.07693500	4.47598300	-6.59093000
H	-6.65119100	2.39948500	-6.64500300
H	-4.80203000	5.65173400	-2.76961800
H	-2.95760500	4.07465000	-2.28444400
H	-4.36530700	1.34844300	-5.31522000
H	-2.78001900	1.91735900	-3.50530400
H	-7.73084700	5.69744300	-2.44250400
H	-6.58943700	-5.04263500	5.01537400
H	-6.68902100	-6.22000000	3.71991800
H	-6.63199900	-5.30941900	1.26163600
H	-5.87388400	-2.82005900	4.67649500
H	-5.37585200	-3.71711200	-0.16809800
H	-4.74554400	-1.17714900	3.22857100
H	-3.28875900	-1.42334900	1.17294500
H	-8.84968500	-4.07731300	4.15500000
H	-7.94982700	-3.45356300	-1.84056600
H	-7.21901400	-5.04384400	-1.93202200
H	-7.15157800	-4.96308300	-4.37110600
H	-8.34961900	-2.14857600	-4.12812000
H	-7.67212800	-2.83911500	-5.60767100
H	-9.12826600	-3.54092300	-4.89386000
H	-5.95874800	-2.44849000	-3.09992200
H	-5.40713400	-3.20931600	-4.60208200
H	-5.22061300	-4.06131300	-3.06740300
H	-10.03765000	-4.27314800	-2.89913000
H	-8.82723200	1.76067000	1.26423800
H	-8.89785300	2.99418000	0.02137400
H	-6.52159100	3.83595000	-1.12471200
H	-4.12191300	1.04365700	0.94829100
H	-8.74341600	3.30241100	3.03309200
H	-5.55067500	6.01995000	1.96678800
H	-7.06526300	4.31565800	3.77164500
H	-1.71448800	2.12836600	2.04955100
H	-1.55498900	3.84660000	1.63566400
H	-0.30132300	2.68864000	3.91725400
H	-3.46972800	2.54357300	3.42777600
H	-3.54384300	4.88137700	1.97557000
H	-4.72243100	1.20054400	6.14003000
H	-6.21911500	0.34709600	6.35676900
H	-7.19652900	2.79485100	6.98570100
H	-1.62457500	-7.60862200	3.84510300
H	-3.04944300	-8.20653200	4.66776100
H	-4.30800900	-6.15839400	3.91332100
H	-1.84626700	-5.64595900	2.16507100
H	-2.55458900	-4.53700600	3.35582900
H	-3.44515500	-4.92736200	1.90988300
H	-4.47090500	-8.39006600	2.75940000
H	-4.69511900	-7.03109200	1.64297500
H	-3.15981400	-7.90261900	1.66930500
H	-1.65966300	-7.15386300	6.33582700
H	1.27034000	-3.92194200	4.68236500
H	-0.21749600	-3.60739700	3.77070200
H	0.09945200	-6.39765000	4.46022300
H	2.77226500	-4.69085700	0.67771500

H	1.57137100	-3.76991600	-0.26011000
H	0.27880200	-3.53094100	1.65157300
H	-0.10555400	-8.14716500	-0.06505800
H	2.05925900	-6.97704700	1.12259400
H	2.45688100	-5.44100900	8.28635100
H	1.79057500	-5.38451300	6.66300100
H	3.57070500	-4.41351900	4.60686000
H	5.44644000	-2.73596500	4.98338600
H	6.34256400	-2.88090100	7.38642300
H	3.60800500	-7.57736200	7.79742500
H	11.05581300	5.76349000	2.42997300
H	11.25653200	4.30301900	3.38012400
H	11.69272500	4.53883300	0.36191800
H	11.90691400	3.04530700	1.30992000
H	13.74461100	4.32545500	2.92181500
H	-1.43072000	0.71145900	4.47658300
H	-1.83394800	-2.30721200	4.49303100
H	-2.94823000	-1.04812600	4.00572400
H	-1.25904500	0.13608800	-5.38909200
H	-4.02096900	-1.39291000	-3.86722800
H	0.20269800	-0.59776300	-3.70372900
H	2.42152000	-2.20463500	-1.79620300
H	5.24524300	4.18942900	1.36672500
H	-6.01305000	1.23257900	2.91443300
H	-0.88681800	0.75341400	-0.92240300
H	-0.74486900	0.18626500	0.76862200
H	0.29513200	1.51691800	0.18173700
H	-4.26564000	2.71371000	-1.01250000
H	0.52388900	-1.22507200	3.85633400
H	13.54712900	5.81089700	1.96797500
H	13.13237800	5.80571000	3.70009400
H	5.28607800	4.70059900	-4.94668600
H	4.79154400	5.56413200	-6.46513800
H	4.33909800	1.93303600	-3.73531600
H	3.91067200	3.53652300	-4.47771400
H	1.19349600	7.44459600	-5.88840700
H	1.06220700	9.13701300	-6.52719600
H	4.63356900	5.18032600	4.30749700
H	3.06553200	5.71778000	5.03496500
H	-2.18386600	10.20703600	3.76376100
H	-2.30256700	9.42406300	5.35388300
H	-8.43334600	7.27006700	-2.79757300
H	-9.16289500	5.78780500	-3.48096200
H	-5.81922100	6.73526500	3.55588500
H	-2.47124500	3.59492200	4.39370800
H	-9.80368800	4.12812800	1.86624400
H	-5.49570100	3.11335700	7.38741500
H	-6.38862600	1.74924600	8.17719000
H	-0.13079900	-0.70920800	9.02989400
H	-0.23947500	-2.42001200	8.58600300
H	4.89232700	0.94342800	7.71134300
H	4.59818600	-0.69878800	8.35473200
H	3.32492500	-7.36098700	6.05400200
H	1.94012100	-7.64016200	7.13300300
H	-3.12266600	-6.17698300	6.12305300
H	-8.87646600	-5.70385700	4.87902700
H	-8.93748500	-5.52231800	3.11563200
H	1.22902800	-9.01365000	0.68412400
H	1.12871900	-8.77301000	-1.10265300
H	-2.65024000	-8.34675500	-5.84338200

H	-4.26498400	-8.83097100	-6.48403700
H	-9.63880400	-5.38706200	-1.59336600
H	-9.29169300	-5.82737500	-3.26711000
H	-11.07382400	0.03628000	-4.62581100
H	-11.16104800	1.71194500	-3.95957600
H	2.59877200	1.15694900	0.72035500
H	7.36151700	1.64656600	-0.66033800
H	9.01761500	0.00195000	0.71523900
H	9.37263000	-1.63944400	-1.01534700
H	8.73352900	-5.65562100	-3.77542100
H	9.00827700	-6.01147400	-2.06325800
H	7.37059700	-5.61877900	-2.62232500
H	9.73385100	-3.67337300	-2.74700100
H	6.94903600	-3.16128400	-3.15438900
H	7.91751300	2.28385900	1.50554700
H	8.99059700	-3.82848900	-8.41548000
H	7.91456900	-5.15654700	-8.06690600
H	0.91974900	-3.31894800	-3.47852200
H	1.41884300	-4.98972700	-3.49589900
H	4.64807600	-4.59216600	-4.44174700
H	-1.76549300	-2.41617500	-0.33716100
H	4.91433100	-1.86979200	-1.26904800
H	8.08276800	-3.49109300	-4.99537100
H	-0.06925900	-2.70263500	-6.82683900
H	-1.69121500	-2.91244800	-9.11304700
H	-4.17340100	-2.80987800	-7.31277300
H	-4.03793500	-2.12376500	-8.94593000
H	-3.98560100	-3.88489800	-8.70551800
H	9.88870700	-5.93979500	-9.44296200
H	9.56289700	1.66434800	-3.53477000
H	8.73625300	2.88992000	-2.59985000
H	7.81582600	1.59532700	-3.43675600
H	10.99346000	-5.38131600	-8.16650800
H	9.90156400	-6.72440700	-7.84969500
H	9.06207200	-4.21908200	-0.42490800
H	7.37637900	-3.84395500	-0.79281700
H	6.01214100	0.15532600	1.20537300
H	7.29815500	0.13896300	2.40141400
H	-2.32619400	-4.21591200	-6.42544700
H	2.46978600	-4.63870600	-5.77053600
H	-1.59245300	-4.90108300	-7.88914300
H	1.88638900	-2.97769100	-5.77199800
H	-2.18259900	-1.39113100	-6.78039500
H	-3.90186600	-2.37950300	-1.57918500

E:HPA-4 (+5.1)

C	-8.59460500	2.31261400	2.74211000
C	-8.77527500	1.31712800	1.62370500
O	-9.80501800	0.64610600	1.50612000
N	-7.68575300	1.14543900	0.83176400
C	-7.77272400	0.32025000	-0.36619900
C	-7.59162200	-1.15861500	0.03633000
O	-6.51736800	-1.77241800	-0.08220000
C	-6.70381700	0.81812100	-1.35560200
O	-6.59838600	2.22472100	-1.32571700
N	-8.70120100	-1.72757800	0.53794500
C	-8.73568900	-3.09799000	1.01193700
C	-9.18223800	-3.25558000	2.47177200
C	-9.20124200	-4.74061200	2.84426900
O	-8.35976900	-2.49659600	3.35544800
C	-10.24259000	-5.01633000	8.49872700
C	-9.12496800	-4.16113400	7.90246200
C	-9.37897600	-3.76297000	6.45589300
O	-10.40975800	-4.02331200	5.85866800
O	-8.34598300	-3.10290800	5.93397100
C	-1.75826700	-3.84315800	5.31598800
C	-0.61894200	-4.44141400	6.13330300
O	-0.41232900	-5.65347200	6.18637700
C	-2.93532700	-4.81762200	5.14681300
C	-3.98721100	-4.28788300	4.22795900
C	-5.25498400	-3.79812000	4.43440200
N	-3.76756200	-4.15294200	2.86764600
C	-4.88354000	-3.60619000	2.31269300
N	-5.80121600	-3.37736400	3.23687500
N	0.16612700	-3.53638700	6.77641300
C	1.46337500	-3.90303400	7.31482600
C	2.07629100	-2.71274300	8.05773400
C	3.51510400	-3.00521800	8.47543500
O	1.99082900	-1.52909700	7.27313300
N	-3.99076100	3.70791200	-1.54317600
N	-2.39049400	2.34971400	-1.03154500
N	-3.71400500	0.25442600	-5.33905100
N	-4.22571700	-1.69495100	-1.55709300
N	-0.51642200	-0.67971100	-4.57991600
N	0.34029200	-1.38551900	-7.01639500
N	7.01429200	2.94772200	5.96918300
N	6.29668800	1.06821400	-1.43023500
N	5.06757700	2.16665000	0.03345900
N	7.26460800	4.51118000	-3.30604600
N	3.88505400	4.18009600	-3.15787000
N	-0.13136800	-5.69314100	-4.63359100
N	-1.39782400	-4.41215100	-1.59335600
N	-2.03290800	-7.04586800	-0.10308000
N	-4.95472200	-4.26437000	-7.59998300
N	-5.35371000	-3.44360300	-5.56859500
C	-3.28815100	5.02093500	-4.54592100
C	-2.76425400	5.33874600	-3.12663400
C	-2.85230900	4.17777100	-2.19325000
C	-1.84882000	3.30909300	-1.86149500
C	-3.69239800	2.61208100	-0.83841800
C	-4.73423900	-0.16246100	-7.49830900
C	-3.57968300	-0.38209200	-6.53897100
C	-2.83311300	-0.00138100	-4.20653300
C	-1.36999800	0.35609800	-4.54837300
C	-3.00822000	-1.42681800	-3.63156800
C	-4.32375500	-1.56085500	-2.89457700
C	0.91005600	-0.58048400	-4.76985400
C	1.33787600	-0.93146000	-6.21660500
C	1.66429000	-1.51560000	-3.78970600
C	2.61986700	-0.71837800	4.05084400
C	0.54310600	-1.79026800	-8.39605600
C	2.35065400	9.01571900	-4.73111000
C	2.77012900	7.70186900	-4.05178900
C	2.02746300	7.37349500	-2.75109000
C	0.60399600	6.85410900	-2.97490700
C	-0.75806200	8.68626300	5.28899800
C	0.40003300	8.97873600	4.30167800
C	0.63726700	8.02681300	3.15217900
C	-0.25413500	7.89429600	2.07521300

C	1.83398100	7.30143400	3.07382400
C	0.02365200	7.07802800	0.98436400
C	2.13746700	6.48790200	1.98513600
C	1.24131400	6.34314400	0.89709300
C	-5.13250000	5.96771200	5.34303800
C	-4.87343900	7.14181700	4.36369400
C	-5.26904900	6.74952400	2.95051300
C	-6.59914200	6.46464200	2.60809500
C	-4.29683200	6.54766900	1.96277000
C	-6.95083400	5.97734900	1.34936000
C	-4.62712200	6.06850500	0.69574400
C	-5.95873200	5.76109900	0.37672000
C	-3.44991800	3.00445700	3.60894600
C	-2.06589100	2.38788400	3.85192800
C	-0.99207500	3.45558400	4.13952300
C	-1.69478100	1.49943100	2.65661500
C	-0.88166200	4.56334200	3.09285000
C	3.28559000	-8.54171300	5.80516500
C	3.95726600	-7.28570900	5.21912200
C	3.31126100	-6.80981900	3.93636300
C	3.99564400	-6.86506400	2.71692600
C	1.99779500	-6.31994500	3.94667000
C	3.39251100	-6.42845000	1.53460900
C	1.38964800	-5.88986200	2.76839700
C	2.08709900	-5.93751500	1.55842700
C	11.15912300	0.12648500	3.60274700
C	10.01953100	-0.19433400	2.62226200
C	8.61499300	0.30506100	3.00952800
C	8.53446400	1.83485200	3.00175900
C	7.55487600	-0.27996100	2.06566900
C	8.55523000	5.89075500	2.92755900
C	7.68807000	6.13299800	4.15789800
C	7.15072300	4.89074400	4.81785500
C	7.86429100	3.93808500	5.50181400
C	5.76939000	4.46810100	4.86444200
C	5.72075100	3.24270300	5.58886600
C	4.56543000	5.02092200	4.39270500
C	4.51944000	2.57377900	5.85033600
C	3.36728100	4.37328100	4.66465000
C	3.34692900	3.16319300	5.39023700
C	8.22484300	-5.73607400	-3.95017500
C	6.68354200	-5.74034500	-3.90791800
C	6.02830600	-4.71728300	-3.00269400
C	5.49457500	-3.54535700	-3.55203400
C	5.88461500	-4.90626200	-1.61999500
C	4.79828700	-2.62489500	-2.77595900
C	5.16610500	-4.00340500	-0.83212800
C	4.57502800	-2.87592400	-1.41810000
C	9.03234300	-5.44525800	2.63178400
C	7.66564500	-4.76807200	2.45176700
C	6.97562700	-4.29378300	3.74421600
C	7.86973300	-3.36471800	4.57626700
C	5.63831500	-3.61583700	3.41791200
C	8.97074200	3.18763700	-2.25303700
C	8.06546100	4.40358500	-2.21549500
C	8.54823000	2.19567800	-1.14263600
C	7.07959100	2.05830800	-0.85579800
C	6.31808400	2.75239400	0.05673500
C	5.09964000	1.15396200	-0.86905300
C	6.06021300	5.31557800	-3.28359600

C	4.96656100	4.46032000	-3.92154400
C	2.99129900	3.10472700	-3.55802000
C	1.71240000	3.03789800	-2.72032200
C	6.34140600	1.69901500	-7.41470800
C	5.85665900	0.77773300	-6.28493200
C	6.38881000	1.11746500	-4.91151500
C	1.83978900	-6.84346100	-5.49500900
C	1.09918300	-5.54337100	-5.21609200
C	2.15375300	-7.64718200	-4.21445200
C	3.04577400	-6.92487500	-3.18178400
C	2.31265200	-5.84368400	-2.37321500
C	3.67836700	-7.94619800	-2.22872700
C	-0.77111600	-4.59021900	-3.94522800
C	-1.52609400	-5.11937800	-2.73469600
C	-2.27961500	-4.63839900	-0.44821200
C	-1.92307500	-5.83375700	0.47433500
C	-1.40507700	-8.30119500	0.32461700
C	-3.31052700	-7.29075900	-6.87743800
C	-3.00660100	-5.79743200	-7.12278600
C	-4.07055600	-4.83137900	-6.70142700
C	-4.30680300	-4.33273900	-5.43721600
C	-5.69530700	-3.43442600	-6.88673900
C	0.87129900	1.07789000	0.28905100
C	1.08401800	2.43441100	0.55861900
C	1.98916300	2.81682300	1.54580300
C	1.55970900	0.09609700	1.02575400
C	2.43644100	0.51212100	2.02118700
C	2.68313300	1.85709700	2.28705300
C	-12.83317700	5.73653200	-3.00106200
C	-11.35158800	5.35416400	-2.73978500
C	-11.03272200	4.42848900	-1.55186700
C	-9.50946500	4.17760200	-1.41582300
C	1.27394300	-0.15204600	4.43472500
O	-2.60880600	-1.08598300	-6.80920800
O	-1.05593000	1.53206700	-4.73598700
O	-5.41584000	-1.52663900	-3.48158900
O	2.51662700	-0.83445200	-6.54926700
O	-0.03435900	7.12995100	-3.98579500
O	0.09454600	6.12167500	-2.00762500
O	1.53677300	5.56368900	-0.12303400
O	-6.24432200	5.26725300	-0.85597000
O	3.80234100	-2.04602700	-0.65098300
O	8.01645500	5.16639700	-1.24865700
O	5.12088900	4.00156100	-5.05251000
O	0.58731300	3.48056400	-3.46670200
O	7.33956000	1.86095100	-4.69997200
O	5.75598800	0.44155000	-3.95724300
O	1.58178200	-4.44068300	-5.44394800
O	-2.24150000	-6.13091700	-2.82824100
O	-1.62614100	-5.64073900	1.65964300
O	-0.04576700	0.74309600	-0.66643400
O	3.09196000	-0.45221700	2.81077200
O	-9.09281200	2.99669300	-1.57262100
O	-8.79823500	5.19994700	-1.17114000
O	3.28582100	-1.42595200	4.78686900
S	0.90581300	-1.77373700	-2.11228700
H	-3.30950900	6.19425300	-2.71181400
H	-1.71525200	5.63130700	-3.16250400
H	-0.82704200	3.30274600	-2.21482700
H	-4.38750500	2.06385300	-0.22912300

H	-3.09779800	3.97492700	-4.80642600
H	-5.66506900	-0.44147500	-6.99113200
H	-2.99106300	-2.14333900	-4.45767300
H	-2.17973700	-1.64605100	-2.95121700
H	-5.08875200	-1.68539100	-0.99152500
H	-3.32691700	-1.60396700	-1.11053100
H	-3.10461400	0.73070200	-3.44064800
H	-4.60988500	0.66328500	-5.11973700
H	1.61552000	-1.82999200	-8.58781200
H	-0.62042100	-1.30339700	-6.70388900
H	2.61378700	6.87239500	-4.74969600
H	3.84835000	7.73129400	-3.84426500
H	1.95181500	8.26127400	-2.10678400
H	2.57456700	6.63416900	-2.16061100
H	1.26325600	9.05614000	-4.81647100
H	0.25542400	9.99825500	3.91469300
H	1.32639900	9.02013900	4.88839600
H	2.55737100	7.39403500	3.88314700
H	-1.18847200	8.45476900	2.08473300
H	3.08858600	5.96506000	1.93311700
H	-0.68273100	7.00115500	0.16424400
H	0.76951000	5.87007500	-1.17700000
H	-1.73486500	8.93985200	4.86520300
H	-5.40729300	8.04336400	4.69099000
H	-3.80719400	7.39272500	4.36874700
H	-7.38415200	6.61726000	3.34622600
H	-3.25478700	6.76857600	2.18159600
H	-7.98576600	5.77854800	1.08973900
H	-3.85148900	5.94153000	-0.05119600
H	-7.25884300	5.16974400	-1.00316600
H	-6.16708600	5.97063300	5.70207800
H	-2.12548700	1.74381300	4.74192400
H	-1.64891700	2.09227700	1.73863700
H	-0.71686000	1.03005900	2.77980500
H	-2.43915900	0.70737400	2.50951200
H	-1.20878200	3.90798700	5.11707300
H	-0.01762100	2.95732600	4.24366700
H	-1.81249100	5.13025100	3.01211400
H	-0.09285000	5.27280100	3.34835500
H	-0.64879100	4.17124400	2.10037500
H	-3.48011600	3.46235400	2.61169800
H	3.91912800	-6.48217900	5.96648600
H	5.01961700	-7.48657500	5.03743800
H	5.01630100	-7.24025500	2.69562300
H	1.43874600	-6.27427700	4.87716100
H	3.94287500	-6.45943700	0.59995900
H	0.36668300	-5.53227300	2.78522800
H	1.62146000	-5.58425900	0.64336400
H	3.30189300	-9.35572400	5.07213600
H	10.26576700	0.22517800	1.63683200
H	9.98008600	-1.28274100	2.48279100
H	8.39939800	-0.04872100	4.03051300
H	7.53692900	2.18257100	3.28190000
H	8.74667100	2.22153000	1.99905100
H	9.24705200	2.29345300	3.69273900
H	6.54491900	0.01711900	2.36559800
H	7.70552500	0.07140800	1.03992600
H	7.58807400	-1.37341700	2.05020300
H	12.11369900	-0.17439700	3.15203500
H	6.83873500	6.76712400	3.87901000

H	8.26223200	6.71863900	4.88882700
H	8.92593400	3.87864400	5.69321100
H	7.30817300	2.09116500	6.40747700
H	4.56738200	5.94850100	3.82868900
H	2.43823900	4.79966400	4.30280700
H	4.49884700	1.63474800	6.39599900
H	2.39440300	2.68262700	5.59179000
H	8.03362400	5.29641800	2.17165100
H	6.30766400	-5.57541200	-4.92447800
H	6.34894000	-6.74677900	-3.62331100
H	6.31025200	-5.79248600	-1.15271900
H	5.60888100	-3.35014800	-4.61509300
H	5.01527300	-4.17724800	0.22809100
H	4.43795900	-1.71033900	-3.22770200
H	2.94233100	-1.89233000	-1.11734600
H	8.61957600	-4.72788700	-4.10261400
H	7.77688700	-3.91037700	1.77473500
H	6.98519300	-5.46103500	1.93969600
H	6.76571500	-5.18846100	4.35235400
H	8.15468900	-2.47871200	3.99893800
H	7.33977200	-3.01771500	5.46996100
H	8.78919500	-3.86091600	4.90397200
H	5.78701800	-2.73672600	2.78195500
H	5.12437100	-3.27108600	4.31957100
H	4.96413500	-4.29773000	2.89257700
H	9.80067800	-4.73587300	2.95393300
H	8.92302700	1.20227300	-1.41070900
H	9.04748400	2.46466600	-0.20736700
H	6.55405600	3.59024600	0.69132400
H	4.26881100	0.48713900	-1.04778200
H	8.91102700	2.69404600	-3.22280300
H	5.84967600	5.59017800	-2.24658900
H	7.28434900	3.76446200	-3.99813500
H	1.55714600	2.01674700	-2.35039300
H	1.78078200	3.69768100	-1.85085600
H	0.26161600	2.76171300	-4.03807600
H	3.54610700	2.15656100	-3.53558300
H	3.86242000	4.48294800	-2.19372900
H	4.76607400	0.70816900	-6.23379900
H	6.18917500	-0.25498700	-6.46104400
H	7.31375800	2.12641300	-7.15910400
H	1.22205000	-7.96667600	-3.72579700
H	2.65723800	-8.57015000	-4.52904900
H	3.85739100	-6.42871100	-3.72909800
H	1.41427300	-6.25487900	-1.89429700
H	2.02350200	-4.98785400	-2.98185700
H	2.96744900	-5.45273500	-1.59139800
H	4.22976000	-8.72184300	-2.77293700
H	4.37495800	-7.45081500	-1.54673700
H	2.91285300	-8.44258400	-1.61901000
H	1.24779300	-7.46287500	-6.18096400
H	-1.50503900	-4.08526000	-4.59014800
H	-0.01782700	-3.85593900	-3.65223300
H	-0.50045500	-6.61231200	-4.43583600
H	-3.30245500	-4.76611600	-0.82044000
H	-2.24194500	-3.74651600	0.17750200
H	-0.71834900	-3.64101900	-1.58404100
H	-0.31330900	-8.18802100	0.26124000
H	-2.24599000	-6.99897800	-1.10149800
H	-2.80173500	-5.63188100	-8.18541600

H	-2.08254100	-5.55320400	-6.58584600
H	-3.86867500	-4.57001800	-4.48001400
H	-5.64110800	-2.77919500	-4.84661900
H	-6.48983000	-2.81088300	-7.27162100
H	-4.01791400	-7.69864200	-7.60423400
H	-10.76566300	6.27076400	-2.60994200
H	-10.96396200	4.87780600	-3.65043500
H	-11.38163900	4.89346300	-0.62064100
H	-11.53741100	3.46365500	-1.66046800
H	-13.47124800	4.85512500	-3.12507300
H	1.18903300	0.45982200	-4.59109000
H	1.78217500	-2.49728400	-4.25062600
H	2.66040900	-1.09750700	-3.67070000
H	0.25892400	-0.07114600	-1.19298900
H	0.56840200	3.20173700	-0.00461100
H	1.41771900	-0.95948300	0.82804100
H	-2.94047800	-4.51865600	2.38859500
H	-4.92019400	4.16110400	-1.49549600
H	6.14959300	0.63673200	-3.04535500
H	1.25135200	0.93065300	4.30658500
H	0.50484100	-0.56265800	3.77442300
H	1.05944400	-0.40931200	5.46982500
H	4.27536000	2.39998300	0.62026600
H	-0.86383900	-1.59978200	-4.36982000
H	-13.24933300	6.36227500	-2.20521900
H	-12.81880400	6.29833800	-3.94039700
H	-5.01133400	5.07162100	4.72499500
H	-4.46749800	5.95763200	6.21334200
H	-4.21394000	2.22004700	3.60478100
H	-3.69972300	3.82255400	4.29266500
H	-0.77835800	7.62795000	5.56854500
H	-0.55695900	9.33141800	6.15068200
H	-4.36625400	5.21083100	-4.54096600
H	-2.77484000	5.64155500	-5.28844000
H	2.70556500	9.89035400	-4.17556200
H	2.77788800	9.05017900	-5.73902100
H	8.81544000	6.84966200	2.46883800
H	9.46958800	5.35506100	3.20010700
H	6.15577700	6.24243300	-3.85873600
H	2.64599300	3.25505900	-4.58634900
H	10.00364300	3.50257500	-2.07407900
H	5.63116400	2.51787800	-7.56760500
H	6.44915700	1.08372100	-8.31321900
H	0.06901400	-1.07183600	-9.07353600
H	0.08999000	-2.77042600	-8.57329800
H	-4.85684600	0.88315500	-7.80206400
H	-4.65075400	-0.79251800	-8.39118400
H	-3.71830500	-7.43242200	-5.87306300
H	-2.35357600	-7.81890200	-6.94449000
H	2.78194400	-6.58927300	-5.99203600
H	8.55674200	-6.37603400	-4.77410300
H	8.63313100	-6.13979800	-3.01776500
H	-1.69314600	-9.01409900	-0.45491300
H	-1.57431700	-8.72008200	1.32273200
H	2.23775400	-8.33524500	6.04050000
H	3.82725600	-8.88159600	6.69409200
H	9.35651100	-5.88597200	1.68301600
H	8.99263000	-6.25210100	3.37101200
H	11.08225900	-0.44772300	4.53166000
H	11.25435000	1.19821000	3.81011600
H	-1.82412200	1.58613500	-0.65673000
H	-7.06320400	1.94041500	0.72117500
H	-8.76813500	0.45456400	-0.80379000
H	-9.44617000	-1.07530500	0.79317500
H	-9.62531100	-4.87019500	3.84080100
H	-9.80827200	-5.31424000	2.13466500
H	-8.18295600	-5.14334700	2.82988700
H	-10.19154500	-2.84089500	2.58491600
H	-7.40311600	-2.76005200	3.20755100
H	-7.51612400	2.61980900	-1.48089800
H	-8.98140300	-3.23679000	8.47576000
H	-8.15939700	-4.67918700	7.93858500
H	-2.53597700	-5.76882800	4.78078900
H	-3.38721300	-5.02093700	6.12225600
H	-5.81352900	-3.71852300	5.35618600
H	3.37547600	2.13715600	3.07105100
H	-4.99807300	-3.37974200	1.26195300
H	-8.50790900	-2.86014600	4.96651900
H	0.06602200	-2.55122400	6.56698300
H	1.46817000	-2.50698100	8.94775800
H	4.13616300	-3.19511200	7.59266000
H	3.93238100	-2.14757100	9.00924500
H	3.57020200	-3.88477300	9.12605200
H	-10.02616400	-5.26379100	9.54245400
H	-9.45691700	2.20230200	3.40349400
H	-8.55913700	3.36675900	2.44534700
H	-7.70453800	2.05419400	3.32316600
H	-11.19854800	-4.48871400	8.45392700
H	-10.36053000	-5.94728000	7.93775000
H	-9.41693700	-3.68429900	0.38074900
H	-7.73285700	-3.51089800	0.88394400
H	-5.73085600	0.41291200	-1.06362600
H	-6.92831000	0.42281100	-2.35398700
H	2.13707600	-4.20323600	6.49645700
H	-2.09946000	-2.89837300	5.75335400
H	1.36781900	-4.77349500	7.97266900
H	-1.34470800	-3.59921200	4.32817500
H	2.56765300	-1.61797700	6.48369500
H	2.14157100	3.87324500	1.72742600

E:HPA-5 (+8.9)

C	-8.66745500	1.96379200	2.99521900
C	-8.83162100	0.93715000	1.90321200
O	-9.80128100	0.17573200	1.85697700
N	-7.77852800	0.85228400	1.04980200
C	-7.86155900	0.01284600	-0.13784300
C	-7.54679300	-1.44179000	0.25936300
O	-6.42312500	-1.94057100	0.08805200
C	-6.85333800	0.56859200	-1.16406300
O	-6.82416100	1.98188600	-1.13012600
N	-8.56313000	-2.12249500	0.81466400
C	-8.43293000	-3.51140400	1.22294900
C	-8.74876300	-3.79772400	2.69803100
C	-8.66211700	-5.30346600	2.95755000
O	-7.89564500	-3.06983400	3.57901200
C	-9.79093500	-5.30692000	8.94840500
C	-8.86735800	-4.27643700	8.29568500
C	-8.94265500	-4.25372100	6.77392900
O	-9.70734700	-4.94936800	6.12680700

O	-8.07724600	-3.38375600	6.25635100
C	-1.57653200	-3.69656500	5.59097800
C	-0.29753600	-4.25764600	6.19899400
O	0.00645000	-5.44489000	6.08345300
C	-1.70570400	-4.11593400	4.11366600
C	-3.02296500	-3.71962300	3.53004100
C	-4.29468200	-4.21640800	3.68923200
N	-3.19124900	-2.60615900	2.71870100
C	-4.51792400	-2.47879700	2.42521200
N	-5.21109200	-3.44317200	3.00267100
N	0.46968400	-3.37010100	6.87957100
C	1.80459000	-3.71787500	7.32214400
C	2.42951300	-2.51067900	8.04738400
C	3.94170500	-2.68454300	8.17253100
O	2.07351100	-1.27913200	7.44017400
N	-4.37339600	3.63153200	-1.49363000
N	-2.75512400	2.55712600	-0.56325900
N	-3.93466400	0.08081200	-5.28427600
N	-4.23778900	-2.17222500	-1.55372600
N	-0.71174000	-0.58335700	-4.66914200
N	0.09113800	-1.53176000	-7.01310800
N	6.99336400	3.55966100	5.96754500
N	5.96266800	1.55808000	-1.74862000
N	4.82238900	2.94952400	-0.45848500
N	6.83465400	4.66776200	-3.67098600
N	3.48122700	4.26027400	-3.36908800
N	-0.04412800	-5.70206500	-4.47214900
N	-1.16592200	-4.40684400	-1.38346200
N	-1.81893300	-7.14496100	0.18009000
N	-5.14079000	-4.71536300	-7.29554400
N	-5.42434300	-3.77044700	-5.29967300
C	-3.74377400	4.74364800	-4.53070200
C	-3.10794900	5.10941600	-3.18008300
C	-3.20517400	4.07445200	-2.10829500
C	-2.18229600	3.38183800	-1.51552500
C	-4.08488000	2.72834000	-0.55827300
C	-5.06580900	-0.55740500	-7.32799900
C	-3.84313100	-0.66021700	-6.43077400
C	-3.02314300	-0.07414500	-4.15008200
C	-1.59429200	0.40782300	-4.49531400
C	-3.05981300	-1.49988200	-3.55527000
C	-4.35986300	-1.76184000	-2.82902000
C	0.70685000	-0.45736600	-4.90221000
C	1.07465500	-0.86882900	-6.34351900
C	1.49298300	-1.36821000	-3.91968600
C	3.89955900	-0.39829900	4.34099600
C	0.24254300	-1.97607900	-8.38392800
C	1.70551800	8.97176600	-4.99668900
C	2.16791400	7.65561900	-4.34930900
C	1.48443900	7.26565400	-3.03513000
C	0.00074300	6.95794600	-3.17020000
C	-1.03110100	8.71924200	5.13358900
C	0.05464800	9.01782900	4.07399400
C	0.19698400	8.00957400	2.95814700
C	-0.82523400	7.78855000	2.02419700
C	1.38140200	7.27796800	2.80940300
C	-0.68631200	6.85359400	1.00283600
C	1.54631500	6.34979800	1.78363500
C	0.49917100	6.11102900	0.88600000
C	-5.27830600	5.81743300	5.39814800
C	-5.10052500	6.98414000	4.39446600
C	-5.54124900	6.56466700	3.00366600
C	-6.87760300	6.25269600	2.71337600
C	-4.60371900	6.37352400	1.98176500
C	-7.26789800	5.75468800	1.47077400
C	-4.97450700	5.89256100	0.72669700
C	-6.31209600	5.56052000	0.45672200
C	-3.53020900	2.89330300	3.66211800
C	-2.18118400	2.25986400	4.06175200
C	-1.08844700	3.29861400	4.36166200
C	-1.73059900	1.23036800	3.01657500
C	-0.71088900	4.19336700	3.18484700
C	3.77480300	-8.30548800	5.83735300
C	4.42544400	-7.04308900	5.24387600
C	3.83470600	-6.57350000	3.93577500
C	4.46195800	-6.86210000	2.71829100
C	2.65179700	-5.82371300	3.92013400
C	3.94312900	-6.38041800	1.51413100
C	2.13976700	-5.33116700	2.72175200
C	2.78971600	-5.59517200	1.51486600
C	11.18126200	0.64473200	3.18506500
C	9.99481900	0.27904800	2.27920400
C	8.59324100	0.64984500	2.80004500
C	8.40390100	2.16456600	2.94458900
C	7.51069000	0.07107400	1.87809800
C	8.30633800	6.27621600	2.49239900
C	7.47299200	6.50726500	3.74797200
C	7.03147500	5.28622500	4.50729500
C	7.77097500	4.56245600	5.40980000
C	5.71418100	4.69083500	4.49443900
C	5.72319100	3.61618200	5.42767800
C	4.51820700	4.97548000	3.80938000
C	4.58309800	2.85069400	5.69966800
C	3.38941700	4.20819200	4.06471200
C	3.42188200	3.15452700	5.00065200
C	8.24055300	-5.49546100	-4.14238300
C	6.70492300	-5.63204300	-4.07599000
C	5.95252600	-4.74567800	-3.10744600
C	5.03192900	-3.81035900	-3.59577400
C	6.06699300	-4.86672700	-1.71356500
C	4.23298000	-3.05721900	-2.74454200
C	5.26526300	-4.12409300	-0.84585400
C	4.30546100	-3.23624800	-1.35844000
C	9.26424100	-5.03391600	2.39790300
C	7.84630400	-4.46136000	2.23901700
C	7.12229600	-4.10940500	3.54903100
C	7.92940400	-3.13190400	4.41290400
C	5.71841600	-3.56522600	3.25447300
C	8.68251700	3.46103500	-2.67662700
C	7.75893300	4.67651700	-2.67265400
C	8.31692100	2.49963600	-1.50277400
C	6.84759700	2.46460200	-1.19047400
C	6.14298900	3.33044000	-0.38108100
C	4.76153600	1.87136000	-1.27656600
C	5.62152400	5.46485200	-3.61247500
C	4.49830900	4.61043900	-4.20812300
C	2.64418700	3.11920300	-3.73279500
C	1.48234300	2.88120800	-2.77457200
C	5.91443400	1.77806200	-7.68032600
C	5.50462900	0.85584400	-6.52088600

C	6.01516700	1.28649200	-5.16548600
C	1.85943100	-6.90729800	-5.43403100
C	1.19437100	-5.59294000	-5.04376300
C	2.22036000	-7.79502900	-4.22578800
C	3.16159900	-7.13838300	-3.19354500
C	2.44591700	-6.17916300	-2.23240300
C	3.90973200	-8.21066700	-2.39187300
C	-0.64391000	-4.59167200	-3.75621600
C	-1.35094400	-5.11779600	-2.51368000
C	-1.91835700	-4.71278900	-0.16618100
C	-1.39483800	-5.93119000	0.63428800
C	-1.11205500	-8.38052000	0.52606900
C	-3.31210600	-7.60362900	-6.62092300
C	-3.06925800	-6.09907700	-6.87945400
C	-4.15392900	-5.16508100	-6.43694600
C	-4.31668400	-4.58794900	-5.19481600
C	-5.87158100	-3.87805400	-6.58228200
C	2.17099700	2.34989500	0.98703000
C	3.11166000	1.78655300	1.87926900
C	2.99236800	0.47220400	2.28777700
C	1.10259000	1.52156500	0.57447100
C	1.02091900	0.19488400	1.00013600
C	1.96192000	-0.36372600	1.86994000
C	-13.25058400	5.08213800	-2.65665100
C	-11.74950300	4.78731500	-2.42587900
C	-11.36186900	3.92891200	-1.21029100
C	-9.82879400	3.76641600	-1.12009000
C	5.23433100	-0.50426100	5.03268100
O	-2.86281200	-1.34368500	-6.71530400
O	-1.35113300	1.61574000	-4.58222900
O	-5.46393500	-1.60720600	-3.37819800
O	2.20314900	-0.66831700	-6.78638100
O	-0.67840600	7.25288500	-4.13571700
O	-0.57061900	6.37455200	-2.10936300
O	0.60208300	5.18027600	-0.09744900
O	-6.64171500	5.06825800	-0.76392200
O	3.47449100	-2.59686800	-0.50138400
O	7.80797300	5.54322700	-1.79808400
O	4.55305200	4.24659400	-5.37864000
O	0.29868400	3.58399000	-3.15288000
O	6.92018800	2.10202200	-5.00784800
O	5.43011200	0.63336000	-4.17282900
O	1.75443300	-4.51395100	-5.19865000
O	-2.07101400	-6.12797700	-2.57626500
O	-0.67007600	-5.78710700	1.61193000
O	2.38238400	3.57495500	0.51689800
O	4.07200500	0.01273400	3.08011500
O	-9.34878400	2.61053000	-1.27264900
O	-9.17166600	4.83603700	-0.92549900
O	2.81655400	-0.63293500	4.85091900
S	0.81437900	-1.47837700	-2.19526100
H	-3.52135600	6.05586700	-2.81233000
H	-2.04709700	5.29613900	-3.31582200
H	-1.12360300	3.41442700	-1.73830900
H	-4.81003000	2.24267200	0.06893500
H	-3.52602900	3.70120300	-4.78680300
H	-5.94958800	-0.87090600	-6.76174300
H	-2.98032700	-2.22144000	-4.37488400
H	-2.20533100	-1.63637900	-2.88268400
H	-5.07709600	-2.22339600	-0.96993000

H	-3.32528500	-2.24147600	-1.13235000
H	-3.36560500	0.64152600	-3.39808300
H	-4.86204100	0.38863300	-5.02985300
H	1.30550800	-1.97625500	-8.62864500
H	-0.85981700	-1.46345700	-6.66841500
H	2.00208500	6.84009700	-5.06129500
H	3.25065400	7.68830600	-4.17510400
H	1.58021200	8.05706600	-2.27812800
H	1.97414200	6.37914600	-2.61716800
H	0.61644800	8.99114300	-5.05769400
H	-0.13780000	10.01533800	3.65469900
H	1.02059900	9.09190700	4.58724700
H	2.19445000	7.42964400	3.51574700
H	-1.75052500	8.35581900	2.09657900
H	2.45843200	5.77326800	1.68208700
H	-1.48307400	6.68535900	0.28688200
H	0.05590000	5.91483100	-1.49189200
H	-2.03720700	8.92092200	4.75243600
H	-5.64933600	7.87231900	4.73322300
H	-4.04355200	7.26928300	4.35222900
H	-7.63636400	6.39374000	3.48056300
H	-3.55670300	6.60125100	2.16672300
H	-8.30763100	5.53181000	1.25416100
H	-4.22685100	5.78673900	-0.05099300
H	-7.66145200	4.90771500	-0.84935100
H	-6.29884900	5.78461500	5.79372400
H	-2.35754100	1.71100700	4.99851300
H	-1.49838100	1.71538100	2.06251900
H	-0.81687200	0.71021300	3.31953800
H	-2.51661800	0.48260900	2.84278900
H	-1.43547100	3.92544400	5.19471700
H	-0.19331400	2.77544400	4.72403200
H	-1.58302600	4.70037500	2.76260700
H	-0.01474400	4.97255100	3.49827400
H	-0.22651300	3.63313800	2.38004800
H	-3.51250800	3.30257500	2.64630100
H	4.35622000	-6.23348500	5.98162400
H	5.49601600	-7.23604600	5.10133700
H	5.37950000	-7.44692900	2.71651600
H	2.11724000	-5.63721400	4.84719600
H	4.45289200	-6.58980500	0.57903200
H	1.21963400	-4.76098000	2.71831900
H	2.40799300	-5.17724700	0.59060400
H	3.80527000	-9.12752500	5.11400600
H	10.13108700	0.75695000	1.29876200
H	10.01860700	-0.80254400	2.09066900
H	8.47712700	0.19534500	3.79792000
H	7.40147200	2.40434500	3.31072100
H	8.51963900	2.66025000	1.97344100
H	9.12192000	2.61283100	3.63781000
H	6.50583300	0.27021000	2.26050400
H	7.56730100	0.51732000	0.87930800
H	7.61710400	-1.01318300	1.76252300
H	12.11970500	0.36993600	2.68779500
H	6.57683400	7.07980800	3.47847800
H	8.03923000	7.15961900	4.42660300
H	8.80095000	4.68749100	5.71249400
H	7.31960200	2.86194900	6.61410800
H	4.47885600	5.77929600	3.07981500
H	2.47554900	4.39984400	3.51624700

H	4.60074400	2.04917400	6.43150000
H	2.53048100	2.55755700	5.16290700
H	7.78629700	5.63996800	1.77016900
H	6.30059200	-5.44638700	-5.07807200
H	6.47478400	-6.68518900	-3.85675300
H	6.78235600	-5.57099200	-1.29165900
H	4.90999800	-3.68152800	-4.66779900
H	5.34332400	-4.24324300	0.22826700
H	3.54319300	-2.33100100	-3.14180000
H	2.73449700	-2.16995000	-1.00580100
H	8.55666100	-4.46548200	-4.32291700
H	7.88278600	-3.57106800	1.59669000
H	7.22612700	-5.18929100	1.70149800
H	7.00665300	-5.04462400	4.11881500
H	8.09683600	-2.18904500	3.87938400
H	7.39833800	-2.89959600	5.34283500
H	8.90904100	-3.53732900	4.68554900
H	5.76593900	-2.61338800	2.71340200
H	5.15289700	-3.40569200	4.17887200
H	5.13954100	-4.26058800	2.64528800
H	9.99449300	-4.27020100	2.68078600
H	8.66117500	1.49066800	-1.74664600
H	8.83648600	2.80978200	-0.59119800
H	6.46611700	4.18808600	0.18511200
H	3.84574000	1.33528300	-1.47796500
H	8.59247200	2.92065000	-3.61926700
H	5.44530600	5.75241000	-2.57204100
H	6.82308800	3.87073500	-4.30659200
H	1.28437500	1.80306300	-2.71475300
H	1.74870100	3.21158900	-1.76611300
H	-0.17662900	3.02536300	-3.79594000
H	3.27831400	2.22178800	-3.76107000
H	3.66378200	4.36028300	-2.37815300
H	4.42265400	0.70088400	-6.46456900
H	5.92068600	-0.15106000	-6.66662300
H	6.87572100	2.24981800	-7.46484300
H	1.30635000	-8.13628500	-3.71866000
H	2.69872300	-8.69999400	-4.62233800
H	3.90637600	-6.55018300	-3.74320300
H	1.61809900	-6.68288800	-1.71633500
H	2.06355000	-5.29206000	-2.73515300
H	3.15067900	-5.82254500	-1.47970700
H	4.47277000	-8.88616800	-3.04678100
H	4.61505000	-7.74474800	-1.69669500
H	3.21268400	-8.81889100	-1.80140200
H	1.20984500	-7.46249700	-6.12286200
H	-1.40010300	-4.08703500	-4.37474100
H	0.12230800	-3.85621400	-3.50023000
H	-0.45172000	-6.61021000	-4.29768000
H	-2.96838700	-4.86816600	-0.43604600
H	-1.84446000	-3.85507200	0.50217700
H	-0.51218700	-3.61337000	-1.42083200
H	-0.03056000	-8.21637200	0.42074300
H	-2.20996700	-7.11085200	-0.75908400
H	-2.89222300	-5.93254000	-7.94732800
H	-2.14421300	-5.81820900	-6.36250200
H	-3.78568400	-4.72652300	-4.26525000
H	-5.71512100	-3.08494700	-4.60318800
H	-6.73017000	-3.32759900	-6.94077000
H	-4.02750500	-8.04315100	-7.32039500

H	-11.20698000	5.73565000	-2.34607200
H	-11.35996700	4.29307100	-3.32605100
H	-11.70920600	4.41608000	-0.29016700
H	-11.81987100	2.93689900	-1.26733000
H	-13.84302700	4.16537900	-2.74486700
H	0.97703100	0.59052400	-4.76827100
H	1.55340800	-2.36718900	-4.35874300
H	2.50848400	-0.96890300	-3.89900500
H	1.34776000	4.46468200	0.12813800
H	3.95560700	2.37365100	2.21331600
H	0.39692200	1.90346900	-0.15658800
H	-2.45349000	-1.97936000	2.43088500
H	-5.32202300	4.03695200	-1.55375100
H	5.80998100	0.91907100	-3.27257800
H	5.88879800	-1.17411400	4.47727700
H	5.70140000	0.48299600	5.03333400
H	5.10389800	-0.86061500	6.05374200
H	3.99002300	3.36759300	0.00805800
H	-1.00804400	-1.51520300	-4.43093000
H	-13.68024400	5.70188700	-1.86310500
H	-13.29380700	5.62428000	-3.60661100
H	-5.14008400	4.91463000	4.79360700
H	-4.58326700	5.85406100	6.24391500
H	-4.25924700	2.07707400	3.70091700
H	-3.79144400	3.71403800	4.33854300
H	-0.99533000	7.66696000	5.43415900
H	-0.82794000	9.39008800	5.97492400
H	-4.82828000	4.88753000	-4.49058100
H	-3.28448800	5.37025200	-5.30315200
H	2.04163600	9.87211500	-4.47128400
H	2.09533400	9.00329200	-6.01968600
H	8.50819000	7.23555300	2.00627100
H	9.25220100	5.78584700	2.74194500
H	5.65635700	6.38281300	-4.20846000
H	2.25641400	3.23341800	-4.75106600
H	9.71459700	3.80495600	-2.55729000
H	5.16411100	2.56236800	-7.82302800
H	6.01700500	1.14930200	-8.56979800
H	-0.28603000	-1.29299700	-9.05754300
H	-0.17343300	-2.97827000	-8.52554800
H	-5.24448400	0.47544600	-7.64733700
H	-4.98650000	-1.20182100	-8.21092500
H	-3.67767700	-7.74156200	-5.59983800
H	-2.33600900	-8.09157000	-6.71210400
H	2.77141000	-6.62352900	-5.96966300
H	8.57077800	-6.13767200	-4.96515100
H	8.69857700	-5.86167600	-3.21760600
H	-1.39612300	-9.12131800	-0.22865000
H	-1.22765500	-8.78544200	1.53748300
H	2.72779300	-8.13897000	6.10620700
H	4.36152200	-8.60294100	6.71270700
H	9.57613100	-5.47854800	1.44836600
H	9.28812300	-5.82407000	3.15595700
H	11.16183800	0.08628000	4.12646100
H	11.23640600	1.72300400	3.36784600
H	-2.24524300	1.94399000	0.05791100
H	-7.25213400	1.70556400	0.88392100
H	-8.87897300	0.07515900	-0.53940800
H	-9.36462800	-1.56070600	1.10371500
H	-8.93800100	-5.51566600	3.99184600

H	-9.33713900	-5.85866600	2.29664400
H	-7.64063700	-5.65582700	2.77432000
H	-9.76429000	-3.44907200	2.92446500
H	-6.94608700	-3.25854000	3.34017200
H	-7.76773900	2.32376000	-1.25714300
H	-9.09452600	-3.26203400	8.64609300
H	-7.81959500	-4.44870300	8.56878200
H	-0.89244000	-3.67786300	3.52648700
H	-1.56448200	-5.19589900	4.03883900
H	-4.60181100	-5.08161100	4.25830700
H	1.93469300	-1.40692200	2.15660000
H	-4.92860500	-1.71905700	1.77719300
H	-8.12025000	-3.34998400	5.25429600
H	0.31061800	-2.37338700	6.79119200
H	1.99080100	-2.45496600	9.05198100
H	4.40168300	-2.75460800	7.18039900
H	4.37940000	-1.83261700	8.70012500
H	4.19160100	-3.60059600	8.71940800
H	-9.70325000	-5.26706800	10.03833100
H	-9.50054100	1.82812400	3.68842700
H	-8.68829400	3.01203800	2.67687600
H	-7.74696800	1.75598700	3.54859100
H	-10.83266700	-5.12278700	8.67352600
H	-9.54225800	-6.31827100	8.61586900
H	-9.10108300	-4.12907900	0.60836900
H	-7.40628100	-3.81140700	1.00378200
H	-5.85276800	0.20815300	-0.90573600
H	-7.08981400	0.16975300	-2.15707900
H	2.42390500	-4.00438700	6.45984200
H	-2.42239300	-4.10911800	6.15198600
H	1.77355200	-4.57864700	7.99896500
H	-1.62639700	-2.60680300	5.69119600
H	2.46932200	-1.19344600	6.54647300
H	0.23470900	-0.43463200	0.59570100

E:HPA-6 (+11.5)

C	-8.74435100	1.87502700	2.90058700
C	-8.84066300	0.82235700	1.82605500
O	-9.78465000	0.02778400	1.76821600
N	-7.75952000	0.75504900	1.00956400
C	-7.76732900	-0.10100000	-0.16985900
C	-7.41558000	-1.54088900	0.25140800
O	-6.30906200	-2.04243600	-0.00103600
C	-6.74308000	0.47697600	-1.17135800
O	-6.75032400	1.89189000	-1.14903000
N	-8.36837100	-2.20562800	0.92584000
C	-8.16713800	-3.56874300	1.38900000
C	-8.48673900	-3.81467400	2.87026100
C	-8.30034700	-5.29920100	3.19233700
O	-7.70410200	-2.99792600	3.74067400
C	-9.73357900	-5.16482100	9.14788100
C	-8.76865800	-4.17584300	8.48556700
C	-8.80744500	-4.18342200	6.96063900
O	-9.56087200	-4.88430200	6.30710600
O	-7.91737300	-3.33238100	6.44841500
C	-1.50558400	-3.45249600	5.70451800
C	-0.22338400	-3.97145900	6.33414400
O	0.14077900	-5.13845300	6.19249300
C	-1.45811600	-3.56515800	4.16736400
C	-2.78688900	-3.23545000	3.56267500
C	-4.05553800	-3.69822200	3.82176500
N	-2.96564900	-2.27792700	2.57621000
C	-4.29902700	-2.19763800	2.28856200
N	-4.98378600	-3.05147900	3.02882600
N	0.48546900	-3.07039300	7.06449900
C	1.83316200	-3.38892800	7.49603300
C	2.49348400	-2.13334000	8.09483700
C	4.00959300	-2.29961700	8.16578500
O	2.12342700	-0.95721400	7.38701300
N	-4.24727900	3.38471200	-1.53274600
N	-2.39174500	2.80687500	-0.57151000
N	-3.85667000	-0.20696400	-5.32577400
N	-4.17744000	-2.50075800	-1.63052700
N	-0.55751800	-0.81706400	-4.56611300
N	0.17055200	-1.83427900	-6.92649400
N	6.79962200	3.90925700	5.81009700
N	6.02949600	1.35985400	-1.73993600
N	4.86571500	2.29695000	-0.12071500
N	6.72808600	4.66753200	-3.81503200
N	3.39209300	4.16037100	-3.51610600
N	0.05390000	-5.94464000	-4.21310200
N	-1.07158100	-4.49393900	-1.21316000
N	-1.67377700	-7.18159500	0.49442200
N	-5.03965400	-5.17848600	-7.10961600
N	-5.36738100	-4.12234400	-5.17772500
C	-3.85034800	4.44786300	-4.71753100
C	-3.33228000	4.98814600	-3.36607200
C	-3.22595000	4.01806600	-2.23688000
C	-2.06864400	3.62857800	-1.62240300
C	-3.72112500	2.67235900	-0.52250200
C	-5.04325800	-0.99315800	-7.29610700
C	-3.78876300	-1.01359000	-6.42911400
C	-2.91356700	-0.31096600	-4.21087800
C	-1.49260300	0.14525500	-4.63049900
C	-2.95033200	-1.70710000	-3.55556400
C	-4.27142400	-1.99104100	-2.87367500
C	0.84564200	-0.67777600	-4.88858300
C	1.13820200	-1.08104300	-6.34387000
C	1.67502500	-1.62432100	-3.98425800
C	3.78655100	0.18102200	4.09939200
C	0.29945700	-2.33657800	-8.27368500
C	1.50714300	8.77244100	-5.34095800
C	2.01579800	7.50041500	-4.64177800
C	1.40759000	7.17754300	-3.27333900
C	-0.07413100	6.83885000	-3.31380800
C	-1.26775200	8.88136700	4.78207400
C	-0.19390400	9.14351400	3.69754200
C	0.02582100	8.04680100	2.67526500
C	-0.99763000	7.59472000	1.82971300
C	1.29524500	7.47633300	2.52030200
C	-0.77343000	6.59263200	0.88695800
C	1.53700800	6.48898900	1.56534800
C	0.50005100	6.02248000	0.75831400
C	-5.45126200	5.89952700	5.15242200
C	-5.29365700	7.03426900	4.11039000
C	-5.70685600	6.55797500	2.73088400
C	-7.03852000	6.25082500	2.41724400
C	-4.74375200	6.30142300	1.74782500
C	-7.39957600	5.69916800	1.18835800

C	-5.08341900	5.76844700	0.50676200
C	-6.41814600	5.44379100	0.21122600
C	-3.63189900	2.94503800	3.54587700
C	-2.30877700	2.35330100	4.08633300
C	-1.26795100	3.42613100	4.45359100
C	-1.76107500	1.29949900	3.12233800
C	-0.92156300	4.38597400	3.32029200
C	3.90803800	-7.98876300	6.21105900
C	4.54033800	-6.72714400	5.58935600
C	4.00703900	-6.32904200	4.23300800
C	4.72279900	-6.62941800	3.06746000
C	2.79508700	-5.63600400	4.11701000
C	4.25402100	-6.22569700	1.81519600
C	2.33054100	-5.22186600	2.86945100
C	3.06098900	-5.50901300	1.71437300
C	11.12707100	1.00441900	3.21307200
C	9.97815800	0.56371900	2.29437400
C	8.54875900	0.94837000	2.72116600
C	8.34874800	2.46767100	2.77338500
C	7.53548400	0.30051300	1.76576000
C	8.13228700	6.53763800	2.27643300
C	7.28910400	6.80190300	3.51894700
C	6.84605900	5.59881900	4.30685800
C	7.58097600	4.89662900	5.23002400
C	5.52708500	5.00645100	4.30755100
C	5.52817500	3.96203400	5.27461600
C	4.33360000	5.27683700	3.61387400
C	4.38144900	3.21895200	5.57677500
C	3.19864600	4.52610000	3.89464500
C	3.22286700	3.50534800	4.86680300
C	8.35460100	-5.49827200	-3.86169300
C	6.82173100	-5.59139000	-3.76317000
C	6.14835800	-4.57147200	-2.87037700
C	5.45730900	-3.49628900	-3.44171000
C	6.14168200	-4.67369600	-1.47141900
C	4.74177500	-2.59365200	-2.66531000
C	5.40684900	-3.78875200	-0.67905100
C	4.65482600	-2.76817800	-1.27921100
C	9.34085100	-4.74122800	2.65856700
C	7.91684900	-4.19289200	2.47153000
C	7.20734600	-3.70784500	3.74853000
C	8.01719900	-2.64246900	4.49813500
C	5.80163000	-3.19460800	3.40792000
C	8.55089200	3.53393400	-2.74711500
C	7.57116700	4.68881500	-2.75160900
C	8.24349200	2.59961700	-1.55737700
C	6.80028000	2.36913100	-1.18136000
C	6.07684900	2.96381900	-0.17131100
C	4.88304200	1.33398800	-1.07765300
C	5.49272100	5.41391500	-3.79811800
C	4.40271400	4.50080200	-4.36396400
C	2.56805900	2.99952700	-3.83166100
C	1.35021300	2.84033400	-2.92444600
C	5.88427900	1.56829200	-7.70764400
C	5.48483600	0.68543800	-6.51723000
C	5.99496300	1.16352600	-5.18012200
C	2.01162600	-7.10381400	-5.11525400
C	1.21881200	-5.81584400	-4.91807100
C	2.41181400	-7.79482200	-3.79609100
C	3.34788900	-6.97121800	-2.88898900
C	2.66235300	-5.78830600	-2.18981800
C	4.01098100	-7.87973400	-1.84660300
C	-0.57716200	-4.79644500	-3.58604900
C	-1.26509100	-5.26079800	-2.30821100
C	-1.79595200	-4.76645900	0.03083300
C	-1.23878800	-5.94426800	0.86825600
C	-0.95268100	-8.39327700	0.89075000
C	-3.13808400	-7.96326500	-6.28978000
C	-2.92608100	-6.46400300	-6.60017600
C	-4.04417900	-5.54180400	-6.22007400
C	-4.23448500	-4.89518800	-5.01687900
C	-5.80177300	-4.32274400	-6.45384000
C	0.68201400	1.66414200	0.88137500
C	1.83304800	1.76361400	1.70683400
C	2.46755100	0.60084500	2.14396400
C	0.25032000	0.36196400	0.52825300
C	0.91092500	-0.77006500	0.98645000
C	2.03649600	-0.67505600	1.80777900
C	-13.37038600	4.65314700	-2.89211000
C	-11.85384600	4.43508700	-2.64717700
C	-11.41301000	3.61174800	-1.42492900
C	-9.86854600	3.54594800	-1.31643100
C	5.20913200	0.25132300	4.58833700
O	-2.79795100	-1.68538300	-6.70623300
O	-1.29180800	1.31024900	-4.98337000
O	-5.36356400	-1.79712500	-3.43239300
O	2.22207700	-0.81386200	-6.86254700
O	-0.81385500	7.10664900	-4.23977700
O	-0.57479900	6.26685300	-2.20821600
O	0.75979900	5.05979200	-0.19135500
O	-6.71112800	4.90709400	-0.99381400
O	3.85594400	-1.98083300	-0.51237600
O	7.50473000	5.51309800	-1.83775400
O	4.48112500	4.08258200	-5.51441700
O	0.21810200	3.51459800	-3.46580500
O	6.86644700	2.01717500	-5.03857700
O	5.45093800	0.49793500	-4.17041600
O	1.61948800	-4.73241300	-5.32642900
O	-1.97498500	-6.27821100	-2.30466900
O	-0.47596300	-5.75127200	1.80835400
O	-0.00972800	2.72350300	0.49090100
O	3.66615600	0.75341100	2.88524200
O	-9.32275700	2.40880400	-1.35685200
O	-9.27342600	4.66133800	-1.20911700
O	2.87306600	-0.35326300	4.70198600
S	1.14396500	-1.65422500	-2.21345500
H	-3.96275600	5.82968100	-3.05330300
H	-2.33255600	5.40037100	-3.48846300
H	-1.05246700	3.84058300	-1.89821100
H	-4.29620200	2.12090400	0.20071000
H	-3.57330700	3.40257500	-4.88040500
H	-5.90400000	-1.30494600	-6.69588900
H	-2.80103400	-2.45790900	-4.33817700
H	-2.13875100	-1.79671800	-2.82626800
H	-5.02273500	-2.51747900	-1.05520700
H	-3.27769900	-2.55433700	-1.17996300
H	-3.23535200	0.44015000	-3.48321200
H	-4.78551700	0.07491900	-5.04701000
H	1.35826100	-2.32974500	-8.53663700
H	-0.75826800	-1.82934200	-6.52166500

H	1.82799500	6.64277500	-5.29659800
H	3.10531200	7.55721900	-4.52366900
H	1.52512200	8.01585700	-2.57169700
H	1.93767000	6.32735800	-2.83078300
H	0.41801300	8.75776100	-5.39609400
H	-0.44078100	10.08202100	3.18206500
H	0.76337600	9.32450700	4.19982800
H	2.11138700	7.80778900	3.15758300
H	-1.98935100	8.03322200	1.90525300
H	2.52440400	6.05989300	1.43504900
H	-1.57188900	6.25206800	0.23700200
H	0.09416800	5.84133700	-1.61857600
H	-2.27959800	9.04156700	4.39702000
H	-5.86766200	7.91940100	4.41402400
H	-4.24325900	7.34452100	4.07018900
H	-7.81667700	6.43930900	3.15460200
H	-3.69921600	6.51534100	1.95876300
H	-8.43565800	5.47859900	0.95215700
H	-4.31925900	5.61211300	-0.24537200
H	-7.72030100	4.75672800	-1.09686400
H	-6.47278100	5.86031000	5.54531600
H	-2.56350400	1.83943500	5.02551800
H	-1.50496500	1.75059500	2.16391900
H	-0.85310400	0.82078400	3.50312700
H	-2.51114900	0.51833600	2.94595700
H	-1.65279700	3.99903600	5.30942400
H	-0.35700100	2.92331700	4.80699500
H	-1.80494700	4.93867200	2.98929400
H	-0.18401000	5.12893100	3.63711000
H	-0.52129600	3.85985300	2.45088100
H	-3.55782300	3.29881200	2.51424200
H	4.40710700	-5.89143600	6.28827700
H	5.62274000	-6.88692400	5.50773300
H	5.66604000	-7.16610600	3.14503400
H	2.19859900	-5.43254300	5.00213300
H	4.82599300	-6.45099500	0.92068900
H	1.38563700	-4.70018000	2.78833400
H	2.70533900	-5.17229300	0.74691400
H	3.96606100	-8.83274500	5.51593000
H	10.14852600	0.97838300	1.29090800
H	10.02354000	-0.52717600	2.17751400
H	8.37695500	0.54930900	3.73340800
H	7.32273400	2.72646900	3.05186100
H	8.54343400	2.91633400	1.79250300
H	9.01192200	2.94952200	3.49767800
H	6.50707900	0.58859300	2.00036200
H	7.72229700	0.60875800	0.73295000
H	7.59592100	-0.79222800	1.79829300
H	12.08212800	0.73489300	2.74456400
H	6.39234600	7.36234000	3.22737900
H	7.84722000	7.47454700	4.18384900
H	8.61041000	5.02653600	5.53240700
H	7.11424300	3.24587900	6.49739200
H	4.29965700	6.07037100	2.87294200
H	2.27204900	4.73502600	3.37320300
H	4.39287500	2.44398700	6.33657000
H	2.32137900	2.93297300	5.06013900
H	7.62993500	5.86576600	1.57370000
H	6.39926100	-5.48926400	-4.76960800
H	6.55827000	-6.60380600	-3.42839400

H	6.68789000	-5.48337600	-0.98996600
H	5.45888200	-3.36989500	-4.52130500
H	5.35691200	-3.90985700	0.39703100
H	4.23418100	-1.76132000	-3.13040700
H	3.03345700	-1.76681000	-1.03851100
H	8.68066400	-4.47921400	-4.08789800
H	7.93954800	-3.37142800	1.74244100
H	7.29063400	-4.97157900	2.01913100
H	7.09478300	-4.57749900	4.41405100
H	8.19814700	-1.77117100	3.85900900
H	7.47820800	-2.29601900	5.38724200
H	8.99055700	-3.02071300	4.82675100
H	5.85336500	-2.34144400	2.72115100
H	5.26150300	-2.87760400	4.30711500
H	5.20070000	-3.96687700	2.92560200
H	10.05977300	-3.95601900	2.91056700
H	8.67906000	1.61820200	-1.77552800
H	8.75225700	2.95832400	-0.65799500
H	6.31888800	3.76911500	0.50235000
H	4.07352600	0.63385100	-1.22923500
H	8.48338200	2.97082700	-3.67785600
H	5.30211700	5.73953000	-2.77171600
H	6.77004200	3.87220500	-4.45108400
H	1.14269900	1.77256600	-2.78087500
H	1.53538800	3.26086400	-1.92918700
H	-0.25275300	2.88760600	-4.04431600
H	3.19690500	2.09861000	-3.78532600
H	3.53577300	4.34724300	-2.53220100
H	4.40338200	0.52591900	-6.45461200
H	5.90455900	-0.32441100	-6.62799100
H	6.83435300	2.06889800	-7.50757000
H	1.51781600	-8.08544700	-3.22602200
H	2.91513900	-8.73421300	-4.06001600
H	4.14155600	-6.55952400	-3.52577000
H	1.77646000	-6.12005800	-1.63339900
H	2.36752000	-5.00135900	-2.88309600
H	3.35356200	-5.32639600	-1.48302300
H	4.51880700	-8.73210100	-2.31363300
H	4.75117400	-7.31737000	-1.27048600
H	3.27109900	-8.27451800	-1.13915300
H	1.43461400	-7.79788300	-5.73967600
H	-1.34354400	-4.35519400	-4.23852700
H	0.17116000	-4.02601100	-3.37857000
H	-0.24751500	-6.84887500	-3.87896600
H	-2.84646700	-4.94967900	-0.21767100
H	-1.72853500	-3.88213800	0.66406600
H	-0.39594500	-3.72411000	-1.27825800
H	0.12638000	-8.21418500	0.78357700
H	-2.10079600	-7.20081800	-0.42867600
H	-2.72602700	-6.33327600	-7.66907500
H	-2.02185900	-6.14189700	-6.07106400
H	-3.70728700	-4.95661800	-4.07717000
H	-5.68065600	-3.40290600	-4.52861700
H	-6.67315900	-3.82095000	-6.85080800
H	-3.84106800	-8.44320900	-6.97502200
H	-11.36084800	5.41084900	-2.57910000
H	-11.43853000	3.95358800	-3.54288000
H	-11.79470000	4.08062500	-0.50820000
H	-11.80615100	2.59164900	-1.47209900
H	-13.92106000	3.70843000	-2.95023100

H	1.14623100	0.36248200	-4.75225000
H	1.63209300	-2.63085500	-4.40797700
H	2.70712800	-1.28861000	-4.07485600
H	0.48439300	4.11401900	0.12982200
H	2.18263200	2.73524900	2.03951000
H	-0.58118900	0.26389200	-0.16153300
H	-2.23576800	-1.68725700	2.19923800
H	-5.25988600	3.51370600	-1.62887400
H	5.84581100	0.79932000	-3.28484200
H	5.66755900	1.20397600	4.32666700
H	5.22753700	0.09529500	5.66749800
H	5.77918200	-0.54639800	4.10729800
H	4.12334400	2.41909100	0.55718100
H	-0.75947100	-1.64377500	-4.02551800
H	-13.83275200	5.28420600	-2.12657600
H	-13.42125300	5.15419800	-3.86396400
H	-5.29064100	4.97552400	4.58639700
H	-4.76086400	5.98654200	5.99818100
H	-4.34276000	2.11510100	3.61597600
H	-3.91376500	3.78726400	4.18646200
H	-1.21015000	7.84311900	5.12573800
H	-1.08318800	9.59064400	5.59460600
H	-4.93788600	4.56886000	-4.68746600
H	-3.40144700	5.05159200	-5.51420300
H	1.82035100	9.70109800	-4.85235100
H	1.89995400	8.76990600	-6.36310600
H	8.31537300	7.48031600	1.75140100
H	9.08779300	6.07947400	2.54978300
H	5.51003200	6.30658200	-4.43206400
H	2.18281300	3.06298200	-4.85396600
H	9.56783400	3.92381900	-2.63798800
H	5.11768500	2.32916700	-7.88581700
H	6.00470400	0.90546500	-8.57001300
H	-0.24088300	-1.69403100	-8.97706700
H	-0.09364400	-3.35283900	-8.37490500
H	-5.24320100	0.02127100	-7.65884300
H	-4.94591700	-1.67186400	-8.15110300
H	-3.50500200	-8.06670200	-5.26514300
H	-2.15112300	-8.43288000	-6.35725300
H	2.91948300	-6.82236800	-5.65910700
H	8.70231800	-6.16686400	-4.65583600
H	8.81641500	-5.81565200	-2.92096500
H	-1.21697300	-9.17084500	0.16671500
H	-1.06365000	-8.75810600	1.91792000
H	2.85672100	-7.83467200	6.46934400
H	4.49763800	-8.23672300	7.10009200
H	9.66423500	-5.21953400	1.72778600
H	9.37655600	-5.50050400	3.44655800
H	11.11631600	0.48642400	4.17744800
H	11.15833800	2.09102500	3.35109000
H	-1.58515800	2.51849400	0.05288900
H	-7.22838400	1.60970300	0.86587100
H	-8.77243200	-0.07986300	-0.60611300
H	-9.18181500	-1.65711700	1.21326700
H	-8.59401700	-5.49289200	4.22547300
H	-8.91298200	-5.92724200	2.53584800
H	-7.25085900	-5.58304300	3.05328000
H	-9.52854600	-3.53170700	3.06765300
H	-6.75143600	-3.03801900	3.45702100
H	-7.69807000	2.20470000	-1.30157000
H	-8.97299600	-3.14764900	8.80834900
H	-7.73196600	-4.37120300	8.78491000
H	-0.69278300	-2.88757100	3.77021400
H	-1.14665600	-4.57612900	3.88703200
H	-4.35647300	-4.45278700	4.53357900
H	2.58792600	-1.55327900	2.11613900
H	-4.71858600	-1.54983300	1.53385400
H	-7.93831500	-3.30459100	5.44740200
H	0.31302400	-2.07855900	6.94639800
H	2.09520200	-1.97899200	9.10567900
H	4.42437400	-2.41062400	7.15693400
H	4.46706300	-1.42311500	8.63287800
H	4.28742300	-3.18911400	8.74202800
H	-9.65947700	-5.10109100	10.23756300
H	-9.58598700	1.72962600	3.58017100
H	-8.78685800	2.90836200	2.53872700
H	-7.82194600	1.71080300	3.46527100
H	-10.76544300	-4.95610400	8.85374000
H	-9.51005700	-6.19032600	8.84197500
H	-8.79014200	-4.25049500	0.79435800
H	-7.12303700	-3.82049100	1.19222000
H	-5.74044400	0.14521700	-0.88607900
H	-6.94455400	0.06392200	-2.16687600
H	2.42126900	-3.73510600	6.63294900
H	-2.32275500	-4.07506900	6.08214700
H	1.81463800	-4.22003500	8.20877900
H	-1.71750900	-2.42064600	6.00314400
H	2.52016000	-0.94405700	6.49285500
H	0.58387200	-1.75101300	0.65523400

E:HPA-7 (+11.7)

C	-8.72277000	1.81931700	2.67409400
C	-8.84679600	0.78389800	1.58929900
O	-9.82524900	0.03574500	1.50595700
N	-7.75513800	0.67720300	0.79377400
C	-7.76843400	-0.14950300	-0.40647400
C	-7.48200400	-1.61318400	-0.01761300
O	-6.43292800	-2.19249400	-0.34205500
C	-6.70315500	0.43191000	-1.36098400
O	-6.70248100	1.84955900	-1.31799800
N	-8.42779900	-2.22168700	0.71606600
C	-8.24744300	-3.57963200	1.20368600
C	-8.63784900	-3.79305700	2.67211600
C	-8.36599400	-5.24962900	3.05766400
O	-7.96858500	-2.88621600	3.54691300
C	-9.99816900	-5.41351900	8.64302600
C	-8.93443800	-4.45918800	8.09780500
C	-9.06464400	-4.18566600	6.60455500
O	-9.97947300	-4.61329200	5.92000000
O	-8.06309800	-3.43378300	6.15312500
C	-1.75844600	-3.69647400	5.77826400
C	-0.45781100	-4.22777800	6.36228600
O	-0.11765700	-5.40224500	6.24419600
C	-1.72334000	-3.63983200	4.24021700
C	-3.05815900	-3.26251000	3.67369000
C	-4.34741600	-3.44032500	4.12353500
N	-3.19901000	-2.63907300	2.44668000
C	-4.52641800	-2.47524100	2.19943800
N	-5.25130700	-2.95145700	3.19802700

N	0.30058300	-3.32134400	7.04607600
C	1.63538200	-3.67480000	7.49613700
C	2.30529000	-2.46646900	8.14804300
C	3.76369900	-2.75052500	8.49157600
O	2.17755500	-1.31317300	7.30745100
N	-4.32530900	3.69298000	-1.67533500
N	-2.86640600	3.10154000	-0.21353000
N	-3.54320500	-0.01641400	-5.41897200
N	-4.04789800	-1.99183800	-1.65758700
N	-0.27432400	-0.63584000	-4.57020500
N	0.53770900	-1.62932900	-6.91800400
N	6.87873800	3.48387900	6.05287700
N	6.17801100	1.41330000	-1.45338900
N	4.88057500	2.69390700	-0.19816300
N	7.00502500	4.67879400	-3.36092700
N	3.65425900	4.22890400	-3.18150100
N	0.35509800	-5.76305700	-4.28063700
N	-0.96739300	-4.49674200	-1.23278100
N	-1.64951000	-7.22912600	0.24148700
N	-4.68121600	-4.94514900	-7.38230500
N	-4.99130100	-3.88669800	-5.44675200
C	-3.52055300	4.60243500	-4.66087100
C	-2.89104800	5.03737300	-3.33201400
C	-3.10242600	4.17420400	-2.13379500
C	-2.17428300	3.77540400	-1.20677700
C	-4.16860900	3.06330100	-0.51120300
C	-4.64752400	-0.74272700	-7.45943200
C	-3.42602400	-0.78732100	-6.54570400
C	-2.64412200	-0.14464600	-4.26953100
C	-1.20892000	0.32700000	-4.60146900
C	-2.71977000	-1.54669000	-3.62638600
C	-4.06958200	-1.76048200	-2.97458500
C	1.13582300	-0.46720800	-4.84992000
C	1.48544500	-0.88375300	-6.28978300
C	1.99337800	-1.34069100	-3.90074000
C	-0.95972700	-0.75699800	0.17180100
C	0.71970000	-2.09382100	-8.27384900
C	1.90576300	8.91629000	-4.91615800
C	2.40704800	7.62833000	-4.24184300
C	1.67627400	7.21051800	-2.96456400
C	0.21059700	6.86173300	-3.16880000
C	-1.32425100	8.67215900	5.04890800
C	-0.26367500	8.98717100	3.96690200
C	-0.01833700	7.93242000	2.91541800
C	-0.92858600	7.69706900	1.87741700
C	1.16437700	7.18308400	2.92515900
C	-0.67504800	6.74808800	0.89208300
C	1.44544800	6.23542500	1.94233200
C	0.51765300	6.00873000	0.91878100
C	-5.49276700	5.74228500	5.18216100
C	-5.28750800	6.90492500	4.18107100
C	-5.69053000	6.49070900	2.77727900
C	-7.00319200	6.10146500	2.46997000
C	-4.74129400	6.39757300	1.75316400
C	-7.35697500	5.62173500	1.21043800
C	-5.07804900	5.94044600	0.47929100
C	-6.39015300	5.52748000	0.19029600
C	-3.63184400	2.82742500	3.54972900
C	-2.32641500	2.15951700	4.02132800
C	-1.10206700	3.08211900	3.98829900

C	-2.10108500	0.85577900	3.24074300
C	-0.75942100	3.61985500	2.60498300
C	3.72886300	-8.24584400	6.14177300
C	4.37763700	-6.96365700	5.58283600
C	3.86030800	-6.51425100	4.23604400
C	4.63910600	-6.67464300	3.08373100
C	2.59753600	-5.91785200	4.11481600
C	4.18404600	-6.22568200	1.84173400
C	2.14183900	-5.46424000	2.87713700
C	2.93828000	-5.60673600	1.73847600
C	11.11567300	0.78430500	3.72030600
C	10.00143200	0.37382100	2.74420100
C	8.57254000	0.82291900	3.10237600
C	8.42028000	2.34576300	3.02832900
C	7.54701900	0.15219400	2.17781900
C	8.19378100	6.36253400	2.85227800
C	7.31593000	6.60728200	4.07446300
C	6.86821100	5.37640900	4.81452600
C	7.66043900	4.47420300	5.47909700
C	5.50785300	4.91932700	4.98340800
C	5.55214400	3.72521800	5.75754700
C	4.25412400	5.41186100	4.57769200
C	4.39709700	3.02792200	6.12717300
C	3.10373400	4.73043500	4.95267800
C	3.17524300	3.55014000	5.72154600
C	8.57767600	-5.45967700	-3.66639800
C	7.04179500	-5.56199600	-3.63598400
C	6.32698900	-4.58202200	-2.73336100
C	5.61439800	-3.51535000	-3.29288200
C	6.31306300	-4.70627600	-1.33594500
C	4.88323700	-2.63340500	-2.50809400
C	5.56565300	-3.84131000	-0.53442200
C	4.80495100	-2.81748600	-1.12156100
C	9.31533700	-4.92646100	2.90808400
C	7.91422300	-4.32457100	2.72803700
C	7.23095600	-3.86720400	4.03107500
C	8.08793600	-2.86964900	4.82257600
C	5.84511300	-3.27712300	3.73716200
C	8.81303700	3.49862800	-2.27445700
C	7.86182100	4.68805600	-2.30452300
C	8.43312200	2.54065400	-1.10932500
C	6.95920600	2.39879200	-0.87442900
C	6.15627800	3.19838900	-0.09125500
C	4.93883700	1.61753800	-1.01795100
C	5.78362300	5.46005400	-3.35811800
C	4.68864100	4.60002100	-3.99314300
C	2.84908000	3.07751500	-3.57767100
C	1.65232600	2.80306000	-2.66454200
C	6.30816500	1.74132100	-7.37286800
C	5.86455700	0.80722000	-6.23563100
C	6.32028800	1.22722400	-4.85868400
C	2.27536700	-6.97141700	-5.20922600
C	1.55559200	-5.65323300	-4.92449700
C	2.61578000	-7.76260700	-3.92914400
C	3.55203500	-7.03761100	-2.93803800
C	2.88104200	-5.89835500	-2.15614300
C	4.16631700	-8.04379600	-1.95694300
C	-0.22238200	-4.64422700	-3.55889400
C	-1.01906900	-5.19671600	-2.38211200
C	-1.83576100	-4.81766000	-0.09779100

C	-1.35212000	-6.00576900	0.75851000
C	-0.92515300	-8.43464600	0.63167900
C	-2.82916400	-7.75525400	-6.61197700
C	-2.57872100	-6.25235200	-6.86893600
C	-3.68752100	-5.31894900	-6.49581900
C	-3.86739600	-4.67156200	-5.29227900
C	-5.43222100	-4.08241400	-6.72073800
C	2.10237200	2.32096200	1.29511300
C	2.54357300	2.13478200	2.62240200
C	2.17077200	1.00657400	3.34341200
C	1.31882600	1.30186100	0.69891100
C	0.91146400	0.21814800	1.46798500
C	1.33607400	0.03866300	2.78355000
C	-13.10261800	4.82078800	-3.19749800
C	-11.61585100	4.54444500	-2.88781900
C	-11.29357100	3.67739200	-1.66002300
C	-9.76793900	3.57218800	-1.46430700
C	-1.16992600	0.39876800	-0.74930700
O	-2.41717700	-1.43652200	-6.80772900
O	-0.99192900	1.51952100	-4.83798900
O	-5.12622700	-1.69915300	-3.63436200
O	2.59156300	-0.62427800	-6.76070200
O	-0.44073400	7.13372000	-4.15836200
O	-0.38394300	6.26587600	-2.12529300
O	0.72998200	5.07760400	-0.04964800
O	-6.68282200	5.05190000	-1.04110100
O	4.01671700	-2.04312300	-0.34005700
O	7.83237300	5.53698800	-1.41153500
O	4.77418300	4.25368900	-5.16647000
O	0.45575600	3.48792500	-3.03215100
O	7.16531800	2.09456800	-4.65448200
O	5.76178700	0.50386900	-3.89910100
O	2.05049900	-4.56702000	-5.20382100
O	-1.69113400	-6.23327900	-2.51342000
O	-0.76590100	-5.84188800	1.82451900
O	2.45446900	3.40732300	0.61480200
O	0.06008400	-0.81670300	1.02679700
O	-9.22976100	2.43899600	-1.58350400
O	-9.17507800	4.67106000	-1.22859300
O	-1.71583500	-1.73253900	0.15581300
S	1.42860200	-1.38049200	-2.14501300
H	-3.21672600	6.05596700	-3.08717700
H	-1.81402800	5.11324000	-3.45611700
H	-1.10414200	3.90770700	-1.19294100
H	-4.96883400	2.61425400	0.04864400
H	-3.27460200	3.55920800	-4.88662500
H	-5.53147500	-1.06862900	-6.90251500
H	-2.58755700	-2.29863200	-4.41033800
H	-1.92688000	-1.66552200	-2.88220700
H	-4.94960000	-2.09001200	-1.18145900
H	-3.18451300	-1.96854600	-1.12174000
H	-2.99445300	0.59843300	-3.54662600
H	-4.48852500	0.23166600	-5.16265700
H	1.78799300	-2.08550500	-8.49348700
H	-0.41517600	-1.59633700	-6.57445800
H	2.32112800	6.80229300	-4.95561100
H	3.47622100	7.71805100	-4.01277900
H	1.71188600	7.99937500	-2.19960500
H	2.17127100	6.33495100	-2.53008100
H	0.81789500	8.88791200	-4.99592200
H	-0.53518900	9.93464100	3.48290900
H	0.68838200	9.17629100	4.47617600
H	1.89014600	7.35459000	3.71702700
H	-1.85011100	8.27309200	1.83105000
H	2.35543200	5.64510700	1.96769400
H	-1.38413900	6.57542500	0.09112900
H	0.24292200	5.83006400	-1.49691200
H	-2.34647300	8.83991400	4.70222000
H	-5.84236400	7.79488500	4.50627600
H	-4.22938400	7.18899900	4.16659200
H	-7.77158600	6.16557300	3.23776100
H	-3.71075200	6.68196000	1.95239900
H	-8.37891000	5.33582700	0.98384600
H	-4.32567600	5.91530900	-0.30109100
H	-7.69229400	4.83171500	-1.13038600
H	-6.52805600	5.69744800	5.53553100
H	-2.48621600	1.87284400	5.07106000
H	-2.03649400	1.03628400	2.16040100
H	-1.16747900	0.37059200	3.53261800
H	-2.92502100	0.15208000	3.39768700
H	-1.27227300	3.92694900	4.66943000
H	-0.23535100	2.53286800	4.37534600
H	-1.52093000	4.31044300	2.23043700
H	0.17793700	4.16958900	2.63307200
H	-0.61142400	2.80841600	1.88631200
H	-3.57109000	3.22421900	2.53177200
H	4.23486300	-6.15633300	6.31370800
H	5.46101500	-7.11972800	5.51066300
H	5.62066000	-7.13685600	3.16468900
H	1.95697900	-5.81586200	4.98763400
H	4.80642400	-6.33857000	0.95968500
H	1.15721800	-5.02216100	2.78787500
H	2.59323400	-5.23128900	0.78116100
H	3.80834900	-9.06481600	5.41958600
H	10.23638700	0.76726300	1.74555200
H	10.01351300	-0.71962400	2.64514800
H	8.36666100	0.50302900	4.13705500
H	7.40991900	2.65926500	3.30286400
H	8.60375200	2.69573500	2.00679100
H	9.11792500	2.86701300	3.68986800
H	6.52410600	0.43362400	2.44681700
H	7.69712000	0.45649300	1.13691900
H	7.61742800	-0.93918500	2.21835000
H	12.08830500	0.52345600	3.28359300
H	6.42222400	7.16207200	3.76542100
H	7.84839500	7.27730700	4.76370600
H	8.73485300	4.44984900	5.58671500
H	7.23331300	2.64570100	6.48183600
H	4.18455700	6.31215800	3.97377900
H	2.13359900	5.09475600	4.63305000
H	4.45084500	2.11510200	6.71361100
H	2.25829900	3.03239300	5.98355300
H	7.70857000	5.70882300	2.12254700
H	6.66186800	-5.42144300	-4.65472200
H	6.77347400	-6.59001200	-3.35528700
H	6.87274800	-5.51224600	-0.86324800
H	5.61484200	-3.37636600	-4.37087700
H	5.52190200	-3.96919900	0.54146300
H	4.35985100	-1.80747400	-2.96470100
H	3.26079300	-1.70790300	-0.89409900

H	8.91262500	-4.43474400	-3.84593000
H	7.96824500	-3.47554200	2.03371700
H	7.26077800	-5.06181000	2.24589000
H	7.09133300	-4.76243400	4.65741200
H	8.30548900	-1.97992400	4.22135900
H	7.56202900	-2.53701700	5.72482300
H	9.04291700	-3.30297600	5.13629800
H	5.92415400	-2.38076100	3.11279300
H	5.33799400	-2.99060400	4.66692800
H	5.20423700	-3.99296100	3.21788100
H	10.04689100	-4.17078600	3.21097300
H	8.85857800	1.55354600	-1.31445200
H	8.89224000	2.89498100	-0.17250400
H	6.38607700	4.07859400	0.48618000
H	4.08138300	1.00267100	-1.25400900
H	8.76590900	2.95121000	-3.21619000
H	5.56340600	5.74916300	-2.32630900
H	7.03677200	3.88424200	-3.99842500
H	1.48539000	1.71704800	-2.65162500
H	1.89135700	3.09907800	-1.63912600
H	0.02482000	2.96492600	-3.73162300
H	3.49649700	2.18814100	-3.57057000
H	3.81843700	4.30995700	-2.18507500
H	4.78131900	0.64775100	-6.22346700
H	6.28765800	-0.19753300	-6.37469800
H	7.25113000	2.23023700	-7.11910300
H	1.69448000	-8.05764600	-3.40677800
H	3.09491600	-8.69951300	-4.24225300
H	4.37053000	-6.59548600	-3.52011300
H	1.97363300	-6.25114800	-1.64929400
H	2.62635300	-5.04961900	-2.78919400
H	3.56729200	-5.51513900	-1.39823300
H	4.68031000	-8.86023600	-2.47820000
H	4.89080400	-7.54524800	-1.30572800
H	3.39489400	-8.48699400	-1.31472900
H	1.67528200	-7.59008000	-5.88780900
H	-0.91689300	-4.07595300	-4.19464300
H	0.56147300	-3.95363000	-3.23065900
H	-0.02214300	-6.66906700	-4.03881100
H	-2.84487300	-5.01210600	-0.47550500
H	-1.85401400	-3.94583300	0.54343900
H	-0.37490100	-3.66917300	-1.18589300
H	0.15693700	-8.24966400	0.57115600
H	-1.93653200	-7.19361000	-0.73498700
H	-2.33879600	-6.09717000	-7.92664800
H	-1.68705900	-5.96286900	-6.30043900
H	-3.33388300	-4.74162600	-4.35669600
H	-5.29138700	-3.16406600	-4.78903100
H	-6.30121100	-3.57305400	-7.11366200
H	-3.51033600	-8.20193600	-7.34042800
H	-11.09148500	5.49799200	-2.76408300
H	-11.16688800	4.06479300	-3.76797000
H	-11.72321400	4.13803100	-0.76126300
H	-11.71199500	2.67199500	-1.76455900
H	-13.68014600	3.89635900	-3.30560300
H	1.37824200	0.58968600	-4.73470200
H	2.01675000	-2.36072400	-4.29406700
H	3.00732700	-0.94540700	-3.97938800
H	1.46387700	4.37550500	0.21835000
H	3.17528300	2.89406700	3.06495000

H	1.09854000	1.36797800	-0.35348700
H	-2.45416100	-2.35088100	1.81259500
H	-5.26637000	3.95129800	-1.98123300
H	6.10175000	0.78996400	-2.98162300
H	-0.93488100	1.34999400	-0.28201200
H	-0.46750100	0.24877100	-1.58572000
H	-2.19960200	0.37814900	-1.10477300
H	4.00125300	3.07594700	0.21435300
H	-0.53120900	-1.54673900	-4.22589800
H	-13.57829700	5.44048600	-2.43046300
H	-13.11327500	5.35360700	-4.15369900
H	-5.31615800	4.83588700	4.59214000
H	-4.83485600	5.79547900	6.05592300
H	-4.35028600	2.00116600	3.56514400
H	-3.93279000	3.65045900	4.20638900
H	-1.23741600	7.65551400	5.40128800
H	-1.08596200	9.38373500	5.89635700
H	-4.60835200	4.72881200	-4.66972800
H	-3.03776500	5.22630800	-5.42098100
H	2.18908500	9.82059400	-4.41200600
H	2.30171700	8.92589600	-5.94682300
H	8.40026500	7.32352200	2.36250900
H	9.13761600	5.89486900	3.14709600
H	5.83363800	6.37866600	-3.95306000
H	2.50246100	3.17402900	-4.61189800
H	9.83307100	3.86625800	-2.12388700
H	5.54499600	2.51385400	-7.54617400
H	6.45242600	1.11418100	-8.25188300
H	0.20938600	-1.42641400	-8.97939900
H	0.32380800	-3.10464000	-8.42707500
H	-4.82847100	0.28395000	-7.79890200
H	-4.52258600	-1.39420800	-8.33446900
H	-3.23602500	-7.89175700	-5.60769300
H	-1.84439400	-8.23281800	-6.66050700
H	3.20577600	-6.68663200	-5.71607500
H	8.94889200	-6.10823300	-4.46775400
H	8.99945700	-5.81437600	-2.71988500
H	-1.16642900	-9.18695600	-0.13341700
H	-1.08058400	-8.83936700	1.63573300
H	2.66859200	-8.09218200	6.36285200
H	4.28118500	-8.52831200	7.04297700
H	9.67108500	-5.37670500	1.97543000
H	9.31536800	-5.71143400	3.67168900
H	11.06495000	0.23450700	4.66583000
H	11.14934700	1.86502100	3.89499300
H	-2.45590500	2.73992400	0.63619300
H	-7.20340900	1.52030100	0.67082600
H	-8.76189800	-0.08131700	-0.86555000
H	-9.21094100	-1.64304400	1.02783800
H	-8.75562900	-5.44938000	4.05649200
H	-8.85002000	-5.93939400	2.35724100
H	-7.28835000	-5.44496500	3.04057500
H	-9.70636100	-3.58063600	2.80001000
H	-6.98039800	-2.91648200	3.37544500
H	-7.64436700	2.17699800	-1.48407500
H	-8.97137700	-3.48960900	8.61004400
H	-7.92285800	-4.84271100	8.27532300
H	-0.97097900	-2.91041200	3.91308600
H	-1.40135800	-4.60848600	3.84078800
H	-4.68674000	-3.88196300	5.04863900

H	1.02009200	-0.84263300	3.32944000
H	-4.92049600	-2.03420900	1.29623900
H	-8.14844000	-3.24962300	5.16445500
H	0.13661700	-2.33237900	6.90945500
H	1.75435700	-2.19606700	9.05653900
H	4.32877300	-3.00748700	7.58735300
H	4.22405500	-1.87008300	8.94684900
H	3.84880200	-3.59117700	9.18793600
H	-9.87096500	-5.55988900	9.72001300
H	-9.57929700	1.68501300	3.33869600
H	-8.74431200	2.86384700	2.34477400
H	-7.82371000	1.62913200	3.26777500
H	-11.00118000	-5.02094500	8.45781500
H	-9.93597300	-6.38727900	8.14977500
H	-8.83766100	-4.27498100	0.59127800
H	-7.19613800	-3.83612600	1.05749600
H	-5.71317600	0.09697300	-1.03923100
H	-6.86252700	0.03609800	-2.37013900
H	2.24527200	-4.00981400	6.64181900
H	-2.54255600	-4.39162700	6.09325500
H	1.58373000	-4.52831700	8.17989900
H	-2.00865000	-2.71107500	6.18564000
H	2.66100400	-1.49455200	6.48722200
H	2.52463500	0.88753000	4.36384100

E:HPA-8 (+15.4)

C	8.65579700	2.07606900	-2.89890900
C	8.78652300	1.15089400	-1.71456700
O	9.77579200	0.43228400	-1.54785800
N	7.68539200	1.09565600	-0.92011500
C	7.71320000	0.34949300	0.33192000
C	7.45115800	-1.13960900	0.02548400
O	6.34321300	-1.68184100	0.18203800
C	6.66037800	0.97357800	1.26626100
O	6.64007400	2.37842100	1.13799700
N	8.52687500	-1.79722800	-0.43845800
C	8.49731400	-3.19495600	-0.82651500
C	9.02074300	-3.46704600	-2.24408100
C	8.99044400	-4.97187300	-2.52487000
O	8.29357500	-2.73115100	-3.22544200
C	10.17395200	-5.61110500	-8.20636300
C	9.06292400	-4.71817200	-7.65437000
C	9.30239200	-4.27308000	-6.21865700
O	10.30541700	-4.55537900	-5.58553900
O	8.28833100	-3.54629300	-5.74988800
C	1.59750500	-4.13991900	-4.94449600
C	0.50346000	-4.76950500	-5.79743500
O	0.31144400	-5.98383700	-5.84677200
C	2.76151100	-5.10176500	-4.65917600
C	3.83805200	-4.45522400	-3.84991300
C	5.10742500	-4.02763400	-4.16025400
N	3.65110500	-4.09494600	-2.52583700
C	4.78820400	-3.48745100	-2.09087700
N	5.68713500	-3.42858000	-3.05913500
N	-0.25871600	-3.87513100	-6.47977100
C	-1.51077800	-4.21302500	-7.11704500
C	-1.96885600	-3.03323400	-7.98894800
C	-3.41093100	-3.21443100	-8.45583300
O	-1.78648700	-1.79693100	-7.30749100
N	4.12476500	4.01335500	1.24959600
N	2.55782300	2.70299600	0.57131800
N	3.63174400	0.63836000	5.31742800
N	4.03455700	-1.42912300	1.65584300
N	0.31278900	-0.11912300	4.56983300
N	-0.41263000	-0.84325700	7.04608600
N	-6.75176600	2.94143000	-6.36319200
N	-6.11402200	1.66714000	1.36818200
N	-5.02437700	2.64612500	-0.27362800
N	-7.16668600	4.91632500	2.97080900
N	-3.80909700	4.51514100	2.86606600
N	0.07375800	-5.44877200	4.88118600
N	1.97967500	-4.37733900	2.02184300
N	2.10645400	-7.08927200	0.49779400
N	4.91572500	-3.86293200	7.84142600
N	5.27550700	-3.04299400	5.80291400
C	3.39204600	5.31044600	4.20323400
C	2.83502600	5.60443000	2.79553700
C	2.95088100	4.46153300	1.84987500
C	1.95902200	3.62142300	1.41901700
C	3.87132400	2.96385200	0.47137400
C	4.73775000	0.28574800	7.46139100
C	3.53971000	0.05005800	6.54588900
C	2.70871800	0.35201700	4.22271400
C	1.27218000	0.82339600	4.55737100
C	2.82640300	-1.11218000	3.73544100
C	4.13571300	-1.30791200	2.99614600
C	-1.08253700	0.13250200	4.88708400
C	-1.41956600	-0.26521400	6.33981800
C	-2.02998000	-0.61247200	3.92945200
C	-0.59488900	-2.67721300	0.17867000
C	-0.57015500	-1.18798800	8.44221400
C	-2.17164100	9.41294700	4.13801200
C	-2.58551700	8.06206000	3.53816400
C	-1.83237300	7.66710900	2.26389000
C	-0.38769000	7.18118600	2.50202000
C	0.94253300	8.43135600	-5.83773800
C	-0.20800400	8.80444100	-4.87128500
C	-0.44453500	7.93622100	-3.65711400
C	0.46345400	7.86612000	-2.58823000
C	-1.65213200	7.23982100	-3.51544000
C	0.19627400	7.12703700	-1.43980300
C	-1.94473400	6.51058200	-2.36689900
C	-1.02518900	6.42230000	-1.30713000
C	5.26560300	5.63375700	-5.72021500
C	5.02154100	6.87356500	-4.82439500
C	5.42220000	6.58437500	-3.38902400
C	6.74351300	6.26521000	-3.04286800
C	4.46154300	6.52907800	-2.37165600
C	7.09671500	5.88326700	-1.74913700
C	4.79555100	6.16458900	-1.06804400
C	6.11680300	5.81958800	-0.74284400
C	3.52595900	2.81050800	-3.81706100
C	2.13603000	2.21705600	-4.10508100
C	1.10145400	3.30799200	-4.44741400
C	1.66430800	1.32963500	-2.94257200
C	0.87206100	4.36133200	-3.36211300
C	-3.42063500	-8.71907700	-5.33930500
C	-4.03287000	-7.40109900	-4.82165600
C	-3.32755800	-6.86029800	-3.59078600

C	-4.01714800	-6.65985800	-2.38755600
C	-1.95437500	-6.57211900	-3.62573600
C	-3.36079800	-6.18328100	-1.24893400
C	-1.28928500	-6.12082200	-2.48350300
C	-1.98984200	-5.92276300	-1.28947800
C	-11.13404900	0.20903500	-3.67366800
C	-9.97162200	-0.04403600	-2.70153400
C	-8.57414100	0.38246000	-3.18884600
C	-8.47293600	1.89751800	-3.40227500
C	-7.49668500	-0.08447400	-2.19980500
C	-8.42411000	5.95428100	-3.33594800
C	-7.55087300	6.10644600	-4.57650200
C	-7.00078700	4.82712600	-5.13987000
C	-7.59280700	4.00229400	-6.06294800
C	-5.71370700	4.24250000	-4.84266800
C	-5.58521000	3.06851200	-5.63809300
C	-4.64171000	4.62040100	-4.01550900
C	-4.41746500	2.29985500	-5.64977400
C	-3.48046300	3.85548600	-4.01693800
C	-3.36858600	2.71371900	-4.83598500
C	-8.31832900	-5.24732500	4.22102100
C	-6.77924400	-5.30483900	4.17732600
C	-6.13138100	-4.32115200	3.23200100
C	-5.54044100	-3.15893000	3.74154400
C	-6.10268000	-4.51010600	1.84274500
C	-4.93314900	-2.22397400	2.91449400
C	-5.47667300	-3.59213300	0.99753500
C	-4.87070400	-2.44452500	1.53242400
C	-9.11252900	-5.33357800	-2.36870100
C	-7.75032500	-4.64906900	-2.26556900
C	-7.14881500	-4.23670000	-3.61823400
C	-8.09028300	-3.33722300	-4.43074600
C	-5.79655500	-3.55233300	-3.41221400
C	-8.90425400	3.55832100	2.00945700
C	-7.99598400	4.77421900	1.90656300
C	-8.49082300	2.50773300	0.94648500
C	-7.01789900	2.42304200	0.63916700
C	-6.34428000	3.03953800	-0.38982900
C	-4.92849300	1.81578300	0.80060800
C	-5.94762700	5.70274900	2.90413900
C	-4.88092000	4.86457700	3.60563400
C	-2.92080900	3.45559500	3.31639500
C	-1.67398400	3.32077000	2.42924600
C	-6.30112300	2.34382000	7.24203700
C	-5.82790700	1.34651000	6.17693200
C	-6.31468400	1.64395600	4.77927400
C	-1.95721600	-6.37939800	5.84310700
C	-1.12673700	-5.15234900	5.47628200
C	-2.32935400	-7.22962200	4.60340300
C	-3.23258100	-6.52371300	3.56760300
C	-2.51721700	-5.45430700	2.72987400
C	-3.87789700	-7.56049900	2.63974100
C	0.80547000	-4.44854000	4.13651500
C	1.73291200	-5.12269100	3.12832900
C	2.85256600	-4.85186800	0.95640100
C	2.13484300	-5.83601200	0.00534100
C	1.26674400	-8.24013000	0.12759600
C	3.18220300	-6.83912100	7.26156100
C	2.90188200	-5.32961900	7.41589000
C	3.98845000	-4.40142700	6.96797000
C	4.20292100	-3.90237600	5.70110700
C	5.65783000	-3.04995100	7.11022000
C	-2.89387000	-1.86309000	-3.77037100
C	-3.08575800	-0.48884300	-3.67367800
C	-2.78552300	0.14970600	-2.47103800
C	-2.42349000	-2.60629600	-2.69398700
C	-2.15385500	-1.94066500	-1.50378500
C	-2.32068900	-0.56499700	-1.36725900
C	12.95061200	5.75611300	2.64058500
C	11.46486000	5.39739600	2.38709900
C	11.13890400	4.41601700	1.24792700
C	9.61247900	4.20676600	1.11042500
C	0.42620300	-1.65401200	-0.25348600
O	2.56837200	-0.62762800	6.87612700
O	1.07610900	2.02274600	4.75660400
O	5.23204300	-1.30294900	3.57336300
O	-2.55516300	-0.08809500	6.77681400
O	0.22265300	7.51706300	3.52484300
O	0.12038800	6.44627600	1.56314200
O	-1.32927400	5.69141600	-0.23599100
O	6.40551400	5.43483200	0.52690600
O	-4.25203900	-1.57465000	0.70424000
O	-7.97288400	5.50148100	0.91150400
O	-5.05789900	4.47687500	4.76016500
O	-0.49566700	3.81150800	3.06439700
O	-7.27562600	2.36239400	4.52269300
O	-5.63763500	0.96775700	3.86029500
O	-1.49780900	-3.99674600	5.63804700
O	2.24841900	-6.22719600	3.34675700
O	1.64869700	-5.47097500	-1.07267700
O	-3.18506300	-2.47505600	-4.97549400
O	-1.78009200	-2.75711700	-0.44431400
O	9.15459800	3.05139000	1.32910900
O	8.93977000	5.23893400	0.80489600
O	-0.37102700	-3.49913100	1.05102600
S	-1.71972200	-0.30339200	2.14034300
H	3.34129600	6.48054400	2.37298600
H	1.77841200	5.87241900	2.84031500
H	0.91451000	3.61807000	1.71338200
H	4.60074600	2.43821200	-0.11677800
H	3.17295900	4.28317700	4.51116600
H	5.64710100	-0.04729100	6.95003000
H	2.78976200	-1.77693000	4.60162100
H	1.99114200	-1.34697600	3.06956600
H	4.89718100	-1.46718200	1.09538300
H	3.13671700	-1.34443300	1.20752200
H	3.00353800	1.01940800	3.40707700
H	4.51141000	1.05859600	5.05944500
H	-1.63741000	-1.20120000	8.66594200
H	0.53584300	-0.79256500	6.69442600
H	-2.42139200	7.27769200	4.28489300
H	-3.66496100	8.07112800	3.32980600
H	-1.78240000	8.51447500	1.56408500
H	-2.37207700	6.88058300	1.72927100
H	-1.08370300	9.44484300	4.22560500
H	-0.05096100	9.84565200	-4.55409900
H	-1.13711100	8.81496300	-5.45468700
H	-2.39000300	7.28361500	-4.31451900
H	1.40062300	8.41764700	-2.64883400
H	-2.90047200	6.01350200	-2.24403100

H	0.89818100	7.09990300	-0.61339200
H	-0.68520200	6.00068400	0.60339600
H	1.92384400	8.69158300	-5.42871300
H	5.55895700	7.74574000	-5.21913700
H	3.95717900	7.13190400	-4.84162600
H	7.51920000	6.30580700	-3.80502400
H	3.42637800	6.77609300	-2.59634000
H	8.12519300	5.65473000	-1.48860800
H	4.03218900	6.15809400	-0.29818100
H	7.41816900	5.28549600	0.66310800
H	6.30031400	5.59637000	-6.07668900
H	2.22539200	1.57230100	-4.99148000
H	1.53732600	1.93033200	-2.03461600
H	0.69659000	0.86258100	-3.15822200
H	2.38793100	0.53396700	-2.72927500
H	1.42902300	3.81107100	-5.36733800
H	0.14636200	2.82253100	-4.69328700
H	1.79539000	4.88750600	-3.10540700
H	0.15726300	5.11516200	-3.69650700
H	0.47205200	3.93318700	-2.43786600
H	3.53395900	3.31681500	-2.84350900
H	-3.98850900	-6.65598100	-5.62856500
H	-5.09596500	-7.54224900	-4.59517300
H	-5.08271300	-6.87279000	-2.34330000
H	-1.38979000	-6.70308000	-4.54354100
H	-3.91723400	-6.00503600	-0.33419900
H	-0.22210300	-5.92612700	-2.51531200
H	-1.48191200	-5.53740200	-0.41181300
H	-3.45400600	-9.48441500	-4.55667500
H	-10.17498400	0.47666600	-1.75531500
H	-9.94656000	-1.11368300	-2.45414900
H	-8.38976800	-0.11112100	-4.15630400
H	-7.46262100	2.18576200	-3.70336700
H	-8.70284500	2.43174400	-2.47311900
H	-9.15894300	2.25896500	-4.17395800
H	-6.49038800	0.13575400	-2.57055700
H	-7.60128800	0.42194200	-1.23541000
H	-7.55698500	-1.16173400	-2.01526200
H	-12.08235700	-0.05278700	-3.18849300
H	-6.70774500	6.76851600	-4.34364900
H	-8.12570400	6.62370600	-5.35552200
H	-8.55799600	4.08737500	-6.54167700
H	-6.96967600	2.18273900	-6.98623000
H	-4.71101700	5.51292800	-3.40094500
H	-2.63382500	4.16366800	-3.41349300
H	-4.32343100	1.42608800	-6.28881600
H	-2.43949400	2.15317500	-4.83570600
H	-7.91514100	5.40051400	-2.54145500
H	-6.39035600	-5.11583700	5.18479400
H	-6.47806600	-6.33029000	3.92393400
H	-6.56412400	-5.39786500	1.41288700
H	-5.55415700	-2.97766100	4.81340000
H	-5.42746200	-3.75402900	-0.07480900
H	-4.52143100	-1.31441700	3.32794000
H	-3.53015900	-1.10586400	1.21508900
H	-8.68189500	-4.22086700	4.31318300
H	-7.83217500	-3.76108200	-1.62477200
H	-7.04002100	-5.31604400	-1.75976700
H	-6.98044100	-5.15747900	-4.20003300
H	-8.35773300	-2.44061600	-3.86260300
H	-7.60682000	-3.00771200	-5.35710100
H	-9.01782700	-3.84902600	-4.70490800
H	-5.89432800	-2.65156600	-2.79811200
H	-5.36968200	-3.24506900	-4.37002200
H	-5.08532700	-4.21492900	-2.91049100
H	-9.88251900	-4.64401000	-2.72851600
H	-8.84231400	1.52180800	1.26662100
H	-8.98379100	2.72997300	-0.00424800
H	-6.67675800	3.71230300	-1.16230300
H	-4.00695400	1.34911200	1.13007100
H	-8.83739400	3.11253200	3.00166500
H	-5.72473900	5.90867400	1.85395400
H	-7.18650000	4.20221500	3.69751700
H	-1.55704800	2.26695500	2.15053300
H	-1.78163600	3.91315900	1.51301000
H	-0.18504500	3.16447500	3.72246600
H	-3.48174800	2.51316100	3.35280700
H	-3.75270000	4.80672800	1.90121600
H	-4.74061200	1.23007800	6.16493400
H	-6.21459200	0.34060200	6.39560600
H	-7.26522900	2.77082200	6.95632200
H	-1.42275800	-7.59645200	4.10152700
H	-2.85164400	-8.12313200	4.96933600
H	-4.03749900	-6.02158200	4.11887200
H	-1.63482700	-5.86832000	2.22692600
H	-2.20485500	-4.59760800	3.32497100
H	-3.18906200	-5.06074600	1.96299000
H	-4.43082500	-8.32351700	3.20059400
H	-4.57430600	-7.07180500	1.95197500
H	-3.11809300	-8.07242500	2.03520600
H	-1.39379900	-6.99738400	6.55377200
H	1.42747400	-3.83020300	4.79923500
H	0.10429100	-3.77780300	3.62947600
H	0.36626400	-6.40479300	4.73748500
H	3.72704700	-5.32401600	1.40961100
H	3.18961900	-3.98756000	0.38383000
H	1.20357000	-3.77242300	1.72273800
H	0.20361800	-7.98409700	0.19340900
H	2.41887800	-7.12583600	1.46609500
H	2.67426800	-5.10356600	8.46292000
H	1.99514800	-5.10109000	6.84453800
H	3.72913600	-4.10499200	4.75492800
H	5.54939800	-2.39650000	5.06114500
H	6.47945800	-2.44954700	7.47480100
H	3.88036700	-7.21343600	8.01505600
H	10.90268900	6.31855400	2.19900400
H	11.05573300	4.98118800	3.31753900
H	11.51259600	4.81907200	0.29771300
H	11.61364300	3.44482700	1.41731000
H	13.56506700	4.86779900	2.82184700
H	-1.24115100	1.20857600	4.80189400
H	-1.96570500	-1.68656600	4.13803100
H	-3.03484900	-0.28962100	4.20344900
H	-3.15648200	-3.43372800	-4.84494800
H	-3.48779500	0.06158400	-4.51311400
H	-2.28125600	-3.67989400	-2.75135800
H	2.83976300	-4.37329600	-1.96491200
H	5.05607100	4.46631600	1.25621300
H	-5.99193700	1.17804000	2.93081600
H	0.26256000	-1.26665300	-1.25835900

H	0.35178000	-0.83412900	0.47196700
H	1.41178800	-2.12346500	-0.19680800
H	-4.27146800	2.93122500	-0.88270300
H	0.55335200	-1.07758900	4.37579700
H	13.38955200	6.32306300	1.81342200
H	12.94560100	6.37290900	3.54489300
H	5.12701400	4.77835300	-5.05036500
H	4.60155300	5.58435400	-6.58990700
H	4.27530000	2.01362400	-3.76468100
H	3.79186200	3.58175000	-4.54756500
H	0.94339400	7.35804600	-6.05393200
H	0.75443200	9.02769800	-6.73668400
H	4.47354000	5.47977600	4.18951100
H	2.88956200	5.98355300	4.90641000
H	-2.50939600	10.25951700	3.53056900
H	-2.59923700	9.51517700	5.14101400
H	-8.66703300	6.94308400	-2.93553700
H	-9.34796300	5.42003800	-3.57827800
H	-6.02663900	6.66372700	3.42301300
H	-2.57395300	3.66037900	4.33479500
H	-9.93787900	3.86613900	1.82247100
H	-5.57592200	3.15701800	7.34768600
H	-6.42137400	1.78508500	8.17524300
H	-0.08335700	-0.43924800	9.07686300
H	-0.13536200	-2.16384100	8.67835000
H	4.87955400	1.34511800	7.70298800
H	4.64158400	-0.28848200	8.39009600
H	3.58863900	-7.04789800	6.26847800
H	2.21567600	-7.34471400	7.35791500
H	-2.89486500	-6.07896400	6.32238100
H	-8.66345200	-5.83163600	5.08022600
H	-8.73327200	-5.69879800	3.31303700
H	1.54095400	-8.91079400	0.94921100
H	1.42970300	-8.72095700	-0.84290200
H	-2.36904300	-8.54632100	-5.58418400
H	-3.96764300	-9.10103700	-6.20783800
H	-9.44613000	-5.71108800	-1.39618900
H	-9.08708600	-6.18358600	-3.05862100
H	-11.06685800	-0.42066600	-4.56665700
H	-11.20910000	1.26814500	-3.94453100
H	2.07768800	1.97269400	0.06695300
H	7.12210400	1.93895200	-0.85891000
H	8.70895400	0.45627700	0.77674400
H	9.30960500	-1.20387900	-0.72360400
H	9.45548800	-5.17954000	-3.48981100
H	9.53544100	-5.52651000	-1.75266800
H	7.95554800	-5.32977100	-2.53964600
H	10.05484100	-3.10742300	-2.31634600
H	7.31470600	-2.91059500	-3.09456800
H	7.57903200	2.73346500	1.27146700
H	8.94013500	-3.81280500	-8.26138200
H	8.08986700	-5.22293600	-7.68510900
H	2.36418100	-5.98821500	-4.15427800
H	3.19087900	-5.44633700	-5.60425400
H	5.64459800	-4.10780300	-5.09465600
H	-2.13248900	-0.08545700	-0.41416800
H	4.94138000	-3.09977300	-1.09315900
H	8.45010000	-3.24931500	-4.79849200
H	-0.12929000	-2.88096600	-6.33488700
H	-1.30273700	-2.97697200	-8.85823600
H	-4.09004900	-3.23631700	-7.59622800
H	-3.70410600	-2.38141800	-9.09959500
H	-3.53507300	-4.14909700	-9.01359600
H	9.96348600	-5.89570100	-9.24178800
H	9.52156000	1.90070500	-3.54068000
H	8.63975500	3.14656400	-2.66528800
H	7.76188100	1.80024500	-3.46562800
H	11.13696100	-5.09541700	-8.17342600
H	10.27390000	-6.52080100	-7.60839600
H	9.10241300	-3.78163900	-0.12212900
H	7.46562500	-3.53995100	-0.73545800
H	5.66887400	0.60663200	0.98395400
H	6.84674800	0.63553800	2.29355700
H	-2.27996400	-4.44351500	-6.36322500
H	1.96967900	-3.22338400	-5.41529500
H	-1.43039900	-5.12226000	-7.72277600
H	1.13039400	-3.83498500	-3.99821600
H	-2.35426600	-1.83077500	-6.51484300
H	-2.94292600	1.22021800	-2.39576600

E:4HR

C	-8.84074300	2.19428900	2.45447500
C	-8.94889600	0.93555300	1.62666800
O	-9.79458700	0.07455700	1.90300800
N	-8.00392200	0.75136100	0.66800100
C	-8.06675600	-0.40992900	-0.21834100
C	-7.51548200	-1.66442800	0.49442300
O	-6.44314000	-2.19144800	0.15856100
C	-7.23857100	-0.09603700	-1.48893500
O	-7.30655300	1.26158600	-1.84015200
N	-8.25919500	-2.16526800	1.49769000
C	-7.80218700	-3.32344700	2.24716300
C	-8.11184200	-3.28405400	3.74696900
C	-7.50449800	-4.51995600	4.41657200
O	-7.64971900	-2.08354400	4.36694400
C	-9.79333100	-3.64590100	9.84291600
C	-8.92527900	-2.61372700	9.11632900
C	-8.82946800	-2.82591600	7.60833700
O	-9.45115700	-3.68650000	7.00829800
O	-7.98901300	-1.95786100	7.04672000
C	-1.34596200	-1.76279600	5.83583200
C	-0.06956400	-2.34044900	6.43167400
O	0.37891400	-3.42350100	6.06539400
C	-1.38162300	-1.84040700	4.29882500
C	-2.77845800	-1.66872100	3.77871500
C	-3.99799200	-1.89917100	4.37367700
N	-3.09199800	-1.24254300	2.49376500
C	-4.44998100	-1.21846600	2.38144200
N	-5.02996900	-1.61989700	3.50005200
N	0.50048100	-1.62172800	7.44579000
C	1.79677300	-1.99132500	7.98504000
C	2.52945000	-0.78211700	8.59091100
C	3.99325500	-1.12563600	8.85940900
O	2.40216900	0.39677500	7.80720700
N	-4.63654700	2.78359300	-2.30273700
N	-3.05627800	1.56272200	-1.35981400
N	-3.76413100	-1.21673800	-5.28697400

N	-3.99689400	-1.98613300	-1.15065300	C	2.79673700	-5.08528000	4.97392700
N	-0.30529400	-1.45023900	-4.33168100	C	4.28719100	-6.09461000	2.84643400
N	0.24654300	-3.03636100	-6.51712600	C	2.28377700	-5.04106500	3.67948000
N	6.94166900	4.94210300	5.08529100	C	3.02575700	-5.54737900	2.61038000
N	5.88345200	1.21539100	-1.94141600	C	11.02512200	1.68253000	3.03442200
N	4.60061200	2.78007600	-1.03513900	C	9.88951200	1.06284300	2.20821700
N	6.65324800	3.98160400	-4.55768600	C	8.45599200	1.48941700	2.56812300
N	3.32380300	3.48114000	-4.19870900	C	8.20737100	2.97483700	2.28830900
N	0.05002300	-6.66108400	-3.35650700	C	7.43905500	0.63509700	1.80158300
N	-1.50213100	-4.73920900	-0.81600700	C	7.97484100	6.92431200	1.11668700
N	-1.68570100	-7.02682000	1.83970100	C	7.12303600	7.37537000	2.27569800
N	-4.95675300	-6.39048000	-6.16042100	C	6.79008700	6.35733700	3.32963000
N	-5.25266600	-5.04577700	-4.40885800	C	7.65441800	5.71036800	4.17697900
C	-3.92168100	3.45280700	-5.46808700	C	5.45965500	5.96496300	3.73040600
C	-3.33492400	4.00606300	-4.15776700	C	5.58997900	5.08441000	4.84034600
C	-3.47039500	3.07225200	-2.99538800	C	4.17154200	6.29771800	3.27577100
C	-2.50567200	2.29369400	-2.39745000	C	4.47991300	4.57284900	5.51971100
C	-4.34122800	1.89135000	-1.32783800	C	3.06613200	5.78818100	3.94318000
C	-5.01939900	-2.37233800	-7.05495000	C	3.22242100	4.94873300	5.06427700
C	-3.74135900	-2.22725800	-6.20498400	C	8.40542100	-6.01101500	-2.79768200
C	-2.75652500	-1.05111900	-4.23637100	C	6.87400000	-6.01729100	-2.65449800
C	-1.36132500	-0.74909800	-4.83997600	C	6.38748800	-4.80027100	-1.90141000
C	-2.76558100	-2.21064500	-3.22014600	C	6.28948400	-3.57047800	-2.56276700
C	-4.07512400	-2.28845100	-2.45806300	C	6.09714300	-4.82602300	-0.53277800
C	1.01634400	-1.33326900	-4.91133900	C	5.93831000	-2.40672200	-1.89095800
C	1.16588300	-2.07586600	-6.25730800	C	5.72224800	-3.67205500	0.15807600
C	2.10796100	-1.97654000	-4.04608900	C	5.65979200	-2.45216000	-0.52146800
C	1.47612700	-2.43411300	-1.38251600	C	9.31855100	-4.10524700	3.48862300
C	0.34840300	-3.78959200	-7.74737200	C	7.88116000	-3.64096000	3.20370200
C	1.39361100	7.65738300	-6.79413100	C	7.14848600	-2.96686300	4.37792500
C	1.88261800	6.53387200	-5.86407100	C	7.92736900	-1.77431300	4.94837400
C	1.22526600	6.44867100	-4.48019900	C	5.73565000	-2.53975400	3.95419500
C	-0.25661900	6.09851100	-4.50383700	C	8.50966200	3.07720400	-3.30347500
C	-1.47461400	9.52960600	3.11453300	C	7.53410000	4.22163600	-3.55078100
C	-0.34075100	9.60563300	2.05487900	C	8.17005600	2.36949300	-1.96040000
C	-0.08764000	8.35922700	1.23282500	C	6.68682600	2.31066600	-1.63866400
C	-1.00858500	7.90597900	0.27862100	C	5.89015900	3.28045500	-1.05887500
C	1.09207300	7.61857700	1.38796600	C	4.64709200	1.53991100	-1.56054100
C	-0.77052800	6.76372800	-0.47790500	C	5.39832200	4.70446900	-4.67985900
C	1.35252100	6.47053400	0.63516200	C	4.34596700	3.66740600	-5.07135800
C	0.41689900	6.03017500	-0.31004700	C	2.50888000	2.28102000	-4.30285500
C	-5.62302700	6.59890800	3.98262100	C	1.31414900	2.29073000	-3.36268800
C	-5.46558700	7.54691100	2.76779900	C	5.87695200	0.23616900	-7.84674000
C	-5.90789400	6.82943300	1.50995600	C	5.46527900	-0.41747900	-6.52422400
C	-7.24766000	6.46693900	1.31663800	C	5.93633800	0.32805700	-5.30393800
C	-4.96980400	6.35121600	0.58730400	C	2.10136900	-7.89043100	-3.78847800
C	-7.64129200	5.63467300	0.27195200	C	1.34361300	-6.56755100	-3.76740400
C	-5.34084900	5.52259400	-0.46998200	C	2.47793100	-8.44942500	-2.40434000
C	-6.67976800	5.13254700	-0.62330600	C	3.46899800	-7.61172500	-1.57878000
C	-3.75094300	3.43546800	2.93953000	C	2.85504500	-6.31585100	-1.03738700
C	-2.37616700	3.09276700	3.53920800	C	4.02862900	-8.45206000	-0.42421300
C	-1.51046100	4.35516800	3.67805300	C	-0.73202900	-5.49021000	-3.01373300
C	-1.68282500	1.99939900	2.73267500	C	-1.43675100	-5.76916700	-1.68958600
C	-1.20483400	5.03618900	2.34997100	C	-2.02672600	-4.98047100	0.51604100
C	3.93872100	-6.78284500	7.52304800	C	-1.09276800	-5.93545600	1.27950400
C	4.59374100	-5.71043000	6.63144300	C	-0.90091000	-8.15152700	2.33619200
C	4.05600900	-5.64457200	5.22288700	C	-3.03080300	-9.02902100	-4.82282500
C	4.79609900	-6.14618800	4.14621800	C	-2.87288600	-7.63327500	-5.46109500

C	-3.97767700	-6.65031400	-5.21876000
C	-4.15290100	-5.82501300	-4.12763100
C	-5.68948800	-5.41902200	-5.64353100
C	-0.35312900	0.28339300	0.34184600
C	0.39950400	-0.58588600	1.13738200
C	1.67264100	-0.19119000	1.53972700
C	0.20316300	1.50076200	-0.07672400
C	1.50811300	1.90796600	0.32450600
C	2.24019700	1.03417700	1.19162500
C	-13.45835800	3.84046200	-3.76331600
C	-11.99133800	3.51847000	-3.42987900
C	-11.69812100	3.13345900	-1.97062600
C	-10.20301500	2.79960100	-1.81014200
C	2.14458700	-2.93743000	-0.14152400
O	-2.75939100	-2.95734900	-6.34668600
O	-1.22541600	0.10739300	-5.70684100
O	-5.13963400	-2.60814600	-3.01075800
O	2.16259400	-1.86114900	-6.94769600
O	-0.97262600	6.22897100	-5.47778000
O	-0.78118900	5.69008500	-3.34060700
O	0.61793900	4.93450400	-1.09053800
O	-7.01016500	4.28673900	-1.63029000
O	5.30087300	-1.33378000	0.17905600
O	7.50449500	5.23055900	-2.84482200
O	4.47154100	3.01411500	-6.10428200
O	0.25803800	3.11113000	-3.85439700
O	6.86056100	1.13052400	-5.29649700
O	5.28847800	-0.04354700	-4.20022200
O	1.86739100	-5.50062400	-4.08559200
O	-1.92876500	-6.87987800	-1.46280000
O	0.10972100	-5.69568400	1.35566900
O	-1.63445100	-0.11570500	0.05147200
O	2.03297400	3.05557600	-0.10207600
O	-9.80726000	1.73875500	-2.36696900
O	-9.48304400	3.63312900	-1.18124900
O	0.38901700	-2.83813200	-1.78461000
S	2.46792900	-1.32171800	-2.36890200
H	-3.78357800	4.97860100	-3.92437300
H	-2.27179700	4.20454200	-4.27549900
H	-1.45613900	2.26055900	-2.64159500
H	-5.08218800	1.52689500	-0.63686700
H	-3.69871000	2.38142100	-5.54689800
H	-5.86797800	-2.58299300	-6.39817000
H	-2.61809800	-3.15182000	-3.75576500
H	-1.95413600	-2.07261500	-2.50350400
H	-4.87085900	-2.00006400	-0.61880400
H	-3.15373600	-1.58041600	-0.76358800
H	-3.02129100	-0.12620800	-3.71171700
H	-4.65347800	-0.77683900	-5.10288700
H	1.40215100	-3.82657900	-8.02719000
H	-0.66591100	-2.98873600	-6.07443800
H	1.72189900	5.57314600	-6.36585700
H	2.96725600	6.62050600	-5.71932800
H	1.31753800	7.39828900	-3.93420300
H	1.73912800	5.69488800	-3.87526500
H	0.30502400	7.62635100	-6.86072900
H	-0.54514700	10.45376900	1.38719700
H	0.58991100	9.85606400	2.57837300

H	1.82645100	7.94606800	2.12073300
H	-1.93784700	8.45082200	0.12763400
H	2.25743300	5.89251200	0.79578900
H	-1.49947600	6.42111800	-1.20197300
H	-0.12553000	5.31615800	-2.70689000
H	-2.46768700	9.60506900	2.66286400
H	-6.02991200	8.47556200	2.92515300
H	-4.41281500	7.83255400	2.66252000
H	-8.00265600	6.82614900	2.01368000
H	-3.92033000	6.61162400	0.70739500
H	-8.67910600	5.35164300	0.13237300
H	-4.59319300	5.15489600	-1.16352600
H	-7.95905300	3.94755500	-1.50866900
H	-6.64909100	6.61359700	4.36494000
H	-2.55354400	2.71555300	4.55628600
H	-1.62374300	2.26911600	1.67771200
H	-0.65929200	1.81189600	3.06916400
H	-2.24096200	1.06440700	2.79909900
H	-2.02729000	5.06235600	4.34321800
H	-0.56920700	4.09625100	4.17864100
H	-2.12036000	5.31120900	1.82031500
H	-0.62725100	5.94920100	2.49598400
H	-0.62786700	4.38395100	1.69031800
H	-3.70767900	3.58358200	1.85701100
H	4.47320800	-4.73259400	7.11601700
H	5.67349700	-5.90073200	6.59328800
H	5.78075300	-6.57190800	4.32695600
H	2.20436800	-4.67701300	5.78589200
H	4.87331700	-6.48563600	2.02003900
H	1.29988500	-4.62185100	3.50248700
H	2.60625700	-5.52409200	1.61419900
H	3.98790400	-7.77172700	7.05826200
H	10.05531500	1.29194300	1.14594500
H	9.95870300	-0.02990500	2.29250900
H	8.31216800	1.31322300	3.64596900
H	7.19789200	3.27416100	2.58377000
H	8.30975200	3.18528200	1.21727300
H	8.90831000	3.62107400	2.82291900
H	6.41473500	0.88603500	2.08806400
H	7.52508300	0.80590600	0.72282000
H	7.58261500	-0.43475100	1.97903600
H	11.98559100	1.34786000	2.62069700
H	6.17860300	7.77530200	1.88773400
H	7.61318600	8.23389000	2.75816400
H	8.73367800	5.73906800	4.21655300
H	7.34707200	4.30740500	5.75192100
H	4.04130500	6.94688500	2.41468400
H	2.06796300	6.03069400	3.59489200
H	4.59200400	3.91135300	6.37404100
H	2.34183300	4.57790200	5.57365500
H	7.49451400	6.12450200	0.54401700
H	6.41082500	-6.03933000	-3.64844300
H	6.55792300	-6.93262600	-2.14132600
H	6.16265600	-5.76590400	0.01007900
H	6.48996400	-3.51831200	-3.63016500
H	5.47750000	-3.70745400	1.21292900
H	5.86475600	-1.47179700	-2.42836300
H	5.43405400	-0.55132200	-0.38206400

H	8.74581100	-5.04993500	-3.19578300
H	7.88751800	-2.95536600	2.34601900
H	7.28278800	-4.50399200	2.88707600
H	7.04766200	-3.71793900	5.17731400
H	8.09003500	-1.01162500	4.17915800
H	7.37153000	-1.30205200	5.76632300
H	8.90639300	-2.06993900	5.33915900
H	5.77379200	-1.80483200	3.14238500
H	5.19454800	-2.08412600	4.79165600
H	5.14636300	-3.39390400	3.61117900
H	10.00511800	-3.26025500	3.60115900
H	8.56255600	1.34904100	-1.98169200
H	8.66440100	2.88971000	-1.13518400
H	6.13180900	4.27268900	-0.71589600
H	3.77439900	0.91051900	-1.64659200
H	8.46095800	2.35169300	-4.11551700
H	5.19230500	5.20095200	-3.72786700
H	6.70112000	3.08572000	-5.03832600
H	0.97458700	1.25869600	-3.21189000
H	1.58196200	2.67992600	-2.37623000
H	-0.15840400	2.64761100	-4.59600400
H	3.13698300	1.40282100	-4.10029600
H	3.40587800	3.89035400	-3.27760800
H	4.38459600	-0.57892400	-6.46746900
H	5.91108900	-1.41845800	-6.42996000
H	6.81972400	0.77466300	-7.73031500
H	1.56491100	-8.60584600	-1.81265700
H	2.90812100	-9.44631900	-2.56618100
H	4.30370900	-7.34004800	-2.23909400
H	1.95194100	-6.51383100	-0.44857500
H	2.60265200	-5.62418500	-1.84066000
H	3.57530200	-5.81006500	-0.39113400
H	4.49279000	-9.37826600	-0.78242400
H	4.78269800	-7.89179700	0.13741600
H	3.23330800	-8.72255700	0.28070400
H	1.47599100	-8.63396200	-4.30033400
H	-1.49288600	-5.28218600	-3.77678000
H	-0.06303900	-4.63460900	-2.96023900
H	-0.32176800	-7.53796500	-3.01543700
H	-3.04421400	-5.37259800	0.44194800
H	-2.06723400	-4.02683400	1.04838700
H	-0.85611700	-3.96406200	-0.95867400
H	0.14884000	-7.90419700	2.16100100
H	-2.62716900	-7.22048200	1.53197300
H	-2.75259000	-7.73970700	-6.54441800
H	-1.93754100	-7.19986200	-5.08982900
H	-3.64159400	-5.76364000	-3.18134600
H	-5.48837200	-4.19867600	-3.88457800
H	-6.54313700	-4.95630700	-6.11803600
H	-3.71509000	-9.66720000	-5.38930200
H	-11.37248100	4.38865800	-3.68566800
H	-11.64264200	2.69814400	-4.06687000
H	-11.96291500	3.95241800	-1.29418500
H	-12.28719400	2.24914300	-1.69845000
H	-14.10713800	2.96759800	-3.63102400
H	1.23874100	-0.28321800	-5.11065600
H	1.92408400	-3.04969400	-3.94246600
H	3.05420800	-1.85024000	-4.57240100
H	1.18313800	4.18106400	-0.64933800
H	-0.01361500	-1.54676700	1.42600200
H	-0.37686500	2.18947800	-0.68026300
H	-2.45501700	-0.89561800	1.77038600
H	-5.55977600	3.21756200	-2.38058400
H	5.67906900	0.42688500	-3.39305600
H	2.72564200	-2.15488000	0.34387700
H	1.42052900	-3.38935000	0.53704100
H	2.85582800	-3.70335400	-0.46757500
H	3.71192800	3.12193700	-0.59310100
H	-0.40378900	-2.08166200	-3.54815600
H	-13.84743600	4.65101400	-3.13659500
H	-13.50774000	4.16149200	-4.80889400
H	-5.44254200	5.59670700	3.58428500
H	-4.94223000	6.84401700	4.80680800
H	-4.45081500	2.62411400	3.14806500
H	-4.05560300	4.37735300	3.39702100
H	-1.41077400	8.57224500	3.63735500
H	-1.30995900	10.37761400	3.79057000
H	-5.01074800	3.56596500	-5.46681900
H	-3.47417100	3.91611800	-6.35566500
H	1.68812900	8.66319000	-6.47690000
H	1.79278400	7.48083800	-7.79953300
H	8.15127200	7.75897000	0.43348000
H	8.93299900	6.53255500	1.47252900
H	5.41134800	5.47228900	-5.45998900
H	2.13056000	2.15581000	-5.32249100
H	9.52417100	3.48332900	-3.24817900
H	5.09980600	0.94189000	-8.15957100
H	6.01123000	-0.56652700	-8.57721900
H	-0.19463900	-3.28767300	-8.55511200
H	-0.03097200	-4.81204400	-7.66923100
H	-5.23100500	-1.44027100	-7.58939100
H	-4.90691100	-3.18965600	-7.77335000
H	-3.40454800	-8.95253200	-3.79632200
H	-2.03649300	-9.48477700	-4.79771800
H	2.97520700	-7.72666400	-4.41332600
H	8.77239000	-6.79349000	-3.46263600
H	8.86600100	-6.14206900	-1.81317100
H	-1.14707400	-9.04848400	1.75791800
H	-1.01307700	-8.33141500	3.40999900
H	2.85328900	-6.55186600	7.74382700
H	4.47942900	-6.80312200	8.46184700
H	9.67293300	-4.71909200	2.66007700
H	9.37121400	-4.69871000	4.40416000
H	11.02709000	1.34404100	4.07386200
H	11.05131600	2.77923200	2.97723300
H	-2.17130600	0.59631000	-0.46449900
H	-7.62226100	1.57409100	0.20351600
H	-9.11619700	-0.58975300	-0.48150200
H	-9.06186500	-1.60612600	1.79496100
H	-7.80542400	-4.56239800	5.46408700
H	-7.84372400	-5.44005900	3.92728100
H	-6.41152500	-4.47925500	4.35033100
H	-9.19734900	-3.29032700	3.90318900
H	-6.72970200	-1.87983600	4.04036500
H	-8.25702000	1.48173700	-2.10371600
H	-9.30650000	-1.59716700	9.27429800

H	-7.90257300	-2.60330600	9.51133600
H	-0.72219700	-1.07957300	3.86744100
H	-0.97184200	-2.81296900	3.99939400
H	-4.20367800	-2.24175700	5.37672000
H	-4.96488300	-0.92484500	1.48105100
H	-7.93081600	-2.06800800	6.04876900
H	0.24242400	-0.64874200	7.53620700
H	2.03950300	-0.52116100	9.53745400
H	4.51013100	-1.34858400	7.91840000
H	4.49819000	-0.28311200	9.33844800
H	4.08459200	-2.00382500	9.50744300
H	-9.83663400	-3.42647500	10.91402200
H	-9.67689700	2.16999300	3.15670500
H	-8.89862000	3.14995300	1.91691900
H	-7.92706700	2.14556700	3.04577100
H	-10.81031100	-3.64599100	9.44261300
H	-9.39263300	-4.65446800	9.70946000
H	-8.24963900	-4.23901000	1.83485100
H	-6.72347000	-3.40034000	2.09542100
H	-6.19163800	-0.33348900	-1.29639300
H	-7.56847600	-0.76616200	-2.29337300
H	2.39221300	-2.45270200	7.18764400
H	-2.15451200	-2.38020900	6.24308600
H	1.72186800	-2.73292900	8.80829300
H	-1.52632000	-0.73884300	6.17240100
H	2.79064200	0.23726600	6.93481100
H	2.25067200	-0.86400400	2.16859200
C	3.57912000	1.40843100	1.78373700
H	4.24193800	0.53450200	1.74137000
H	4.04868100	2.18361400	1.18360200
C	3.55301300	1.93633300	3.23192700
H	4.55225500	2.33220300	3.45249700
H	2.88321100	2.79913900	3.28354500
C	3.19121100	0.90299600	4.31993900
H	3.75249100	1.16072900	5.23327700
H	3.56040900	-0.08379500	4.01181200
C	1.70211400	0.80466500	4.69322500
H	1.10677000	0.60500300	3.79897000
H	1.55123600	-0.06324000	5.34992100
C	1.16941800	2.06256600	5.38846900
H	1.09021400	2.86458000	4.64912200
H	1.89583800	2.40211500	6.13715400
C	-0.18139100	1.86339300	6.07469600
H	-0.06173200	1.24902100	6.97274900
H	-0.61714700	2.81646900	6.38936500
H	-0.90081900	1.37873100	5.40984200
